# (NHC)Pd(II) hydride-catalyzed dehydroaromatization by olefin chain-walking isomerization and transfer-dehydrogenation

**DOI:** 10.1038/s41467-022-33163-6

**Published:** 2022-09-20

**Authors:** Weihao Chen, Yang Chen, Xiao Gu, Zaizhu Chen, Chun-Yu Ho

**Affiliations:** 1 Guangdong Provincial Key Laboratory of Catalysis, Southern University of Science and Technology (SUSTech), Shenzhen, China; 2 Shenzhen Grubbs Institute, Department of Chemistry, Southern University of Science and Technology (SUSTech), Shenzhen, China; 3 Department of Chemistry, Southern University of Science and Technology (SUSTech), Shenzhen, China

**Keywords:** Homogeneous catalysis, Synthetic chemistry methodology

## Abstract

Transition-metal-catalyzed homogeneous dehydrogenation and isomerization are common organic molecular activation reactions. Palladium hydrides are good olefin isomerization catalysts but are usually short-lived species under redox-active dehydrogenation conditions. Here, we show that Pd-H in the presence of an N-heterocyclic carbene ligand and an alkene regulator enables transfer-dehydroaromatization, avoiding the homo-disproportionation pathway. The desired product is obtained with up to 99:1 selectivity, and the exo-to-endo olefin isomerization can be carried out in one pot. In contrast to previously reported methods that rely on the efficient removal of Pd-H, the approach reported herein benefits from the steric effects of the N-heterocyclic carbene and the choice of alkene to regulate the competing reactivity of allylic C‒H activation and hydropalladation. This method circumvents the challenges associated with tedious olefin separation and a low exo-to-endo olefin isomerization ratio and expands the scope to include challenging endo- and exo-cyclic olefins under mild, neutral, and oxidant-free conditions. Overall, herein, we provide a strategy to synthesize (hetero)aromatic compounds via chemoselective dehydrogenation of cyclic alkenes over ketones and the dehydrogenative Diels-Alder reaction of a cyclic enamine.

## Abstract (web summary)

Aromatic compounds can be prepared via dehydrogenation of cyclic compounds. Here the authors report the dehydroaromatization of endocyclic and exocyclic olefins via chain-walking isomerization and transfer-dehydrogenation catalyzed by palladium N-heterocyclic carbene complexes in the presence of alkenes as sacrificial reagents.

## Introduction

**Fig. 1:**
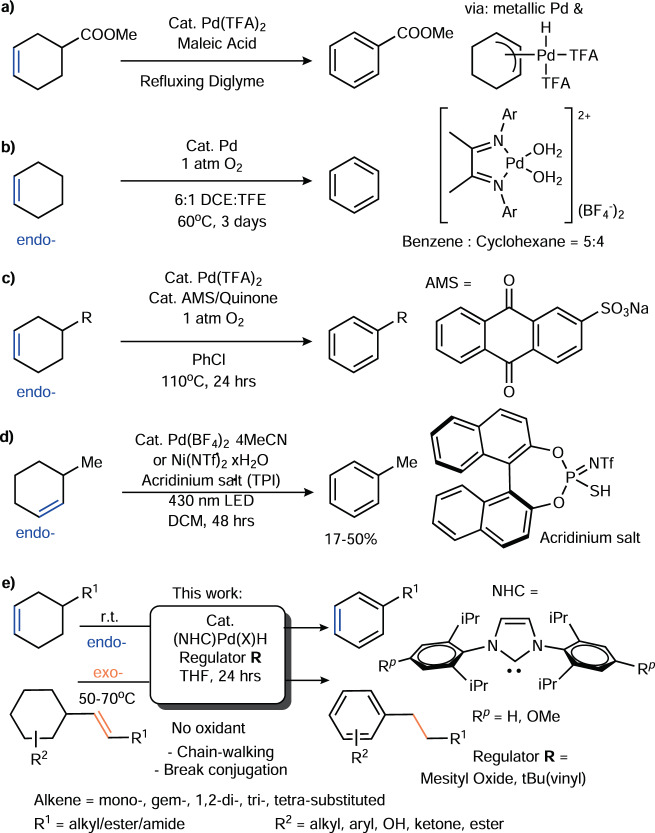
Strategies in Pd-Catalyzed Dehydroaromatization (DHA). **a** Conventional dehydroaromatization. **b** Pd-H recycled/removed by O_2_. **c** AMS/quinone-assisted Pd(II) regeneration. **d** Photoredox catalytic cycles via thiyl radicals. **e** NHC-Pd-H directed transfer-dehydroaromatization.

Dehydrogenation (DH)^1–4^ and chain-walking functionalization^5,6^ are two essential strategies for molecular activation. These methods add value to abundant natural resources, such as carbonyls, alkenes, Diels-Alder adducts, and alkanes^7^. In particular, DH of cyclic compounds by transition-metal catalysis has long been vital for aromatic compound synthesis^8–11^ (Fig. 1a, dehydroaromatization (DHA))^12–18^. Transition-metal hydrides are common intermediates in catalytic cycles. A longstanding fundamental challenge has been the precise control of transition-metal hydride behavior; otherwise, disproportionation and hydrometallation often limit the yield and efficiency of this process. Certain innovative approaches, such as the use of Ir, Pt, Cu, and Pd catalysts with pincer and diimine ligands^7,19–24^, as well as Pd/C heterogeneous systems, have been developed to eliminate unproductive hydrometallation that can cause undesired transformations or slow catalyst regeneration. Some of these schemes can be implemented by using inexpensive oxidants or O_2_ for the efficient removal of M-hydrides (Fig. 1b, c)^20,25–27^. Photoredox-active transition-metal catalysis can be used to effectively prepare naphthalenes and N-heterocycles at r.t.^28,29^, where less activated cyclohexenes have not yet been utilized as good substrates (Fig. 1d). An alkene can be used as a gaseous H_2_ acceptor to enable DHA of 1,3-diones and cyclohexa-1,4-dienes catalyzed by Pd/C^30,31^, B(C_6_F_5_)_3_^32^ and alkaline-earth metals^33^ at 60–130 °C. However, the aforementioned powerful strategies also completely block some desired functions of transition-metal hydrides, such as olefin isomerization. Some conditions, such as high temperature and highly redox-active environments, limit the substrate scope to specific endocyclic olefins. Many exocyclic olefins that can be accessed easily from either natural resources or standard 1,n-diyne/diene cycloisomerizations have not yet been utilized as ideal substrates. It is challenging to develop a dual-function catalyst or to combine two catalysts in one pot to carry out both exo-to-endo alkene isomerization and DH. Different redox potentials of the catalysts and reagents may negatively affect the desired elementary steps. Branched and conjugated exocyclic olefins may result in additional complications, such as a high styrene-to-ethylbenzene ratio that is frequently observed when using 4-vinyl-cyclohexene, as well as subsequent hydrogenation by high-pressure steam to afford high ethylbenzene selectivity^34^. The high relative yield of the styrene product also implies that the Pd-H generated in the redox-active catalytic cycle is short-lived.

**Fig. 2:**
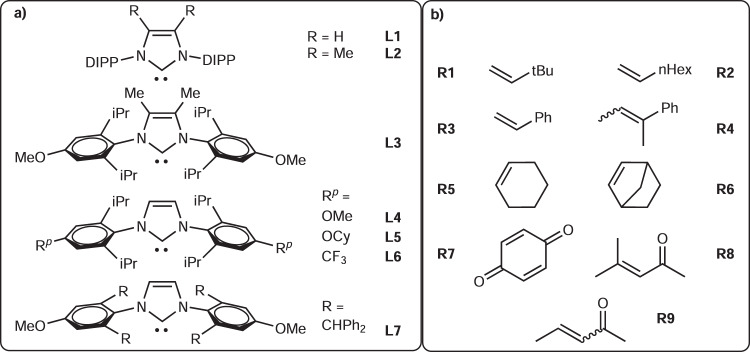
N-Heterocyclic carbene L and regulator R employed in this study. **a** NHC **L1-7** structures**. b** Regulator **R1-9** structures.

In this work, instead of developing more practical Pd-H removal methods by oxidation or reductive elimination, we employ an alternate strategy that relies on the conservation of Pd-H. Herein, NHC^35^ stabilizes Pd(II)H to perform transfer-dehydroaromatization (TDHA) for several challenging exo- and endocyclic olefins under mild and neutral conditions (Fig. 1e. endo- at r.t.; exo- at 50–70 °C, without a strong oxidant or cocatalyst). Unlike approaches based on fast Pd(II)H removal by an oxidant/acid/base, the desired olefin isomerization and DH can be carried out simultaneously in one pot facilitated by suitable selections of NHC **L** and regulator **R** (Fig. 2). This dynamic combination provides an efficient way to control hydropalladation vs. allyl DH for a broad range of exo- and endo-olefins.

## Results

### Catalyst development and optimization

**Fig. 3:**
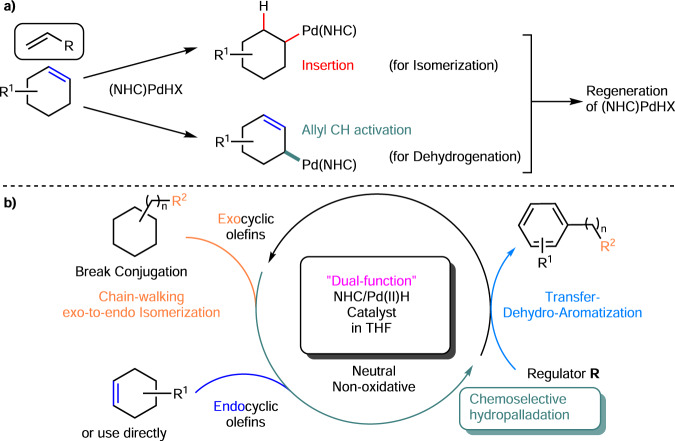
Catalytic action of (NHC)Pd(II)H. **a** Cooperative steric effects of NHC and the alkene enable interchangeability of the Pd(II)H reactivity. **b** Conversion of a mechanism consisting of undesired disproportionation and hydropalladation into the desired TDHA mechanism.

An unexpected discovery resulted from our continuing efforts to use an NHC-ligated transition-metal hydride to catalyze olefin cross-hydroalkenylation^36–38^. An in situ-generated Pd(II)H catalyst was prepared from PdCl_2_/NHC/HSi(OEt)_3_, characterized by ^1^H NMR, and used instead of an (NHC)Ni(allyl)X catalyst to afford a notable quantity of product **2a** from endocyclic olefin **1a** and α-olefin **R1**^10^ (Fig. 1e, Table 1, Entry 1), and no cross-hydroalkenylation of **1a** and **R1** was observed even when the catalyst was treated with NaBArF (**1a** conv. 45%, 24% **2a**, 19% **4a**). The reaction was carried out simply under N_2_ in THF at r.t. in the presence of a catalytic quantity of a reductant rather than the stoichiometric quantity of an oxidant used for related CH activations (i.e., HSi(OEt)_3_ vs. PhIX_2_, Br_2_, CuCl_2_, or O_2_), in sharp contrast to the elegant redox-active catalytic cycles based on the reoxidation of Pd species by a common oxidant [Pd(0→II)^20,21^; Pd(II→IV)^39,40^]^41–45^. Indeed, control experiments (Entries 2 and 3) further showed that our reaction did not proceed effectively in O_2_ or without HSi(OEt)_3_. No oxidation of olefin **1a** to the corresponding alcohol/ketone was detected. Thus, the catalytic formation of **2a** did not involve the sequential dehydration of a Wacker-oxidation product in the presence of NHC/PdCl_2_/THF/O_2_^46,47^, and allylic alcohols were not intermediates in the mechanism. This conclusion was supported by the successful formation of the phenol product and the lack of formation of the arene product (benzene) from allylic alcohol (2-cyclohexenol) and 3-cyclohexenol (**1e**) under standard conditions (as detailed below). The formation of an (NHC)Pd(II)H intermediate species was also supported by the detection of disproportionation product **3a** and olefin isomerization product **4a**. These observations revealed a unique opportunity to convert a mechanism consisting of undesired disproportionation^9^ (e.g., 4-*t*-butyl-cyclohexene disproportionation mediated by Pd(TFA)_2_ in the absence of an external oxidant, which was attributed to the loss of H-TFA) and hydropalladation into the desired TDHA mechanism with optimal isomerization under mild and neutral conditions. Here, we hypothesize that the steric effect of NHC may enable the desired chemoselective transfer-DH and isomerization reactivity (Fig. 3). Furthermore, the electronic effect of NHC may suppress the undesired Pd(X)H reductive elimination to a metallic Pd(0) species. In particular, the reversible and chemoselective hydropalladation ability of the relatively more stable (NHC)Pd(II)H may catalyze olefin isomerization, as well as allyl DH, by forming an alkyl-Pd(II) species similar to Me-Pd(II)/Pt(II)^22^.

**Table 1:** Optimization of (NHC)Pd(II) catalyzed transfer-dehydroaromatization (TDHA)


Entry^a^	NHC	PdX_2_	R	Conv. 1 (%)	Yield 2 (%)	2:3	4 (%)
1	**L1**	PdCl_2_	**R1**	64	49	96:4	15
2^b^			**R1**	17	5	n.d.	11
3^c^			**R1**	16	3	n.d.	13
4			**R2**	35	<5	n.d.	25
5			**R3**	< 5	<5	n.d.	<5
6			**R4**	< 5	<5	n.d.	n.d.
7			**-**	100	30	34:66	10
8^d^			**R5**	53	1	3:97	8
9			**R6**	6	1	n.d.	4
10			**R7**	0	0	0	0
11			**R8**	33	22	71:29	2
12			**R9**	72	37	92:8	12
13	**L2**	PdCl_2_	**R1**	60	38	88:12	22
14	**L3**			88	71	91:9	10
15	**L4**			92	74	96:4	15
16	**L5**			71	46	92:8	14
17	**L6**			57	35	98:2	21
18	**L7**			27	7	98:2	16
19	**-**			<5	<5	n.d.	<5
20	**L4**	PdCl_2_	-	100	25	32:68	29
21^e^	**L1**	Pd(TFA)_2_	**R1**	90	64	93:7	21
22 ^f^	**L1**			36	13	91:9	21
23^e^	**L4**			65	39	92:8	25

^a^Standard condition: substrate **1a** (0.25 mmol) and regulator **R** (0.5 mmol) were added to NHC **L**/PdCl_2_ (0.025 mmol) with HSi(OEt)_3_ (0.0375 mmol) in 2 mL THF at r.t. and stirred for 24 hrs. The conversion of **1a** and selectivity and yield of product **2a** were determined by ^1^H NMR.

^b^Under O_2_ instead of N_2_.

^c^No HSi(OEt)_3_.

^d^~50% benzene based on **R5**.

^e^HBpin was used instead of silane.

^f^No HBpin or silane was used.

*n.d.* not determined.

The analysis presented above shows that the selection of substituents on **R** is crucial for achieving a good yield and a high **2**:**3** ratio (Fig. 2). Screening for acyclic alkene **R** confirmed that **R1** is not a molecular H_2_ acceptor^10,30^. Replacing **R1** with other simple α-olefins, such as 1-octene or aromatic olefins, resulted in a low conversion of **1a** and afforded the 1-octene isomerization product (Entries 4–6). These findings suggested that the isomerization reactivity of a linear olefin is highly competitive and that the hydropalladation of aromatic alkenes may limit the desired reactivity by benzylic stabilization (see below for the solution to this problem and its application). Using cyclic olefins with allyl CH as **R** (such as **1a** itself and cyclohexene (**R5**)) resulted in a shift in the product selectivity. **3a** was favored over **2a**, and ~50% benzene based on **R5** was produced (see Entries 7 and 8, c.f. 1, respectively). This set of results suggested that the undesired reduction of **1a** could be favored if an unsuitable **R** group was selected and that the ester moiety in **1a** is not an essential directing group for the desired reactivity. Norbornene **R6**, an excellent hydropalladation substrate that only contains a bridgehead allyl CH, was expected to be an excellent **R** group that would prevent **1a** homodisproportionation. However, **R6** exhibited only very limited reactivity (Entry 7 vs. Entry 9), possibly due to strong steric repulsion. In addition to **R1**, acyclic carbonyl-activated olefins are also possible, albeit less efficient, choices for **R** for this reaction (Entries 10-12). Overall, the structural features and properties of **R** were identified as the controlling factors for the desired reactivity and selectivity.

Screening of NHC revealed its critical role in this reaction as well (Fig. 2, Table 1, Entries 13-18). The choice of ligand was expected to be very important because cyclohexene is a common sacrificial olefin that produces Pd(II)H from (dcpe)Pd(II)Me for isomerization^48^. In the presence of NHC (Entries 1 and 13–18), the desired reactivity and high selectivity were generally observed. In contrast, the desired reactivity was not observed in the absence of NHC, even when **R1** and silane were used (Entry 19). The most significant advance was realized by adding an electronic activator at the N-aryl p-position of NHC (Entry 1 vs. Entry 15), where an electron donor was found to be the most effective (**L4**). Placing additional steric pressure at the core (**L2**), N-aryl p-position (**L5**), and o-position (**L7**) of NHC caused the reactivity to decrease. The enhanced performance obtained by using **L4** was attributed mainly to the increased σ-donating ability of NHC^49,50^ and partly to the optimal steric effect either at the NHC V%_bur_^51,52^ or the N-aryl p-position, since the donor may stabilize Pd(II)H against undesirable reductive elimination to Pd(0) and increase the hydropalladation selectivity (where the olefin **R1** is less electron-rich and smaller than **1a**). Adding **R1** to the reaction remains important even when using the electronically and sterically optimized NHC **L4**; otherwise, homodisproportionation remains the main pathway for DH (Entry 20). This result confirmed that the concomitant use of a hydride and **R1** with NHC/PdCl_2_ is key to achieving the desired reactivity and suggested that the direct DH of **1a** by (NHC)Pd(II)H to produce H_2_ is quite slow at r.t.

Based on our hypothesis, the desired reactivity should be obtainable from other precatalyst combinations that can produce (NHC)Pd(II)H; thus, we also explored the use of **L1**/Pd(TFA)_2_/HBpin (Entry 21). The reaction was catalyzed under the same physical conditions, as expected. This result did not correspond to background DH caused by Pd(TFA)_2_ because a considerably lower reactivity was observed in the absence of HBpin (Entry 22). Comparing the results obtained using the two systems showed that the silyl and Bpin groups are not actively involved in the catalytic cycle. These combinations may be useful for expanding the substrate scope or precatalyst combinations based on differences in the steric and electronic properties of the respective compounds. However, **L4** is not a good choice for Pd(TFA)_2_/HBpin. A significant decrease in the DH-to-isomerization ratio was observed (Entry 15 vs. Entry 23).

### Scope of endocyclic and exocyclic olefin TDHA

**Fig. 4:**
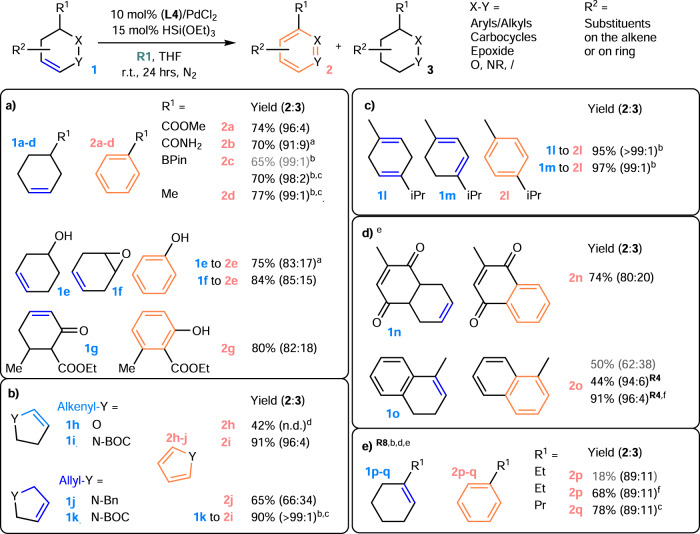
Endocyclic olefin 1 transfer-dehydroaromatization. **a** Cyclohexenes. **b** Heterosubstituted rings. **c** Dienes. **d** Fused rings. **e** Trisubstituted alkenes. Standard conditions: The olefin (0.25 mmol), **L4**/PdCl_2_ catalyst (0.025 mmol), HSi(OEt)_3_ (0.0375 mmol), and **R1** (0.5 mmol) were stirred in 2 mL of THF for 24 hrs at r.t. except if otherwise indicated. The yields of **2** and **2**:**3** (shown in parentheses) were determined by ^1^H NMR (average of two runs). ^a^48 h; ^b^as determined by GCMS (see the Supplementary Information for details); ^c^the standard condition shown in Fig. 5 was used; ^d^poor material balance due to a low b.p.; ^e^conversion, **1o**: 83; 47; 100%; **1p-q**: 25; 75; 89%; _f_50 °C.

With this basic information in hand, other endocyclic olefins were tested (Fig. 4). Several substrates with other functional groups and substituents were generally tolerated under the standard neutral condition with **L4** at r.t. (Fig. 4a)^21^, such as those substrates that can be obtained easily from Diels-Alder reactions (e.g., alkenyl ester/amide/Bpin/ether and dienes that afford **1a**/**1b**/**1c**/**1f**). No apparent decrease in the catalytic reactivity was observed even with a cyclohexene containing an acid-sensitive epoxide (**1f**). Phenol can be produced directly from a cyclohexenol (**1e**), an epoxide (**1f**) and an α,β-unsaturated ketone (**1g**)^15,53^ without the use of a protective or trapping group for OH. Moreover, aza-/oxa-heteroaromatics can be produced from endocyclic allyl-/vinyl-N/O (Figs. 4b, **1h-k**). The **2**:**3** ratio was increased by using an allyl carbamate instead of an amine (**1j** and 1**k**). Although elegant procedures have been developed for fused piperidine DH^28,54–58^, an N-substituted pyrrole (**2i**) was easily obtained instead of an N-oxide under our oxidant-free conditions. As conjugated dienes are formed as conspicuous intermediates before the final DHA step, the typical condition with **R1** remains effective (Fig. 4c).

The key advantage of our protocol is the use of logical combinations of NHC, X (e.g., TFA/Cl) and **R** to adapt to the structural characteristics of the substrate. Thus, a wide range of challenging alkenes can be used rather easily by simple modifications of the standard protocol. For instance, the relative hydropalladation efficiency between **R** and the substrates can be fine-tuned by a suitable choice and quantity of **R**. This selection can be guided by the literature on standard cross-hydroalkenylation (e.g., styrene is a better olefin acceptor than α-olefin in Ni(II)H-catalyzed cross-hydroalkenylation due to benzylic stabilization)^36,51,59^, thereby alleviating problems such as the low conversion and **2**:**3** ratio caused by the stabilization of the Pd(II) center after undesired hydropalladations at benzylic or tertiary carbon positions. Replacing **R1** in the aforementioned standard protocol with aromatic olefin **R4** and electronically activated trisubstituted olefin **R8** (mesityl oxide, which is an inexpensive, low-boiling-point, and activated trisubstituted olefin that is removable by vacuum) restored the high **2**:**3** ratio and reactivity at 50 °C by reacting a fused aromatic olefin and a trisubstituted olefin with **L1** (Fig. 4d, e, **1o**, **1p**, and **1q**). Replacing **R1** with **R8** in the initial condition including **L1** resulted in a larger reduction of **1a** (**2a**:**3a** = 71:29 vs. 96:4 in Table 1, Entry 11 vs. Entry 1), showing that **R8** is a less effective hydropalladation substrate than the disubstituted alkene **1a**; thus, it is unlikely that the successful result shown in Fig. 4e involved a tri-to-disubstituted olefin isomerization (**2**:**3** = 90:10) mechanism.

**Fig. 5:**
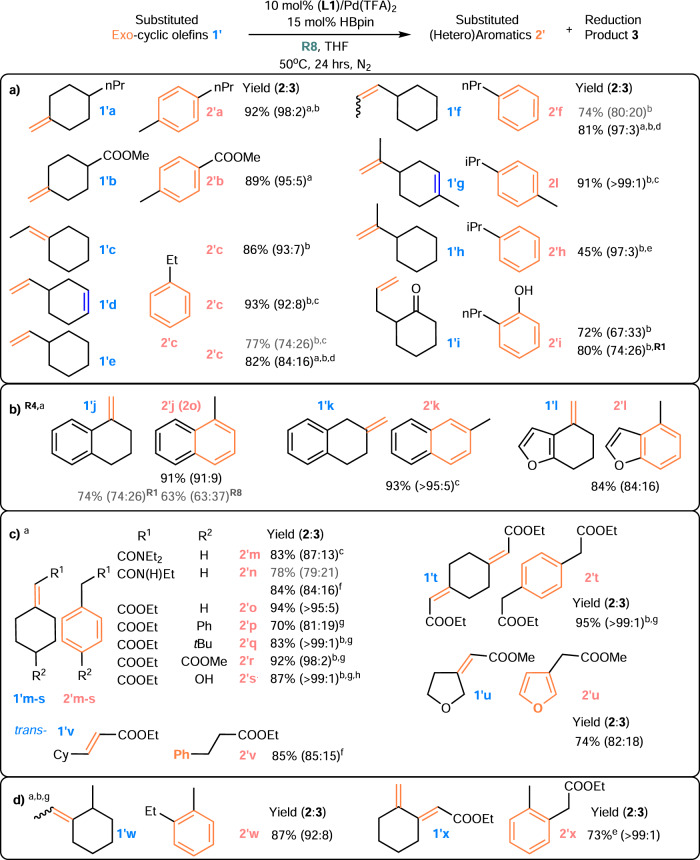
Exocyclic olefin 1′ transfer-dehydroaromatization. **a** Aliphatic exocyclic olefins. **b** Fused aromatic rings. **c** Exo-conjugated systems. **d** Unsymmetric cycloisomerization products. Standard conditions: The olefin (0.25 mmol), **L1**/Pd(TFA)_2_ catalyst (0.025 mmol), HBpin (0.0375 mmol), and **R8** (0.5 mmol) were stirred in 2 mL of THF for 24 h at 50 °C, unless otherwise indicated. The yields of **2** and **2**:**3** (shown in parentheses) were determined by ^1^H NMR (as the average of two runs). ^a^48 h; ^b^as determined by GCMS (see the Supplementary Information for details); ^c^60 °C; ^d^2.5 equiv. of **R1**; ^e^Other products are from olefin isomerization; ^f^3 equiv. of **R8**; ^g^70 °C; ^h^with a 9% aromatic product without OH.

One of the most remarkable aspects of this discovery is the ability of the NHC-Pd hydride to facilitate both olefin isomerization and TDHA in one pot for a suitably chosen **R**. The DH of 4-vinylcyclohexene has often been reported to produce a mixture of styrene and ethylbenzene in low yield and with low selectivity (41% and 14%, respectively)^24^. Our dual-function catalytic system limits undesired disproportionation, enables chain-walking alkene isomerization, and expands the substrate scope to many exocyclic olefins with diverse structural characteristics (Fig. 5). In general, reasonably high yield and selectivity were achieved using **L1**/Pd(TFA)_2_/HBpin/**R8** at 50 °C instead of **L1**/PdCl_2_/HSi(OEt)_3_, probably by altering the isomerization reactivity. Exocyclic olefins with diverse structural characteristics, e.g., methylene-, vinyl- and allyl-olefins with different degrees of substitution, including tri-/tetra-substituted olefins generated by chain walking, are all compatible (Fig. 5a). **R4** was used (Fig. 5b) rather than **R1** and **R8** to address a similar challenge related to aromatic substituents, as previously discussed (**1o** in Fig. 4d), to produce fused (hetero)arenes in both a high yield and **2′**:**3′** ratio, as predicted. Remarkably, as suggested by the **R8** structural features, this method is also applicable to certain exocyclic *α*,*β*-conjugated carbonyl systems by disrupting the conjugation (Fig. 5c). This ability is expected to be useful in combination with [(allyl)Pd(II)Cl]_2_-catalyzed α,β-DH of an ester/amide^60,61^ system to develop a one-pot synthesis of an aromatic product under appropriate conditions. Moreover, the good functional group tolerance of the system is maintained. Substituents such as OH, COOMe, and CONHEt were found to be compatible, and no trans-esterification was observed (**1′s, 1′r**, and **1′n** in Fig. 5c). However, the **2′**:**3′** ratio decreased when tBu was replaced with a Ph group, showing that the decrease in the **2**:**3** ratio in the Ph case was likely caused by styrenyl formation (**1′p** and **1′q** in Fig. 5c vs. **1o** in Fig. 4d) and not steric hindrance. **R8** is an optimal **R** group for both trisubstituted endocyclic olefins and exocyclic systems. This result suggests that trisubstituted olefin is a common starting point for the desired TDHA. However, the generally higher yield and **2′**:**3′** ratio obtained from conjugated systems indicate that the carbonyl group may offer a more acidic allyl CH for direct activation. In addition to substituted methylene-cycloalkane structures accessible via typical 1,6-diene cycloisomerization, unsymmetrical 1,7-diene/diyne cycloisomerization products are also effective substrates for this system at 70 °C (Fig. 5d).

**Fig. 6:**
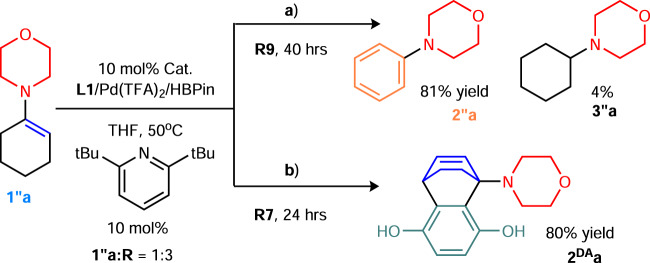
Catalytic synthesis of a substituted aniline and dehydrogenative Diels-Alder product. **a** Synthesis of a substituted aniline. **b** Trapping the diene for the Diels-Alder reaction.

Replacing **R8** with *β*-monosubstituted ketones (**R7**/**R9**) in our modular system enables the catalytic synthesis of substituted aniline and Diels-Alder derivatives from the corresponding enamine **1″a** (Fig. 6, c.f. **2″a:3″a** ~37:63 with **R8**; ~50:50 with **R1**, 10 mol% of base, where no DH background was observed in the absence of our catalyst). These successful results were notable for easily bypassing highly competitive reactions (e.g., Michael addition and cyclic amine DH) and enabled identification of the preferred allyl DH site for our catalyst. Note that although **R7** (BQ) is a stoichiometric oxidant that is frequently used in several redox-active DH systems, the dehydrogenative Diels-Alder product has rarely been observed when using **R7**. Hence, under redox-neutral and mild conditions with NHC, it is now possible to trap the in situ-generated endocyclic diene using challenging dienophiles or carry out other useful reactions, and the molecular complexity can be appropriately increased, as for acyclic diene systems^62^. This reaction sequence complements other one-pot reactions that primarily rely on aromatization first^63^.

## Discussion

**Fig. 7:**
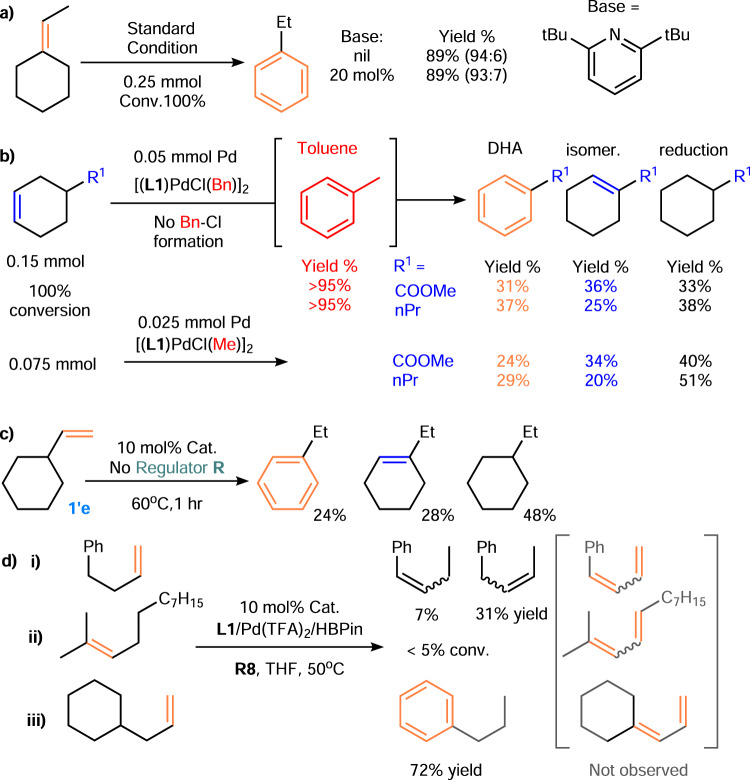
Control experiments and mechanistic studies. **a** Exocyclic olefin DHA using excess base. **b** Using [(**L1**)PdCl(**R**)]_2_ as an initiator in a mimic of the CH activation step at 50 °C for 24 h. **c** Disproportionation by **L1**/Pd(TFA)_2_/HBpin in the absence of the regulator **R**. **d** Role of the cycloalkane in allylic dehydrogenation.

A lower operating temperature, a broader substrate scope, and a higher isomerization ability than those of several well-known systems based on simple Pd(TFA)_2_ were demonstrated for the proposed NHC system (e.g., **1a** at r.t., c.f. 162 °C in maleic acid^9^ and 105 °C in AMS/PhCl^21^). While the current system was compatible with several known inhibitors^9^ and useful for exocyclic olefins **1′** at ~50 °C, reported Pd(TFA)_2_ systems afforded a <5% yield upon applying the known conditions to exocyclic olefins **1′o** and allylcyclohexane, with or without NHC. These results prompted us to investigate the functions of NHC, silane/borane, and alkene **R**. First, the functions of silane/borane were studied. It might be reasonable to attribute the isomerization to the trace quantity of acid in the system (c.f. limonene isomerization by HCl/TFA^64^), which was obtained via X-Si(OEt)_3_/-Bpin hydrolysis during catalyst generation or the reductive elimination of H-PdX^9^. However, adding a bulky organic base (2 equiv.)^64^ to our system did not affect the desired reactivity (Fig. 7a).

Indeed, silane/borane was not found to be an essential component for isomerization or DH when an (NHC)Pd(alkyl)X equivalent was used instead of NHC/PdX_2_ (e.g., [(**L1**)Pd(Me)Cl]_2_, Fig. 7b). When (**L1**)Pd(Bn)Cl was used in the absence of silane/borane and **R**, toluene was obtained as a byproduct instead of a conceivable BnCl via reductive elimination to Pd(0), as in other typical Pd(II)→(0)→(II) pathways. The desired product was obtained along with isomerization and disproportionation, similar to our result obtained under standard conditions in the absence of **R**. When all other conditions remained constant, trace amounts of products **2’** (<8%) and **3’** (<6%) were obtained from **1′a** and **1′o**, and 81% of the isomerization product of **1’a** was observed. Again, the low **2’** productivity could be the result of missing an appropriate regulator in the system. These results suggest that an exo-to-endo isomerization step occurs before TDHA. Additionally, similar (NHC)Pd(alkyl)X performance was observed for both sets of substrates (alkyl = Bn/Me). These findings showed that (NHC)Pd(alkyl)X was an active species for endocyclic allyl CH activation (R^1^ = COOMe or nPr), as was the case for methylene cyclohexane derivatives (see the Supplementary Information for details). Next, a comparison of the results obtained using this catalyst precursor with those obtained for our standard conditions showed that silane/borane served as a reagent only for generating (NHC)Pd(II)H from NHC/PdX_2_ and not for generating (NHC)Pd(IV)H by oxidative addition. This result again showed that the role of **R** was to increase DH competitiveness with the undesired hydropalladations and generate a (NHC)Pd(alkyl)X species. **R** did not function as an acceptor for gaseous H_2_^30^ (**R** could not undergo reduction by gaseous H_2_ in the presence of NHC-Pd(TFA)_2_ without silane/borane) or to facilitate indirect (NHC)Pd(alkyl)X reductive elimination to produce Pd(0) or generate a nonhydride catalyst^9,10^. Thus, the **2’**:**3’** ratio decreased when the system was depleted of **R** or the hydropalladated **1′** and **R** were sterically similar (e.g., **1′e** shown in Fig. 7c). Therefore, a higher selectivity and yield were obtained by slightly increasing the quantity of **R** (e.g., **1′e** in Fig. 5a).

As L_n_Pd(II)H can be obtained from a common (c-hexenyl)Pd(X)L_n_ species to realize isomerization in all the relevant systems, our result suggested that the use of NHC in the absence of a strong oxidant is key to favouring the desired isomerization steps. First, the strong σ-donating ability of NHC prevented other competing steps in many other redox-active catalytic cycles, such as metallic Pd formation and Pd(II)HX reductive elimination. Pd-H oxidation was expected to be slower in the absence of a strong oxidant, and the steric hindrance of NHC increased the efficacy of chemoselective hydropalladation when employing different substrates and **R** (Table 1 and Figs. 4, 5). Thus, hydropalladation chemistry and the quantitative analysis of the NHC effect that has been reported in the literature can serve as design guidelines. In addition, the cyclic backbone of the substrate may facilitate the desired DH. Under our standard conditions at 50 °C, acyclic olefins, such as 2-methyl-dodec-2-ene and homoallylbenzene, did not undergo DH to produce a diene as in other Pd(TFA)_2_ systems (Fig. 7di–ii). Therefore, isomerization likely occurred first in the examples using exocyclic olefins, such as allylcyclohexane (Fig. 7diii).

**Fig. 8:**
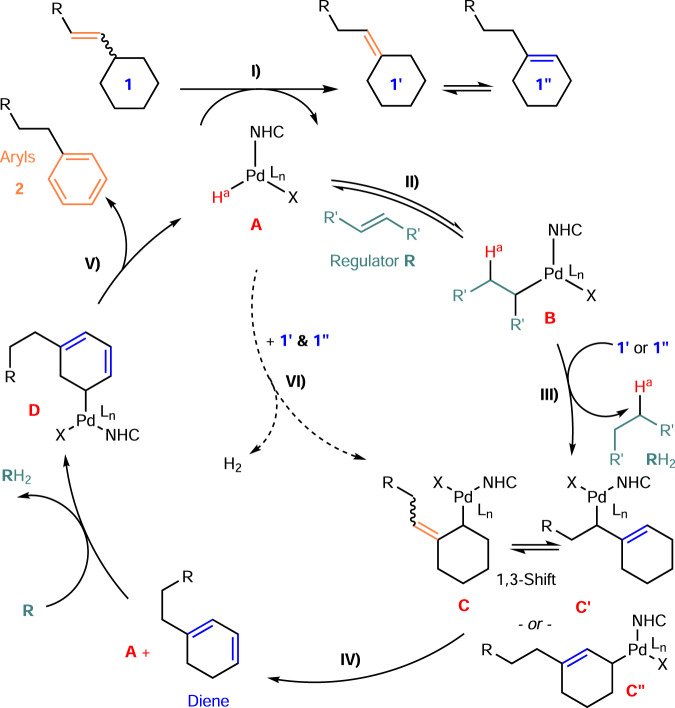
Proposed catalytic cycle at this stage. **I** Chain-walking isomerization. **II** Chemoselective and regioselective hydropalladation of regulator **R** vs. **1**, **1′** & **1″**. **III** H-transfer (major). **IV** Catalyst regeneration by isomerization and β-H elimination. **V** Catalyst regeneration by isomerization and β-H elimination. **VI** H-transfer (minor).

Figure 8 shows possible scenarios for chain-walking TDHA based on the experimental results obtained in this study and previous reports. The successful result obtained in this study was mainly attributed to the high allyl CH activation reactivity by (NHC)Pd(alkyl)X and the controlled release of (NHC)Pd(II)H (A) by the reversible hydropalladation of **R**. Then, the DHA step and **R** reduction drove the reaction forward. First, the exocyclic alkenes readily underwent exo-to-endo isomerization/chain walking (**1** → **1′** and **1″**) in the presence of a catalyst (A) generated from NHC/PdX_2_/silane or borane. As isomerization continued, chemoselective hydropalladation of regulator **R** occurred over the substrate to form species B. Next, similar to other Pd(II)/Pt(II)Me species with diimines^22,65^, this Pd(alkyl) species (B) **(**instead of Pd(TFA)_2_) served as an olefin (**1′** and **1″**) allyl CH acceptor^66^ and provided the (c-hexenyl)Pd species (C/C′/C″). Therefore, a hydropalladated **R** reduction (H-C formation) occurred instead of the more frequent H-X formation at the end by Pd(TFA)_2_. Finally, a subsequent *β*-H elimination generated the diene for another CH activation, yields final product **2**, and regenerated the catalyst (A). Thus, the quantity and choice of **R** significantly impacted isomerization, reduction, and the transfer-DH product ratio. That is, although the additives and conditions reported in the literature (heating, as well as the use of an oxidant, alkene, and acid) were used to remove Pd-H quickly for PdX_2_ catalyst regeneration^67^, the function of the **R** used in this study was to preserve Pd-H and regulate the desired isomerization and transfer-DH reactivity at the right time assisted by NHC. However, a pathway that utilizes A and **1′**/**1″** to form molecular H_2_ may still be possible at high temperature.

**Fig. 9:**
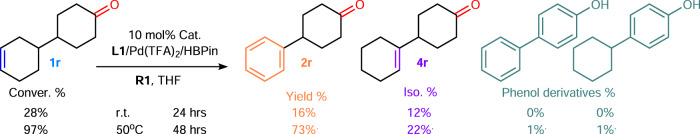
Chemoselective dehydrogenation. Cyclohexene vs. cyclohexanone.

A comparison of the proposed DH catalytic cycles of olefins by (NHC)Pd(II)H and cyclohexanone by PdX_2_^13–15,20^ showed that a highly chemoselective DH of cyclohexene over cyclohexanone is possible (Fig. 9). This reaction occurred because NHC can suppress Pd(II)HX reductive elimination and block the DH pathway based on PdX_2_ and Pd(enolate)X. As a result, **1r** underwent chemoselective DH to produce **2r** and yield <2% phenol/cyclohexenone (~73:2 or 97:3). This chemoselectivity was useful because existing DH methods based on using PdX_2_ at a higher temperature or under acidic conditions mostly favor cyclohexanones or both phenol and cyclohexenone. Thus, the **1′i** DH to produce a phenol shown in Fig. 5a was likely initiated by allyl isomerization, not by ketone DH catalyzed by soluble Pd nanoparticles^23^.

In summary, the unwanted Pd-H species produced in most previous dehydrogenative aromatization catalytic cycles were repurposed as an active catalyst by NHC to carry out both olefin chain-walking isomerization and transfer-(de)hydrogenation. Both endocyclic olefins and many easily accessible exocyclic olefins were found to act as effective DHA substrates in the presence of logical combinations of (NHC)Pd(II)HX and a regulator **R**. This change in the active DH species resulted in good reactivity and functional group tolerance under redox-neutral and mild conditions and provided a basis for the chemoselective DH of an allyl CH instead of other highly competitive CHs next to ketone and N-sites by removing the PdX_2_ species in the catalytic cycle. Using Pd-H species also obviated the oxidative and high-temperature conditions regularly employed in related redox-active catalytic cycles using common oxidants, which should be useful for late-stage aromatization. The dual-function catalyst also enabled us to make a synthetic advance that bypassed the challenges associated with incomplete exo-to-endo isomerization and tedious separation of structurally similar olefins using stepwise approaches. Overall, this study demonstrates the potential of exploiting NHC effects to regulate the reactivity of highly competitive hydrometallation and allyl CH activation. Explorations of transfer-hydrogenation using cyclohexenes as nonpolar H-donors and other transformations that involve exo-to-endo isomerization are now underway.

## Methods

### General procedure for TDHA

**1** or **1′** (0.25 mmol) and **R** (0.50 mmol) were added to an NHC/PdX_2_ catalyst mixture (0.025 mmol) with a hydride source (0.0375 mmol) in 2 mL of THF, and the resulting mixture was stirred for the indicated time period at the indicated temperature. The yield and selectivity were then determined by ^1^H NMR or GCMS (as the average of two runs). The product structures were confirmed by isolation.

## Supplementary information

Supplementary Information

## Data Availability

The data generated in this study are provided in the Supplementary Information and this published article.
