# Supplementary material for: (NHC)Pd(II) hydride-catalyzed dehydroaromatization by olefin chain-walking isomerization and transfer-dehydrogenation
Source: Nat Commun. 2022 Sep 20;13:5507. doi: 10.1038/s41467-022-33163-6 (PMC9489721; doi:10.1038/s41467-022-33163-6)
Supplement: Supplementary file 1 — Supplementary Information [file 41467_2022_33163_MOESM1_ESM.pdf]

Supplementary Information

(NHC)Pd(II) Hydride-catalyzed Dehydroaromatization by Olefin Chain-walking Isomerization and Transfer-dehydrogenation

Weihao Chen [1,2,3†], Yang Chen [1,2,3 †], Xiao Gu [1,2,3], Zaizhu Chen [1,3], Chun–Yu Ho[* 1,2,3]

[1] Guangdong Provincial Key Laboratory of Catalysis, Southern University of Science and Technology (SUSTech), Shenzhen, China.

[2] Shenzhen Grubbs Institute, Department of Chemistry, Southern University of Science and Technology (SUSTech), China.

[3] Department of Chemistry, Southern University of Science and Technology (SUSTech), Shenzhen, China.

[†] These authors contributed equally.

E-mail: jasonhcy@sustech.edu.cn

Table of Contents

Supplementary Methods...3

General Aspects...3

Synthesis and Characterization of NHC*HX (X = Cl^- or BF_4^-) 4

Synthesis and Characterization of Substrates and Alkene Regulators...9

A) Endo-cyclic olefins...9

B) Exo-cyclic olefins...13

C) Regulator **R**...18

General Procedure for Transfer-Dehydroaromatization: 19

A) General NHC Generation Procedure:...19

B) General [(NHC)PdH(X)] Catalyst Generation Procedure: 19

C) General Transfer-Dehydroaromatization Procedure:...19

D) General Work-up Procedure:...19

Representative Procedure for Transfer-Dehydroaromatization:...21

^{1}H NMR of In-situ Palladium Hydride and Palladium Alkyl Species...22

A) ^{1}H NMR of in-situ palladium hydride:...22

B) ^{1}H NMR of $[(NHC)PdMe(Cl)]_2$:...22

C) ^{1}H NMR of $[(NHC)PdBn(Cl)]_2$: 22

Control Experiment with $[(NHC)PdMe(Cl)]_2$ for dehydroaromatization:...24

Dehydroaromatization vs. Dehydrogenative Diels-Alder Reaction: 25

Chemo-Selective Transfer-Dehydroaromatization:...26

Selectivity Determination:...29

A) Separable products...31

B) Inseparable products...46

C) Volatile products...55

Characterization data of isolated products: 65

NMR spectra 69

Supplementary References...107

Supplementary Methods

General Aspects

Unless otherwise indicated, all reactions were performed under a nitrogen atmosphere from which oxygen and moisture were rigidly excluded from reagents and glassware. $PdCl_2$ and $Pd(TFA)_2$ were purchased from Sigma-Aldrich, TCI or J&K, stored in a glovebox, and used without further purification. (COD)Pd(Me)Cl was purchased from Acme, stored in a glovebox, and used without further purification. (COD)$PdCl_2$ was purchased from Bide, stored in a glovebox, and used without further purification. IPr [1,3-Bis(2,6-diisopropylphenyl)imidazol-2-ylidene] was purchased from TCI, stored under a nitrogen atmosphere, and used without further purification. Anhydrous THF (no BHT) was purchased from J&K and dried again with CaH_2 before use. Potassium bis(trimethylsilyl)amide (KHMDS) were purchased from Aldrich, stored in a glovebox, and used without further purification.

Both commercially available and synthesized endo-/exo-cyclic olefins were dried with CaH_2 or Na_2SO_4 before use. All the regulators were dried with CaH_2 or molecule sieves before use.

Analytical thin layer chromatography (TLC) was performed using EM Science silica gel 60 F254 plates. The developed chromatogram was analyzed by UV lamp (254 nm), ethanolic phosphomolybdic acid (PMA), or potassium permanganate ($KMnO_4$). Purification of the product was performed by using Silica Gel (230–400 mesh, 0.040-0.063 mm) coarse fritted glass column.

^{1}H and ^{13}C NMR spectra were recorded on Bruker spectrometers in $CDCl_3$, C_6D_6, *d*8-THF or *d*6-Acetone (400 or 500 MHz for ^{1}H and 100 or 125 MHz for ^{13}C). Chemical shifts in ^{1}H NMR spectra are reported in ppm on the δ scale from an internal standard of TMS. Data are reported as follows: chemical shift, multiplicity (s = singlet, d = doublet, t = triplet, q = quartet, m = multiplet, br = broad), coupling constant in hertz (Hz), and integration. Chemical shifts of ^{13}C NMR spectra are reported in ppm from the central peak of $CDCl_3$ (77.16 ppm), C_6D_6 (128.06 ppm), *d*8-Acetone (29.84 ppm), on the δ scale. Yield and selectivity were determined by integration of areas of selected peaks in crude ^{1}H NMR with relaxation time d1 = 10 seconds and nitromethane (CH_3NO_2) or mesitylene as standard. High resolution mass spectra (HRMS) were obtained on a Finnigan MAT 95XL GC Mass Spectrometer of Southern University of Science and Technology, China.

Synthesis and Characterization of NHC*HX (X = Cl⁻ or BF₄⁻)

The NHCs or NHC*HX involved in this work are commercially available or synthesized according to the previous reports.

NHC*HX for **L1** and **L7** are commercially available.

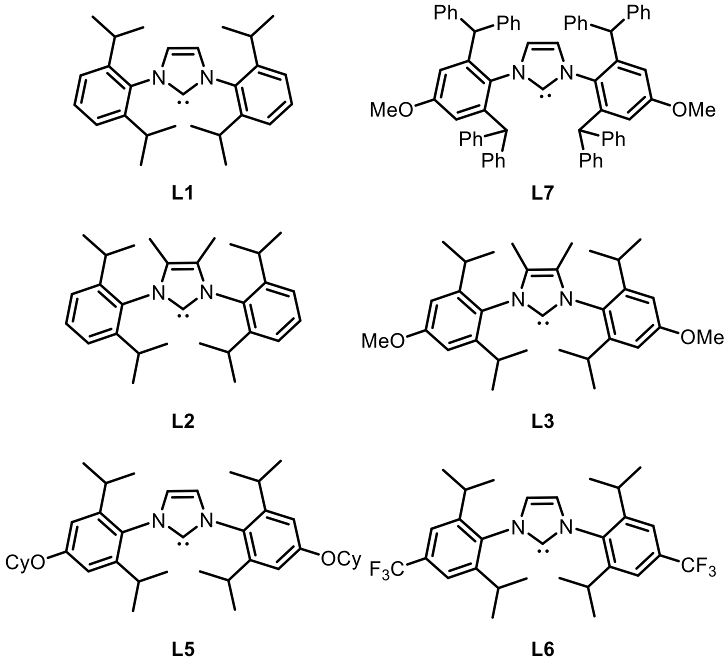

Supplementary Figure 1. Structures of NHCs

NHC*HX for **L2**, **L3**, **L4**, **L5**, **L6** were synthesized according to reported methods[1].

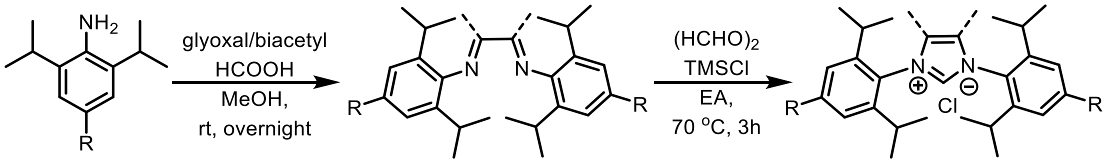

In a round bottom flask, aniline (40 mmol) and glyoxal 40% w/w in water or biacetyl (2.3 mL, 20 mmol) were introduced in MeOH (40 mL). The reaction mixture was stirred at room temperature overnight. The precipitate was collected on a frit, washed with MeOH and dried under vacuum. The product was obtained as a yellow powder and used without further purification. In case the diamine was liquid, the reaction mixture was concentrated, and the crude product was subjected to column chromatograph for purification.

In an oven-dried flask, the above diimine was introduced in anhydrous EtOAc (40 mL) and cooled down to 0 °C. In a separate flask, paraformaldehyde (24 mmol) was stirred in a 4 M dioxane solution of HCl (32 mmol) for 10 minutes and added to the diimine solution at 0 °C. The reaction mixture was stirred 70 °C for 3 hrs. The precipitate was collected on a frit and washed with EtOAc.

Then, the residue was dissolved in MeOH and anhydrous $NaHCO_3$ (2.5 equiv.) was added. The resulting mixture was stirred at room temperature for 1 hour. Diethyl ether was added affording a precipitate which was collected on a frit and dried under vacuum leading to the pure imidazolium chloride salt.

When the resultant imidazolium salt was sticky, an anion exchange was conducted. To an oven-dried flask, imidazolium chloride salt (1 equiv.) was dissolved in DCM (0.2 M). Sodium tetrafluoroborate (1.5 equiv.) was added and the mixture was stirred at room temperature for 3 hrs. After removing precipitate by filtration, the filtrate was concentrated to afford imidazolium tetrafluoroborate salt.

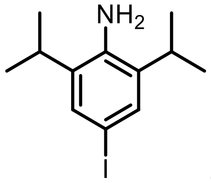

4-iodo-2,6-di-*iso*-propylaniline[1]

In a round bottom flask 2,6-di-*iso*-propylaniline (3.8 mL, 20 mmol) and sodium bicarbonate (5.1 g, 60 mmol) were introduced in MeOH (50 mL). A ICl solution 1M in DCM (22 mL) was added dropwise and the reaction mixture was stirred at room temperature for 3 days. Then, the solids were filtered off the mixture and rinsed with Et_2O. The filtrate was concentrated under vacuum leading to a dark orange oil. A saturated solution of sodium thiosulfate was added to this oil and the reaction mixture was stirred for 10 minutes. After an extraction with Et_2O, the combined organic layers were dried over MgSO4 and concentrated under vacuum. The pure product was obtained as brown oil in 75% yield.

^{1}H NMR (500 MHz, $CDCl_3$) δ 7.28 (s, 2H), 2.85 (sept, J = 6.8Hz, 2H), 1.24 (d, J = 6.8 Hz). The characterization data is in agreement with the literature.

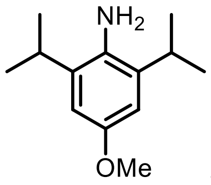

4-methoxy-2,6-di-*iso*-propylaniline[1]

In a Schlenk tube, 4-iodo-2,6-di-iso-propylaniline (294 mg, 0.97 mmol) was introduced in anhydrous toluene (60 mL). CuI (9.2 mg, 0.05 mmol), tetramethylphenanthroline (23 mg, 0.10 mmol) and CsOH (948 mg, 2.91 mmol) were added. The reaction mixture was stirred 1 hour at 80 °C and anhydrous MeOH (3.2 mL) was added. The reaction mixture was stirred for 24 hrs at

80 °C. After returning to room temperature, the reaction mixture was filtered through a pad of silica, the cake was washed with EtOAc and the filtrate was concentrated under vacuum. The crude product was subjected to chromatograph to afford pure product in 90% yield.

^{1}H NMR (500 MHz, $CDCl_3$) δ 6.55 (s, 2H), 3.78 (s, 3H), 2.98 (sept, *J* = 6.8Hz, 2H), 1.27 (d, *J* = 6.8Hz, 12H). The characterization data is in agreement with the literature.

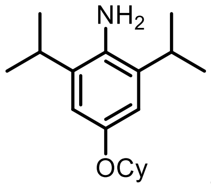

4- cyclohexyloxy-2,6-di-*iso*-propylaniline[2]

To an oven-dried screw-cap test tube, CuI (9.5 mg, 0.050 mmol), Me_4Phen (24 mg, 0.10 mmol), 4-iodo-2,6-di-*iso*-propylaniline (1.0 mmol), Cs_2CO_3 (490 mg, 1.5 mmol), and a magnetic stir bar were charged. The reaction vessel was fitted with a rubber septum. The test tube was evacuated and re-filled with dry argon, and toluene (0.50 mL) were then added by syringe. The rubber septum was removed and the reaction tube was quickly sealed with a Teflon-lined septum. The vessel was immersed in a pre-heated oil bath and stirred vigorously at 110 °C for 48 hrs. The reaction mixture was cooled to room temperature, diluted with ethyl acetate (15 mL), and filtered through a plug of silica, eluting with additional ethyl acetate. The filtrate was concentrated under vacuum, and the crude product was subject to chromatograph to afford pure product in 32% yield.

^{1}H NMR (400 MHz, $CDCl_3$) δ 6.64 (s, 2H), 4.07 (m, 1H), 3.80-2.95 (br, 2H), 2.95 (hept, *J* = 6.8 Hz, 2H), 2.04 - 1.95 (m, 2H), 1.84 - 1.76 (m, 2H), 1.60 - 1.28 (m, 6H), 1.25 (d, *J* = 6.8 Hz, 12H).

^{13}C NMR (100 MHz, $CDCl_3$) δ 150.9, 134.4, 134.0, 111.9, 76.6, 32.3, 28.3, 25.9, 24.1, 22.7. The characterization data is in agreement with the literature.

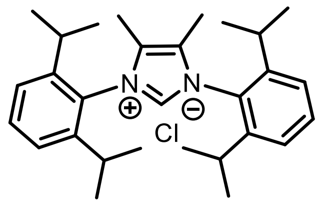

L2*HCl, synthesized from 2,6-di-*iso*-propylaniline and biacetyl in overall 40% yield[1].

^{1}H NMR (500 MHz, *d*4-MeOD) δ 9.78 (s, 1H), 7.67 (t, *J* = 7.8Hz, 2H), 7.52 (d, *J* = 7.8Hz, 4H), 2.39 (sept, *J* = 6.8Hz, 4H), 2.15 (s, 6H), 1.32 (d, *J* = 6.8Hz, 12H), 1.20 (d, *J* = 6.8Hz, 12H). The characterization data is in agreement with the literature.

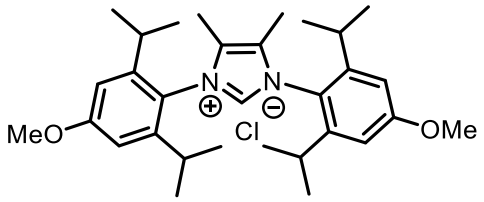

L3*HCl, synthesized from 4-methoxy-2,6-di-*iso*-propylaniline and biacetyl in overall 32% yield.[1]
^{1}H NMR (500 MHz, *d*4-MeOD) δ 9.72 (s, 1H), 7.02 (s, 4H), 3.92 (s, 6H), 2.37 (sept, *J* = 6.7Hz, 4H), 2.17 (s, 6H), 1.33 (d, *J* = 6.7Hz, 12H), 1.23 (d, *J* = 6.7Hz, 12H). The characterization data is in agreement with the literature.

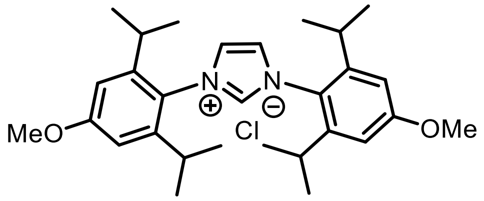

L4*HCl, synthesized from 4-methoxy-2,6-di-*iso*-propylaniline and glyoxal in overall 45% yield.[1]
^{1}H NMR (500 MHz, *d*4-MeOD) δ 9.89 (s, 1H), 7.00 (s, 4H), 3.92 (s, 6H), 2.45 (sept, *J* = 6.8Hz, 4H), 1.33 (d, *J* = 6.8Hz, 12H), 1.26 (d, *J* = 6.8Hz, 12H). The characterization data is in agreement with the literature.

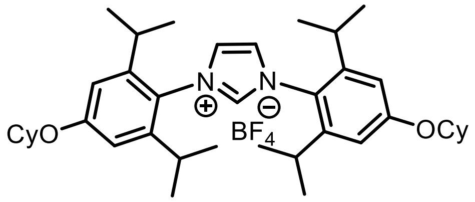

L5*HBF_4, synthesized from 4- cyclohexyloxy-2,6-di-*iso*-propylaniline and glyoxal in overall 33% yield.

^{1}H NMR (500 MHz, $CDCl_3$) δ 8.52 (s, 1H), 7.74 (s, 2H), 6.77 (s, 4H), 4.34 (m, 2H), 2.34 (sept, *J* = 6.9 Hz, 4H), 2.00 (m, 4H), 1.84 (m, 4H), 1.59 (m, 6H), 1.48 - 1.31 (m, 6H), 1.24 (d, *J* = 6.9 Hz, 12H), 1.16 (d, *J* = 6.9 Hz, 12H). ^{13}C NMR (125 MHz, $CDCl_3$) δ 160.4, 146.6, 137.6, 127.1, 122.2, 111.7, 75.6, 31.7, 29.2, 25.6, 24.5, 23.8, 23.7.

HRMS-ESI (m/z): $[M-BF_4]^+$: calculated for $C_{39}H_{57}N_2O_2$ 585.4415; found 585.4416.

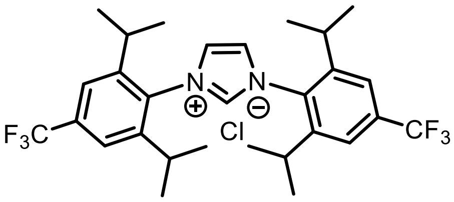

L6*HCl, synthesized from 4- trifluoromethyl-2,6-di-*iso*-propylaniline and glyoxal in overall 8% yield.

^{1}H NMR (500 MHz, Acetone-*d*6) δ 13.14 (s, 1H), 8.52 (d, *J* = 1.5 Hz, 2H), 7.77 (s, 4H), 2.65 (p, *J* = 6.9 Hz, 4H), 1.35 - 1.31 (m, 24H). ^{13}C NMR (125 MHz, Acetone-*d*6) δ 205.4, 205.3, 205.1, 147.2, 142.9, 142.6, 133.9, 133.3, 133.1, 132.8, 132.6, 127.2, 125.7, 125.0, 122.9, 121.5, 121.4, 121.4, 121.4, 23.7, 22.4. ^{19}F NMR (375 MHz, Acetone-*d*6) δ -63.3.

HRMS-ESI (m/z): $[M-BF_4]^+$: calculated for $C_{39}H_{57}N_2O_2$ 585.4415; found 585.4416.

Synthesis and Characterization of Substrates and Alkene Regulators

Most of the substrates and alkene regulators are commercially available, and others were prepared according to the literatures.

A) Endo-cyclic olefins

1a, **1d**, **1g**, **1h**, **1i**, **1j**, **1k**, **1l,** and **1m** are commercially available.

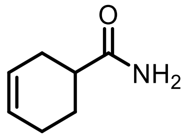

1b was synthesized according to reported method[3].

To an oven-dried flask, 3-cyclohexene-l-carboxylic acid (0.62 g, 5 mmol) and thionyl chloride (2 mL) were heated at reflux under anhydrous conditions for 1 h, and the excess thionyl chloride was removed under reduced pressure. The residue was added slowly to a stirred ice-cold concentrated ammonium hydroxide solution. The solid that precipitated was filtered, washed with cold water, and dried in vacuo. Further crystallization ($CHCl_3$/Hexane) afforded 3-cyclohexane-l-carboxamide **1b** in 40% yield.

^{1}H NMR (500 MHz, $CDCl_3$) δ 6.04 (1H, br), 5.68-5.64 (3H, m), 2.45-2.38 (1H, m), 2.24-2.07 (4H, m), 1.98-1.95 (1H, m), 1.72-1.64 (1H, m). ^{13}C NMR (125 MHz, $CDCl_3$) δ 178.6, 126.8, 125.2, 40.6, 28.0, 25.6, 24.5. The characterization data is in agreement with the literature.

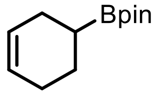

1c was synthesized according to reported method[4].

To a flame dried 25-mL Schlenk flask equipped with a magnetic stir bar, Co(dppp)Cl_2 (68.0 mg, 0.125 mmol) and methylaluminoxane (290.0 mg, 5.0 mmol) were added inside a glove box. The ground glass joint of the flask was closed with a rubber septum, and taped using electrical tape around the septum. The flask was then taken out of the box, and anhydrous ether (25 ml) was added through the septum, stirred for a few minutes. 1,3-cyclohexadiene (200 mg, 2.5 mmol) was added neat via a microliter syringe immediately followed by addition of pinacolborane (HBpin, 336 mg, 2.62 mmol, 380 μL). After overnight, the reaction mixture was filtered over a pad of Celite® with 50% hexane-ether to rinse the column. The crude product was purified by flash chromatography to afforded **1c** in 60% yield.

^{1}H NMR (500 MHz, $CDCl_3$) δ 5.66-5.74 (m, 2H), 1.98-2.12 (m, 4H), 1.78-1.85 (m, 1H), 1.50-1.61 (m, 2H), 1.25 (s, 12H). ^{13}C NMR (125 MHz, $CDCl_3$) δ 127.7, 127.1, 83.0, 26.4, 25.4, 24.9, 24.8,

24.0. The characterization data is in agreement with the literature.

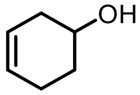

1e was synthesized according to reported method[5].

A mixture of oxalic acid (14 mg, 0.16 mmol) and 1-methoxycyclohexa-1,4-diene (0.6 mL, 4.3 mmol) in MeOH-H2O (3:1, 3.3 mL) was stirred at room temperature for 1 h. After dilution with DCM, the mixture was separated, and the aqueous layer was extracted with DCM. The combined organic layers were dried over $MgSO_4$ and concentrated under reduced pressure to afford cyclohex-3-en-1-one. The resultant ketone was dissolved in MeOH (5.0 mL) and $NaBH_4$ (377 mg, 9.96 mmol) was added at 0 °C. The mixture was warmed up to room temperature and was stirred for 20 min. After quenching with acetone, the resulting mixture was concentrated under reduced pressure, and the residue was diluted with a saturated aqueous NH_4Cl solution. The mixture was separated, and the aqueous layer was extracted with EA. The combined organic layers were dried over $MgSO_4$ and concentrated under reduced pressure. The crude product was purified by flash chromatography to afforded **1e** in 38% yield over two steps.

^{1}H NMR (500 MHz, $CDCl_3$) δ 5.72-5.54 (m, 2H), 4.00-3.91 (m, 1H), 2.42-2.33 (m, 1H), 2.25-2.05 (m, 2H), 2.05-1.96 (m, 1H), 1.91-1.84 (m, 1H), 1.8-1.59 (m, 1H). ^{13}C NMR (125 MHz, $CDCl_3$) δ 126.9, 124.1, 67.1, 34.5, 31.0, 23.7. The characterization data is in agreement with the literature.

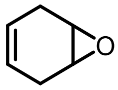

1f was synthesized according to reported method[6].

To a solution of 1,4-cyclohexadiene (1.6 g, 20 mmol) in DCM (50 mL) at 0 °C was added m-CPBA (4.8 g, 21 mmol). The mixture was allowed to stir for 72h while warming to r.t.. Na_2SO_4 was added while stirring followed by $NaHCO_3$. After 5 minutes the mixture was filtered and carefully concentrated under slight vacuum, which afforded **1f** as a DCM solution in around 70% yield (due to the low boiling point).

^{1}H NMR (500 MHz, $CDCl_3$) δ 5.44 (d, J = 2.3 Hz, 2 H), 3.25 (s, 2 H), 2.64- 2.37 (m, 4 H). The characterization data is in agreement with the literature.

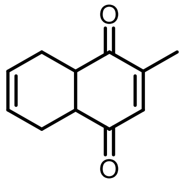

1n was synthesized according to reported method[7].

To an oven-dried flask, 1, 3-butadiene (20 mmol, 2M in toluene) and methylquinone (20 mmol, 2.44 g) was mixed in toluene (40 ml). The mixture was stirred at r.t. for 3 days. Then the reaction mixture was concentrated, and the crude product was purified by flash chromatography to afforded **1o** in 50% yield.

^{1}H NMR (500 MHz, $CDCl_3$) δ 6.53 (q, J = 1.5 Hz, 1 H), 5.73-5.66 (m, 2 H), 3.25-3.16 (m, 2H), 2.44-2.37 (m, 2 H), 2.12 (m, 2 H), 2.00 (d, J = 1.5 Hz, 3 H). ^{13}C NMR (125 MHz, $CDCl_3$) δ 200.3, 199.8, 148.9, 136.0, 124.4, 124.4, 46.5, 46.2, 24.3, 16.2. The characterization data is in agreement with the literature.

1o, **1p**, **1q** were synthesized according to reported method[8].

Inside a glovebox, [Ni(μ-Cl)(**IPr**)]$_2$ (0.1 mmol) and olefins (2 mmol) was added to a oven-dried testtube. The testtube was closed with a rubber septum and taped using electrical tape around the septum. The flask was then taken out of the box, and anhydrous DCM (5 ml) was added through the septum. After reacted for 3 hrs at r.t., the obtained solution was concentrated carefully, and the crude product was purified by flash chromatography to afforded pure product.

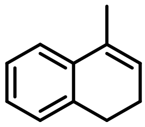

1o, 90% yield from 1-methylene-1,2,3,4-tetrahydronaphthalene[8].

^{1}H NMR (500 MHz, $CDCl_3$) δ 7.25-7.20 (m, 2H), 7.17-7.13 (m, 2H), 5.88-5.86 (m, 1H), 2.77 (dd, J = 8.4, 8.4 Hz, 2H), 2.28-2.24 (m, 2H), 2.66 (d, J = 1.7 Hz, 3H). ^{13}C NMR (125 MHz, $CDCl_3$) δ 136.3, 135.8, 132.1, 127.3, 126.6, 126.3, 125.4, 122.7, 28.3, 23.2, 19.2. The characterization data is in agreement with the literature.

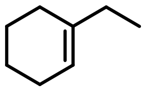

1p, 60% yield from vinyl cyclohexane[8].

^{1}H NMR (500 MHz, $CDCl_3$) δ 5.42-5.38 (m, 1H), 2.01-1.90 (m, 5H), 1.66-1.54 (m, 5H), 0.99 (t, J = 7.4 Hz, 3H). The characterization data is in agreement with the literature.

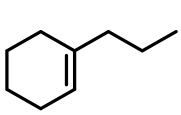

1q, 85% yield from allyl cyclohexane[8].

^{1}H NMR (500 MHz, $CDCl_3$) δ 5.42-5.34 (m, 1H), 2.01-1.95 (m, 2H), 1.93-1.86 (m, 4H), 1.66-1.50 (m, 4H), 1.40 (dq, J = 14.7, 7.4 Hz, 2H), 0.87 (t, J = 7.3 Hz, 3H). ^{13}C NMR (125 MHz, $CDCl_3$) δ 138.0, 120.8, 40.4, 28.4, 25.4, 23.2, 22.8, 21.0, 14.0. The characterization data is in agreement with

the literature.

Endocyclic olefin **1r** was synthesized based on reported method[9].

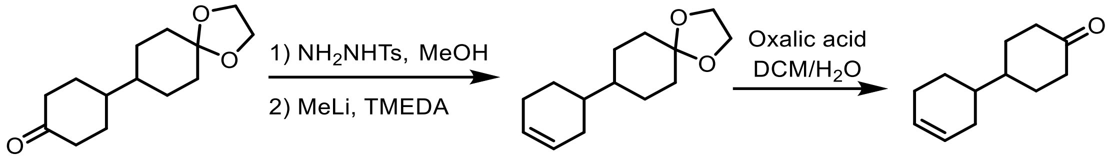

To a solution of p-toluenesulfonylhydrazide (12.0 mmol, 1.2 equiv.) in 50 mL of methanol was added bicyclohexane-4,4-dione monoethylene ketal (10.0 mmol, 1.0 equiv.). After stirring overnight, the resultant white crystals were filtered, washed with 1:1 pentane:ether, and dried in a vacuum oven. The crude product was used directly without further purification. Under nitrogen atmosphere, 1.6 M MeLi in ether (12.0 mmol, 1.2 equiv.) was added via syringe to a solution of above tosylhydrazone in 40 mL of TMEDA in an ice bath. After warm-up and stir overnight, it was cooled with ice water, quenched with water, and extracted successively with pentane. The organic layer was washed with water and brine, dried over MgSO4, concentrated. Pure product was collected after column chromatography, with 75% yield over two steps.

The resultant product was dissolved in 10 ml DCM. Then, 10 ml water and oxalic acid (15.0 mmol, 2.0 equiv.) were added. The mixture was stirred vigorously at r.t. overnight. After extraction with DCM, pure product was obtained in 95% yield.

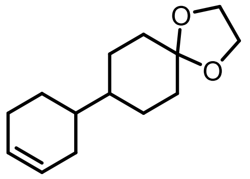

^{1}H NMR (500 MHz, $CDCl_3$) δ 5.65 - 5.59 (m, 2H), 3.90 (s, 4H), 2.07 - 1.93 (m, 3H), 1.80 - 1.63 (m, 6H), 1.47 (td, J = 12.7, 3.9 Hz, 2H), 1.42 - 1.33 (m, 1H), 1.34 - 1.09 (m, 4H). ^{13}C NMR (125 MHz, $CDCl_3$) δ 127.0, 126.9, 109.2, 64.3, 64.2, 41.4, 38.4, 34.9, 34.9, 29.2, 27.2, 27.0, 26.4, 26.0. HRMS-ESI (m/z): $[M+H]^+$: calculated for $C_{14}H_{22}O_2^+$: 223.1693; found 223.1691.

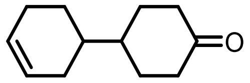

^{1}H NMR (500 MHz, $CDCl_3$) δ 5.67 - 5.60 (m, 2H), 2.39 - 2.24 (m, 4H), 2.10 - 1.97 (m, 5H), 1.82 - 1.72 (m, 2H), 1.64 - 1.38 (m, 4H), 1.28 - 1.19 (m, 1H). ^{13}C NMR (125 MHz, $CDCl_3$) δ 127.0, 126.9, 109.2, 64.3, 64.2, 41.4, 38.4, 34.9, 34.9, 29.2, 27.2, 27.0, 26.4, 26.0.

HRMS-ESI (m/z): $[M+H]^+$: calculated for $C_{12}H_{19}O^+$: 179.1430; found 179.1430.

B) Exo-cyclic olefins

i) **1′c**, **1′d**, **1′e**, **1′g**, **1′i**, **1′o** are commercially available.

ii) **1′a**, **1′b**, **1′f**, **1′h**, **1′j**, **1′k**, **1′l**, and **1′w** were synthesized by Wittig reaction.

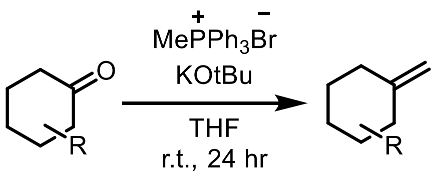

A round bottom flask equipped with a magnetic stir bar and a rubber septum was flame-dried and cooled to room temperature under vacuum then refilled with nitrogen. The flask was briefly opened, charged with methyltriphenylphosphonium bromide (4.5 mmol), and resealed. Anhydrous THF (60 mL) was then added via syringe and the stirred solution was cooled to 0 °C under positive nitrogen pressure. The flask was charged with KOtBu (4.5 mmol) in one portion by quickly removing and replacing the septum. The resulting solution was allowed to stir at 0 °C for 1 hour under positive nitrogen pressure, at which time ketone (3 mmol) was added. The resulting yellow suspension was stirred at r.t. for 24 hours. After that, the reaction solution was quenched carefully with ice water, and then extracted with diethyl ether (3 x 20 mL). The combined organic layers were dried with $MgSO_4$ and concentrated in vacuo. The crude material was purified via silica column chromatography to afford the desired olefins.

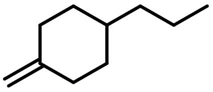

1′a, 92% yield from 4-propylcyclohexan-1-one[10].

^{1}H NMR (500 MHz, $CDCl_3$) δ 4.58 (s, 2H), 2.33-2.24 (m, 2H), 2.06-1.94 (m, 2H), 1.84-1.76 (m, 2H), 1.40-1.24 (m, 3H), 1.22-1.13 (m, 2H), 1.03-0.92 (m, 2H), 0.89 (t, *J* = 7.2, 3H). ^{13}C NMR (125 MHz, $CDCl_3$) δ 150.3, 106.4, 39.0, 36.9, 34.7, 34.5, 20.1, 14.4. The characterization data is in agreement with the literature.

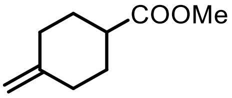

1′b, 90% yield from methyl 4-oxocyclohexane-1-carboxylate[11].

^{1}H NMR (400 MHz, $CDCl_3$) δ 4.65 (s, 2H), 3.67 (s, 3H), 2.46 (tt, *J* = 11.0, 3.6 Hz, 1H), 2.34 (dt, *J* = 13.0, 3.9 Hz, 2H), 2.12 - 1.95 (m, 4H), 1.64 - 1.57 (m, 2H). ^{13}C NMR (100 MHz, $CDCl_3$) δ 176.0, 147.7, 108.1, 51.7, 42.7, 33.8, 30.3.

HRMS-ESI (m/z): $[M+H]^+$ calcd for $C_9H_{15}O_2^+$: 155.1067; found 155.1065

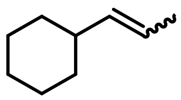

1′f, 95% yield (E/Z = 10:90) from cyclohexanecarbaldehyde and ethyltriphenylphosphonium bromide[12].

^{1}H NMR (500 MHz, $CDCl_3$) δ 5.38-5.20 (m, 2H), 2.33-2.23 (m, 1H), 1.75-1.59 (m, 8H), 1.35-1.25 (m, 3H), 1.22-1.12 (m, 1H), 1.10-1.00 (m, 1H). The characterization data is in agreement with the literature.

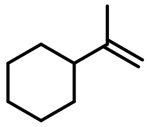

1′h, 90% yield from 1-cyclohexylethan-1-one[13].

^{1}H NMR (500 MHz, $CDCl_3$) δ 4.60-4.56 (m, 2H), 1.85-1.64 (m, 6H), 1.63 (s, 3H), 1.30-1.00 (m, 5H). ^{13}C NMR (125 MHz, $CDCl_3$) δ 151.2, 108.0, 45.8, 32.2, 26.9, 26.6, 21.0. The characterization data is in agreement with the literature.

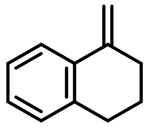

1′j, 95% yield from 1-Tetralone[14].

^{1}H NMR (500 MHz, $CDCl_3$) δ 7.67-7.63 (m, 1H), 7.22-7.13 (m, 2H), 7.13-7.08 (m, 1H), 5.48 (d, J = 1.4 Hz, 1H), 4.96 (d, J = 1.4 Hz, 1H), 2.86 (t, *J* = 6.3 Hz, 2H), 2.55-2.52 (m, 2H), 1.90-1.84 (m, 2H). ^{13}C NMR (125 MHz, $CDCl_3$) δ 136.3, 135.8, 132.2, 127.3, 126.6, 126.3, 125.4, 122.7, 28.3, 23.2, 19.3. The characterization data is in agreement with the literature.

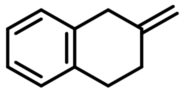

1′k, 95% yield from beta-Tetralone[15].

^{1}H NMR (500 MHz, $CDCl_3$) δ 7.10-7.07 (m, 4H), 4.86 (d, *J* = 1.2 Hz, 1 H), 4.83 (d, *J* = 1.2 Hz, 1 H), 3.52 (s, 2 H), 2.84 (t, *J* = 6.4 Hz, 2 H), 2.46 (t, *J* = 6.4 Hz, 2 H); ^{13}C NMR (125 MHz, $CDCl_3$) δ 145.4, 137.0, 128.5, 128.3, 125.9, 125.8, 125.5, 108.2, 37.1, 31.8, 31.2. The characterization data is in agreement with the literature.

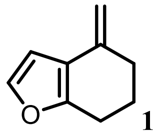

1′l, 70% yield from 6,7-dihydro-4(5H)-benzofuranone[16].

^{1}H NMR (500 MHz, $CDCl_3$) δ7.24 (d, *J* = 1.9 Hz, 1H), 6.47 (d, *J* = 1.9 Hz, 1H), 4.99 (d, *J* = 1.4 Hz, 1H), 4.77 (d, *J* = 1.4 Hz, 1H), 2.68 (t, *J* = 6.3 Hz, 2H), 2.47-2.37 (2H, m), 2.01-1.80 (2H, m), ^{13}C NMR (125 MHz, $CDCl_3$) δ153.3, 141.2, 138.5, 119.2, 106.4, 104.8, 31.3, 31.3, 23.4. The

characterization data is in agreement with the literature.

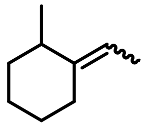

1′w, 65% yield from 2-methlycyclohexan-1-one and methyltriphenylphosphonium bromide by Wittig reaction[17].

^{1}H NMR (500 MHz, $CDCl_3$) δ 5.17-5.08 (m, 1H), 2.95-2.87 (m, 0.45H), 2.56-2.50 (m, 0.55H), 2.27-2.19 (m, 0.55H), 2.13-2.05 (m, 0.55H), 1.98-1.94 (m, 0.45H), 1.82-1.79 (m, 0.45H), 1.79-1.09 (m, 6H), 1.06 (d, *J* = 7.2Hz, 1.35H), 1.03 (d, *J* = 6.7Hz, 1.65H), 0.93-0.89 (m, 3H). The characterization data is in agreement with the literature.

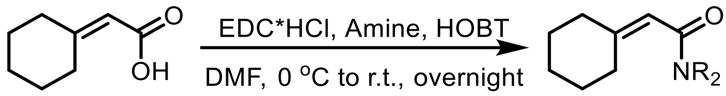

1′m, **1′n** were synthesized based on reported methods[18]. 3-Ethyl-1-[3-(dimethylamino)propyl] carbodiimide hydrochloride (EDC*HCl) (6.0 mmol, 1.2 equiv.) was added to mixture of 2-cyclohexylideneacetic acid (5.0 mmol, 1.0 equiv.), amine (6.0 mmol, 1.2 equiv.), 1-hydroxybenzotriazole hydrate (6.0 mmol, 1.2 equiv.) and DMF (40 ml) with ice-cooling and the mixture was stirred for 15 h at room temperature. Water and a mixture of $CHCl_3$ and MeOH (9 : 1) were added to the reaction mixture, and the aqueous layer was separated. The aqueous layer was extracted with a mixture of $CHCl_3$ and MeOH (9 : 1) four times. The combined organic layer was dried with Na_2SO_4, concentrated and chromatographed to afford pure product.

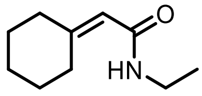

1′m, 56% yield from ethylamine.

^{1}H NMR (500 MHz, $CDCl_3$) δ 5.47 (s, 1H), 5.35 (br, 1H), 3.32 (qd, *J* = 7.3, 5.5 Hz, 2H), 2.80 (t, *J* = 5.7 Hz, 2H), 2.13 (t, *J* = 5.9 Hz, 2H), 1.68 - 1.55 (m, 6H), 1.15 (t, *J* = 7.3 Hz, 3H). ^{13}C NMR (125 MHz, $CDCl_3$) δ 167.2, 157.5, 115.9, 37.9, 34.2, 29.8, 28.7, 27.9, 26.5, 15.1.

HRMS-ESI (m/z): $[M+H]^+$: calculated for $C_{10}H_{18}NO$ 168.1388; found 168.1384.

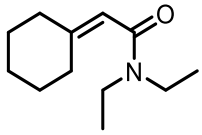

1′n, 80% yield from diethylamine.

^{1}H NMR (400 MHz, $CDCl_3$) δ 5.72 (s, 1H), 3.45 - 3.32 (m, 4H), 2.43 (t, *J* = 5.7 Hz, 2H), 2.17 (t, *J* = 5.5 Hz, 2H), 1.68 - 1.52 (m, 6H), 1.15 (t, *J* = 7.1 Hz, 6H). ^{13}C NMR (100 MHz, $CDCl_3$) δ 167.8,

152.0, 115.5, 42.6, 39.4, 37.0, 30.5, 28.4, 27.6, 26.3, 14.3, 13.2.

HRMS-ESI (m/z): [M+H]$^+$: calculated for $C_{12}H_{22}NO$ 196.1701; found 196.1698.

iii) **1′p**, **1′q**, **1′r**, **1′s**, **1′t**, **1′u**, **1′v**, and **1′x** were synthesized by HWE reactions.

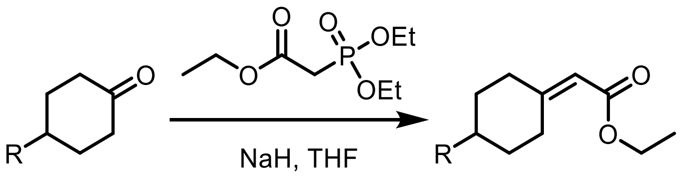

Triethyl phosphonoacetate (10.4 mmol, 1.3 equiv.) was added dropwise to a suspension of 60% NaH (10.4 mmol, 1.3 equiv.) in dry THF at 0ºC and the mixture was stirred for 30 minutes. Then, the corresponding ketone (8.0 mmol, 1 equiv.) was added at 0 ºC and the mixture was stirred overnight at room temperature. After quenched with water, the organic layer was separated, and the aqueous layer was extracted with diethyl ether twice. The combined organic solutions were dried over $MgSO_4$, concentrated and purified by column chromatography to afford the desired product.

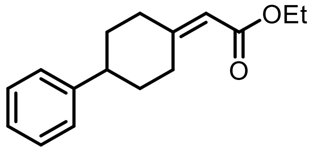

1′p, 65% yield from 4-phenylcyclohexan-1-one[19].

^{1}H NMR (500 MHz, $CDCl_3$) δ7.33-7.10 (m, 5H), 5.67 (s, 1H), 4.15 (q, J = 7.1 Hz, 2H), 4.03-3.92 (m, 1H), 2.77 (tt, J = 12.2, 3.4 Hz, 1H), 2.43-2.27 (m, 2H), 2.10-1.96 (m, 3H), 1.72-1.54 (m, 2H), 1.28 (t, J = 7.1 Hz, 3H). ^{13}C NMR (125 MHz, $CDCl_3$) δ166.1, 161.2, 145.5, 128.1, 126.4, 125.8, 113.5, 59.6, 44.2, 37.8, 35.7, 34.9, 29.6, 14.6. The characterization data is in agreement with the literature.

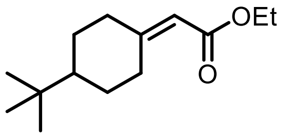

1′q, 60% yield from 4-*tert*-butylcyclohexan-1-one[20].

^{1}H NMR (500 MHz, $CDCl_3$) δ5.58 (s, 1H), 4.12 (q, J = 7.1 Hz, 2H), 3.91-3.80 (m, 1H), 2.27-2.32 (m, 1H), 2.19-2.11 (m, 1H), 1.98-1.72 (m, 3H), 1.26 (t, J = 7.1 Hz, 3H), 1.28-1.04 (m, 3H), 0.84 (s, 9H). ^{13}C NMR (125 MHz, $CDCl_3$) δ166.9, 163.5, 112.7, 59.4, 47.8, 37.9, 32.4, 29.5, 29.2, 28.4, 27.5, 14.3. The characterization data is in agreement with the literature.

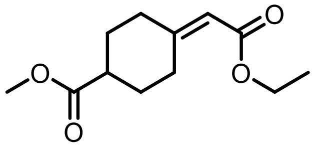

1′r, 75% yield from methyl 4-oxocyclohexane-1-carboxylate[20].

^{1}H NMR (400 MHz, $CDCl_3$) δ 5.58 (s, 1H), 4.18 - 4.01 (m, 2H), 3.64 (s, 3H), 3.63 - 3.53 (m, 1H), 2.60 - 2.48 (m, 1H), 2.36 - 2.25 (m, 1H), 2.25 - 2.07 (m, 2H), 2.07 - 1.98 (m, 2H), 1.74 - 1.53 (m, 2H), 1.28 - 1.16 (m, 3H). ^{13}C NMR (100 MHz, $CDCl_3$) δ 175.3, 166.6, 160.5, 114.3, 59.7, 51.8, 42.2, 36.0, 30.1, 29.5, 27.9, 14.4.

HRMS-ESI (m/z): $[M+H]^+$: calculated for $C_{12}H_{19}O_4$ 227.1283; found 227.1278.

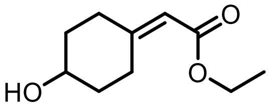

1′s, 65% yield from 4-hydroxycyclohexan-1-one[21].

^{1}H NMR (500 MHz, $CDCl_3$) δ 5.64 (s, 1H), 4.14 (q, J = 7.1 Hz, 2H), 3.93 (br, 1H), 3.41-3.27 (m, 1H), 2.53-2.36 (m, 2H), 2.24-2.12 (m, 1H), 1.98-1.92 (m, 2H), 1.64-1.49 (m, 3H), 1.27 (t, J = 7.1 Hz, 3H);^{13}C NMR (125 MHz, $CDCl_3$) δ166.8, 160.9, 114.2, 68.5, 59.8, 35.9, 35.3, 34.1, 25.7, 14.4. The characterization data is in agreement with the literature.

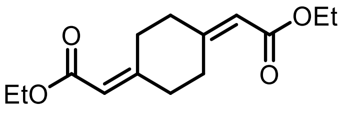

1′t, 90% yield from cyclohexane-1,4-dione[22].

^{1}H NMR (500 MHz, $CDCl_3$) δ 5.68 (s, 2H), 4.1 (q, J = 7.1, 4H), 2.96 (t, J = 6.8, 4H), 2.35 (t, J = 6.8, 4H), 1.24 (t, J = 7.1, 6H). ^{13}C NMR (125 MHz, $CDCl_3$) δ 166.6, 160.1, 114.5, 59.5, 35.7, 29.3, 14.0. The characterization data is in agreement with the literature.

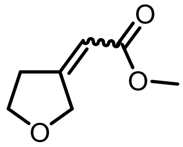

1′u, 84% yield (E/Z = 50/50) from dihydrofuran-3(2H)-one[20].

^{1}H NMR (500 MHz, $CDCl_3$) δ 5.88 (s, 0.55H), 5.82 (s, 0.45H), 4.73 (s, 1.10H), 4.39 (s, 0.90H), 3.98 (t, J = 6.9 Hz, 0.90H), 3.89 (t, J = 6.9 Hz, 1.11H), 3.73 (s, 1.35), 3.71 (s, 1.65H), 3.04 (t, J = 6.9 Hz, 0.90H), 2.73 (t, J = 6.9 Hz, 1.10H). ^{13}C NMR (125 MHz, $CDCl_3$) δ 166.8, 166.6, 163.3, 162.3, 111.0, 109.9, 72.2, 71.6, 69.0, 67.1, 51.3, 51.3, 34.6, 32.7.

HRMS-ESI (m/z): $[M+H]^+$: calculated for $C_7H_{11}O_3$ 143.0708; found 143.0704.

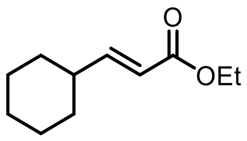

1′v, 90% yield from cyclohexanecarbaldehyde[23].

^{1}H NMR (500 MHz, $CDCl_3$) δ 6.91 (dd, J = 15.8, 6.8 Hz, 1H), 5.75 (dd, J = 15.8, 1.4 Hz, 1H), 4.18 (q, J = 7.1 Hz, 2H), 2.19-2.06 (m, 1H), 1.81-1.71 (m, 4H), 1.71-1.62 (m, 1H), 1.32-1.21 (m, 5H), 1.20-1.07 (m, 3H). ^{13}C NMR (125 MHz, $CDCl_3$) δ 167.3, 154.4, 119.0, 60.3, 40.5, 31.8, 26.1, 25.9,

14.4. The characterization data is in agreement with the literature.

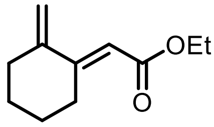

1′x, 58% yield from ethyl (E)-2-(2-oxocyclohexylidene)acetate[24].

^{1}H NMR (400 MHz, $CDCl_3$) δ5.82 (s, 1H), 4.98 (s, 1H), 4.75 (s, 1H), 4.15 (q, *J* = 7.1 Hz, 2H), 2.96-2.87 (m, 2H), 2.37-2.27 (m, 2H), 1.72-1.63 (m, 4H), 1.28 (t, *J* = 7.1 Hz, 4H). ^{13}C NMR (100 MHz, $CDCl_3$) δ167.0, 161.1, 149.8, 113.1, 110.9, 59.8, 35.5, 29.9, 26.6, 26.1, 14.4. The characterization data is in agreement with the literature.

C) Regulator **R**

i) Alkene regulators **R1**, **R2**, **R3**, and **R5** are commercially available.

ii) Alkene regulator **R4** was synthesized by Wittig reaction as described in exo-cyclic olefins section.

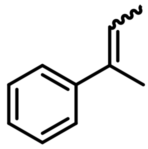

R4, 90% yield from acetophenone and ethyltriphenylphosphonium bromide by Wittig reaction as described in the previous section[18].

(E/Z = 20:80). ^{1}H NMR (500 MHz, $CDCl_3$) δ 7.46 – 7.18 (m, 5H), 5.91 (q, *J* = 6.8, 0.2H), 5.61 (q, *J* = 6.8, 0.8H), 2.08 (s, 3H), 1.84 (d, *J* = 6.8Hz, 0.6H), 1.64 (d, *J* = 6.8Hz, 2.4H). The characterization data is in agreement with the literature.

General Procedure for Transfer-Dehydroaromatization:

A) General NHC Generation Procedure:

Some of the NHCs are commercially available (**L1** and **L7**) and used directly for reactions, while some of the NHCs (**L2**, **L3**, **L4**, **L5**, **L6**) were prepared from the deprotonation of corresponding imidazolium salts (NHC*HCl or NHC*HBF_4). The procedure is shown below:

In the glove box, 0.05 mmol NHC*HCl or NHC*HBF_4 and 0.06 mmol KHMDS were added to an oven-dried test tube. After 1 mL THF was added, the mixture was stirred at r.t. for 1 hr. The resulted solution was filtered and used without further purification.

B) General [(NHC)PdH(X)] Catalyst Generation Procedure:

The catalyst was generated by modifying a literature procedure, where the palladium hydride can be observed by 1H NMR[25].

- From NHC/$PdCl_2$/$HSi(OEt)_3$

In a glove box, $PdCl_2$ and NHC (0.025 mmol each) were added to an oven-dried test tube. The mixture was dissolved with 1 mL anhydrous THF and stirred for 15 mins. Then, $HSi(OEt)_3$ (0.0375 mmol) was added to the mixture and the test tube was rinsed with 0.5 ml anhydrous THF. The whole mixture was stirred for additional 10 mins to generate the hydride catalyst.

- From NHC/$Pd(TFA)_2$/HBPin

In a glove box, $Pd(TFA)_2$ and NHC (0.025 mmol each) were added to an oven-dried test tube. The mixture was dissolved with 1 mL anhydrous THF and stirred for 15 mins. Then, HBPin (0.0375 mmol) was added to the mixture and the test tube was rinsed with 0.5 mL anhydrous THF. The whole mixture was stirred for additional 10 mins to generate the hydride catalyst.

C) General Transfer-Dehydroaromatization Procedure:

The olefin **1** or **1′** (0.25 mmol, 1 equiv.) and indicated regulator **R** (0.50 mmol, 2 equiv.) were added to the indicated catalyst. Anhydrous THF (0.5 mL) was added to rinse the residues on the inner test tube wall to the reaction mixture. This test tube was sealed properly and then put at r.t. or put into a preheated oil bath (for indicated temperature), stirred for indicated reaction time.

D) General Work-up Procedure:

The reaction (cooled to r.t. if heated) was added 4 mL hexane, and the mixture was stirred for 30 mins in the open air. The mixture was then filtered through a short plug of silica gel and rinsed with 75 mL EA/hex solution. The solvent was then removed carefully by rotary evaporation at below

30 ^{o}C. The residue was then subjected to ^{1}H NMR analysis (d1 = 10s) by using nitromethane or mesitylene as internal standard and GC-MS analysis. The product structure was confirmed by a series of NMR spectra of the isolated product.

Note: For substrates with *low boiling point* (e.g., vinyl cyclohexane, 4-vinyl-1-cyclohexene, etc.), the work-up procedure was modified as follow:

The reaction (cooled to r.t. if heated) was added 4 mL hexane, and the mixture was stirred for 30 mins in the open air. Nitromethane or mesitylene was directly added to the mixture as a standard and the mixture was mixed thoroughly for additional 10 mins. After settled, a small portion of the reaction mixture was sampled out for ^{1}H NMR (d1 = 10s) and GC-MS analysis. The product structure was also confirmed by a series of NMR spectra of the isolated product.

Representative Procedure for Transfer-Dehydroaromatization:

For Endocyclic Olefin **1**:

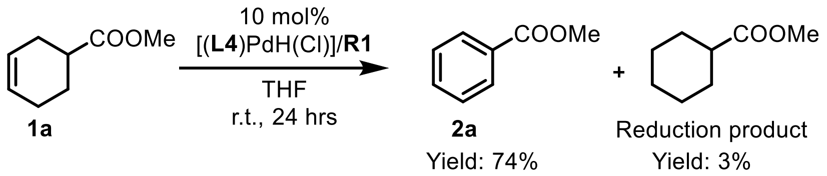

Methyl 4-cyclohexenecarboxylate **1a** (0.25 mmol, 1 equiv.) and 3,3-dimethylbutene **R1** (0.50 mmol, 2 equiv.) were added to the in-situ prepared "[(**L4**)PdH(Cl)]" catalyst (0.025 mmol) in THF (2 mL). The mixture was stirred at r.t. for 24 hrs. After that, 4 mL hexane was added and the mixture was stirred in the open air for 30 mins. Then the mixture was filtered through a short plug of silica gel and rinsed with 75 mL EA/hex (1:4). The solvent was removed carefully on rotary evaporation at below 30 °C, and the residual was mixed with 10 uL CH_3NO_2 for NMR analysis.

For Exocyclic Olefin **1′**:

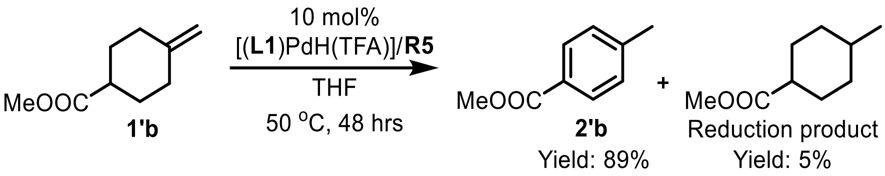

4-Methylenecyclohexane-1-carboxylate **1′b** (025 mmol, 1 equiv.) and mesityl oxide **R5** (0.50 mmol, 2 equiv.) were added to the in-situ prepared "[(**L1**)PdH(TFA)]" catalyst (0.025 mmol) in THF (2 mL). The mixture was put into a preheated oil bath (50 °C) and stirred for 48 hrs. After that, 4 mL hexane was added and the mixture was stirred in the open air for 30 mins. Then the mixture was filtered through a short plug of silica gel and rinsed with 75 mL EA/hex (1:4). The solvent was removed carefully on rotary evaporation at below 30 °C, and the residual was mixed with 10 uL mesitylene for NMR analysis.

^{1}H NMR of In-situ Palladium Hydride and Palladium Alkyl Species

A) ^{1}H NMR of in-situ palladium hydride:

The preparation method was described as in General [(NHC)PdH(X)] Catalyst Generation Procedure[25]:

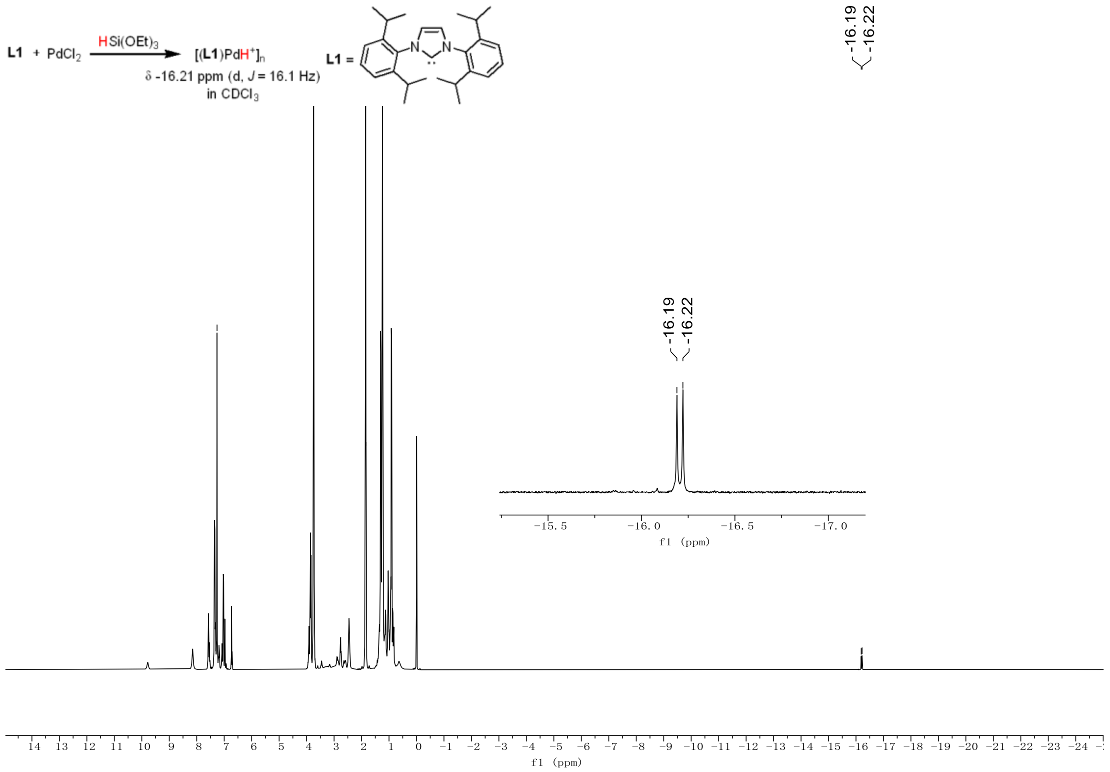

Supplementary Figure 2. ^{1}H NMR of in-situ palladium hydride

B) ^{1}H NMR of $[(NHC)PdMe(Cl)]_2$:

The preparation method was modified from literatures[26]: To an oven-dried test tube, (COD)Pd(Me)Cl (0.1 mmol) and **L1** (0.1 mmol) was added. With 2 mL of THF, the mixture was stirred at r.t. for 30 min. After filtration of the unsolved solid, the afforded light-yellow solution was concentrated. Pale-white solid was precipitated when hexane was added, and the precipitate was washed with hexane for two more times to afford the desired product in 55% yield.

^{1}H NMR (500 MHz, C_6D_6) δ 7.31 (t, J = 7.7 Hz, 4H), 7.18 (s, 4H), 6.49 (s, 4H), 3.65 – 2.54 (m, 8H), 1.40 (d, J = 6.7 Hz, 24H), 0.99 (d, J = 6.9 Hz, 24H). ^{13}C NMR (125 MHz, C_6D_6) δ 179.2, 136.4, 129.9, 128.4, 124.3, 123.8, 28.9, 26.4, 23.4, -5.36. The characterization data is in agreement with the literature.

C) ^{1}H NMR of $[(NHC)PdBn(Cl)]_2$:

The preparation method was modified from literatures[27]: To an oven-dried test-tube,

(COD)Pd(Bn)Cl[28] (0.1 mmol) and **L1** (0.1 mmol) was added. With 2 mL of THF, the mixture was stirred at r.t. for 30 min. After filtration of the unsolved solid, the afforded dark orange solution was concentrated. Pale-yellow solid was precipitated when hexane was added, and the precipitate was washed with hexane for two more times to afford the desired product in 82% yield.

^{1}H NMR (400 MHz, C_6D_6) δ 7.38 - 7.24 (m, 8H), 7.18 - 7.10 (m, 3H), 7.09 - 7.02 (m, 6H), 6.83 - 6.75 (m, 6H), 6.48 (s, 4H), 3.24 - 3.05 (m, 4H), 3.02 - 2.83 (m, 4H), 2.60 (s, 4H), 1.51 - 1.39 (m, 12H), 1.27 - 1.12 (m, 12H), 1.02 (d, 24H). ^{13}C NMR (100 MHz, C_6D_6) δ 178.3, 148.9, 136.4, 130.0, 129.9, 127.5, 124.9, 124.1, 122.6, 29.0, 26.3, 24.1, 23.0, 17.8. The characterization data is in agreement with the literature.

Control Experiment with [(NHC)PdMe(Cl)]$_2$ for dehydroaromatization:

Dehydroaromatization of the following alkenes can be done by using [(NHC)PdMe(Cl)]$_2$ in the absence of silane/borane.

Supplementary Table 1. Dehydroaromatization using [(NHC)PdMe(Cl)]$_2$

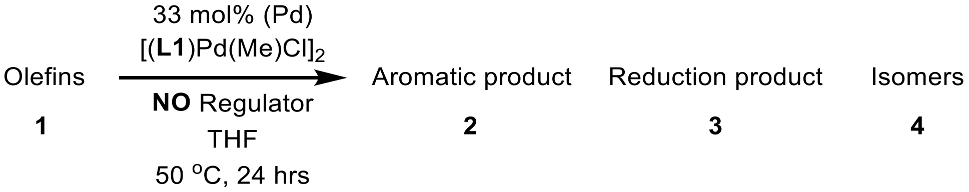

Entry	Olefins	Conv. **1**/**cat.**(%)	Aromatization product	Yield **2** (%)	Yield **3** (%)	**2:3**	Yield **4** (%)
1	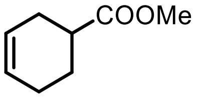	100/100	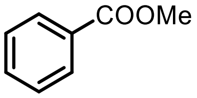	24	40	38:62	34
2	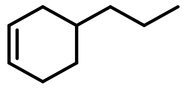	100/100	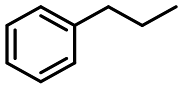	29	51	36:64	20
3	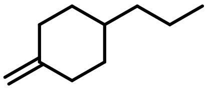	100/100	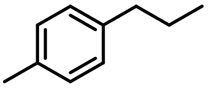	12	20	38:62	68
4	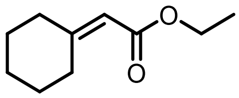	63/100	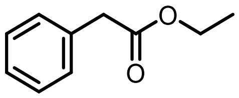	19	33	37:63	13

Condition: Olefins **1** (0.075 mmol) was added to [(**L1**)PdMe(Cl)]$_2$ catalyst (0.0125 mmol) in THF (2 mL). The mixture was put into a preheated oil bath (50 °C) and stirred for 24 hrs. After that, 4 mL hexane was added, and the mixture was stirred in the open air for 30 mins. Then the mixture was filtered through a short plug of silica gel and rinsed with 75 mL EA/hex (1:4). The solvent was removed carefully on rotary evaporation at below 30 °C, and the residual was mixed with 10 uL mesitylene for NMR analysis.

Dehydroaromatization vs. Dehydrogenative Diels-Alder Reaction:

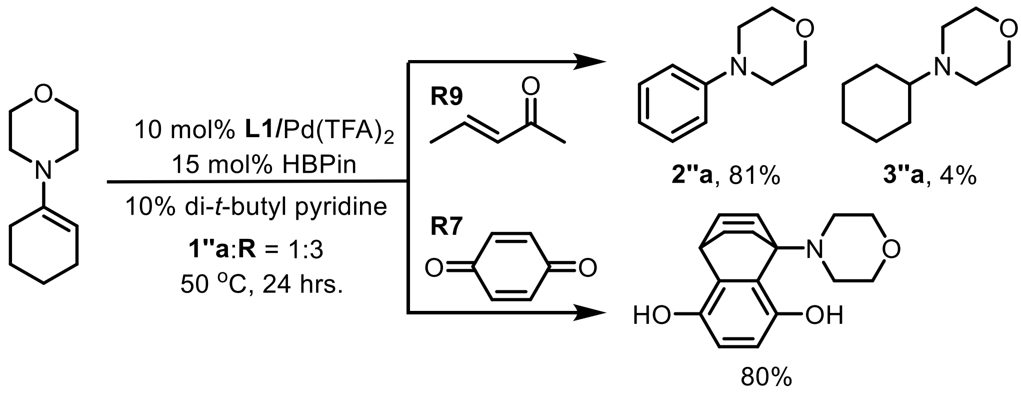

General transfer-dehydroaromatization procedure was applied, except 3 equiv. **R7**/**R9** were used and 10 mol% base was added before substrate.

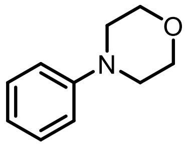

^{1}H NMR (500 MHz, $CDCl_3$) δ 7.31 – 7.27 (m, 2H), 6.95 – 6.86 (m, 3H), 3.90 – 3.84 (m, 4H), 3.19 – 3.14 (m, 4H). ^{13}C NMR (125 MHz, $CDCl_3$) δ 151.4, 129.3, 120.2, 115.9, 67.1, 49.5.

HRMS-ESI (m/z): $[M+H]^+$: calculated for $C_{10}H_{14}NO$:164.1070; found 164.1066

The characterization data is in agreement with the literature[29].

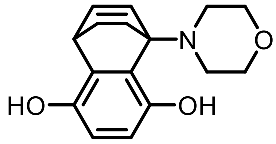

^{1}H NMR (500 MHz, DMSO-d_6) δ 8.89 (br, 1H), 6.73 (d, J = 2.5 Hz, 1H), 6.59 (d, J = 8.5 Hz, 1H), 6.53 (dd, J = 8.5, 2.5 Hz, 1H), 6.01 (dd, J = 5.4, 3.1 Hz, 1H), 3.53 – 3.41 (m, 4H), 2.59 – 2.48 (m, 4H), 2.40 – 2.21 (m, 2H), 1.90 – 1.77 (m, 1H), 1.69 – 1.58 (m, 1H), 1.35 (td, J = 13.3, 4.9 Hz, 1H). ^{13}C NMR (100 MHz, DMSO-d_6) δ 152.9, 151.29, 138.8, 125.88, 120.4, 115.9, 108.9, 106.1, 100.8, 66.5, 45.4, 29.1, 23.2, 16.6.

HRMS-ESI (m/z): $[M+H]^+$: calculated for $C_{16}H_{20}NO_3$:274.1438; found 274.1432

Chemo-Selective Transfer-Dehydroaromatization:

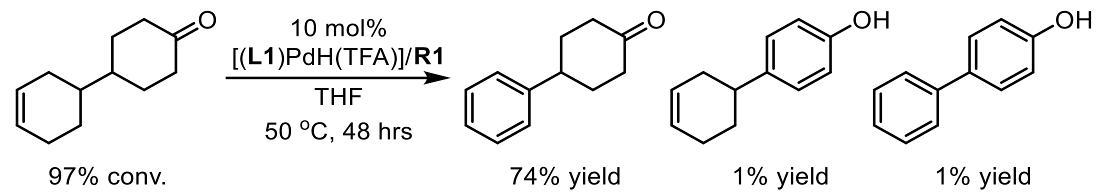

A) ^{1}H NMR comparison (phenol derivatives were obtained independently by other methods)

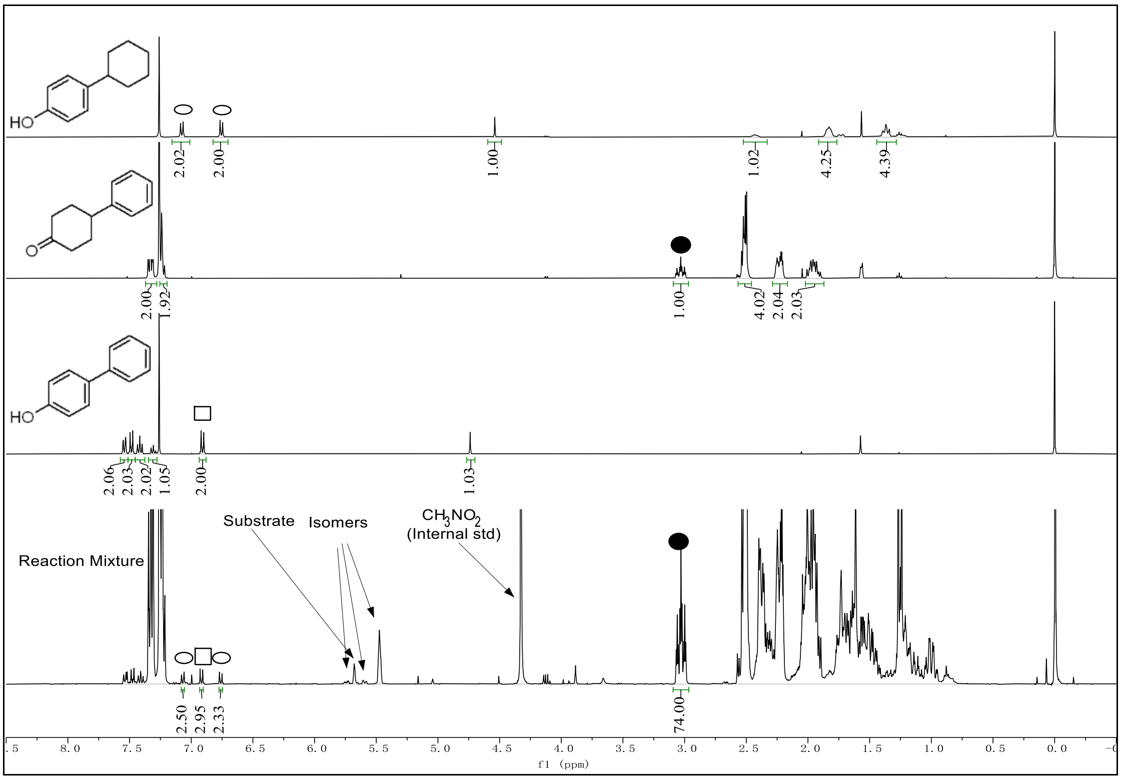

Supplementary Figure 3. Characterization of reaction mixture by ^{1}H NMR

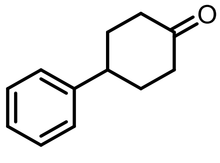

^{1}H NMR (400 MHz, $CDCl_3$) δ 7.35 – 7.30 (m, 2H), 7.26 – 7.20 (m, 3H), 3.03 (tt, J = 12.1, 3.4 Hz, 1H), 2.54 – 2.46 (m, 4H), 2.27 – 2.17 (m, 2H), 2.01 – 1.88 (m, 2H). ^{13}C NMR (100 MHz, $CDCl_3$) δ 211.4, 144.9, 128.8, 126.8, 126.7, 42.9, 41.5, 34.1.

MS (m/z) calculated for $C_{12}H_{14}O$: 174.1; found 174.1

The characterization data is in agreement with the literature[30].

B) GC-MS comparison

GC–MS: Agilent Tech 7890B GC / 5977A MS

Carrier gas: He

Column: HP–5MS 30m x 0.25mm x 0.25μm.

Temperature: Initial 50°C for 2 mins; increase 25°C/min to 150°C and hold for 2 mins; increase 5°C/min to 200°C and hold for 2 mins; increase 30°C/min to 270°C and hold for 1 mins;

Flow rate = 1.0 mL mL/min

Peaks in reaction mixture were compared with the authentic samples' retention time.

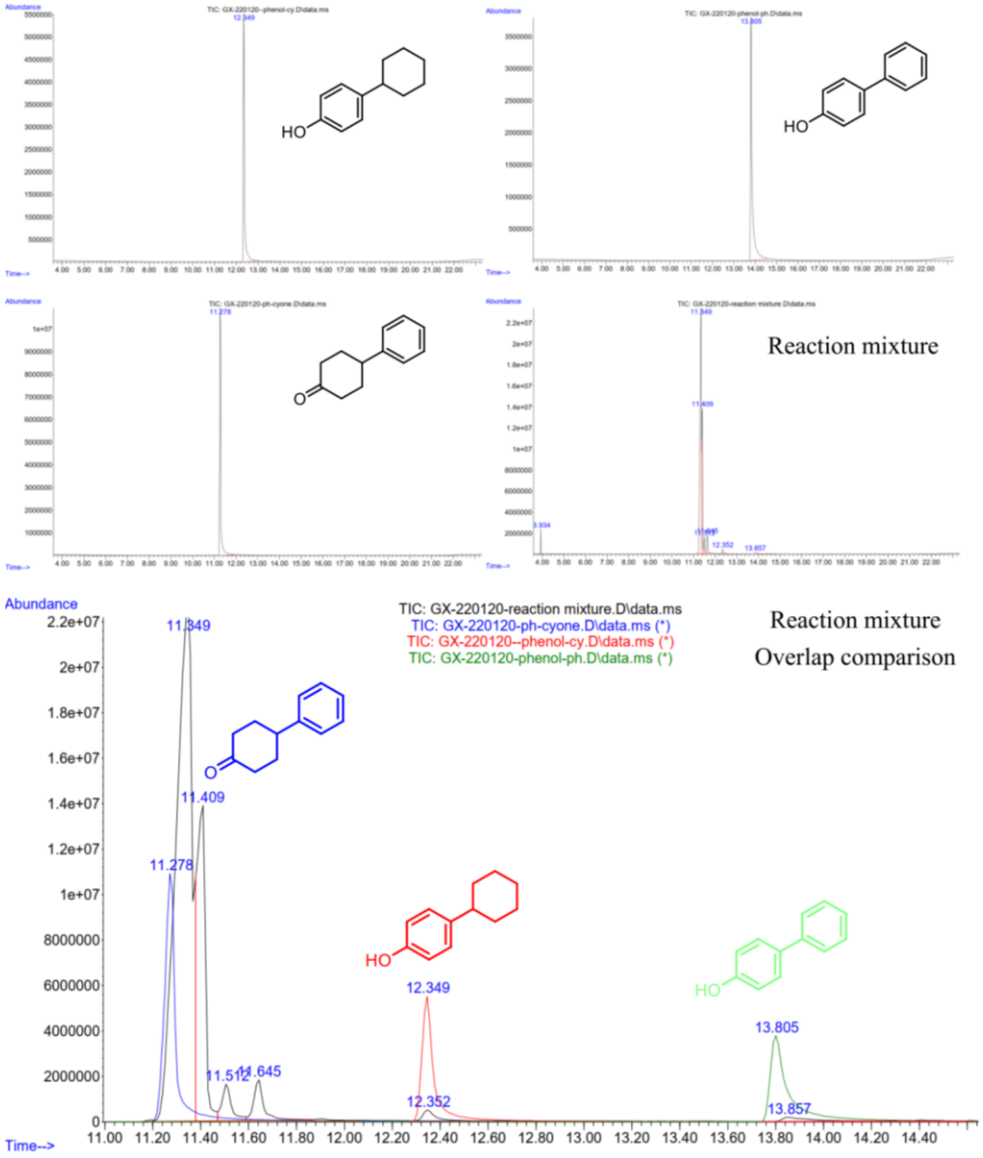

Supplementary Figure 4. Characterization of reaction mixture by GC-MS

Signal : EIC TIC: GX-220120-reaction mixture.D\data.ms

peak #	R.T. min	first scan	max scan	last scan	PK TY	peak height	corr. area	corr. % max.	% of total
1	3.934	28	33	50	BB	2071929	26472058	2.54%	1.696%
2	11.349	707	730	733	BV	23090631	1043324786	100.00%	66.850%
3	11.409	733	736	742	VV	14073933	363752663	34.86%	23.307%
4	11.512	742	746	753	VV	1567207	44961317	4.31%	2.881%
5	11.645	753	758	779	VV	1715203	50807935	4.87%	3.255%
6	12.352	816	825	849	BV	463655	16993212	1.63%	1.089%
7	13.857	958	966	1001	BB	176862	14376077	1.38%	0.921%

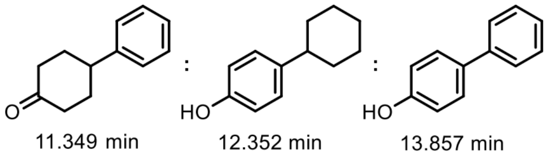

= 100: 1.63 : 1.38 = 73 : 1.2 : 1.0

Supplementary Figure 5. Calculation of product ratio by GC-MS. Linear fit calibration equations were applied, and the response factors of each product were determined. Non-calibrated product ratio = 73:1.2:1.0; Calibrated product ratio = 73:1.2:1.0

The calibration equations of peak area vs conc are as follow:

Product 1 (4-phenylcyclohexan-1-one)**:** **A**=5.92×10^{8}**c**-2.00×10^{6};

Product 2 (4-cyclohexylphenol)**:** **A**=6.26×10^{8}**c**-7.44×10^{5};

Product 3 ([1,1'-biphenyl]-4-ol)**:** **A**=5.82×10^{8}**c**-8.66×10^{5}.

Selectivity Determination:

Products and isomeric structures were assigned according to the data reported in the literature. Selectivity of the low boiling and simple non-polar hydrocarbon examples were examined by GCMS according to the literature, and the product structural identity was further confirmed by the crude NMR as shown below.

Most of the other examples were isolated in pure form and the selectivity was determined by crude product mixture. Isolated products were fully characterized by NMR and HRMS.

Supplementary Table 2. Summary of products

	List of products
Isolated products	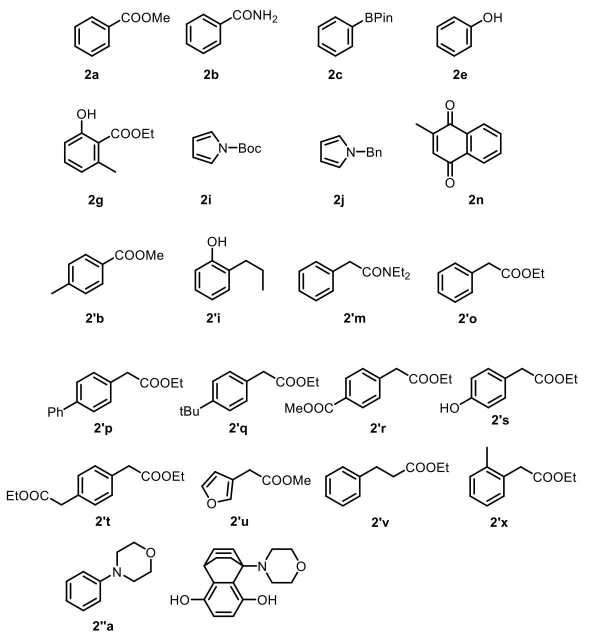
Inseparable simple non-polar hydrocarbons	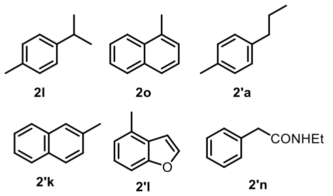
Volatile products	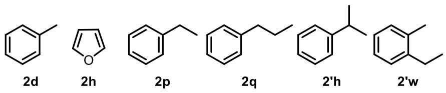

See the last section on page S65 for isolated product spectra.

GC-MS methods:

GC–MS: Agilent Tech 7890B GC / 5977A MS

Carrier gas: He

Column: HP–5MS 30m x 0.25mm x 0.25μm.

AcqMethod: 20151029-ms 1000 new program

Initial 50°C for 2 mins; increase 30°C/min to 200°C and hold for 1 min; increase 40°C/min to 280°C and hold for 15 mins; Flow rate = 1.0 mL mL/min

AcqMethod: slow T increasing

Initial 40°C for 2 mins; increase 0.1°C/min to 43°C and hold for 2 mins; increase 40°C/min to 250°C and hold for 2 mins; Flow rate = 1.0 mL mL/min

AcqMethod: C8

Initial 55°C for 3.5 mins; increase 2°C/min to 70°C and hold for 2 mins; increase 30°C/min to 280°C and hold for 1 min; Flow rate = 1.0 mL mL/min

AcqMethod: CY-C6

Initial 50°C for 3.5 mins; increase 0.5°C/min to 55°C and hold for 2 mins; increase 1°C/min to 60°C and hold for 2 mins; increase 30°C/min to 270°C and hold for 2 mins; Flow rate = 1.0 mL mL/min

AcqMethod: phenylBpin and isomers

Initial 50°C for 2 mins; increase 20°C/min to 150°C and hold for 2 mins; increase 5°C/min to 200°C and hold for 2 mins; increase 25°C/min to 270°C and hold for 1 min; Flow rate = 1.0 mL mL/min

A) Separable products

See the last section on page S65 for isolated product spectra.

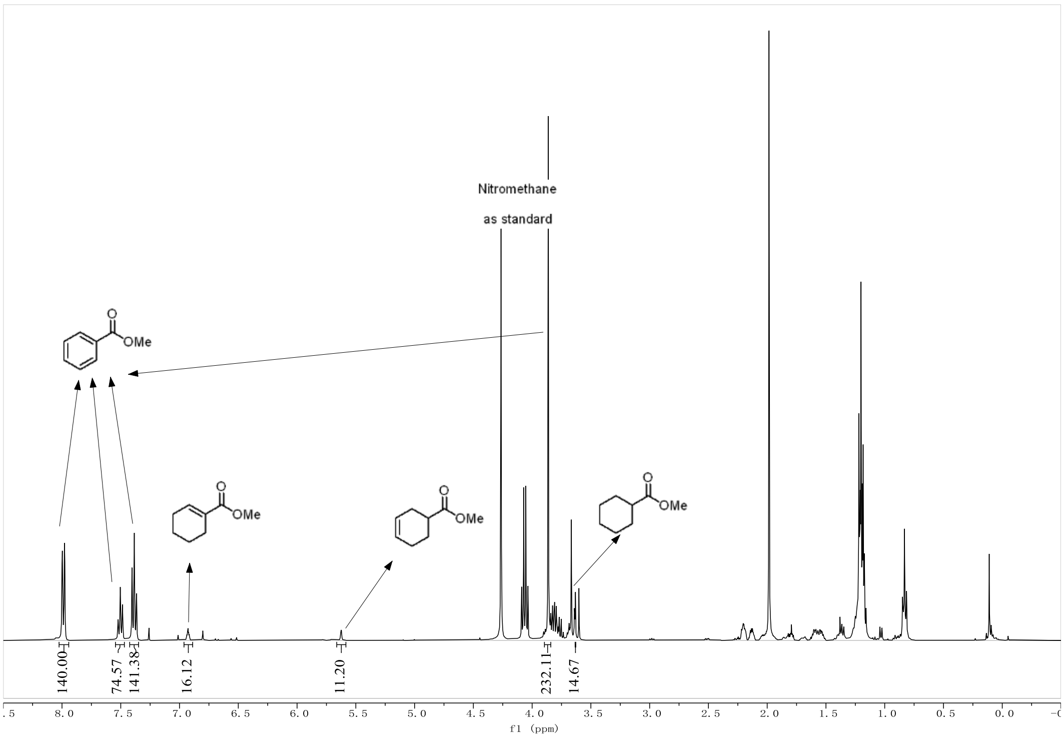

Supplementary Figure 6. Crude ^{1}H NMR| Fig.4**I**, **1a** → **2a**, 74% yield, **2a**:**3a** = 96:4. The characterization data of the purified product are the same as the literature[31].

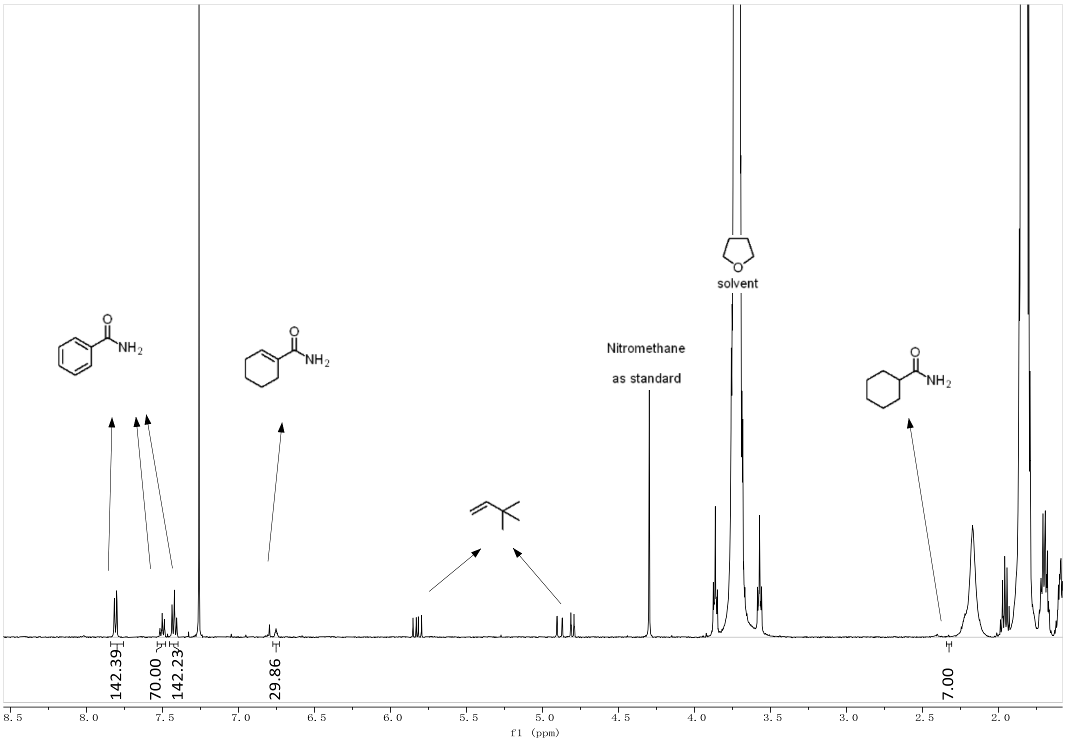

Supplementary Figure 7. Crude ^{1}H NMR| Fig.4**I**, **1b** → **2b**, 70% yield, **2b**:**3b** = 91:9. The characterization data of the purified product are the same as the literature[32].

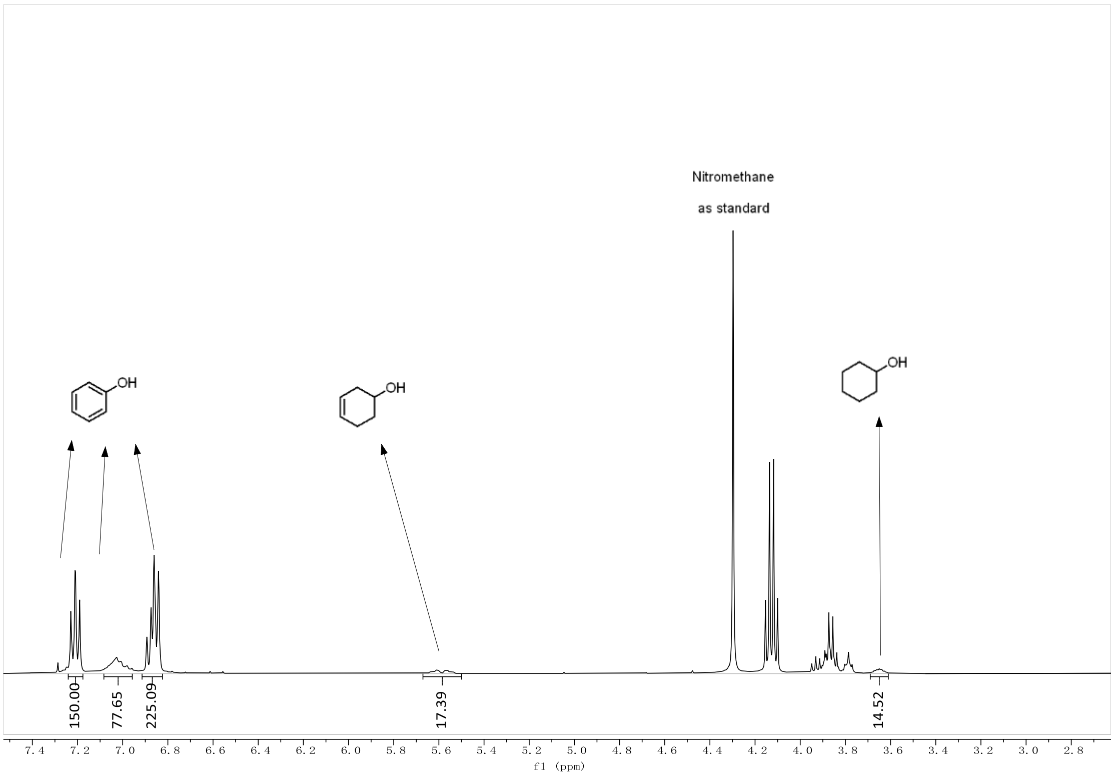

Supplementary Figure 8. Crude ^{1}H NMR| Fig.4**I**, **1e** → **2e**, 75% yield, **2e**:**3e** = 83:17. The characterization data of the purified product are the same as the literature[33].

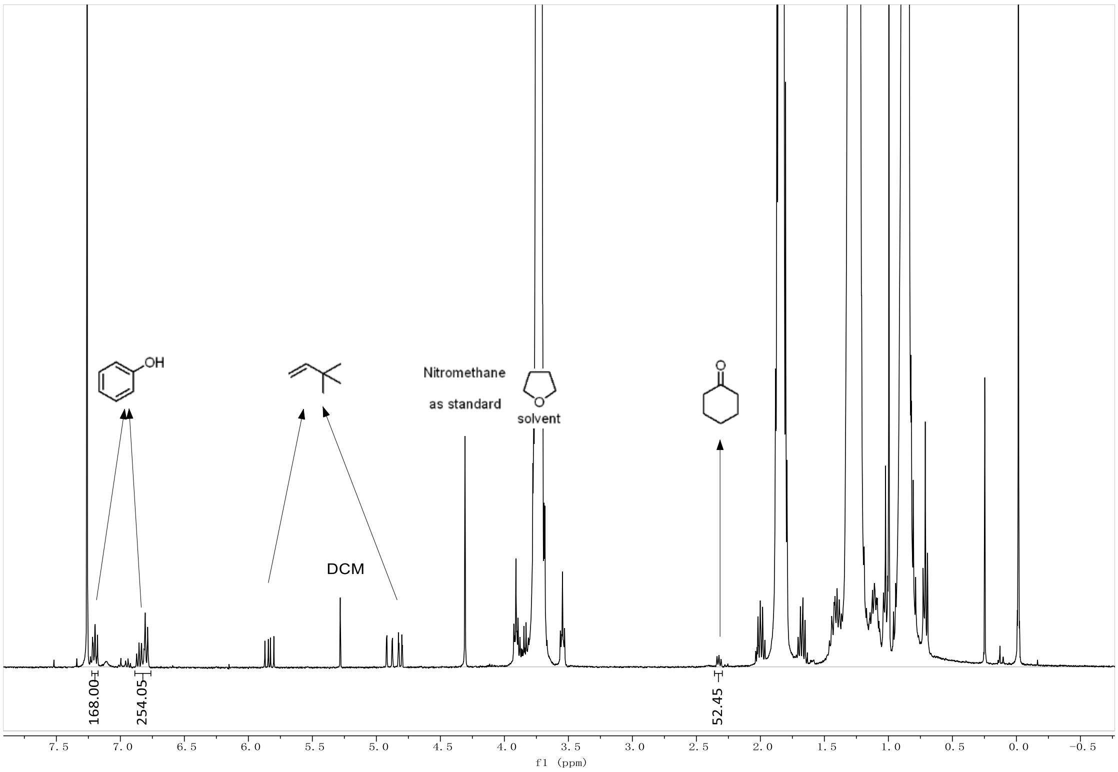

Supplementary Figure 9. Crude ^{1}H NMR| Fig.4**I**, **1f** → **2e**, 84% yield, **2e**:**3e** = 85:15. The characterization data of the purified product are the same as the literature[33].

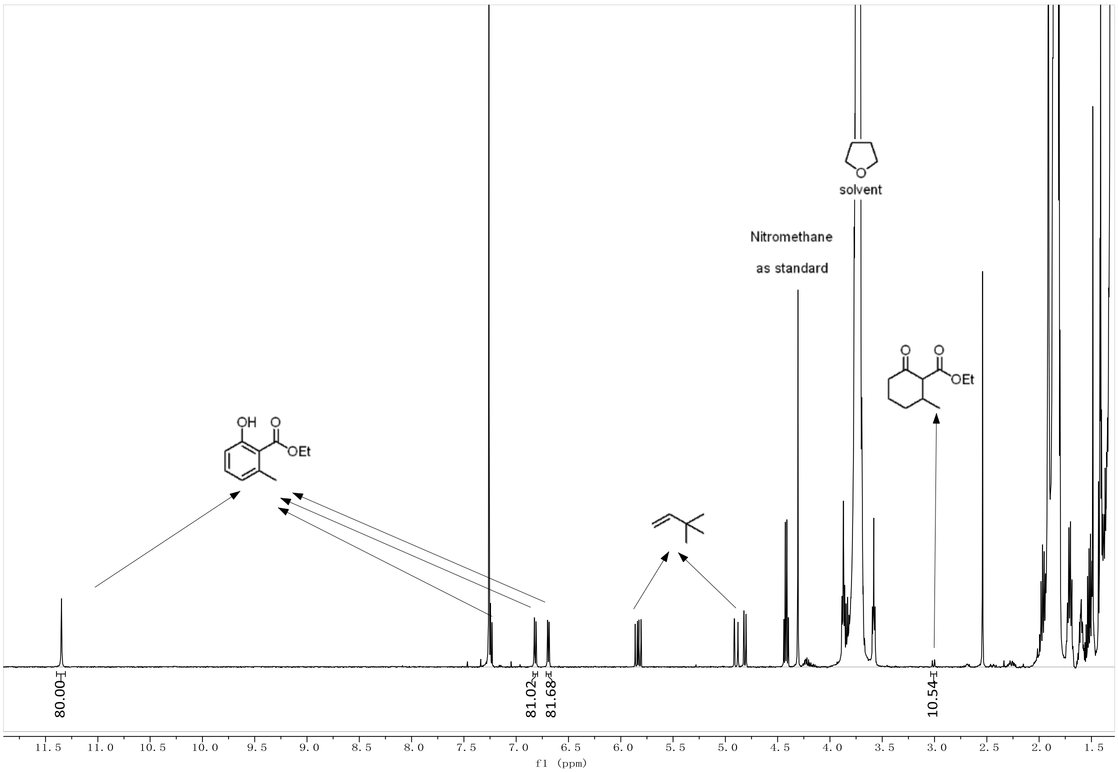

Supplementary Figure 10. Crude ^{1}H NMR| Fig.4**I**, **1g** → **2g**, 80% yield, **2g**:**3g** = 88:12. The characterization data of the purified product are the same as the literature[34].

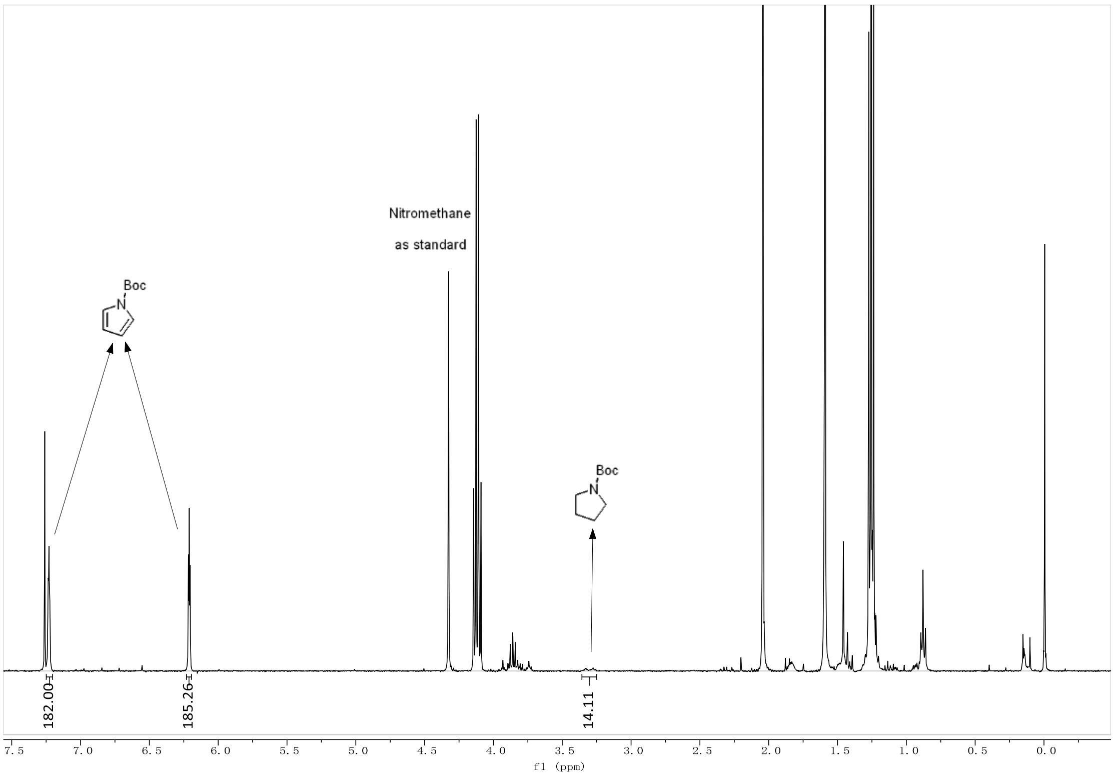

Supplementary Figure 11. Crude ^{1}H NMR| Fig.4**II**, **1i** → **2i,** 91% yield, **2i**:**3i** = 96:4. The characterization data of the purified product are the same as the literature[35].

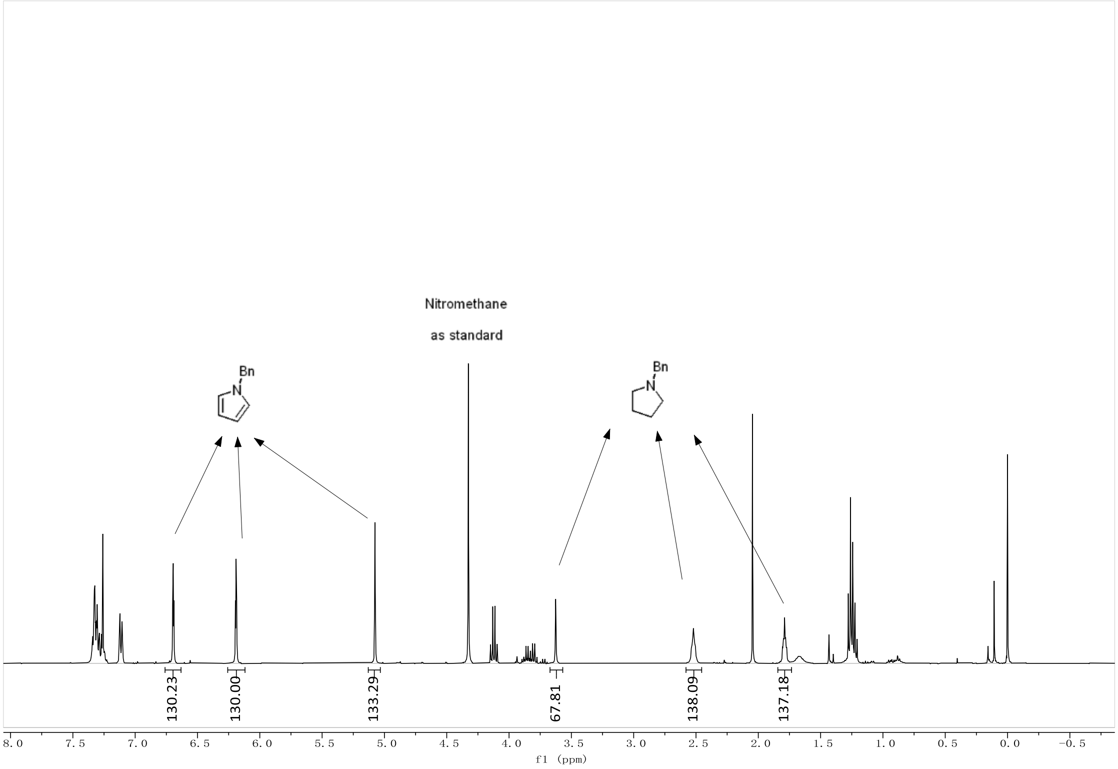

Supplementary Figure 12. Crude ^{1}H NMR| Fig.4**II**, **1j** → **2j,** 65% yield, **2j**:**3j** = 66:34. The characterization data of the purified product are the same as the literature[35].

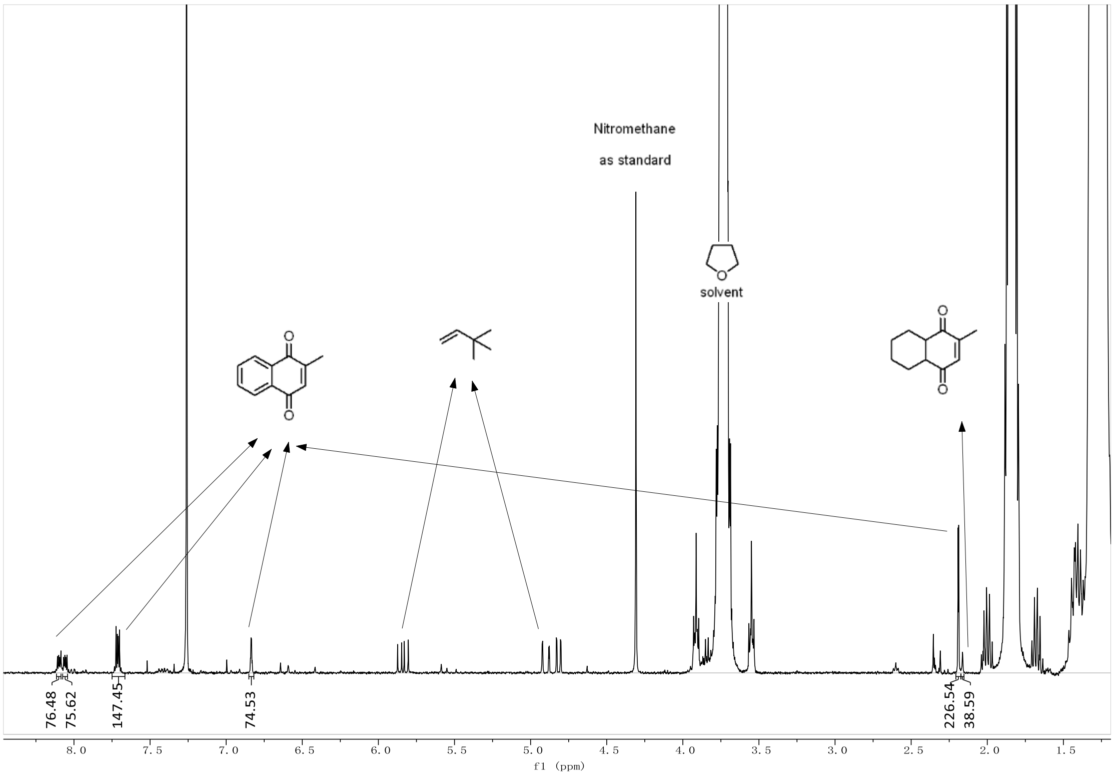

Supplementary Figure 13. Crude ^{1}H NMR| Fig.4**IV**, **1n** → **2n,** 74% yield, **2n**:**3n** = 80:20. The characterization data of the purified product are the same as the literature[36].

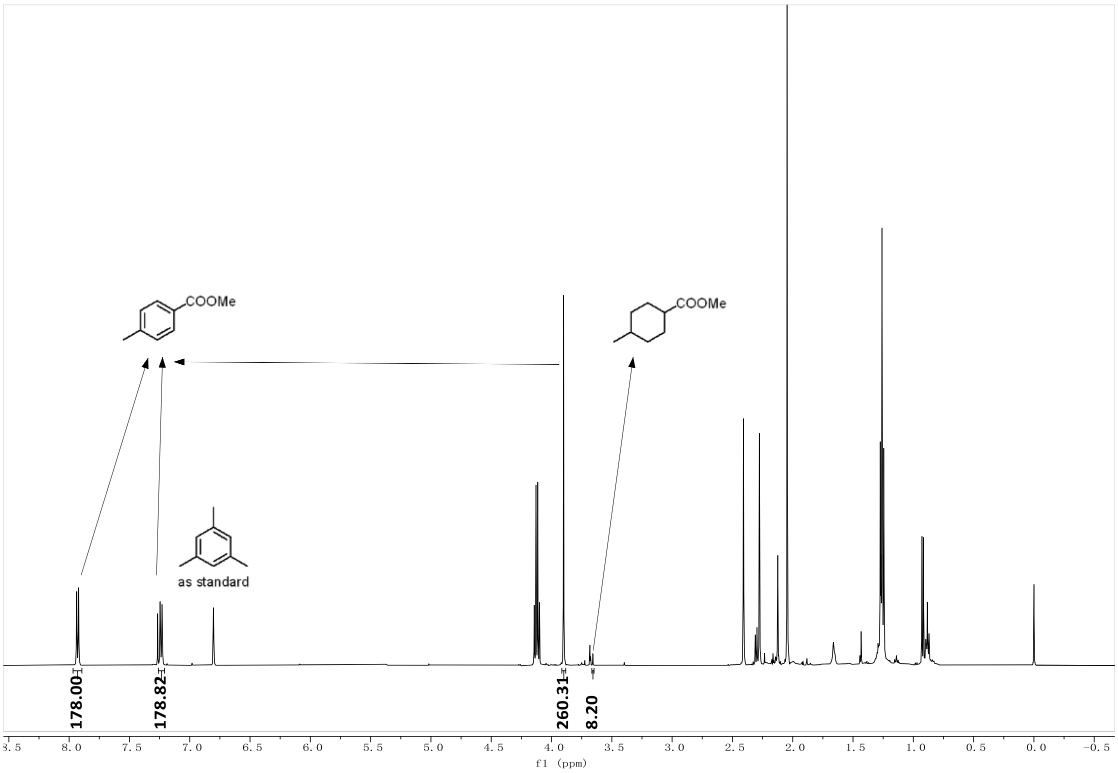

Supplementary Figure 14. Crude ^{1}H NMR| Fig.5**I**, **1′b → 2′b,** 89% yield, **2′b**:**3′b** = 95:5. The characterization data of the purified product are the same as the literature[31].

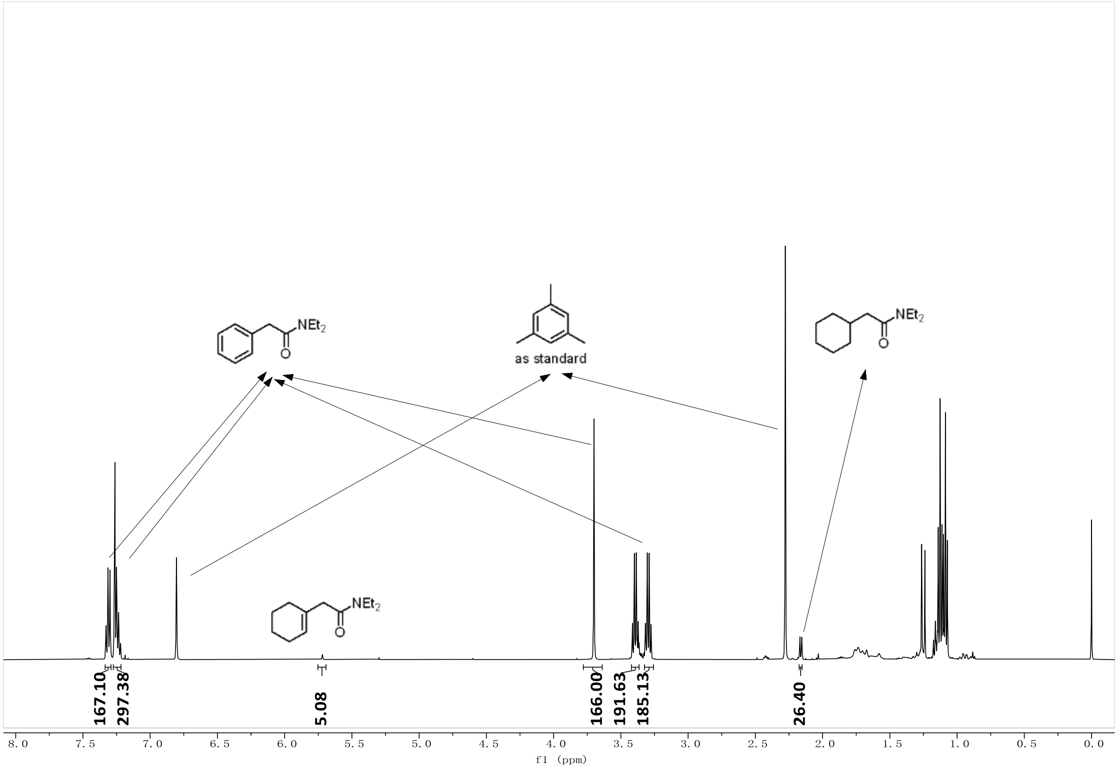

Supplementary Figure 15. Crude ^{1}H NMR| Fig.5**III**, **1′m→ 2′m,** 83% yield, **2′m**:**3′m** = 87:13. The characterization data of the purified product are the same as the literature[37].

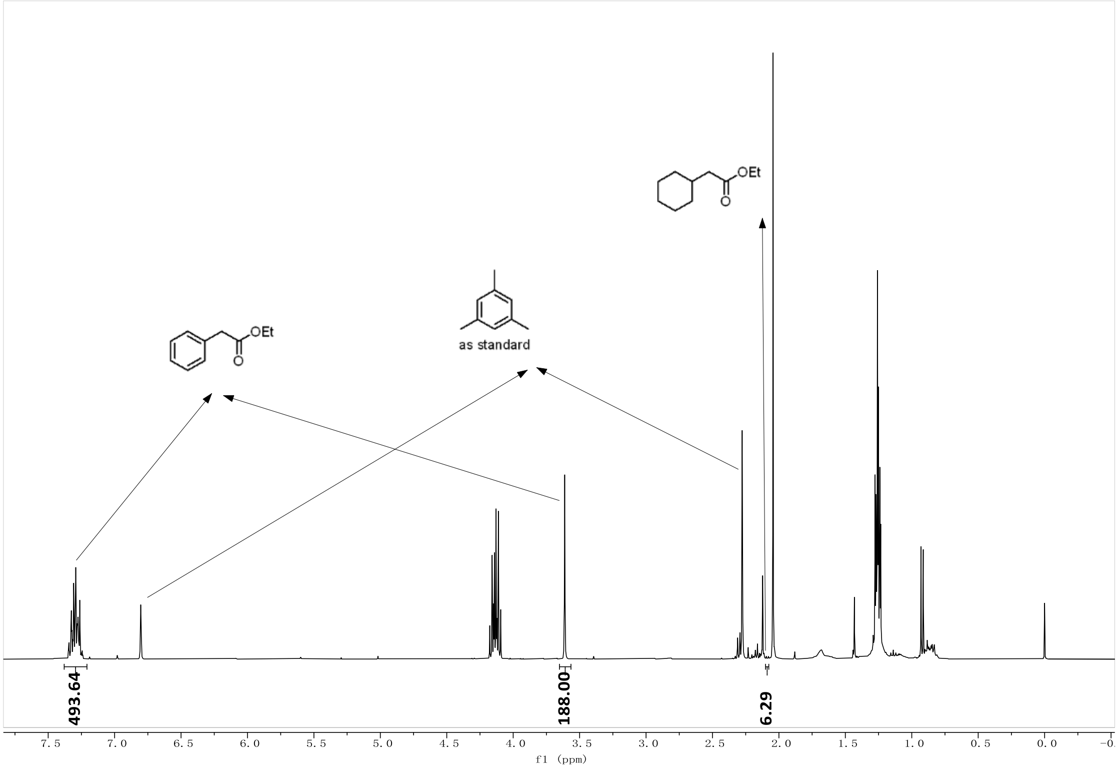

Supplementary Figure 16. Crude ^{1}H NMR| Fig.5**III**, **1′o → 2′o,** 94% yield, **2′o**:**3′o** = 97:3. The characterization data of the purified product are the same as the literature[38].

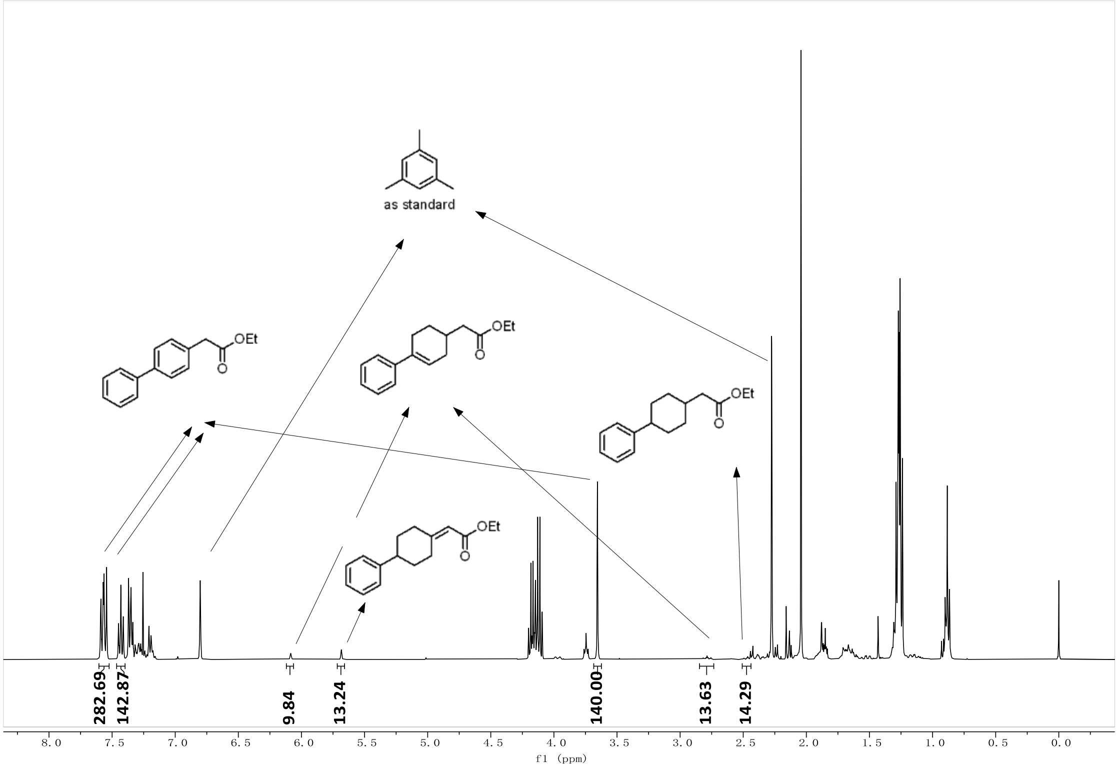

Supplementary Figure 17. Crude ^{1}H NMR| Fig.5**III**, **1′p → 2′p,** 70% yield, **2′p**:**3′p** = 81:19. The characterization data of the purified product are the same as the literature[39].

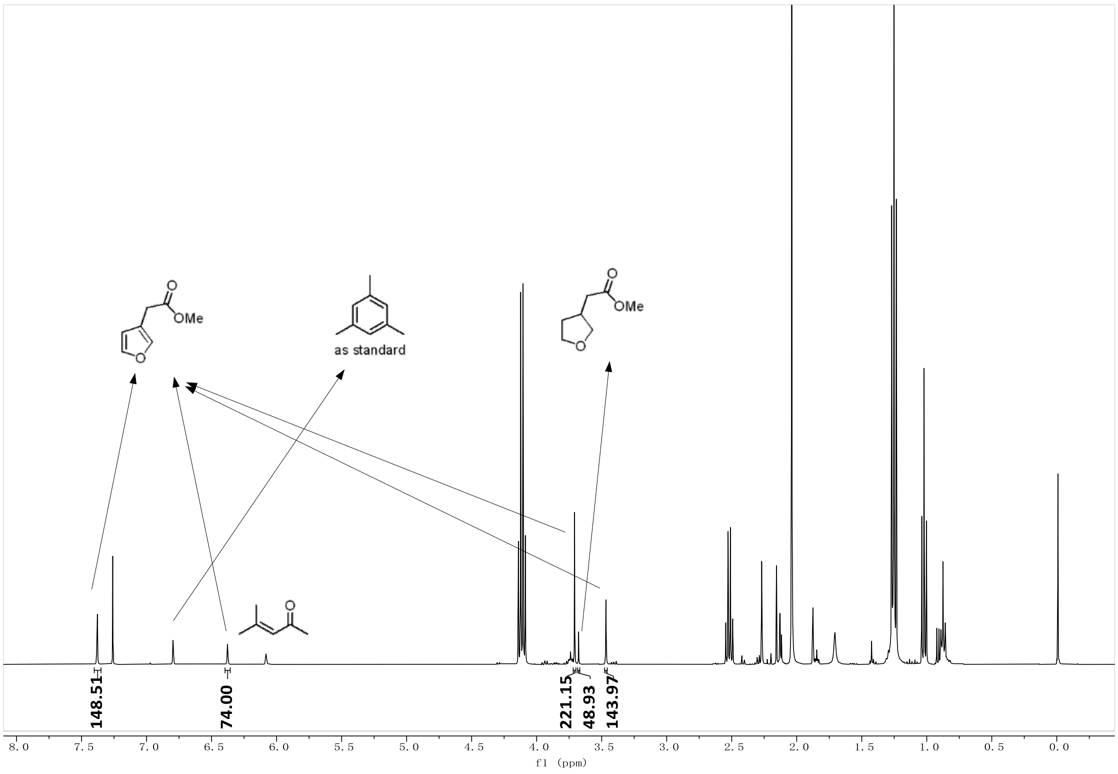

Supplementary Figure 18. Crude ^{1}H NMR| Fig.5**III**, **1′u → 2′u,** 74% yield, **2′u**:**3′u** = 82:18. The characterization data of the purified product are the same as the literature[40].

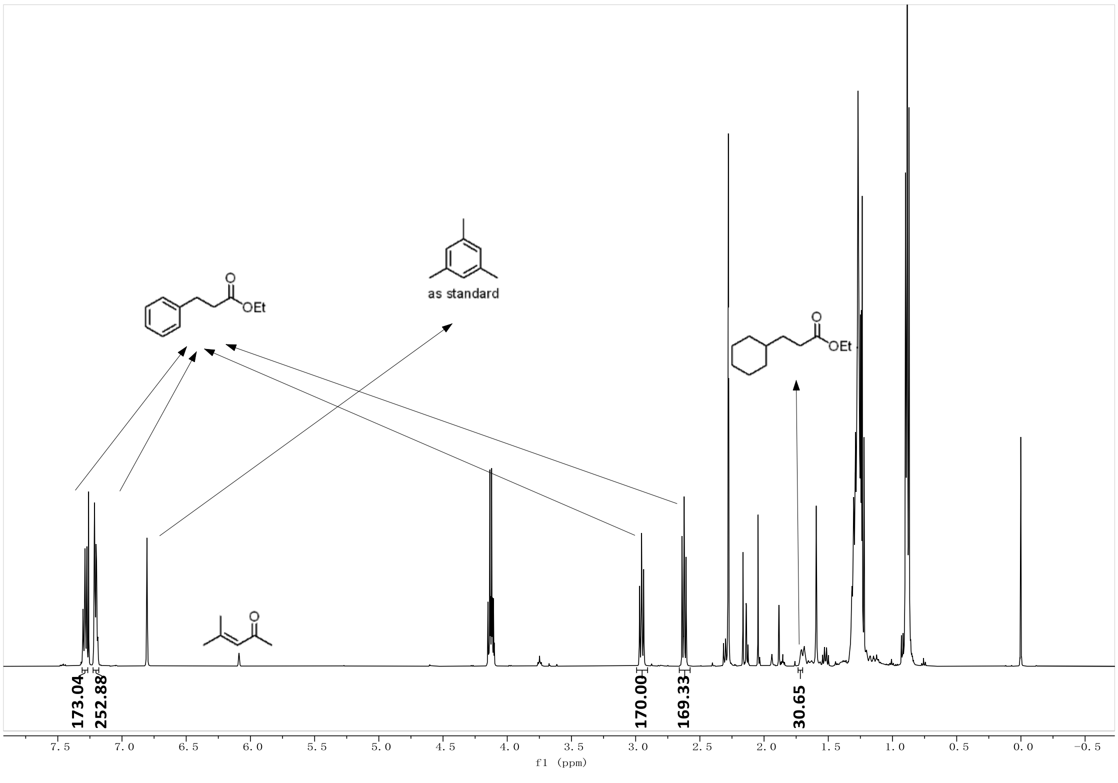

Supplementary Figure 19. Crude ^{1}H NMR| Fig.5**III**, **1′v → 2′v,** 85% yield, **2′v**:**3′v** = 85:15

The characterization data of the purified product are the same as the literature[41].

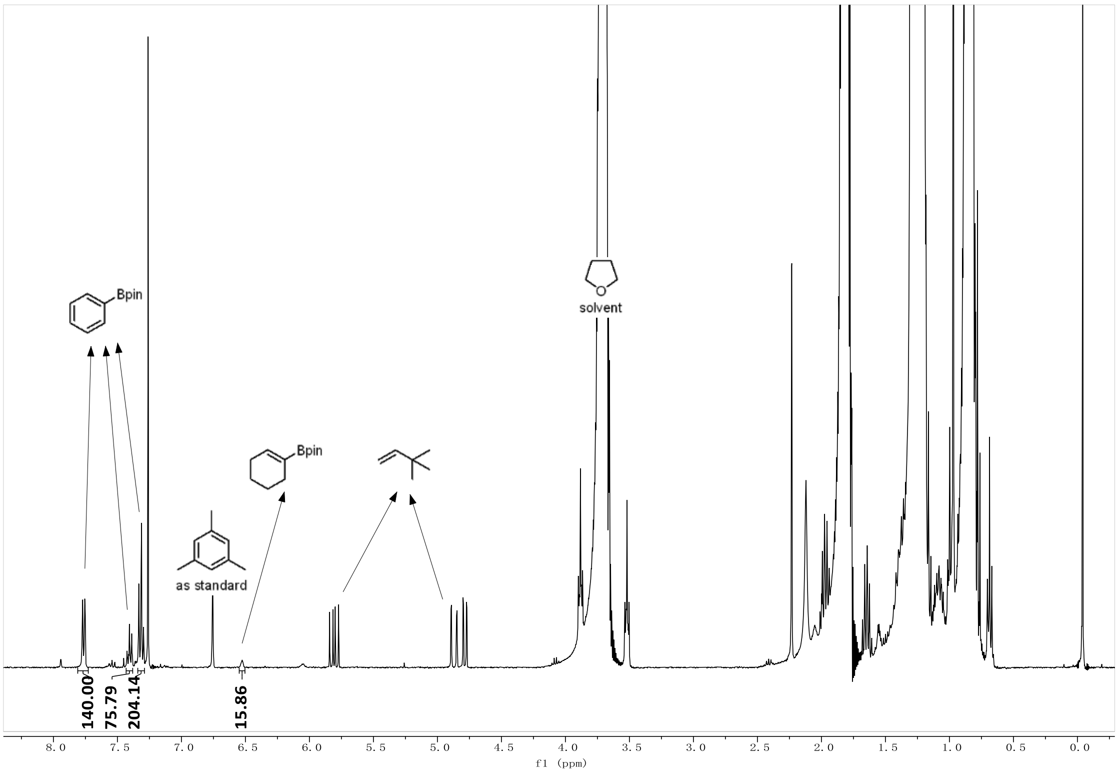

Supplementary Figure 20. Crude ^{1}H NMR| Fig.4**I**, **1c** → **2c**, 70% yield. The characterization data of the purified product are the same as the literature[42].

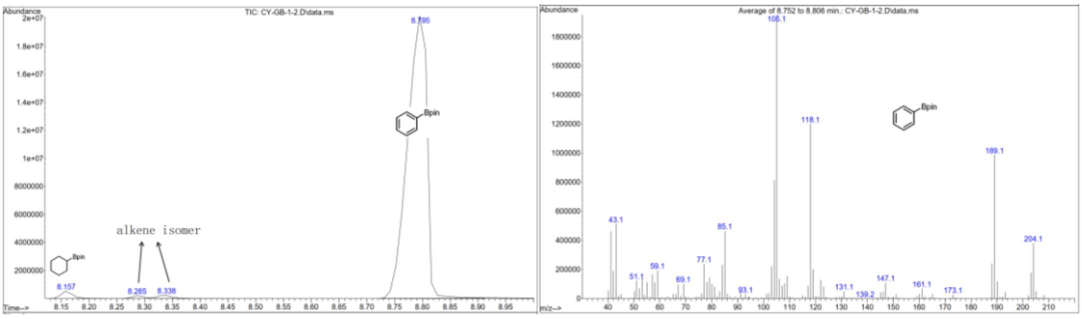


```
File        :D:\Data-1\ChenY\2021\12\24\CY-GB-1-2.D
Operator    :
Acquired    : 25 Dec 2021  18:07     using AcqMethod phenylBpin and isomers.M
Instrument  :     5977msd
Sample Name: CY-GB-1-2
Misc Info   :
Vial Number: 22
```

peak #	R.T. min	first scan	max scan	last scan	PK TY	peak height	corr. area	corr. % max.	% of total
1	8.157	428	430	435	rM	532931	777001	1.58%	1.531%
2	8.285	440	442	444	rM	206854	312805	0.64%	0.616%
3	8.338	444	447	451	rM	235537	434263	0.88%	0.856%
4	8.795	484	490	501	rM 3	20124334	49224028	100.00%	96.997%

Supplementary Figure 21. Selectivity was further determined by GCMS due to the limitation of NMR. Linear fit calibration equations were applied, and the ratio of response factors of the product **2c** and **3c** is close to one (1.0404).

2c:**3c** = 98:2 (non-calibrated); 98:2 (calibrated).

The calibration equations of peak area vs conc are as follow:

2c: A=6.50×10^8**c**-8.43×10^5; **3c: A**=6.73×10^8**c**-5.32×10^4

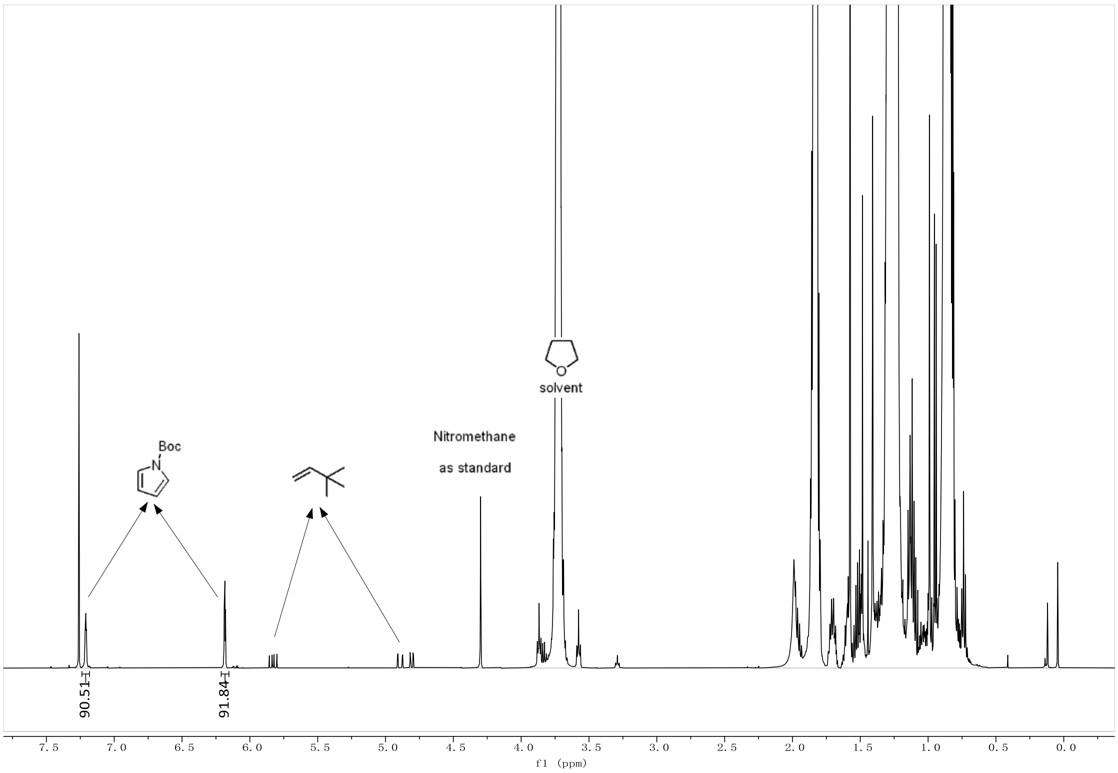

Supplementary Figure 22. Crude ^1H NMR| Fig.4**II**, **1k** → **2i,** 90% yield. The characterization data of the purified product are the same as the literature[35].

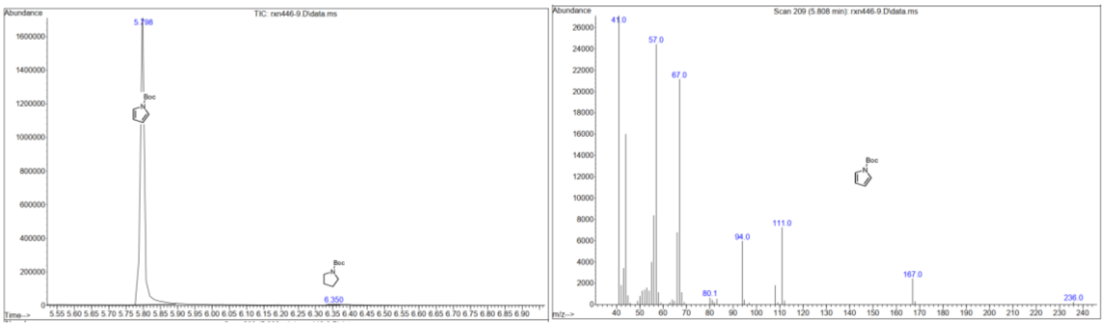

File :D:\Data-1\CWH\2021\rxn446-9.D
Operator :
Acquired : 18 Mar 2021 22:45 using AcqMethod 20151029-ms 1000 new program.M
Instrument : 5977msd
Sample Name: rxn446-9
Misc Info :
Vial Number: 5

peak #	R.T. min	first scan	max scan	last scan	PK TY	peak height	corr. area	corr. % max.	% of total
1	5.798	206	208	217	rM	1775512	1488538	100.00%	99.164%
2	6.350	258	260	262	rM 2	6168	12547	0.84%	0.836%

Supplementary Figure 23. Selectivity was further determined by GCMS due to the limitation of NMR. Linear fit calibration equations were applied, and the response factors of the product **2i** and **3i** were determined.

2i:**3i** = 99:1 (non-calibrated); >99:1 (calibrated).

The calibration equations of peak area vs conc are as follow:

2i: **A**$=2.65\times10^8$**c**$+3.37\times10^5$; **3i:** **A**$=2.52\times10^8$**c**$+1.18\times10^6$

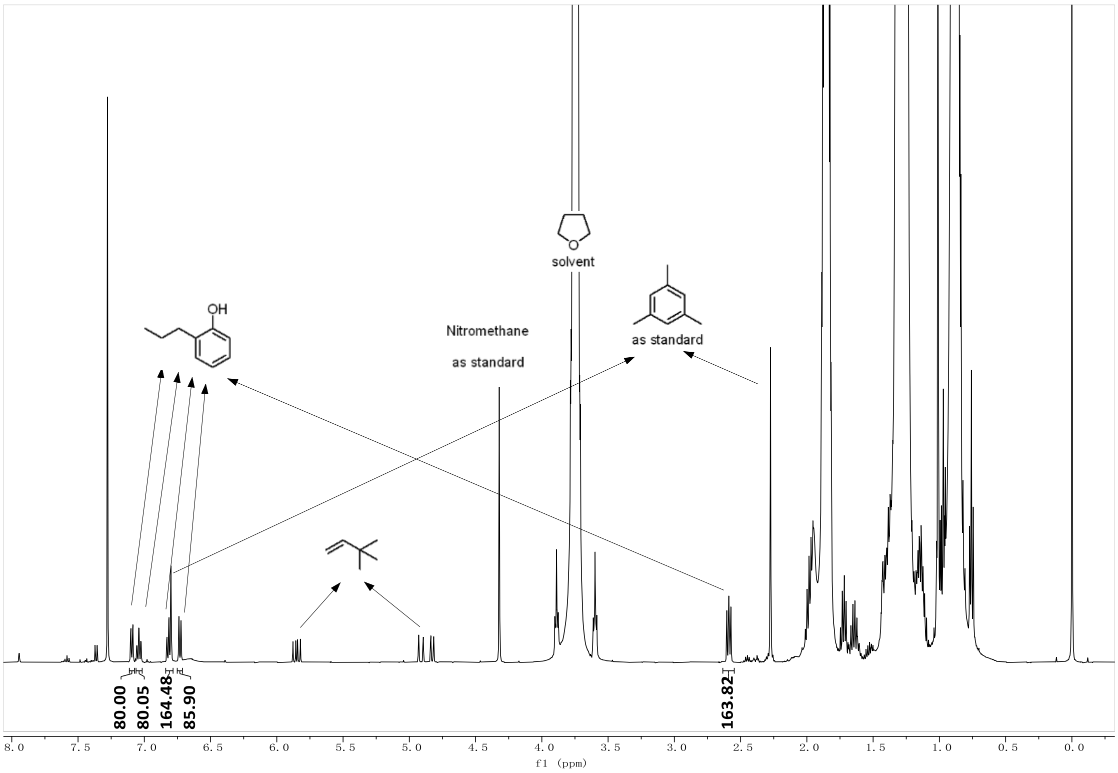

Supplementary Figure 24. Crude ^{1}H NMR| Fig.5**I**, **1′i → 2′i,** 80% yield. The characterization data of the purified product are the same as the literature[43].

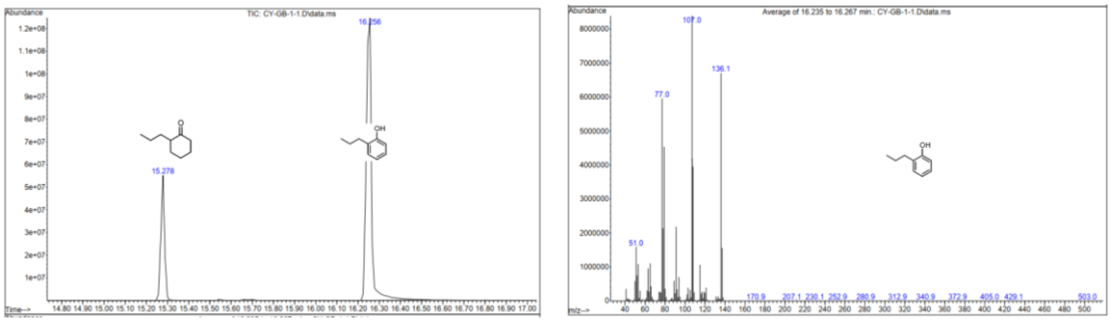

File :D:\Data-1\ChenY\2020\10\31\CY-GB-1-1.D
Operator :
Acquired : 01 Nov 2020 13:42 using AcqMethod C8.M
Instrument : 5977msd
Sample Name: CY-GB-1-1
Misc Info :
Vial Number: 42

peak #	R.T. min	first scan	max scan	last scan	PK TY	peak height	corr. area	corr. % max.	% of total
1	15.278	1097	1100	1103	rM	55099731	66879543	30.79%	23.544%
2	16.256	1188	1192	1218	rM	124632105	217181607	100.00%	76.456%

Supplementary Figure 25. Selectivity was further determined by GCMS due to the limitation of NMR. Linear fit calibration equations were applied, and the response factors of the product **2′i** and **3′i** were determined.

2′i: **3′i** = 76:24 (non-calibrated); 74:26 (calibrated).

The calibration equations of peak area vs conc are as follow:

2′i: A=4.30×10^{8}**c**-9.20×10^{5}; **3′i: A**=3.90×10^{8}**c**-12.20×10^{5}

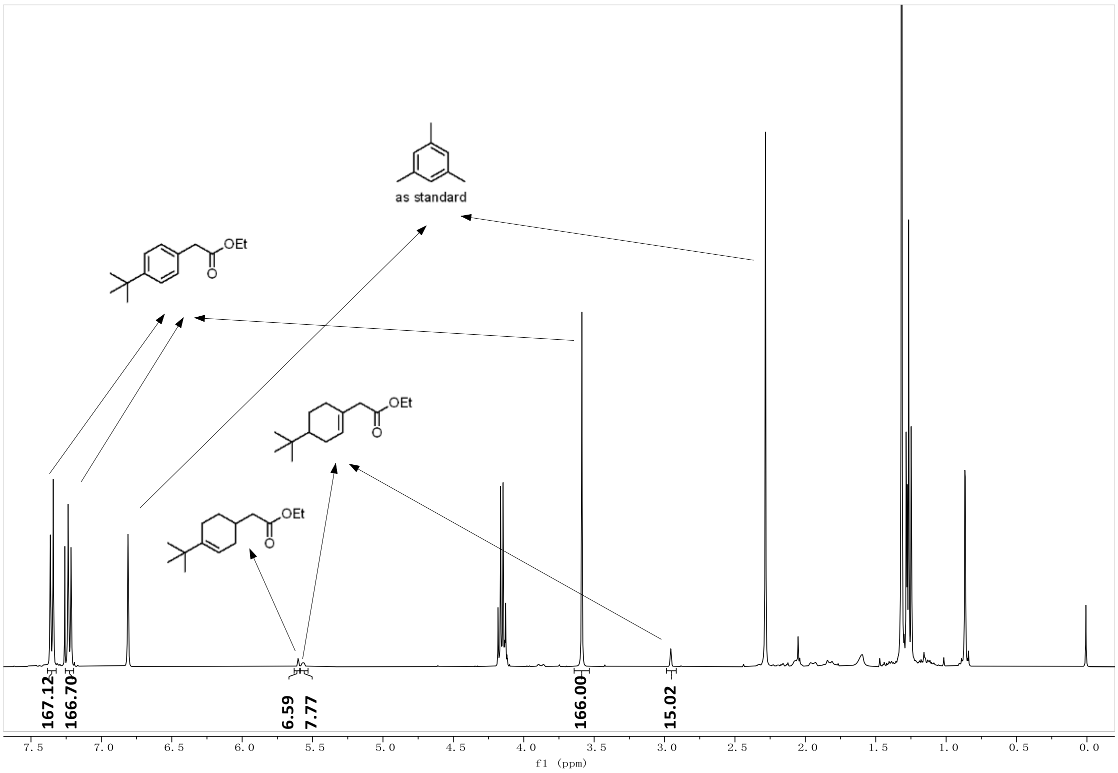

Supplementary Figure 26. Crude ^{1}H NMR| Fig.5**III**, **1′q → 2′q,** 83% yield. The characterization data of the purified product are the same as the literature[44].

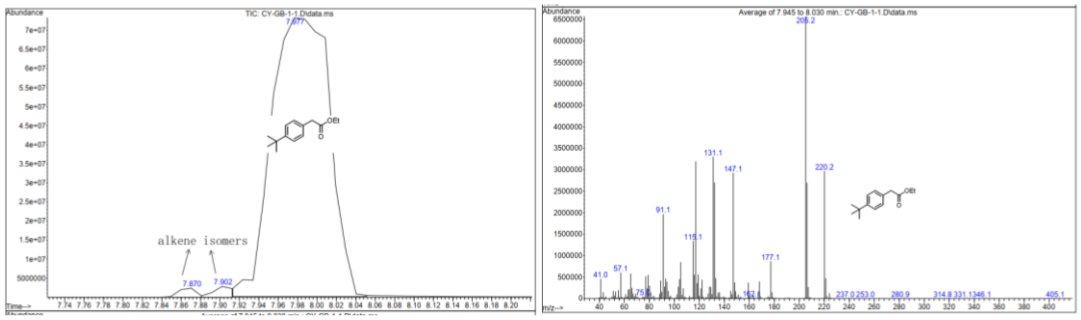

File : D:\Data-1\ChenY\2021\04\17\CY-GB-1-1.D
Operator :
Acquired : 19 Apr 2021 19:58 using AcqMethod 20151029-ms 1000 new program.M
Instrument : 5977msd
Sample Name: CY-GB-1-1
Misc Info :
Vial Number: 17

peak #	R.T. min	first scan	max scan	last scan	PK TY	peak height	corr. area	corr. % max.	% of total
1	7.870	400	403	404	rM	2384935	3125477	1.02%	0.997%
2	7.902	404	406	407	rM	2841690	4036051	1.32%	1.287%
3	7.977	407	413	420	rM 4	73132048	306376879	100.00%	97.716%

Supplementary Figure 27. Selectivity was further determined by GCMS due to the limitation of NMR. **2′q**:**3′q** = >99:1 by crude GC-MS. The retention time of **3′q** is 7.775 min, which was not detected in the reaction mixture.

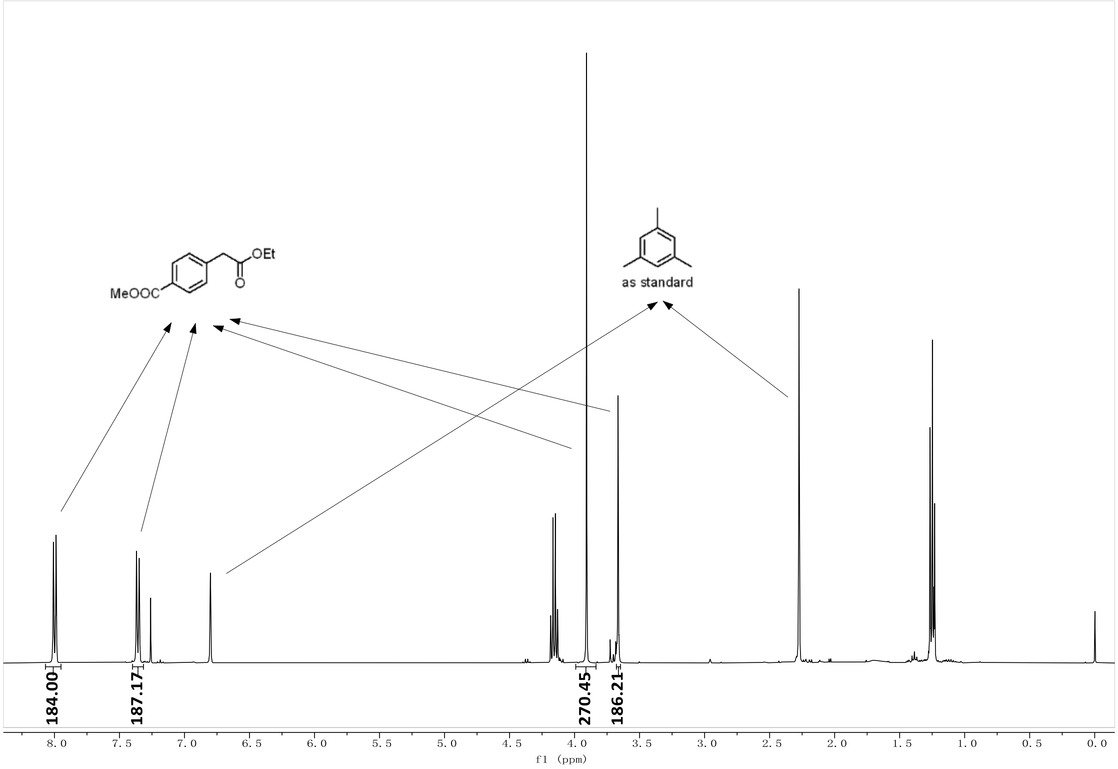

Supplementary Figure 28. Crude ^{1}H NMR| Fig.5**III**, **1′r → 2′r,** 92% yield. The characterization data of the purified product are the same as the literature[45].

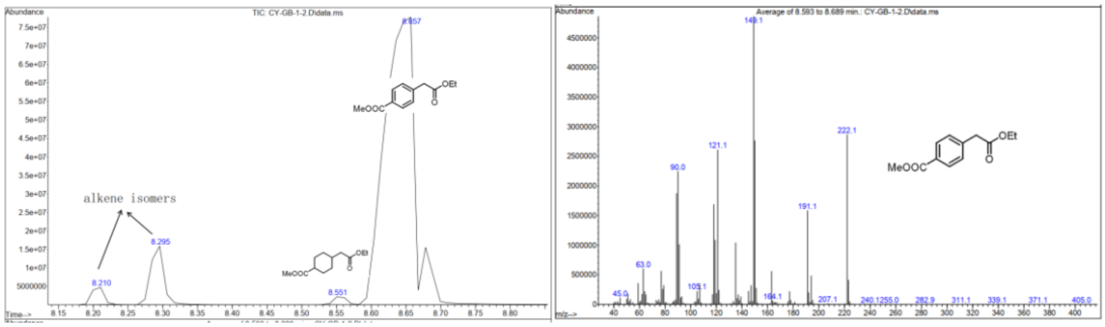

File :D:\Data-1\ChenY\2021\04\17\CY-GB-1-2.D
Operator :
Acquired : 19 Apr 2021 21:21 using AcqMethod 20151029-ms 1000 new program.M
Instrument : 5977msd
Sample Name: CY-GB-1-2
Misc Info :
Vial Number: 18

peak #	R.T. min	first scan	max scan	last scan	PK TY	peak height	corr. area	corr. % max.	% of total
1	8.210	432	435	437	rM	4644794	6031482	2.54%	2.250%
2	8.295	440	443	447	rM	15891109	21265553	8.94%	7.934%
3	8.551	465	467	470	rM	2147964	2948964	1.24%	1.100%
4	8.657	470	477	486	rM 3	77809467	237790968	100.00%	88.716%

Supplementary Figure 29. Selectivity was further determined by GCMS due to the limitation of NMR. Linear fit calibration equations were applied, and the response factors of the product **2′r** and **3′r** were determined.

2′r: **3′r** = >99:1 (non-calibrated); 98:2 (calibrated).

The calibration equations of peak area vs conc are as follow:

2′r: A=1.06×10^{9}**c**-1.49×10^{6}; **3′r: A**=5.92×10^{8}**c**+6.23×10^{5}

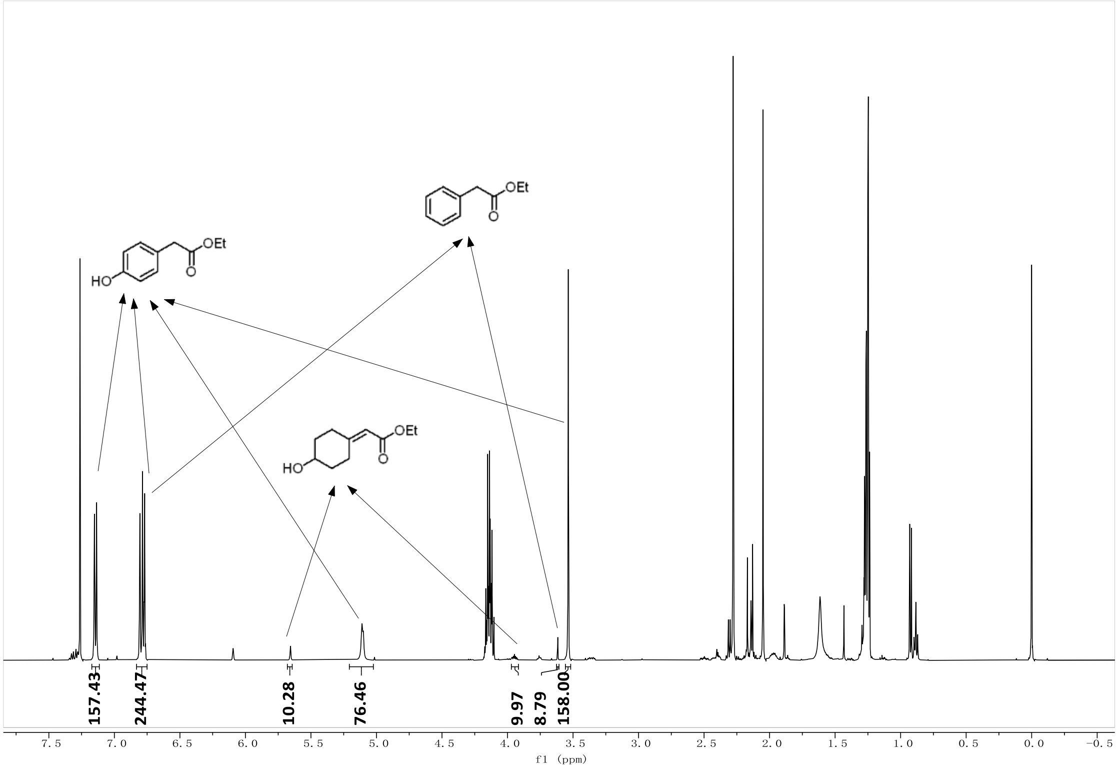

Supplementary Figure 30. Crude ^{1}H NMR| Fig.5**III**, **1′s → 2′s,** 87% yield. The characterization data of the purified product are the same as the literature[46].

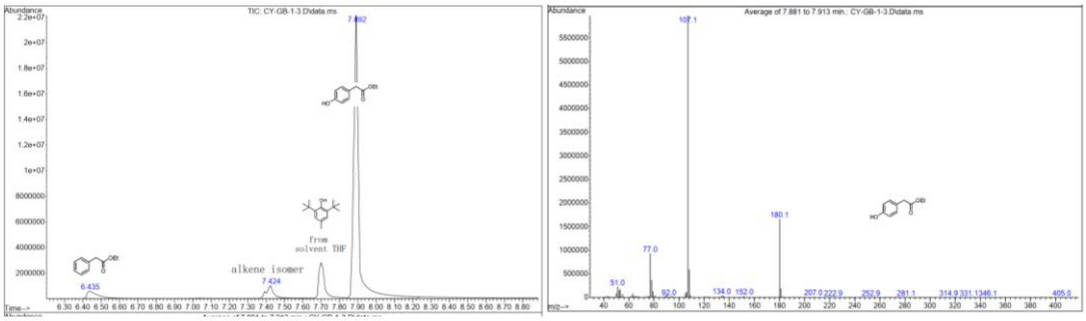


```
File        :D:\Data-1\ChenY\2021\04\12\CY-GB-1-3.D
Operator    :
Acquired    : 14 Apr 2021   18:01     using AcqMethod 20151029-ms 1000 new program.M
Instrument  :    5977msd
Sample Name: CY-GB-1-3
Misc Info   :
Vial Number: 48
```

peak #	R.T. min	first scan	max scan	last scan	PK TY	peak height	corr. area	corr. % max.	% of total
1	6.435	265	268	283	rM	575884	2369989	5.61%	4.992%
2	7.424	355	361	372	rM 2	1017621	2876702	6.81%	6.060%
3	7.892	401	405	437	rM	22108850	42224660	100.00%	88.948%

Supplementary Figure 31. Selectivity was further determined by GCMS due to the limitation of NMR. **2′s**:**3′s** = >99:1 by crude GC-MS. The retention time of **3′s** is 7.286 min, which was not detected in the reaction mixture.

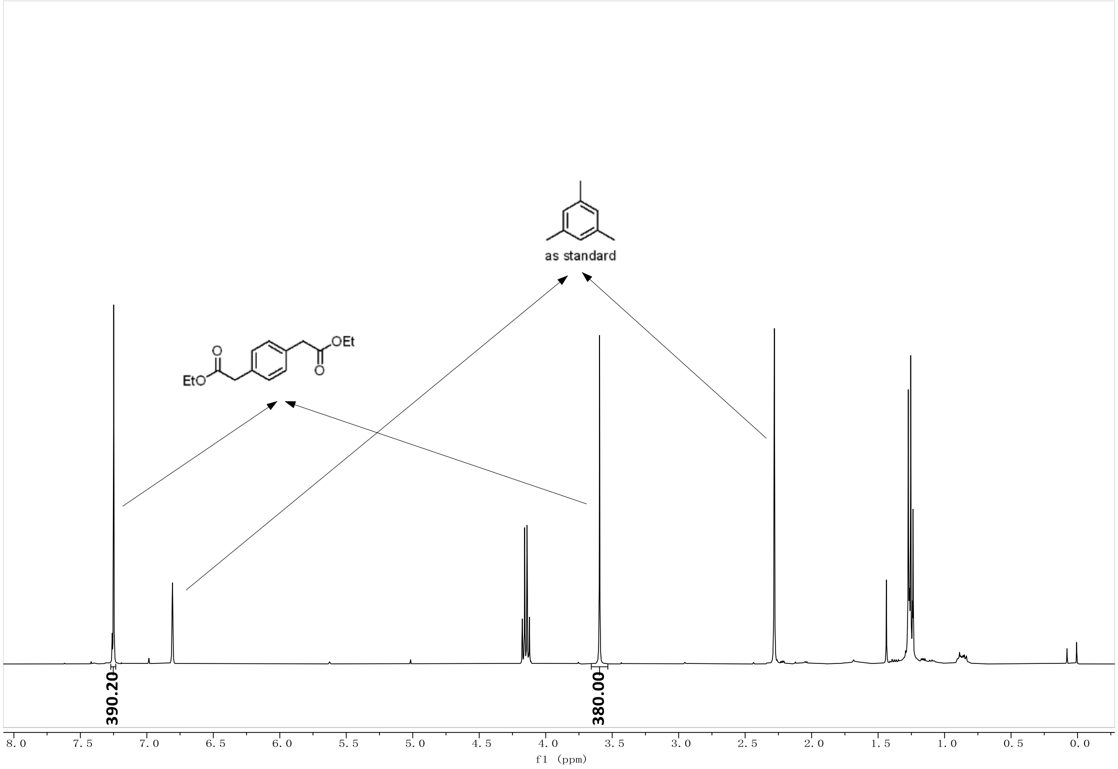

Supplementary Figure 32. Crude ^{1}H NMR| Fig.5**III**, **1′t → 2′t,** 95% yield. The characterization data of the purified product are the same as the literature[47].

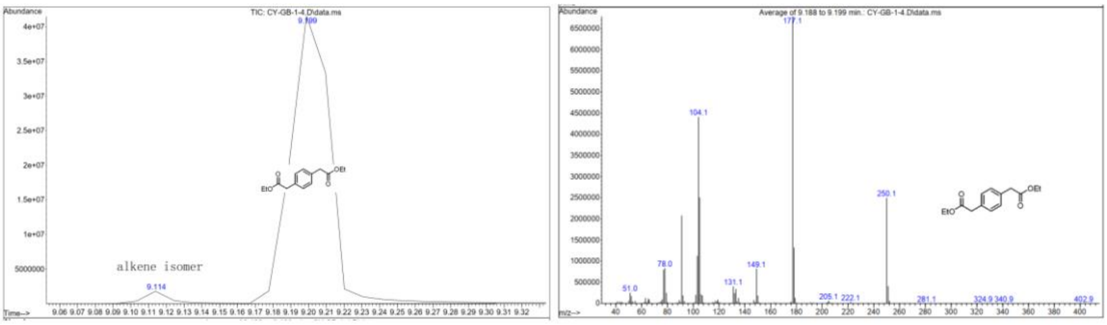


```
File       :D:\Data-1\ChenY\2021\03\29\CY-GB-1-4.D
Operator   :
Acquired   : 31 Mar 2021  15:19     using AcqMethod 20151029-ms 1000 new program.M
Instrument :    5977msd
Sample Name: CY-GB-1-4
Misc Info  :
Vial Number: 50
```

peak #	R.T. min	first scan	max scan	last scan	PK TY	peak height	corr. area	corr. % max.	% of total
1	9.114	518	520	523	rM	1776925	1766725	2.76%	2.687%
2	9.199	525	528	538	rM	41573862	63977391	100.00%	97.313%

Supplementary Figure 33. Selectivity was further determined by GCMS due to the limitation of NMR. **2′t**:**3′t** = >99:1 by crude GC-MS. The retention time of **3′t** is 8.976 min, which was not detected in the reaction mixture.

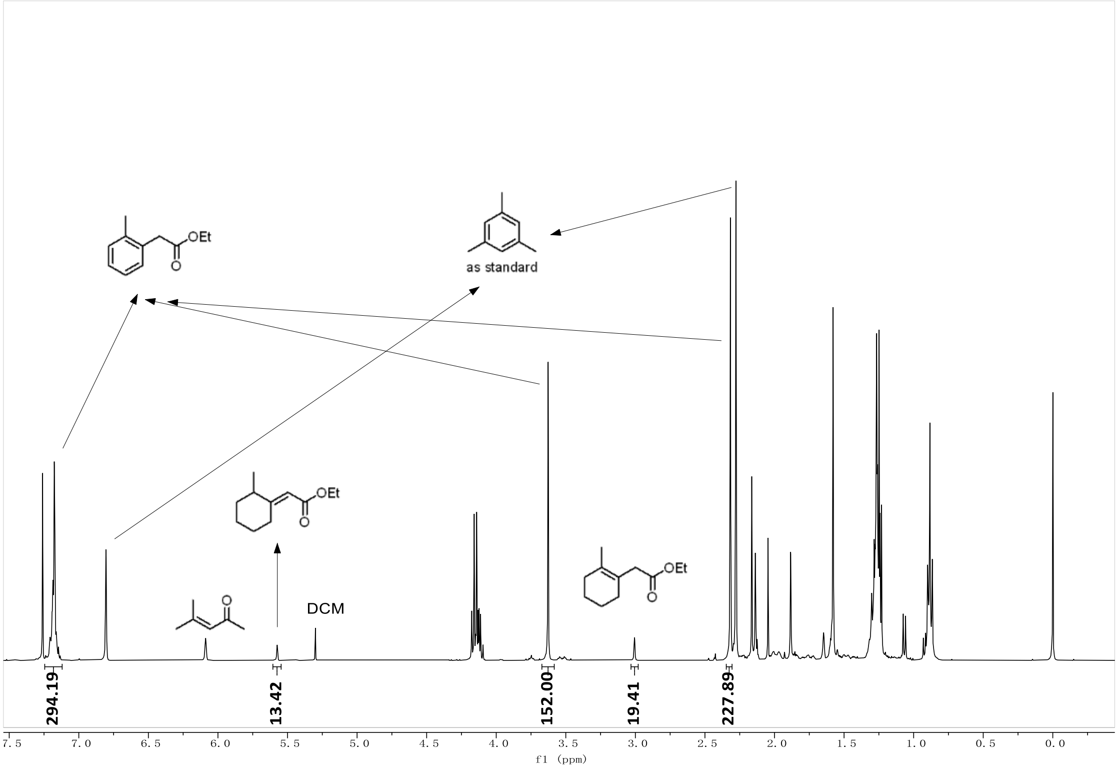

Supplementary Figure 34. Crude ^{1}H NMR| Fig.5**IV**, **1′x → 2′x,** 73% yield (>95 conversion). The characterization data of the purified product are the same as the literature[48].

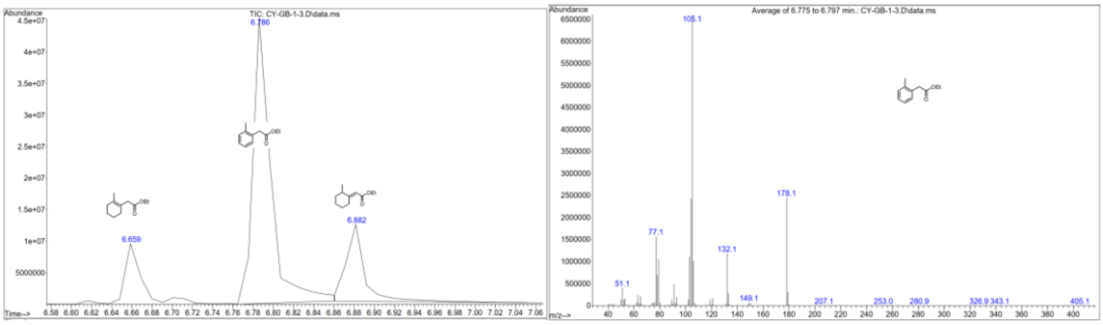

File :D:\Data-1\ChenY\2021\10\19\CY-GB-1-3.D
Operator :
Acquired : 20 Oct 2021 14:44 using AcqMethod 20151029-ms 1000 new program.M
Instrument : 5977msd
Sample Name: CY-GB-1-3
Misc Info :
Vial Number: 3

peak #	R.T. min	first scan	max scan	last scan	PK TY	peak height	corr. area	corr. % max.	% of total
1	6.659	284	289	295	rM	9605168	11977871	21.45%	14.445%
2	6.786	299	301	308	rM	45268045	55832034	100.00%	67.334%
3	6.882	308	310	326	rM	12175857	15108446	27.06%	18.221%

Supplementary Figure 35. Selectivity was further determined by GCMS due to the limitation of NMR. **2′x**:**3′x** = >99:1 by crude GC-MS. The retention times of diastereomers of **3′x** are 6.467 and 6.627 min, which were not detected in the reaction mixture.

B) Inseparable products

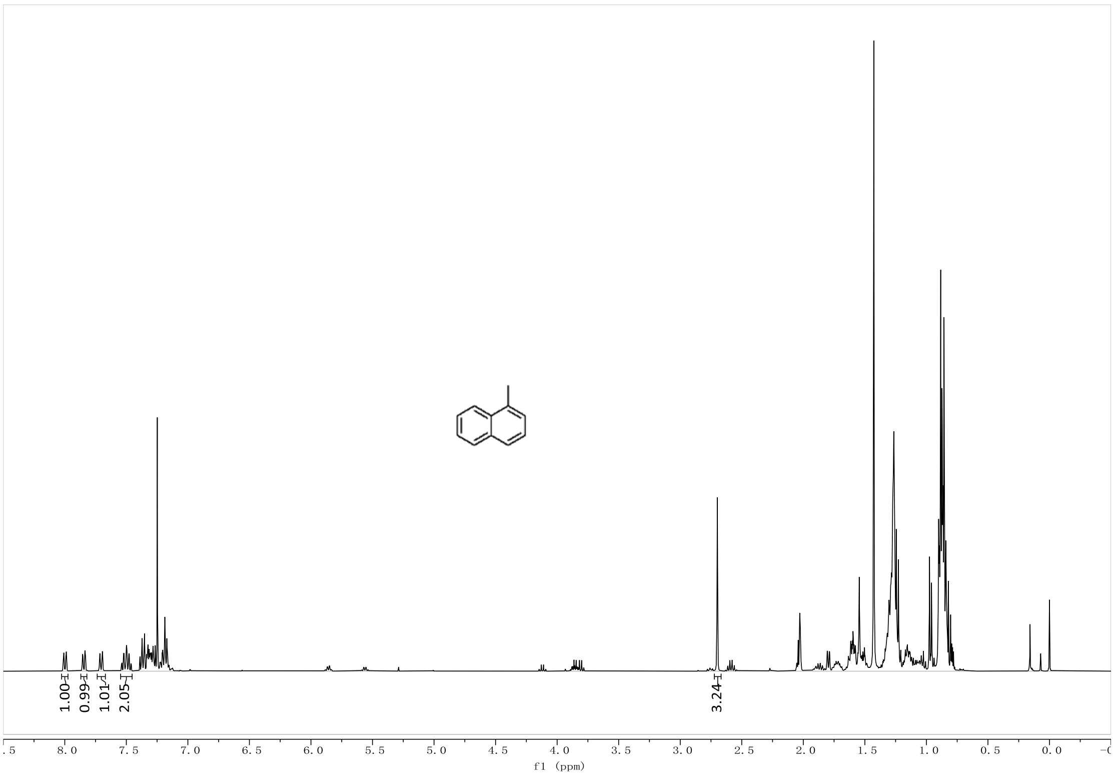

Supplementary Figure 36. Inseparable ^{1}H NMR| Fig.4**IV**, **1o** → **2o**, 91% yield. Product characteristic peaks are in line with commercially available products.

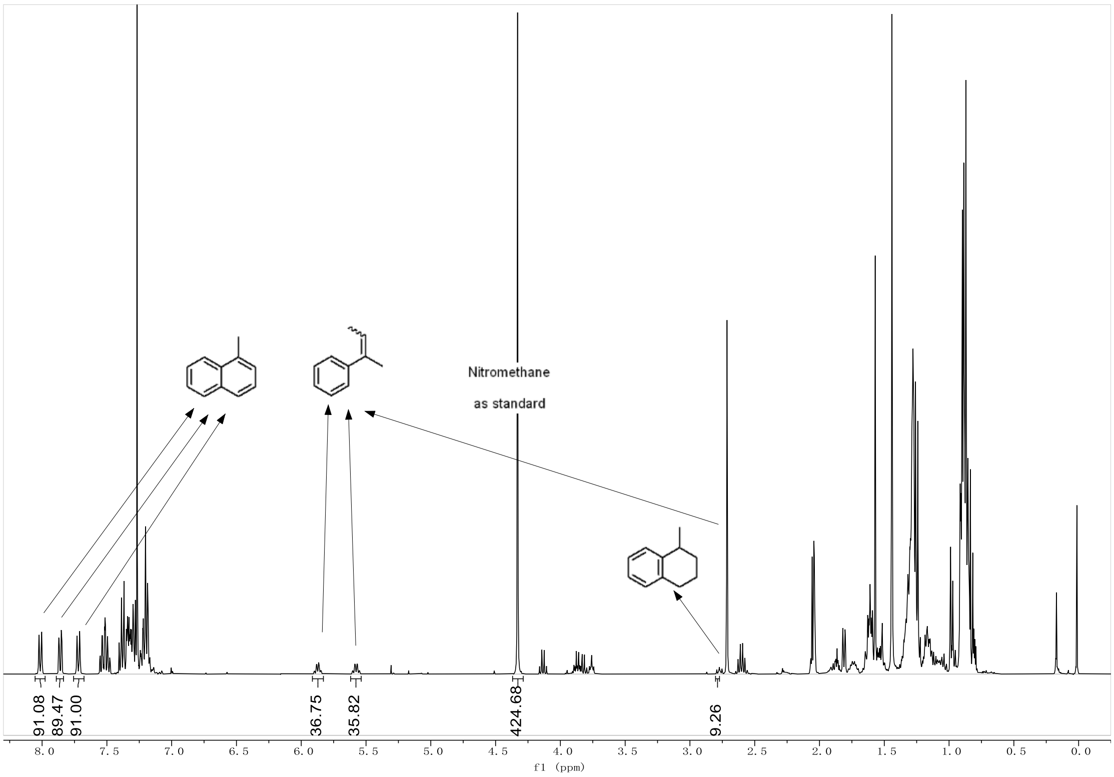

Supplementary Figure 37. 2o:**3o** = 96:4 by crude ^{1}H NMR

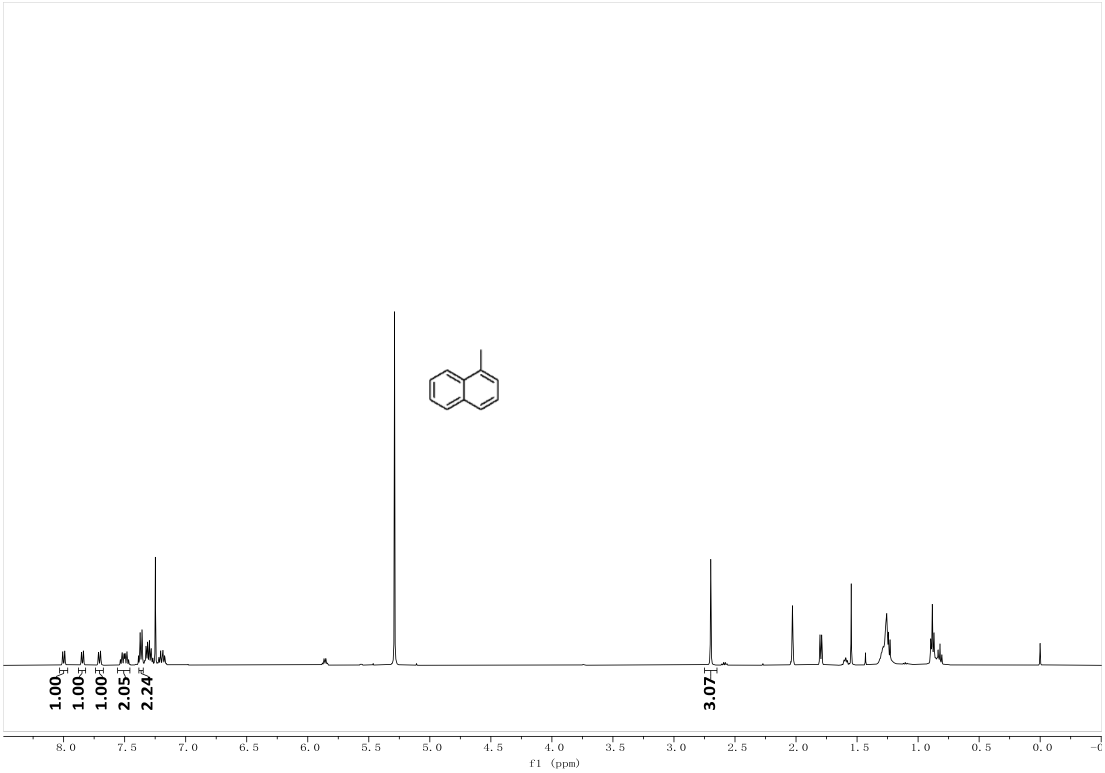

Supplementary Figure 38. Inseparable ^{1}H NMR| Fig.5**II**, **1′j → 2′j** (**2o**), 91% yield. Product characteristic peaks are in line with commercially available products.

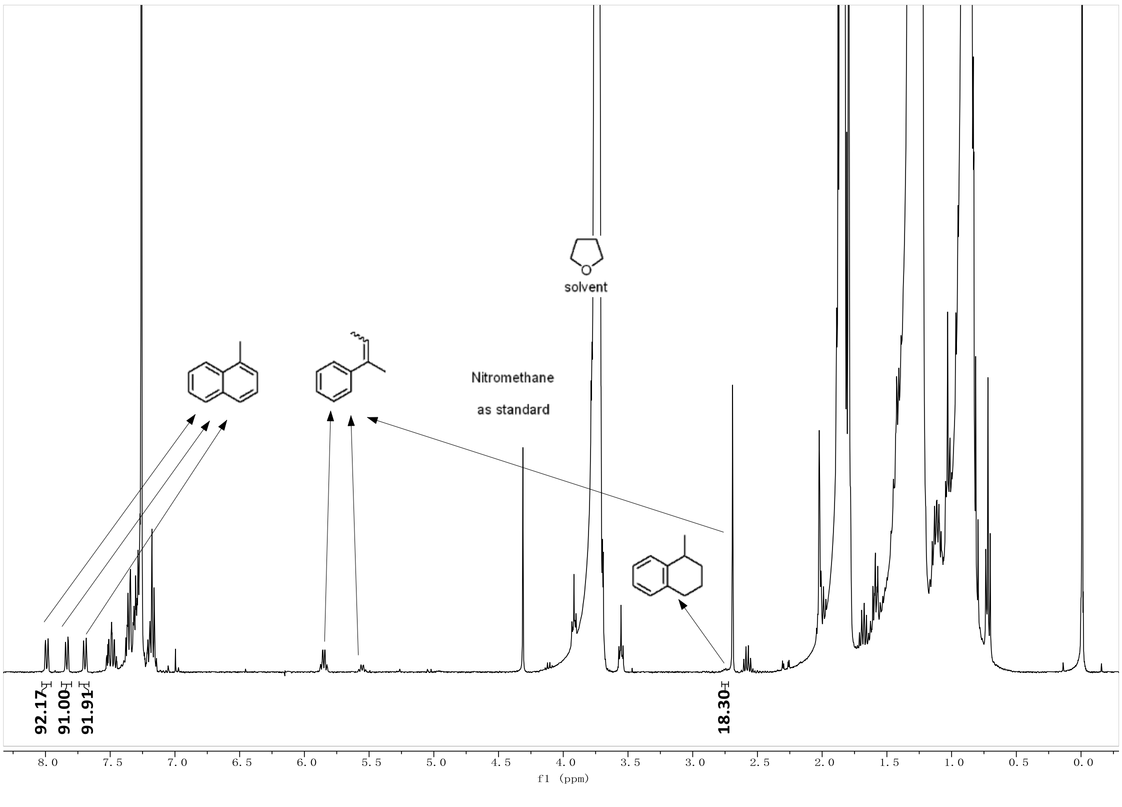

Supplementary Figure 39. 2′j:**3′j** = 91:9 by crude ^{1}H NMR.

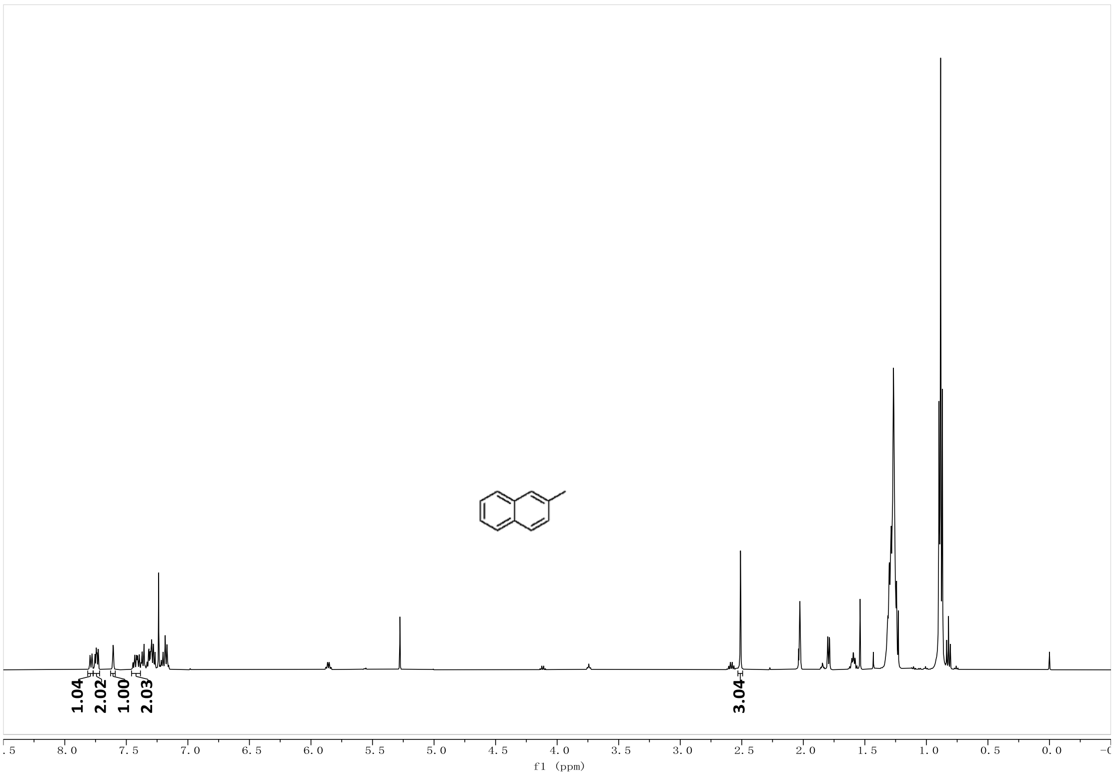

Supplementary Figure 40. Inseparable ^{1}H NMR| Fig.5**II**, **1′k** → **2′k**, 93% yield. Product characteristic peaks are in line with commercially available products.

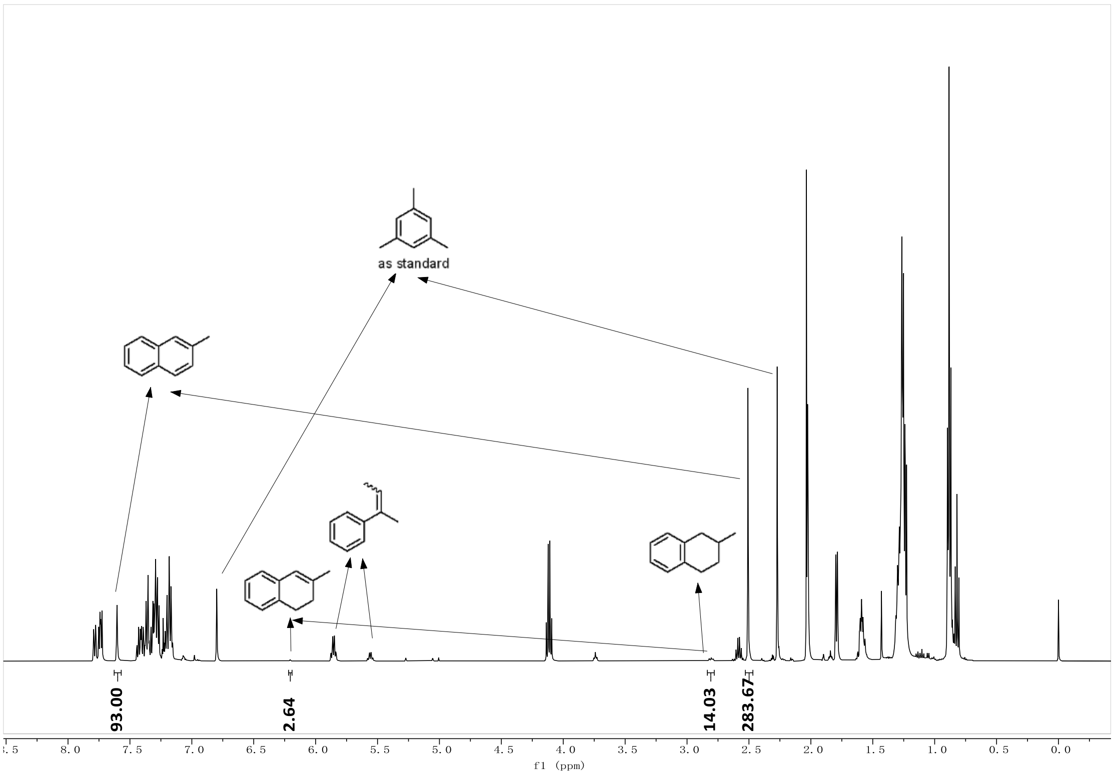

Supplementary Figure 41. 2′k:**3′k** = >95:5 by crude ^{1}H NMR.

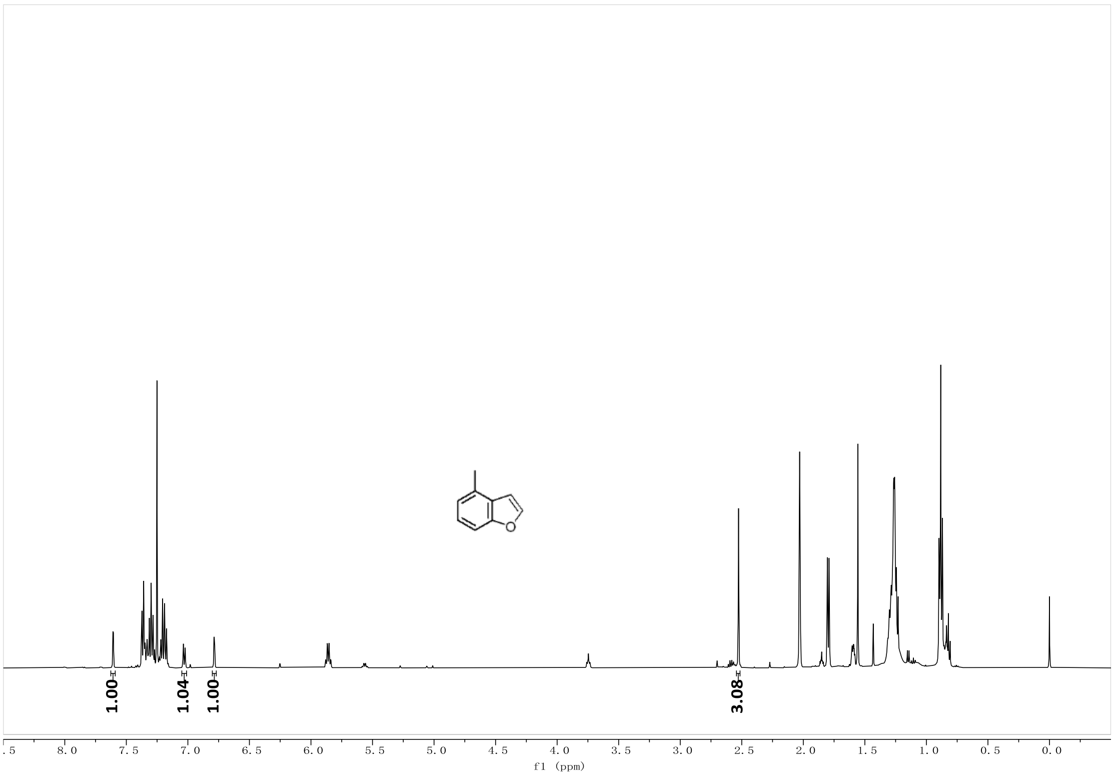

Supplementary Figure 42. Inseparable ^{1}H NMR| Fig.5**II**, **1′l** → **2′l**, 84% yield. Product characteristic peaks are in line with the literature[49].

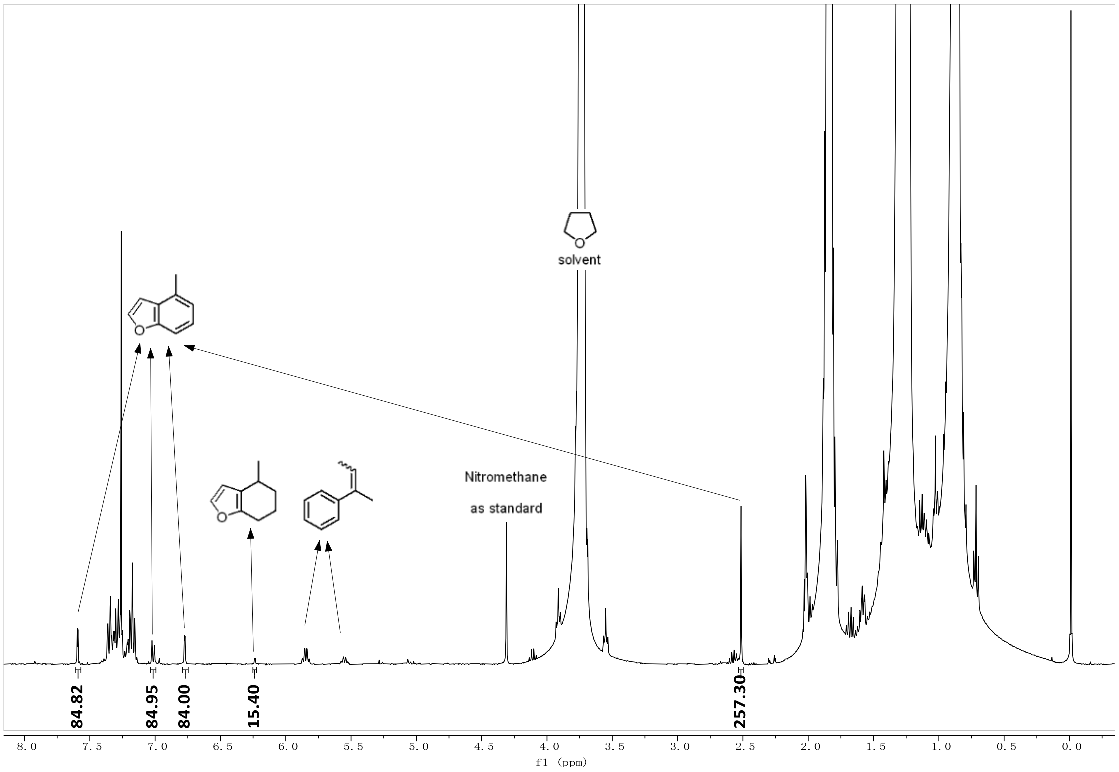

Supplementary Figure 43. 2′l:**3′l** = 84:16 by crude ^{1}H NMR

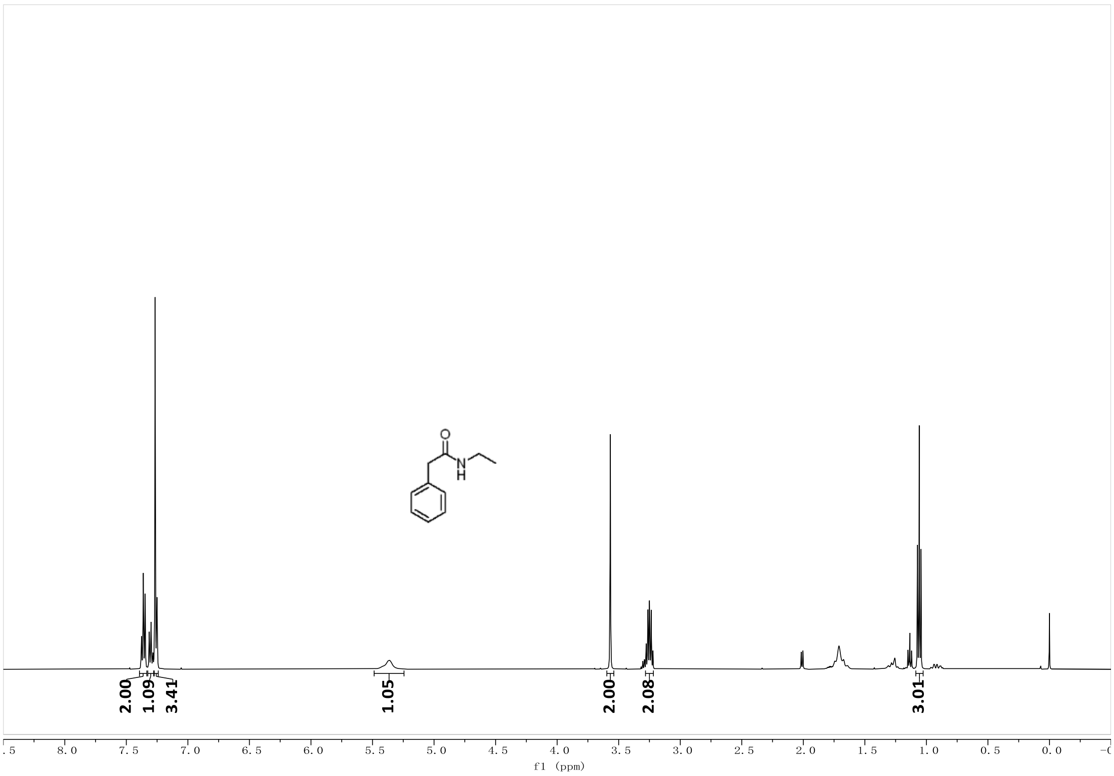

Supplementary Figure 44. Inseparable ^{1}H NMR| Fig.5**III**, **1′n→ 2′n,** 84% yield. Product characteristic peaks are in line with the literature[50].

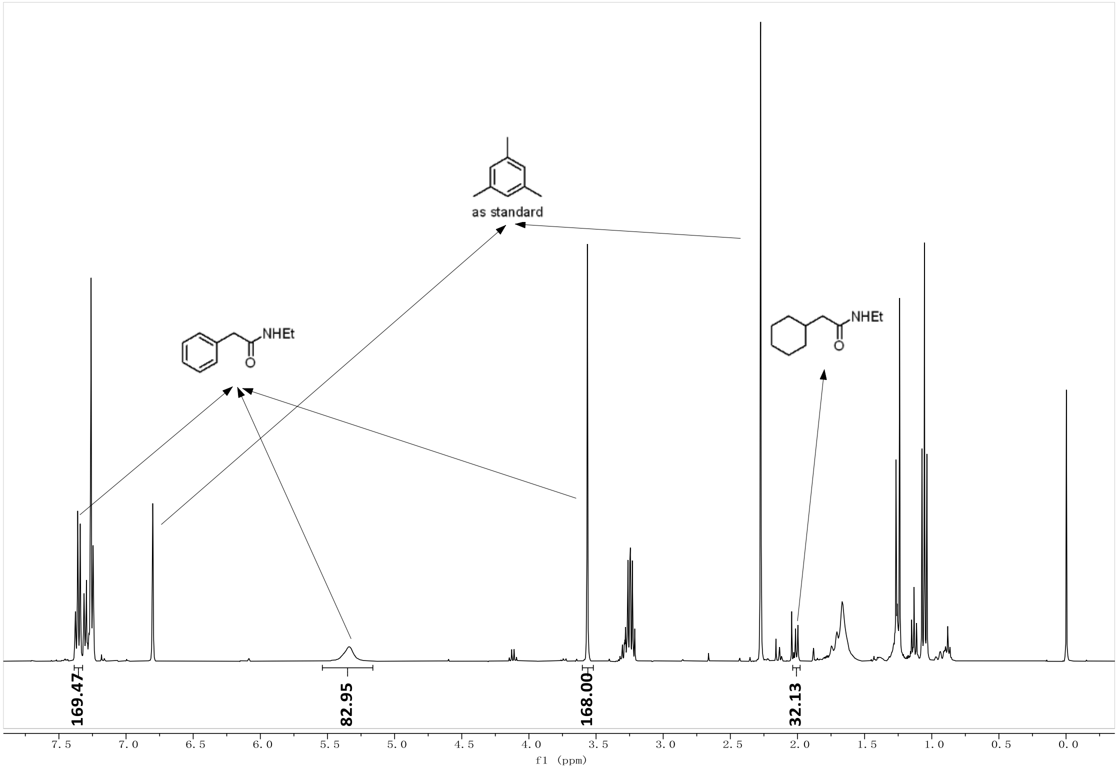

Supplementary Figure 45. 2′n:**3′n** = 84:16 by crude ^{1}H NMR

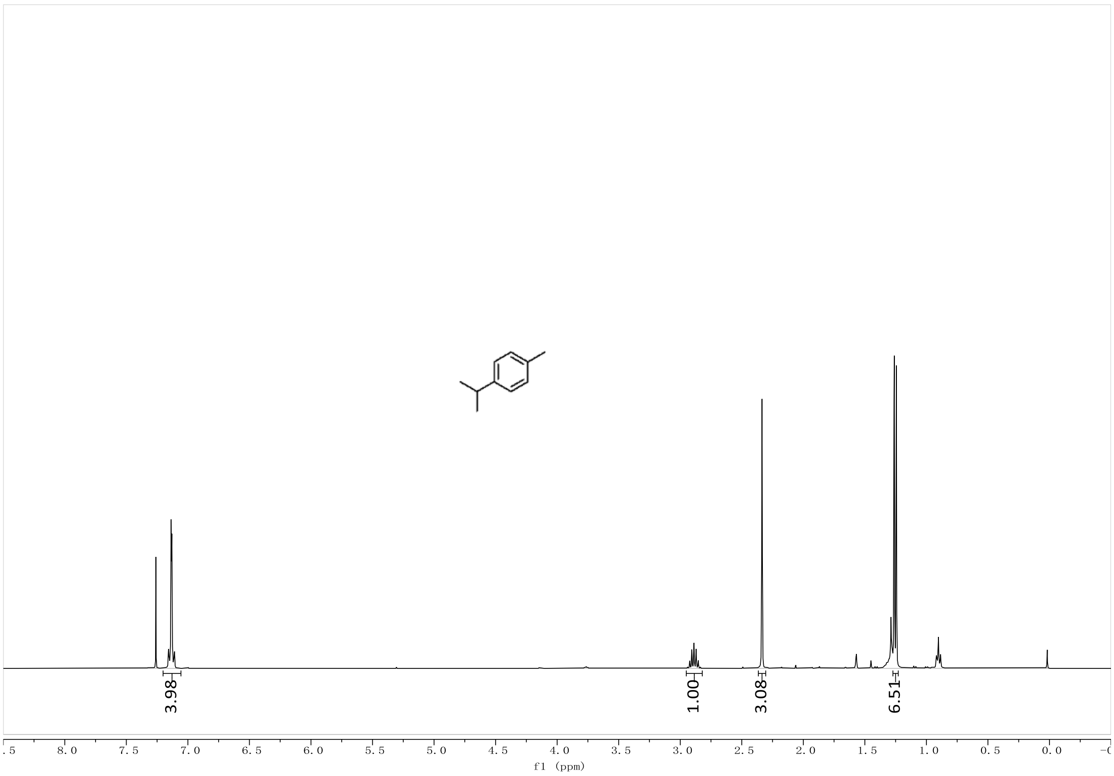

Supplementary Figure 46. Inseparable ^{1}H NMR| Fig.4**III**, **1l** → **2l**, 95% yield. Product characteristic peaks are in line with commercially available products.

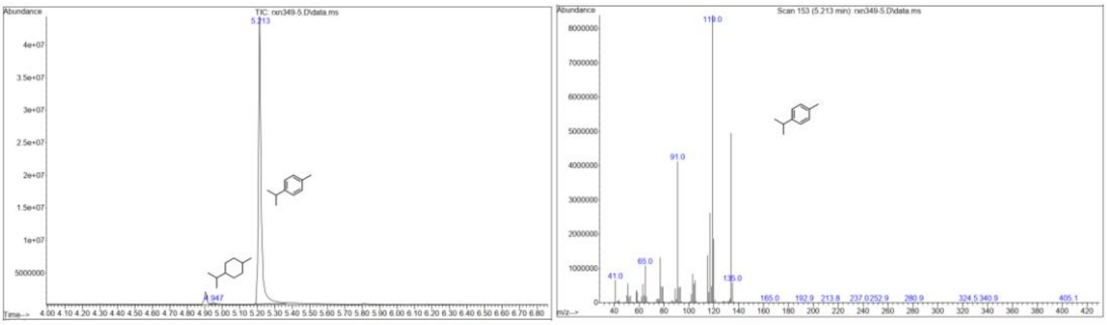

File :D:\Data-1\CWH\2020\rxn349-5.D
Operator :
Acquired : 27 Nov 2020 02:56 using AcqMethod 20151029-ms 1000 new program.M
Instrument : 5977msd
Sample Name: rxn349-5
Misc Info :
Vial Number: 2

peak #	R.T. min	first scan	max scan	last scan	PK TY	peak height	corr. area	corr. % max.	% of total
1	4.947	126	128	130	rM	250675	328116	0.69%	0.688%
2	5.213	151	153	167	rM	44431997	47360968	100.00%	99.312%

Supplementary Figure 47. The pure hydrocarbon product was inseparable from the reduction product and the selectivity was further determined by GCMS. **2l**:**3l** = >99:1 by crude GC-MS. The products **2l** and **3l** are inseparable by column chromatography. Uncalibrated GCMS was used to determine the ratio of this particular hydrocarbon according to the report in the literature[51].

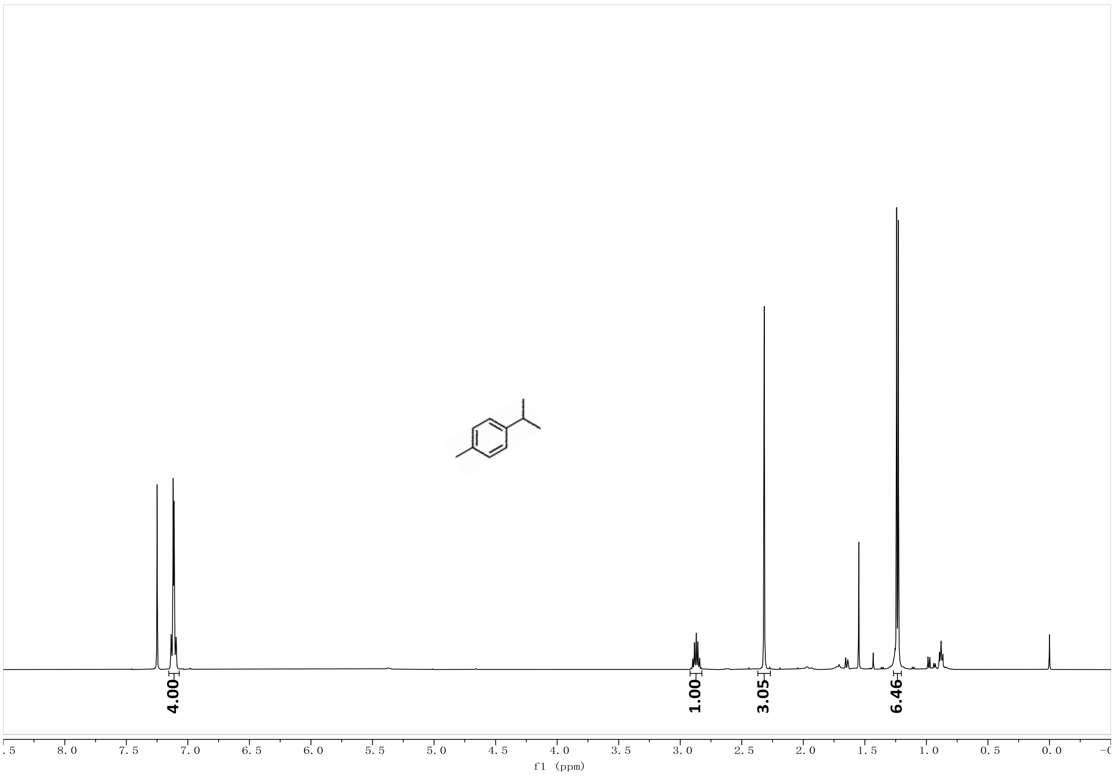

Supplementary Figure 48. Inseparable ^{1}H NMR| Fig.4**III**, **1m** → **2l**, 97% yield. Product characteristic peaks are in line with commercially available products.

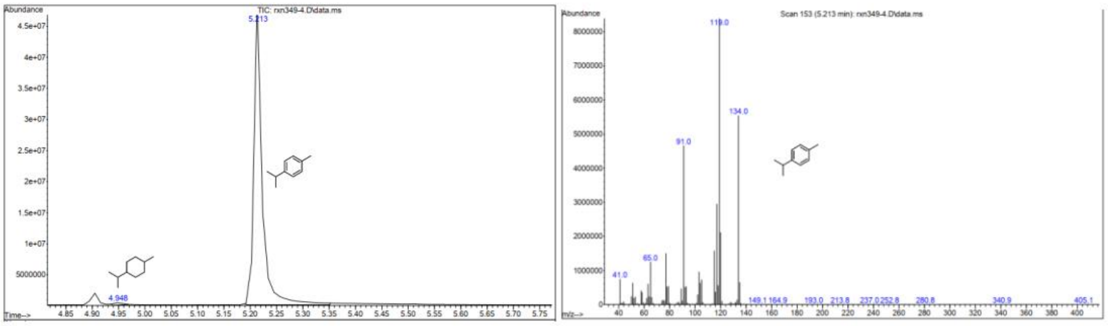


```
File       :D:\Data-1\CWH\2020\rxn349-4.D
Operator   :
Acquired   : 27 Nov 2020  02:23     using AcqMethod 20151029-ms 1000 new program.M
Instrument :    5977msd
Sample Name: rxn349-4
Misc Info  :
Vial Number: 1
```

peak #	R.T. min	first scan	max scan	last scan	PK TY	peak height	corr. area	corr. % max.	% of total
1	4.948	127	128	130	rM	383915	428594	0.81%	0.802%
2	5.213	151	153	166	rM	49789297	53033475	100.00%	99.198%

Supplementary Figure 49. The pure hydrocarbon product was inseparable from the reduction product and the selectivity was further determined by GCMS. **2l**:**3l** = 99:1 by crude GC-MS. The products **2l** and **3l** are inseparable by column chromatography. Uncalibrated GCMS was used to determine the ratio of this particular hydrocarbon according to the report in the literature[51].

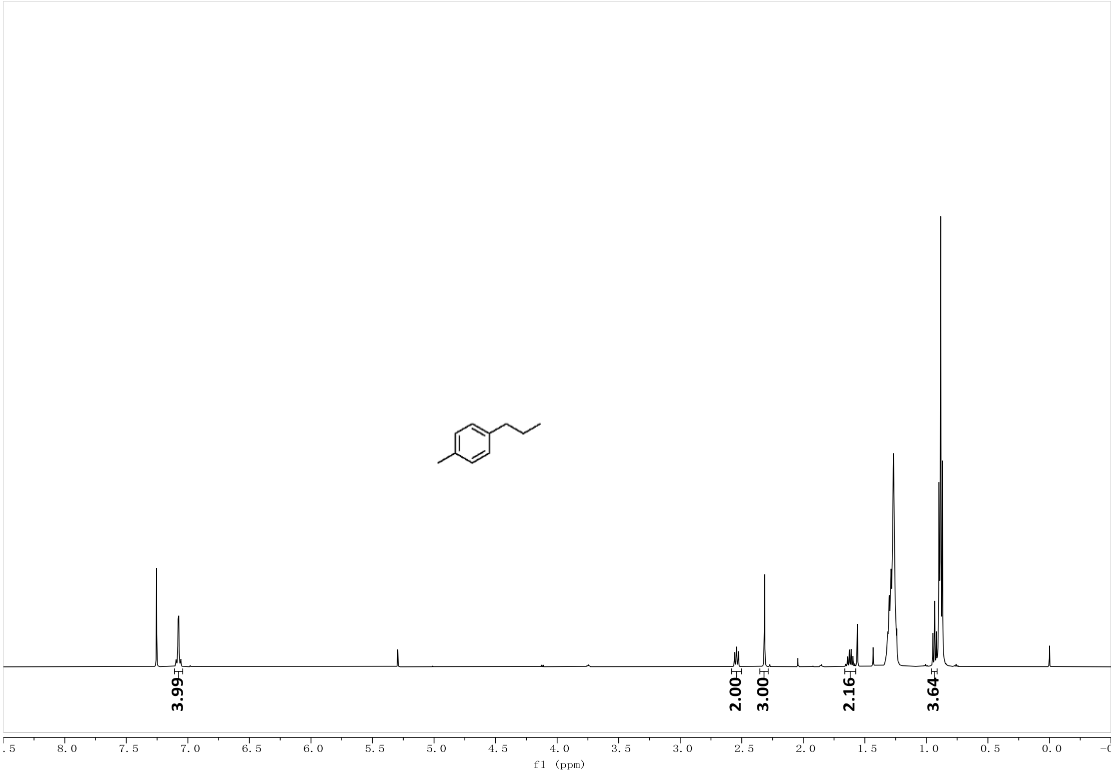

Supplementary Figure 50. Inseparable ^{1}H NMR| Fig.5**I**, **1′a** → **2′a**, 92% yield. Product characteristic peaks are in line with commercially available products.

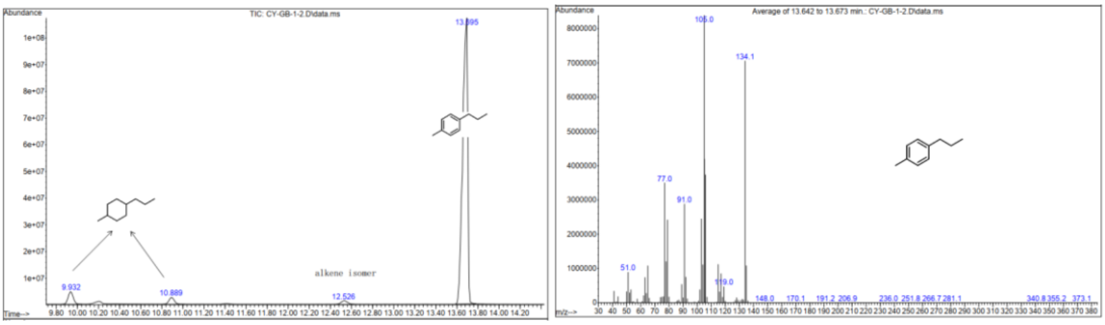

File :D:\Data-1\ChenY\2020\11\26\CY-GB-1-2.D
Operator :
Acquired : 28 Nov 2020 15:33 using AcqMethod C8.M
Instrument : 5977msd
Sample Name: CY-GB-1-2
Misc Info :
Vial Number: 12

peak #	R.T. min	first scan	max scan	last scan	PK TY	peak height	corr. area	corr. % max.	% of total
1	9.932	590	597	607	rM	4697676	16471647	4.61%	4.235%
2	10.889	680	687	699	rM	2532943	9438609	2.64%	2.427%
3	12.526	835	841	850	rM	1334067	5998268	1.68%	1.542%
4	13.695	941	951	959	rM 2	107038530	357008035	100.00%	91.796%

Supplementary Figure 51. The pure hydrocarbon product was inseparable from the reduction product and the selectivity was further determined by GCMS. Linear fit calibration equations were applied, and the response factors of the product **2′a** and **3′a** were determined.

2′a:**3′a** = 93:7 (non-calibrated); 98:2 (calibrated).

The calibration equations of peak area vs conc are as follow:

2′a: **A**=8.68×10^{7}**c**-1.54×10^{6}; **3′a:** **A**=3.70×10^{8}**c**-1.00×10^{7}

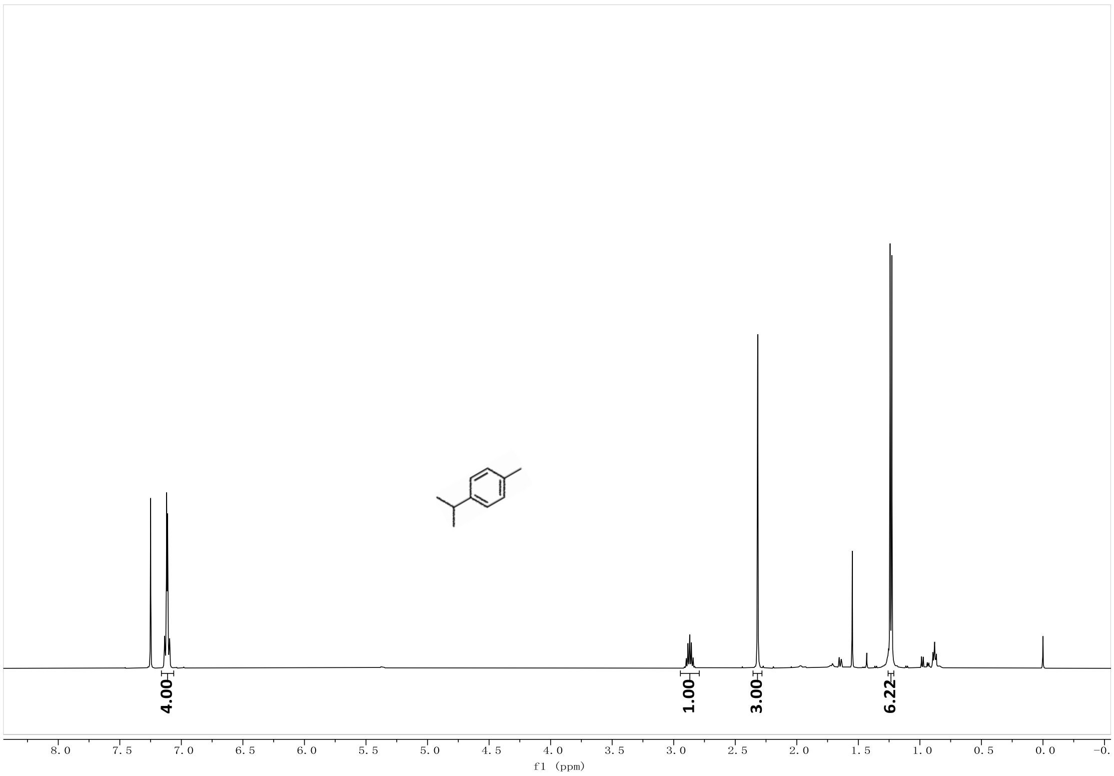

Supplementary Figure 52. Inseparable ^{1}H NMR| Fig.5**l**, **1′g** → **2l**, 91% yield. Product characteristic peaks are in line with commercially available products.

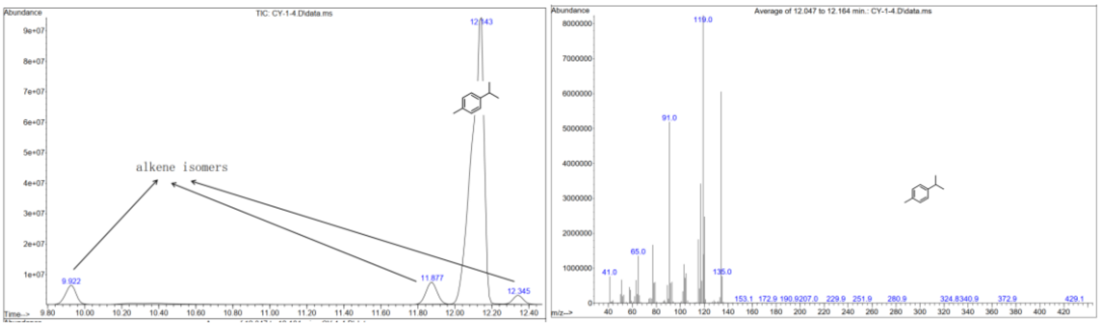

File :D:\Data-1\ChenY\2020\11\16\CY-1-4.D
Operator :
Acquired : 17 Nov 2020 20:42 using AcqMethod C8.M
Instrument : 5977msd
Sample Name: CY-1-4
Misc Info :
Vial Number: 47

peak #	R.T. min	first scan	max scan	last scan	PK TY	peak height	corr. area	corr. % max.	% of total
1	9.922	589	596	604	rM 2	6197174	24853814	5.64%	4.897%
2	11.877	772	780	788	rM	7245051	30498563	6.92%	6.009%
3	12.143	789	805	815	rM 2	94193144	440641307	100.00%	86.819%
4	12.345	817	824	832	rM	2918272	11546777	2.62%	2.275%

Supplementary Figure 53. The pure hydrocarbon product was inseparable from the reduction product and the selectivity was further determined by GCMS. **2l**:**3l** = >99:1 by crude GC-MS. No reduction product was detected in the reaction mixture.

C) Volatile products

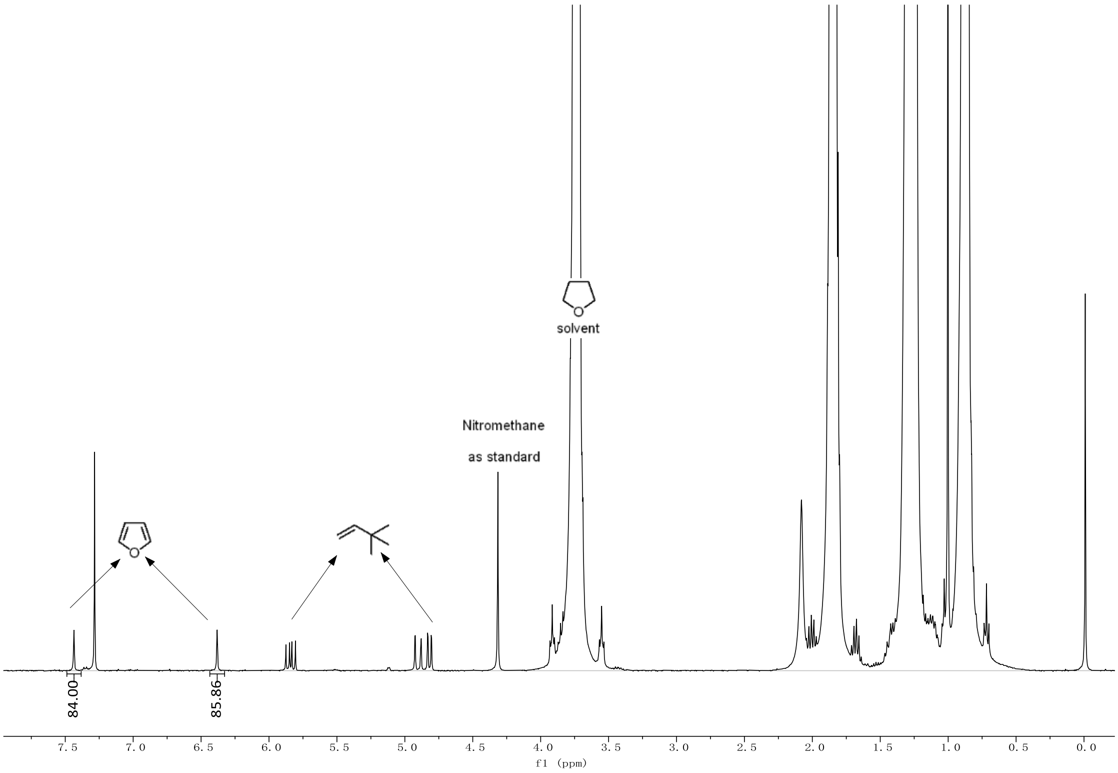

Supplementary Figure 54. Crude ^{1}H NMR| Fig.4**II**, **1h → 2h,** 42% yield. Product characteristic peaks are in line with commercially available products.

No GC-MS spectrum was provided due to the low boiling point of desired product.

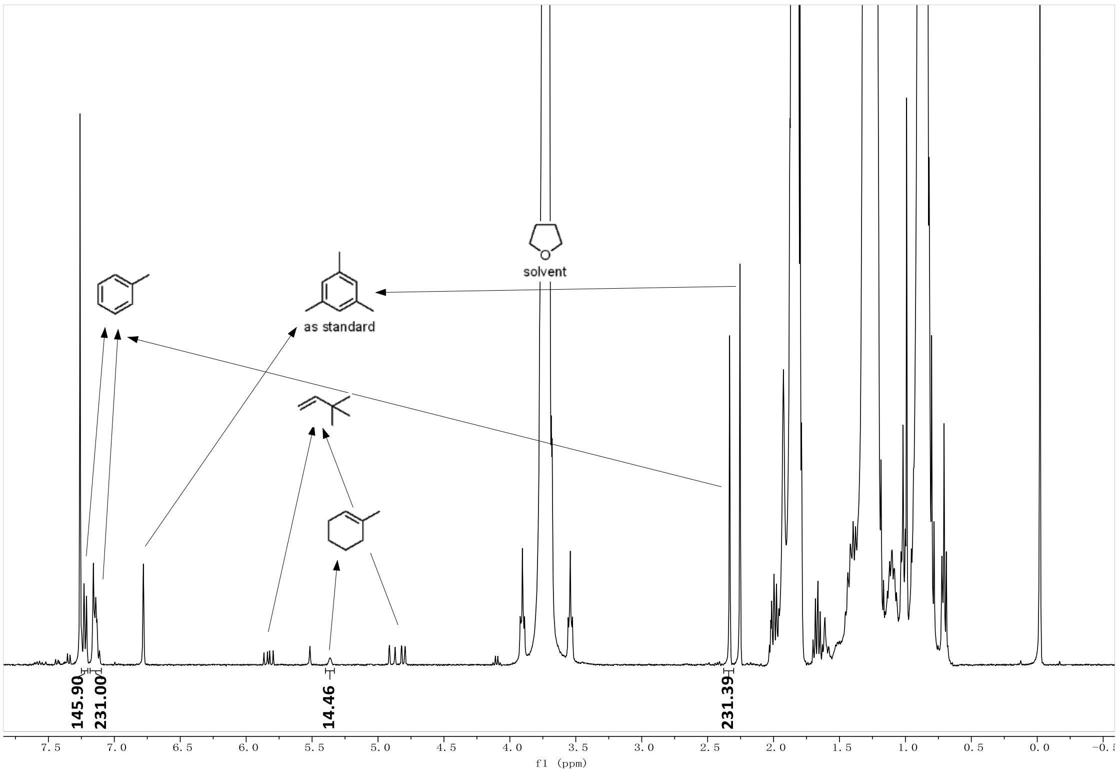

Supplementary Figure 55. Crude ^{1}H NMR| Fig.4**I**, **1d** → **2d,** 77% yield (>95% conversion). Product characteristic peaks are in line with commercially available products.

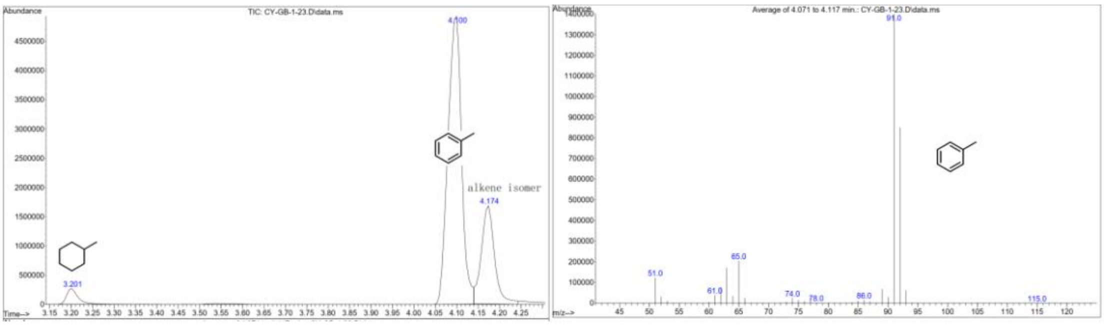

File :D:\Data-1\ChenY\2022\05\28\CY-GB-1-23.D
Operator :
Acquired : 31 May 2022 17:20 using AcqMethod slow T increasingt.M
Instrument : 5977msd
Sample Name: CY-GB-1-23
Misc Info :
Vial Number: 32

peak #	R.T. min	first scan	max scan	last scan	PK TY	peak height	corr. area	corr. % max.	% of total
1	3.201	15	20	34	rM	269097	507197	4.64%	3.375%
2	4.100	168	177	184	rM	4926757	10923444	100.00%	72.681%
3	4.174	184	190	202	rM	1681832	3598636	32.94%	23.944%

Supplementary Figure 56. The pure hydrocarbon product was inseparable from the reduction product and the selectivity was further determined by GCMS. Linear fit calibration equations were applied, and the response factors of the product **2d** and **3d** were determined.

2d: **3d** = 96:4 (non-calibrated); 99:1 (calibrated).

The calibration equations of peak area vs conc are as follow:

2d: A=1.31×10^{8}**c**-6.92×10^{4}; **3d: A**=6.02×10^{8}**c**-1.66×10^{5}

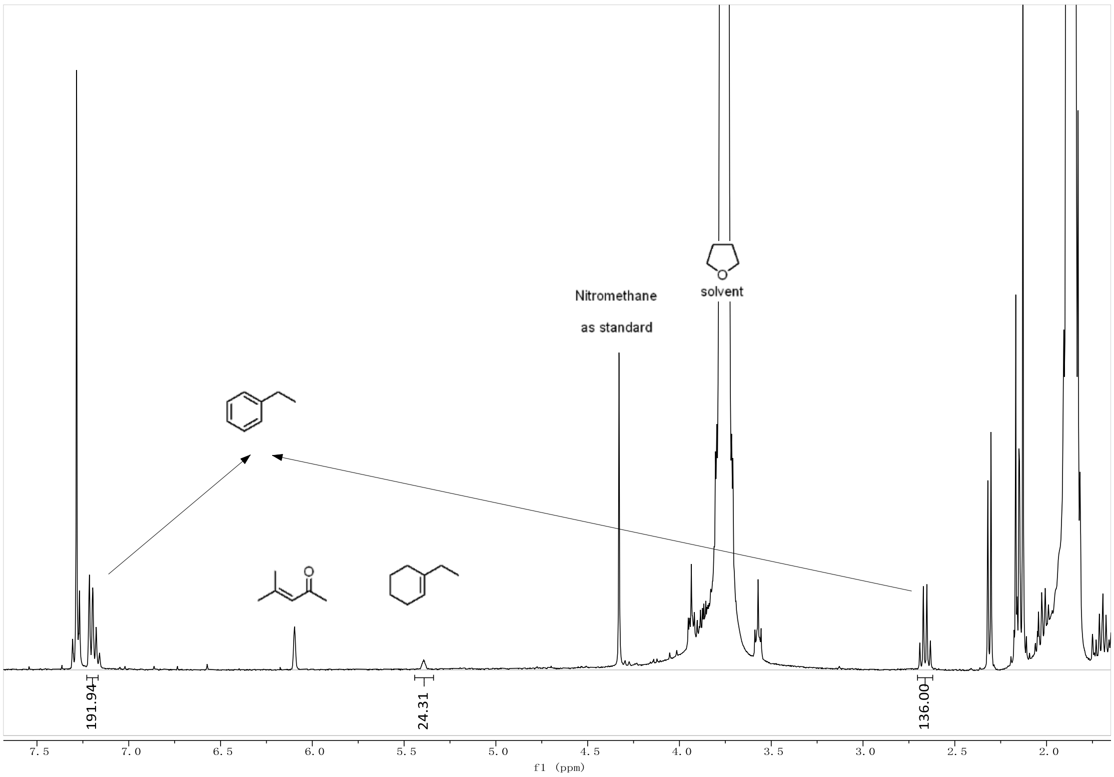

Supplementary Figure 57. Crude ^{1}H NMR| Fig.4**V**, **1p** → **2p,** 68% yield (75% conversion). Product characteristic peaks are in line with commercially available products.

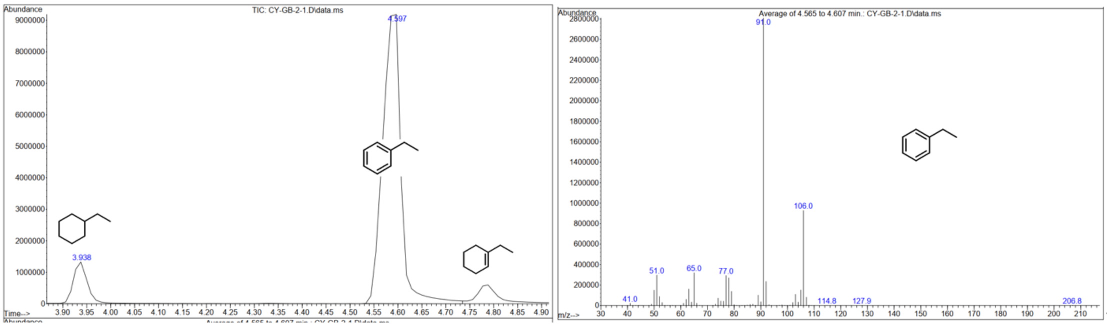

File :D:\Data-1\ChenY\2022\06\01\CY-GB-2-1.D
Operator :
Acquired : 02 Jun 2022 08:38 using AcqMethod C8.M
Instrument : 5977msd
Sample Name: CY-GB-2-1
Misc Info :
Vial Number: 29

peak #	R.T. min	first scan	max scan	last scan	PK TY	peak height	corr. area	corr. % max.	% of total
1	3.938	29	33	39	rM	1324068	2690951	10.97%	9.882%
2	4.597	89	95	108	rM	9180953	24539677	100.00%	90.118%

Supplementary Figure 58. The pure hydrocarbon product was inseparable from the reduction product and the selectivity was further determined by GCMS. Linear fit calibration equations were applied, and the response factors of the product **2p** and **3p** were determined.

2p: **3p** = 90:10 (non-calibrated); 89:11 (calibrated).

The calibration equations of peak area vs conc are as follow:

2p: A=2.81×10^{8}**c**-1.53×10^{6}; **3p: A**=2.76×10^{8}**c**-4.94×10^{5}

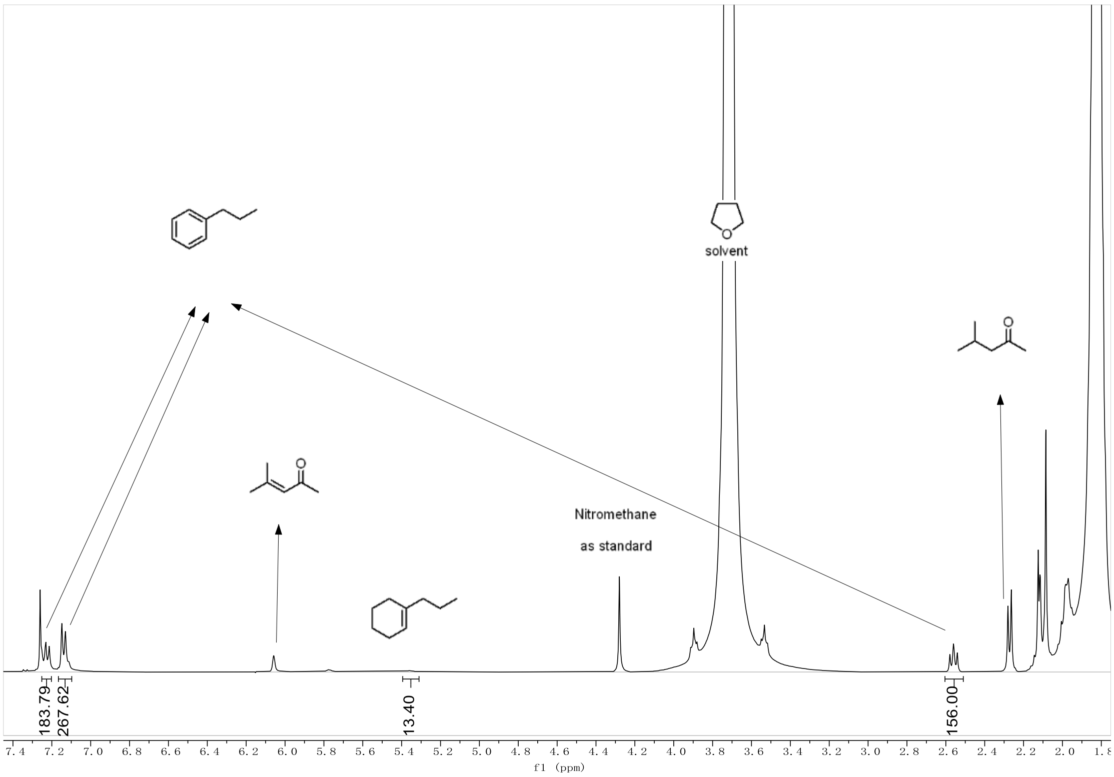

Supplementary Figure 59. Crude ^{1}H NMR| Fig.4**V**, **1q → 2q,** 78% yield. Product characteristic peaks are in line with commercially available products.

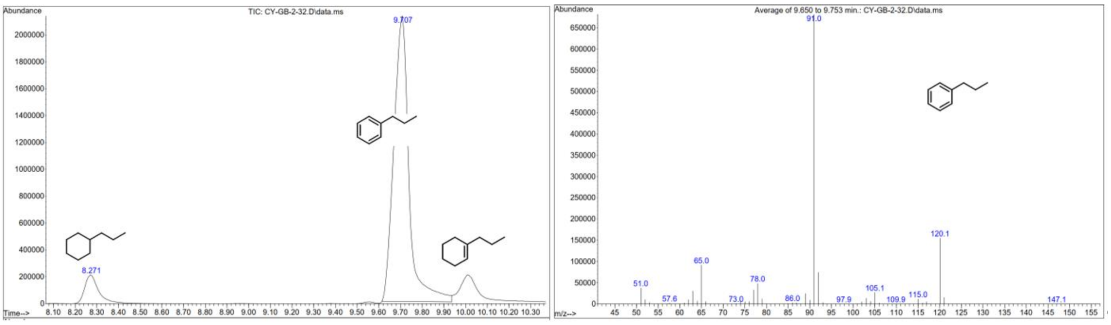

File :D:\Data-1\ChenY\2022\06\01\CY-GB-2-32.D
Operator :
Acquired : 02 Jun 2022 13:13 using AcqMethod CY-C6.M
Instrument : 5977msd
Sample Name: CY-GB-2-32
Misc Info :
Vial Number: 26

peak #	R.T. min	first scan	max scan	last scan	PK TY	peak height	corr. area	corr. % max.	% of total
1	8.271	893	906	946	rM 2	214612	959730	9.29%	8.496%
2	9.707	1141	1157	1197	rM	2126594	10336008	100.00%	91.504%

Supplementary Figure 60. The pure hydrocarbon product was inseparable from the reduction product and the selectivity was further determined by GCMS. Linear fit calibration equations were applied, and the response factors of the product **2q** and **3q** were determined.

2q: **3q** = 90:10 (non-calibrated); 89:11 (calibrated).

The calibration equations of peak area vs conc are as follow:

2q: A$=2.12\times10^{8}$**c**-6.45×10^{5}; **3q: A**$=1.40\times10^{8}$**c**$+3.63\times10^{4}$

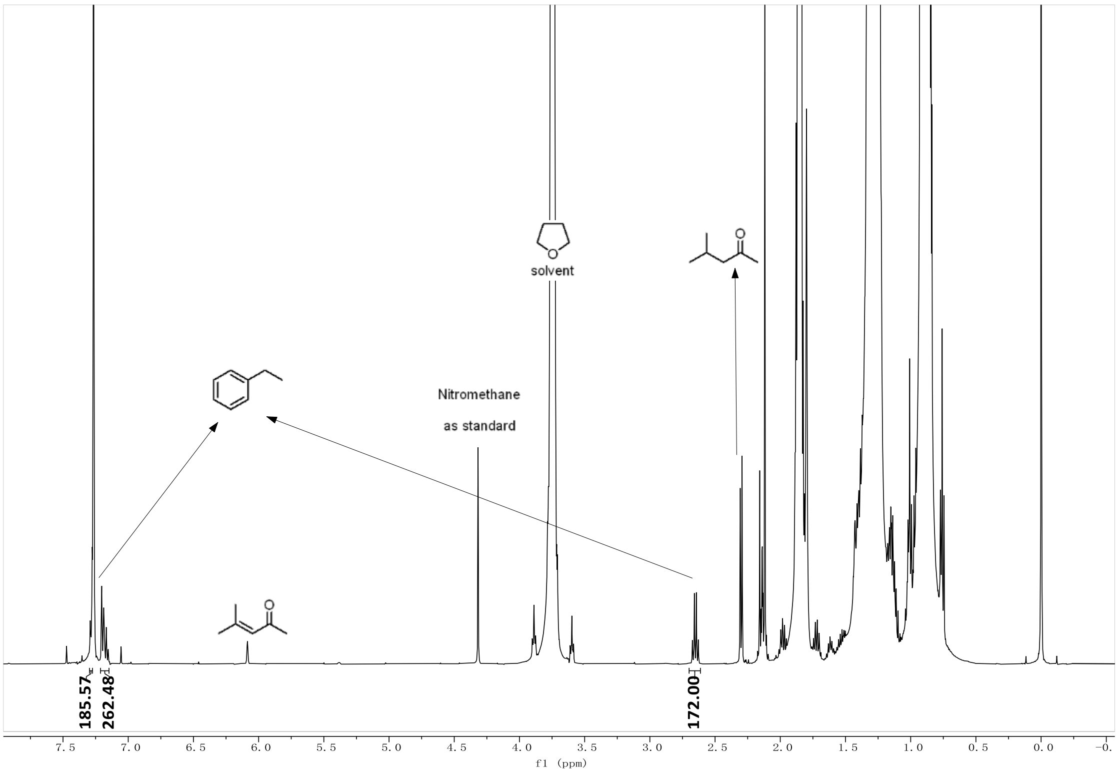

Supplementary Figure 61. Crude ^{1}H NMR| Fig.5**I**, **1′c → 2p,** 86% yield. Product characteristic peaks are in line with commercially available products.

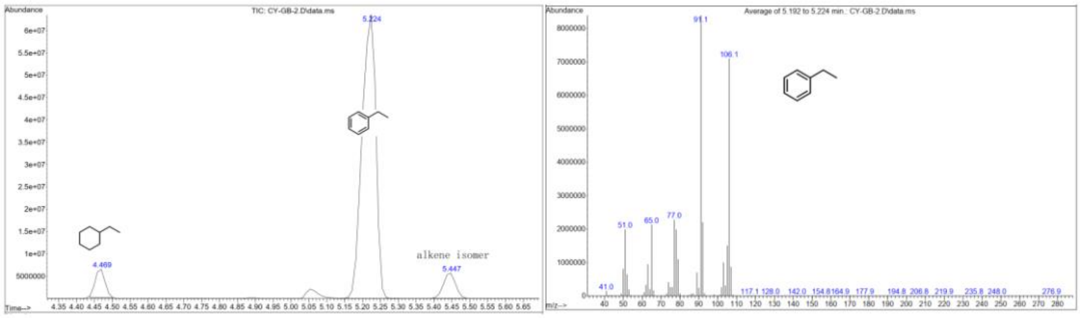

File :D:\Data-1\ChenY\2020\10\12\CY-GB-2.D
Operator :
Acquired : 16 Oct 2020 14:33 using AcqMethod C8.M
Instrument : 5977msd
Sample Name: CY-GB-2
Misc Info :
Vial Number: 28

peak #	R.T. min	first scan	max scan	last scan	PK TY	peak height	corr. area	corr. % max.	% of total
1	4.469	80	83	86	rM	6316409	12016692	7.01%	6.108%
2	5.224	146	154	161	rM 2	63512024	171392905	100.00%	87.120%
3	5.447	170	175	179	rM 2	5628692	13322599	7.77%	6.772%

Supplementary Figure 62. The pure hydrocarbon product was inseparable from the reduction product and the selectivity was further determined by GCMS. Linear fit calibration equations were applied, and the response factors of the product **2p** and **3p** were determined.

2p: **3p** = 94:6 (non-calibrated); 93:7 (calibrated).

The calibration equations of peak area vs conc are as follow:

2p: $\mathbf{A}=2.81\times10^{8}\mathbf{c}-1.53\times10^{6}$; **3p:** $\mathbf{A}=2.76\times10^{8}\mathbf{c}-4.94\times10^{5}$

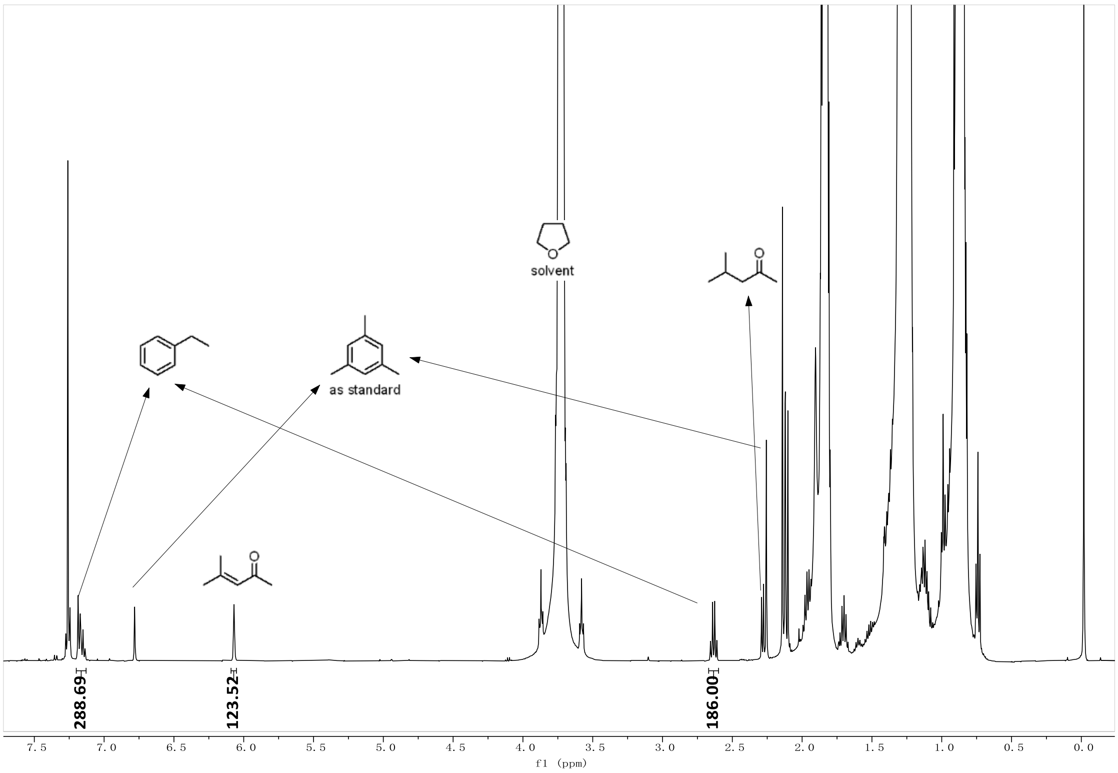

Supplementary Figure 63. Crude ^{1}H NMR| Fig.5**I**, **1′d → 2p,** 93% yield. Product characteristic peaks are in line with commercially available products.

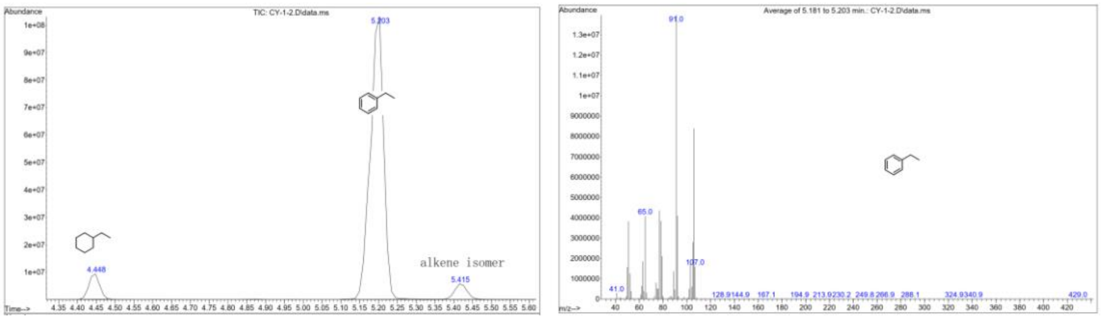

File :D:\Data-1\ChenY\2020\11\16\CY-1-2.D
Operator :
Acquired : 17 Nov 2020 19:43 using AcqMethod C8.M
Instrument : 5977msd
Sample Name: CY-1-2
Misc Info :
Vial Number: 45

peak #	R.T. min	first scan	max scan	last scan	PK TY	peak height	corr. area	corr. % max.	% of total
1	4.448	77	81	84	rM	9242355	18621173	7.33%	6.515%
2	5.203	145	152	159	rM 2	102907786	254088753	100.00%	88.902%
3	5.415	168	172	177	rM	5654357	13097988	5.15%	4.583%

Supplementary Figure 64. The pure hydrocarbon product was inseparable from the reduction product and the selectivity was further determined by GCMS. Linear fit calibration equations were applied, and the response factors of the product **2p** and **3p** were determined.

2p: **3p** = 93:7 (non-calibrated); 92:8 (calibrated).

The calibration equations of peak area vs conc are as follow:

2p: $\mathbf{A}=2.81\times10^{8}\mathbf{c}-1.53\times10^{6}$; **3p:** $\mathbf{A}=2.76\times10^{8}\mathbf{c}-4.94\times10^{5}$

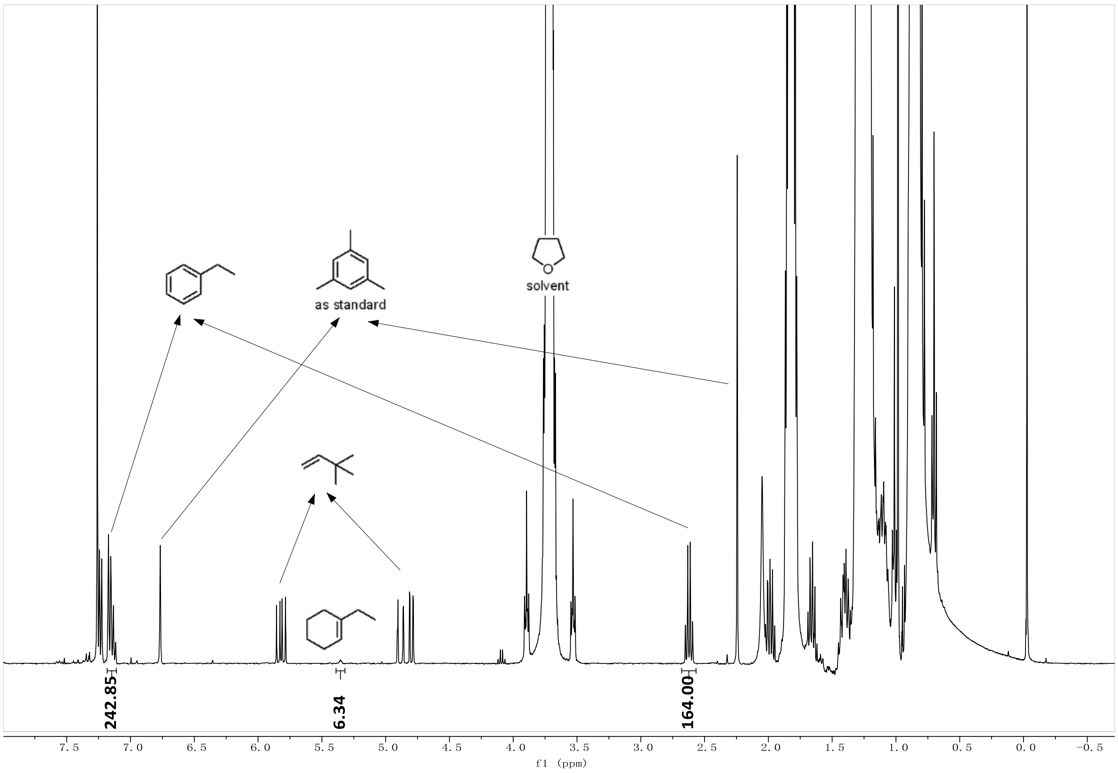

Supplementary Figure 65. Crude ^{1}H NMR| Fig.5**I**, **1′e → 2p,** 82% yield. Product characteristic peaks are in line with commercially available products.

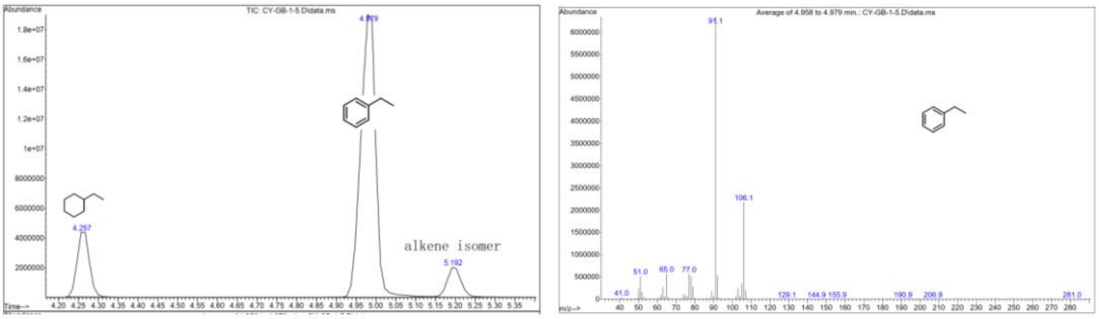

File :D:\Data-1\ChenY\2021\04\12\CY-GB-1-5.D
Operator :
Acquired : 14 Apr 2021 11:44 using AcqMethod C8.M
Instrument : 5977msd
Sample Name: CY-GB-1-5
Misc Info :
Vial Number: 50

peak #	R.T. min	first scan	max scan	last scan	PK TY	peak height	corr. area	corr. % max.	% of total
1	4.257	60	63	68	rM	4356741	9410485	18.66%	14.524%
2	4.979	125	131	144	rM	19018839	50433754	100.00%	77.837%
3	5.192	147	151	158	rM	2011848	4949654	9.81%	7.639%

Supplementary Figure 66. The pure hydrocarbon product was inseparable from the reduction product and the selectivity was further determined by GCMS. Linear fit calibration equations were applied, and the response factors of the product **2p** and **3p** were determined.

2p: **3p** = 84:16 (non-calibrated); 84:16 (calibrated).

The calibration equations of peak area vs conc are as follow:

2p: **A**=2.81×10^{8}**c**-1.53×10^{6}; **3p:** **A**=2.76×10^{8}**c**-4.94×10^{5}

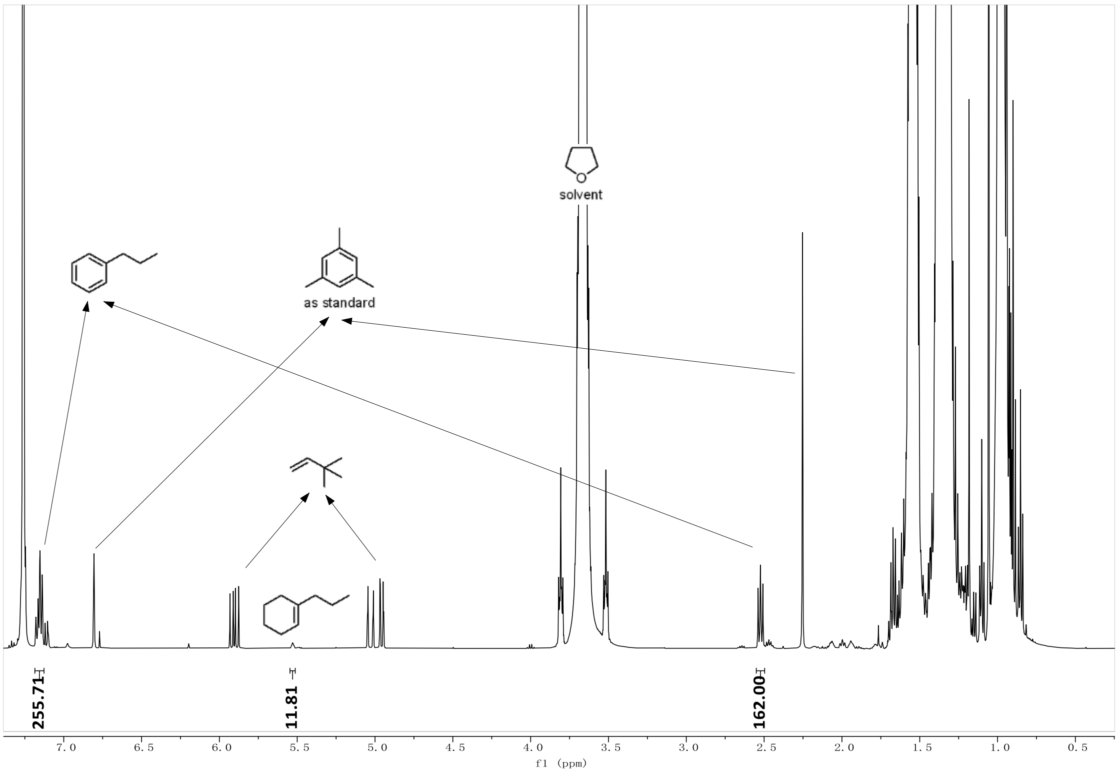

Supplementary Figure 67. Crude ^{1}H NMR| Fig.5**I**, **1′f → 2q,** 81% yield. Product characteristic peaks are in line with commercially available products.

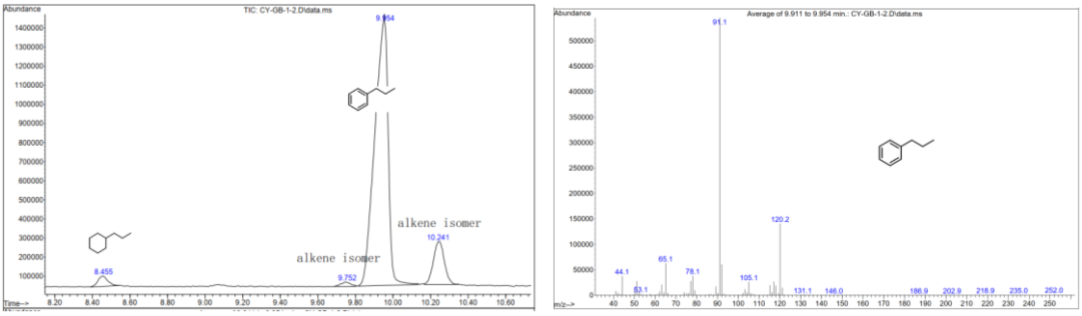


```
File        :D:\Data-1\ChenY\2021\12\09\CY-GB-1-2.D
Operator    :
Acquired    : 11 Dec 2021  19:24      using AcqMethod CY-C6.M
Instrument  :    5977msd
Sample Name: CY-GB-1-2
Misc Info   :
Vial Number: 22
```

peak #	R.T. min	first scan	max scan	last scan	PK TY	peak height	corr. area	corr. % max.	% of total
1	8.455	452	458	466	rM 3	54266	197380	2.83%	2.417%
2	9.752	574	580	585	rM 4	22321	84885	1.22%	1.039%
3	9.954	586	599	616	rM	1420654	6974537	100.00%	85.402%
4	10.241	619	626	636	rM	224926	909871	13.05%	11.141%

Supplementary Figure 68. The pure hydrocarbon product was inseparable from the reduction product and the selectivity was further determined by GCMS. Linear fit calibration equations were applied, and the response factors of the product **2q** and **3q** were determined.

2q: **3q** = 96:4 (non-calibrated); 97:3 (calibrated).

The calibration equations of peak area vs conc are as follow:

2q: $\mathbf{A}=2.12\times10^{8}\mathbf{c}-6.45\times10^{5}$; **3q:** $\mathbf{A}=1.40\times10^{8}\mathbf{c}+3.63\times10^{4}$

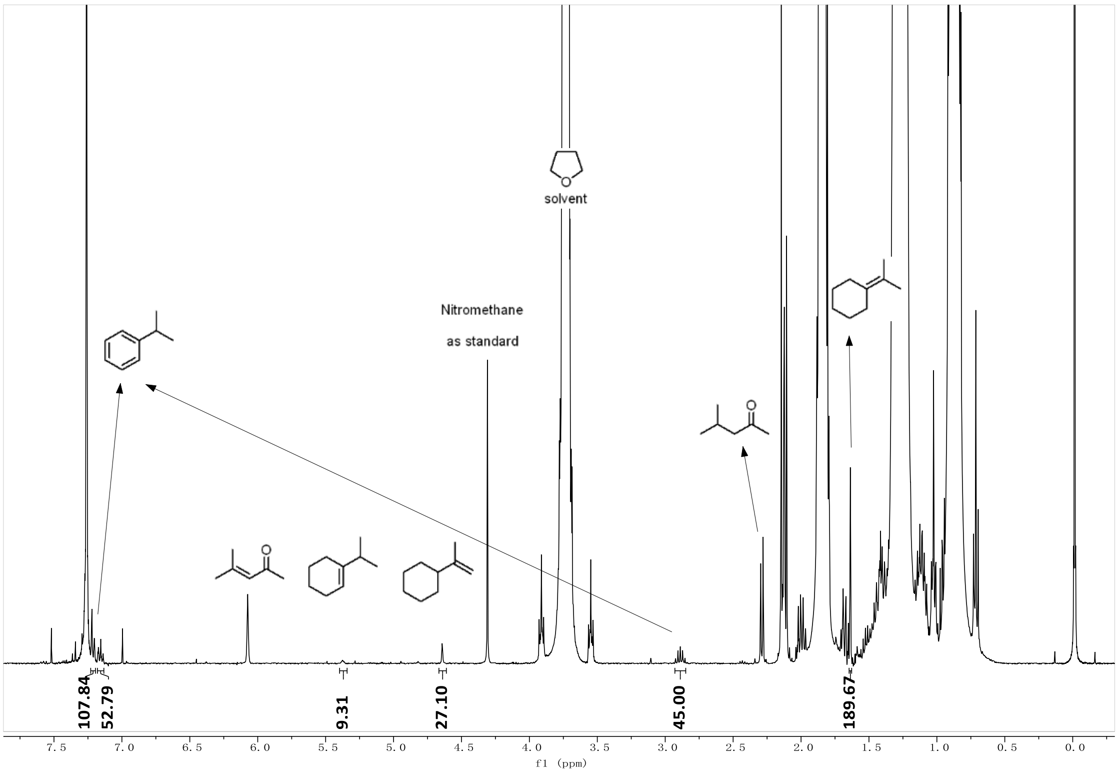

Supplementary Figure 69. Crude ^{1}H NMR| Fig.5**I**, **1′h → 2′h,** 45% yield (86% conversion). Product characteristic peaks are in line with commercially available products.

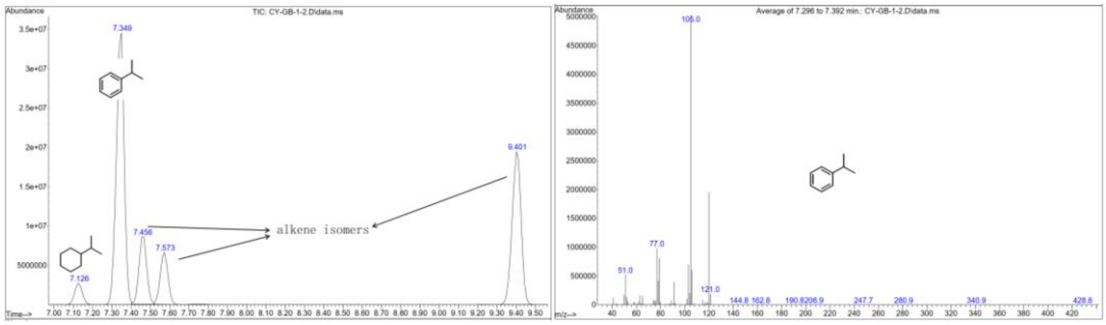

File :D:\Data-1\ChenY\2020\10\21\CY-GB-1-2.D
Operator :
Acquired : 22 Oct 2020 13:47 using AcqMethod C8.M
Instrument : 5977msd
Sample Name: CY-GB-1-2
Misc Info :
Vial Number: 48

peak #	R.T. min	first scan	max scan	last scan	PK TY	peak height	corr. area	corr. % max.	% of total
1	7.126	328	333	339	rM	2723744	7478773	7.57%	3.517%
2	7.349	346	354	359	rM 2	34549043	98757753	100.00%	46.449%
3	7.456	359	364	370	rM	8578681	24338655	24.64%	11.447%
4	7.573	370	375	381	rM	6623254	18319630	18.55%	8.616%
5	9.401	539	547	555	rM	19448587	63721824	64.52%	29.970%

Supplementary Figure 70. The pure hydrocarbon product was inseparable from the reduction product and the selectivity was further determined by GCMS. Conversion of the substrate (at 7.456 min) is 86%. Yield of the isomers (at 7.573 min and 9.401 min) is 38%. Linear fit calibration equations were applied, and the response factors of the product **2′h** and **3′h** were determined.

2′h: **3′h** = 92:8 (non-calibrated); 97:3 (calibrated).

The calibration equations of peak area vs conc are as follow:

2′h: **A**=2.12×10^8**c**-6.45×10^5; **3′h:** **A**=1.40×10^8**c**+3.63×10^4

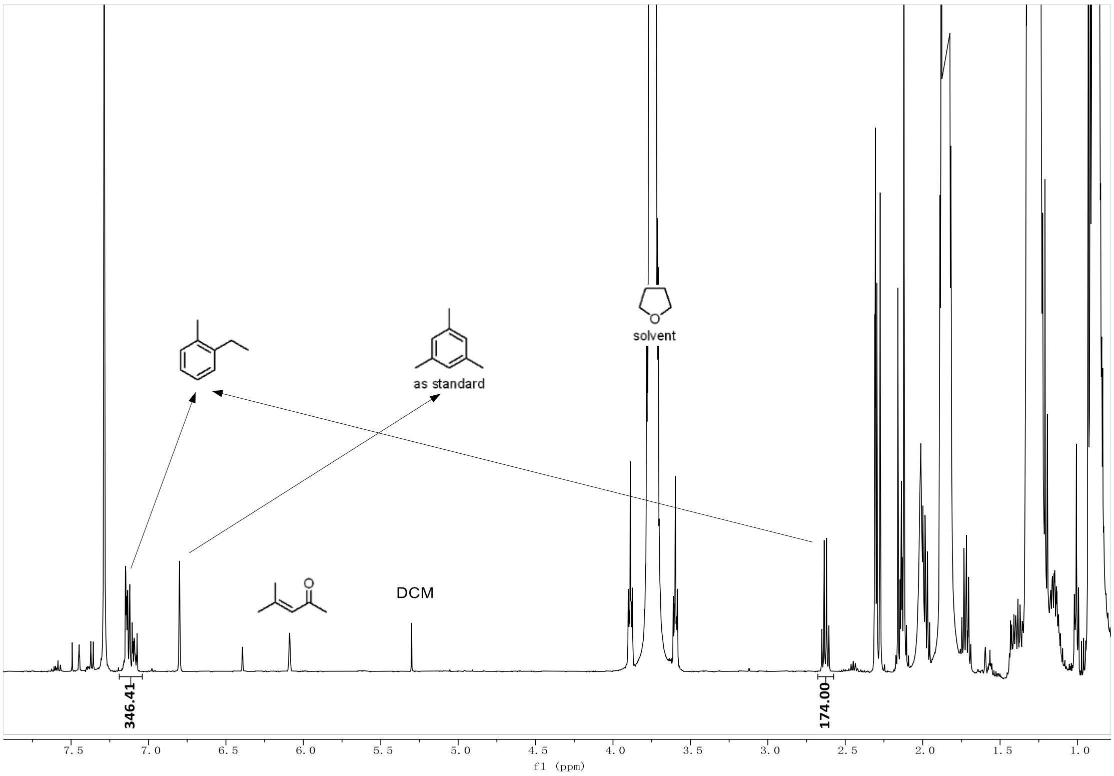

Supplementary Figure 71. Crude ^{1}H NMR| Fig.5**IV**, **1′w → 2′w,** 87% yield. Product characteristic peaks are in line with commercially available products.

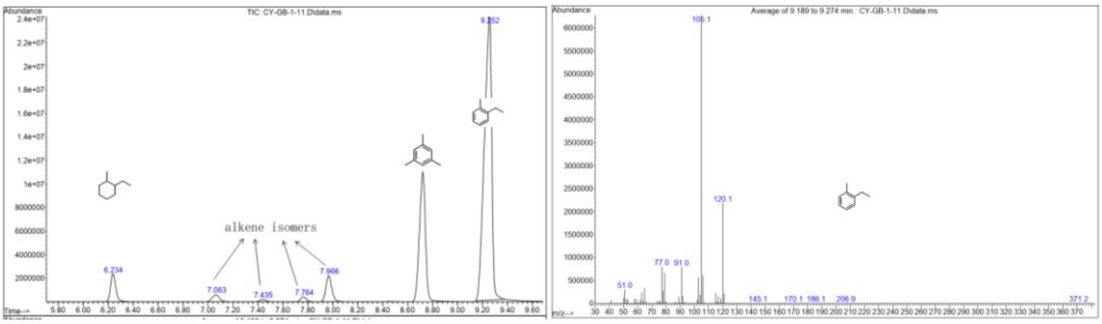

File   :D:\Data-1\ChenY\2021\07\06\CY-GB-1-11.D
Operator   :
Acquired   : 08 Jul 2021   22:10   using AcqMethod C8.M
Instrument :   5977msd
Sample Name: CY-GB-1-11
Misc Info   :
Vial Number: 46

peak #	R.T. min	first scan	max scan	last scan	PK TY	peak height	corr. area	corr. % max.	% of total
1	6.234	244	249	260	rM	2344807	6816561	6.86%	5.797%
2	7.063	321	327	335	rM 2	596292	2352322	2.37%	2.000%
3	7.435	358	362	368	rM	202642	639044	0.64%	0.543%
4	7.764	389	393	399	rM	425896	1288452	1.30%	1.096%
5	7.966	407	412	421	rM	2226799	7108818	7.15%	6.045%
6	9.252	523	533	545	rM 2	24104833	99384248	100.00%	84.518%

Supplementary Figure 72. The pure hydrocarbon product was inseparable from the reduction product and the selectivity was further determined by GCMS. **2′w**:**3′w** = 92:8 by crude GC-MS. The products **2′w** and **3′w** are inseparable by column chromatography. Uncalibrated GCMS was used to determine the ratio of this particular hydrocarbon according to the report in the literature[51].

Characterization data of isolated products:

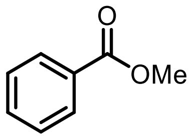

^{1}H NMR (400 MHz, $CDCl_3$) δ 8.07 – 8.02 (m, 2H), 7.59 – 7.53 (m, 1H), 7.47 – 7.41 (m, 2H), 3.92 (s, 3H). ^{13}C NMR (100 MHz, $CDCl_3$) δ 167.3, 133.0, 130.3, 129.7, 128.5, 52.3.

MS (m/z): M^+: calculated for $C_8H_8O_2$: 136.1; found 136.0.

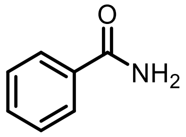

^{1}H NMR (500 MHz, $CDCl_3$) δ 7.82 (d, *J* = 8.2 Hz, 2H), 7.54 (t, *J* = 7.4 Hz, 1H), 7.46 (t, *J* = 7.6 Hz, 2H), 6.30 – 5.79 (m, 2H). ^{13}C NMR (125 MHz, $CDCl_3$) δ 169.6, 133.5, 132.2, 128.8, 127.5.

HRMS-ESI (m/z): $[M+H]^+$: calculated for C_7H_8NO 122.0600; found 122.0599.

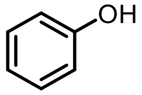

^{1}H NMR (400 MHz, $CDCl_3$) δ 7.29 – 7.20 (m, 2H), 6.93 (t, *J* = 7.4 Hz, 1H), 6.83 (d, *J* = 8.0 Hz, 2H), 5.10 (s, 1H). ^{13}C NMR (100 MHz, $CDCl_3$) δ 155.6, 129.8, 120.9, 115.4.

MS (m/z): M^+: calculated for C_6H_6O: 94.0; found 94.0.

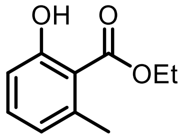

^{1}H NMR (400 MHz, $CDCl_3$) δ 11.37 (s, 1H), 7.26 (dd, J = 8.3, 7.5 Hz, 1H), 6.84 (d, *J* = 8.3 Hz, 1H), 6.72 (d, *J* = 7.5 Hz, 1H), 4.44 (q, *J* = 7.1 Hz, 2H), 2.56 (s, 3H), 1.44 (t, *J* = 7.1 Hz, 3H). ^{13}C NMR (100 MHz, $CDCl_3$) δ 171.9, 163.0, 141.5, 134.2, 123.0, 115.7, 61.8, 24.3, 14.4.

MS (m/z): M^+: calculated for $C_{10}H_{12}O_3$: 180.1; found 180.1.

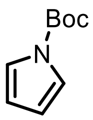

^{1}H NMR (400 MHz, $CDCl_3$) δ 11.37 (s, 1H), 6.84 (d, *J* = 8.3 Hz, 1H), 6.72 (d, *J* = 7.5 Hz, 1H), 4.44 (q, *J* = 7.1 Hz, 2H), 2.56 (s, 3H), 1.44 (t, *J* = 7.1 Hz, 3H). ^{13}C NMR (100 MHz, $CDCl_3$) δ 149.1, 120.1, 112.0, 83.7, 28.1.

MS (m/z): M^+: calculated for $C_9H_{13}NO_2$: 167.1; found 167.0.

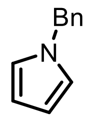

^{1}H NMR (400 MHz, $CDCl_3$) δ 7.38 – 7.26 (m, 3H), 7.11 (d, *J* = 6.7 Hz, 2H), 6.69 (t, *J* = 2.1 Hz, 2H), 6.19 (t, *J* = 2.1 Hz, 2H), 5.07 (s, 2H). ^{13}C NMR (100 MHz, $CDCl_3$) δ 138.3, 128.8, 127.8, 127.1, 121.3, 108.6, 53.46.

HRMS-ESI (m/z): $[M+H]^+$: calculated for $C_{11}H_{12}N$: 158.0964; found 158.0961.

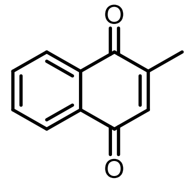

^{1}H NMR (400 MHz, $CDCl_3$) δ 8.14 – 8.04 (m, 2H), 7.76 – 7.70 (m, 2H), 6.87 – 6.83 (m, 1H), 2.21 (d, *J* = 1.6 Hz, 3H). ^{13}C NMR (100 MHz, $CDCl_3$) δ 185.7, 185.2, 148.3, 135.8, 133.8, 133.7, 132.4, 132.3, 126.7, 126.2, 16.6.

MS (m/z): M^+: calculated for $C_{11}H_8O_2$: 172.1; found 172.0.

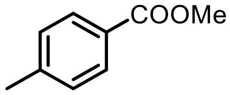

^{1}H NMR (500 MHz, $CDCl_3$) δ 7.93 (d, *J* = 8.0 Hz, 2H), 7.23 (d, *J* = 8.0 Hz, 2H), 3.90 (s, 3H), 2.41 (s, 3H). ^{13}C NMR (125 MHz, $CDCl_3$) δ 167.3, 143.7, 129.7, 129.2, 127.5, 52.1, 21.8.

HRMS-ESI (m/z): $[M+H]^+$: calculated for $C_9H_{11}O_2$:151.0754; found 151.0751.

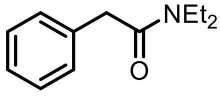

^{1}H NMR (500 MHz, $CDCl_3$) δ 7.34 - 7.29 (m, 2H), 7.26 - 7.21 (m, 3H), 3.70 (s, 2H), 3.39 (q, *J* = 7.1 Hz, 2H), 3.30 (q, *J* = 7.1 Hz, 2H), 1.13 (t, *J* = 7.1 Hz, 3H), 1.09 (t, *J* = 7.1 Hz, 3H). ^{13}C NMR (125 MHz, $CDCl_3$) δ 170.2, 135.6, 128.8, 128.7, 126.8, 42.5, 41.0, 40.3, 14.3, 13.1.

HRMS-ESI (m/z): $[M+H]^+$: calculated for $C_{12}H_{18}NO$:192.1383; found 192.1379.

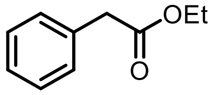

^{1}H NMR (500 MHz, $CDCl_3$) δ 7.35 – 7.26 (m, 5H), 4.15 (q, *J* = 7.1 Hz, 2H), 3.61 (s, 2H), 1.25 (t, *J* = 7.1 Hz, 3H). ^{13}C NMR (125 MHz, $CDCl_3$) δ 171.8, 134.3, 129.4, 128.7, 127.2, 61.0, 41.6, 14.3.

HRMS-ESI (m/z): $[M+H]^+$: calculated for $C_{10}H_{13}O_2$:165.0910; found 165.0908.

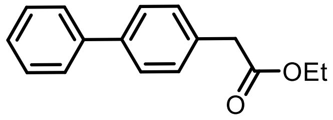

^{1}H NMR (500 MHz, $CDCl_3$) δ 7.60 – 7.53 (m, 4H), 7.43 (t, *J* = 7.7 Hz, 2H), 7.37 – 7.31 (m, 3H), 4.17 (q, *J* = 7.1 Hz, 2H), 3.65 (s, 2H), 1.27 (t, *J* = 7.1 Hz, 3H). ^{13}C NMR (125 MHz, $CDCl_3$) δ 171.7, 140.9, 140.1, 133.3, 129.8, 128.9, 127.4, 127.4, 127.2, 61.1, 41.2, 14.3.

HRMS-ESI (m/z): $[M+H]^+$: calculated for $C_{16}H_{17}O_2$:241.1223; found 241.1218.

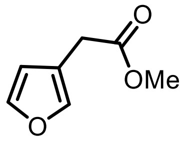

^{1}H NMR (500 MHz, $CDCl_3$) δ 7.39 (s, 2H), 6.39 (s, 1H), 3.72 (s, 3H), 3.48 (s, 2H). ^{13}C NMR (125 MHz, $CDCl_3$) δ 171.8, 143.2, 140.5, 117.4, 111.5, 52.2, 30.8.

MS (m/z): M^+: calculated for $C_7H_8O_3$: 140.0; found 140.0.

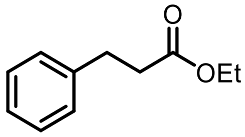

^{1}H NMR (500 MHz, $CDCl_3$) δ 7.32 – 7.27 (m, 2H), 7.23 – 7.18 (m, 3H), 4.13 (q, *J* = 7.1 Hz, 2H), 2.95 (t, *J* = 7.9 Hz, 2H), 2.62 (t, *J* = 7.9 Hz, 2H), 1.23 (t, *J* = 7.1 Hz, 3H). ^{13}C NMR (125 MHz, $CDCl_3$) δ 173.1, 140.7, 128.6, 128.4, 126.4, 60.6, 53.6, 36.1, 31.1, 14.3.

HRMS-ESI (m/z): $[M+H]^+$: calculated for $C_{11}H_{15}O_2$:179.1067; found 179.1062.

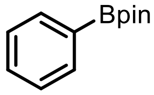

^{1}H NMR (500 MHz, $CDCl_3$) δ 7.81 (d, *J* = 6.4 Hz, 2H), 7.46 (t, *J* = 7.4 Hz, 1H), 7.37 (t, *J* = 7.3 Hz, 2H), 1.35 (s, 12H). ^{13}C NMR (125 MHz, $CDCl_3$) δ 134.9, 131.4, 127.8, 83.9, 25.0.

MS (m/z): M^+: calculated for $C_{12}H_{17}BO_2$: 204.1; found 204.1.

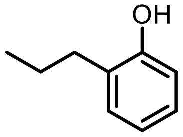

^{1}H NMR (400 MHz, $CDCl_3$) δ 7.15 – 7.04 (m, 2H), 6.87 (t, *J* = 7.4 Hz, 1H), 6.76 (d, *J* = 7.9 Hz, 1H), 4.74 (s, 1H), 2.58 (t, *J* = 7.8 Hz, 2H), 1.70 – 1.59 (m, 2H), 0.98 (t, *J* = 7.3 Hz, 3H). ^{13}C NMR (100 MHz, $CDCl_3$) δ 153.6, 130.4, 128.5, 127.2, 120.8, 115.3, 32.1, 23.0, 14.2.

HRMS-ESI (m/z): $[M+H]^+$: calculated for $C_9H_{13}O$:137.0961; found 137.0960.

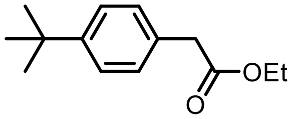

^{1}H NMR (400 MHz, CDCl$_3$) δ 7.34 (d, *J* = 8.4 Hz, 2H), 7.22 (d, *J* = 8.4 Hz, 2H), 4.15 (q, *J* = 7.1 Hz, 2H), 3.58 (s, 2H), 1.31 (s, 9H), 1.26 (t, *J* = 7.1 Hz, 3H). ^{13}C NMR (100 MHz, CDCl$_3$) δ 172.0, 150.0, 131.2, 129.0, 125.6, 60.9, 41.0, 34.6, 31.5, 14.4.

HRMS-ESI (m/z): [M+H]$^+$: calculated for $C_{14}H_{21}O_2$:221.1536; found 221.1531

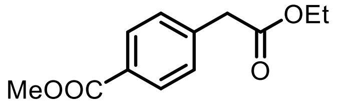

^{1}H NMR (400 MHz, CDCl$_3$) δ 8.00 (d, *J* = 8.3 Hz, 2H), 7.36 (d, *J* = 8.3 Hz, 2H), 4.16 (q, *J* = 7.1 Hz, 3H), 3.91 (s, 3H), 3.67 (s, 2H), 1.25 (t, *J* = 7.1 Hz, 7H). ^{13}C NMR (100 MHz, CDCl$_3$) δ 171.0, 167.0, 139.4, 130.0, 129.5, 129.1, 61.2, 52.3, 41.5, 14.3.

HRMS-ESI (m/z): [M+H]$^+$: calculated for $C_{12}H_{15}O_4$:223.0965; found 223.0959

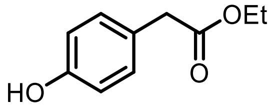

^{1}H NMR (400 MHz, CDCl$_3$) δ 7.13 (d, *J* = 8.5 Hz, 2H), 6.76 (d, *J* = 8.5 Hz, 2H), 5.37 (s, 1H), 4.15 (q, *J* = 7.1 Hz, 2H), 3.54 (s, 2H), 1.25 (t, *J* = 7.1 Hz, 3H). ^{13}C NMR (100 MHz, CDCl$_3$) δ 172.5, 154.9, 130.6, 126.2, 115.6, 61.1, 40.6, 14.3.

HRMS-ESI (m/z): [M+H]$^+$: calculated for $C_{10}H_{13}O_3$:181.0859; found 181.0855

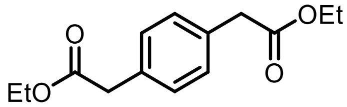

^{1}H NMR (400 MHz, CDCl$_3$) δ 7.25 (s, 4H), 4.14 (q, *J* = 7.1 Hz, 4H), 3.59 (s, 4H), 1.25 (t, *J* = 7.1 Hz, 6H). ^{13}C NMR (100 MHz, CDCl$_3$) δ 171.7, 133.0, 129.6, 61.0, 41.2, 14.3.

HRMS-ESI (m/z): [M+H]$^+$: calculated for $C_{14}H_{19}O_4$:251.1278; found 251.1272

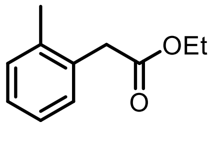

^{1}H NMR (500 MHz, CDCl$_3$) δ 7.22 – 7.13 (m, 4H), 4.15 (q, *J* = 7.1 Hz, 2H), 3.63 (s, 2H), 2.32 (s, 3H), 1.25 (t, *J* = 7.1 Hz, 3H). ^{13}C NMR (125 MHz, CDCl$_3$) δ 171.7, 137.0, 133.0, 130.4, 130.3, 127.5, 126.2, 60.9, 39.4, 19.8, 14.3.

HRMS-ESI (m/z): [M+H]$^+$: calculated for $C_{11}H_{15}O_2$:179.1067; found 179.1063

NMR spectra

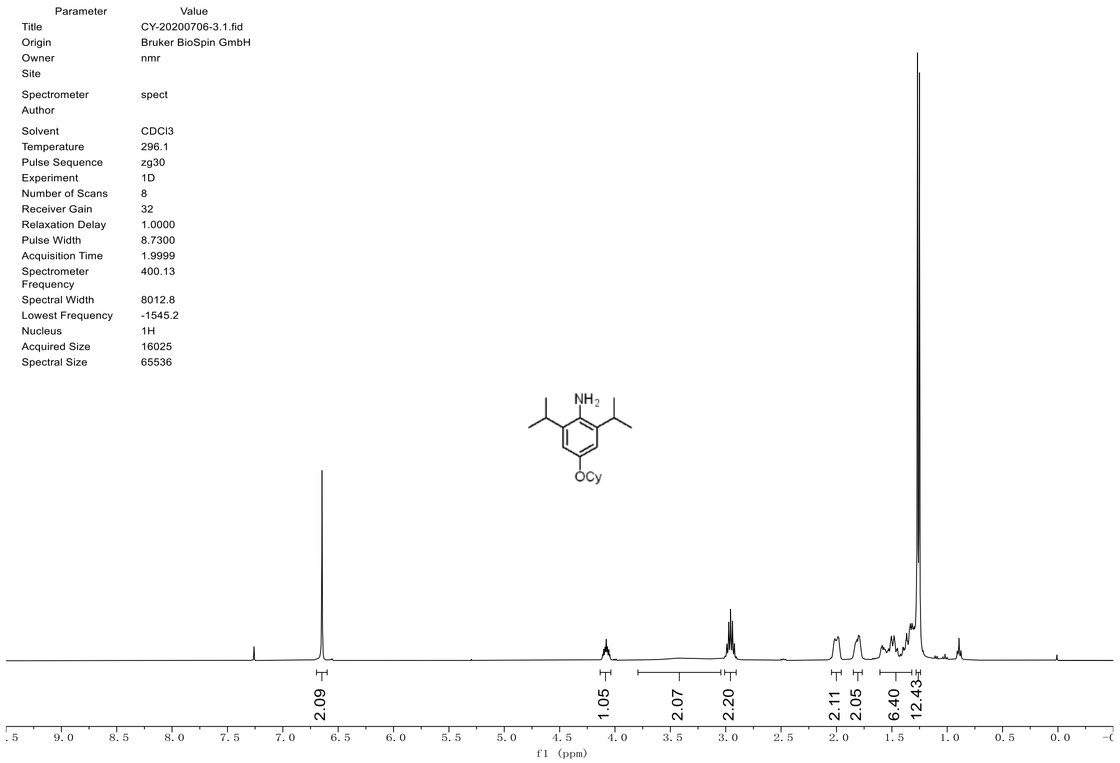

Supplementary Figure 73. ^{1}H NMR of amine for **L5***HBF_4

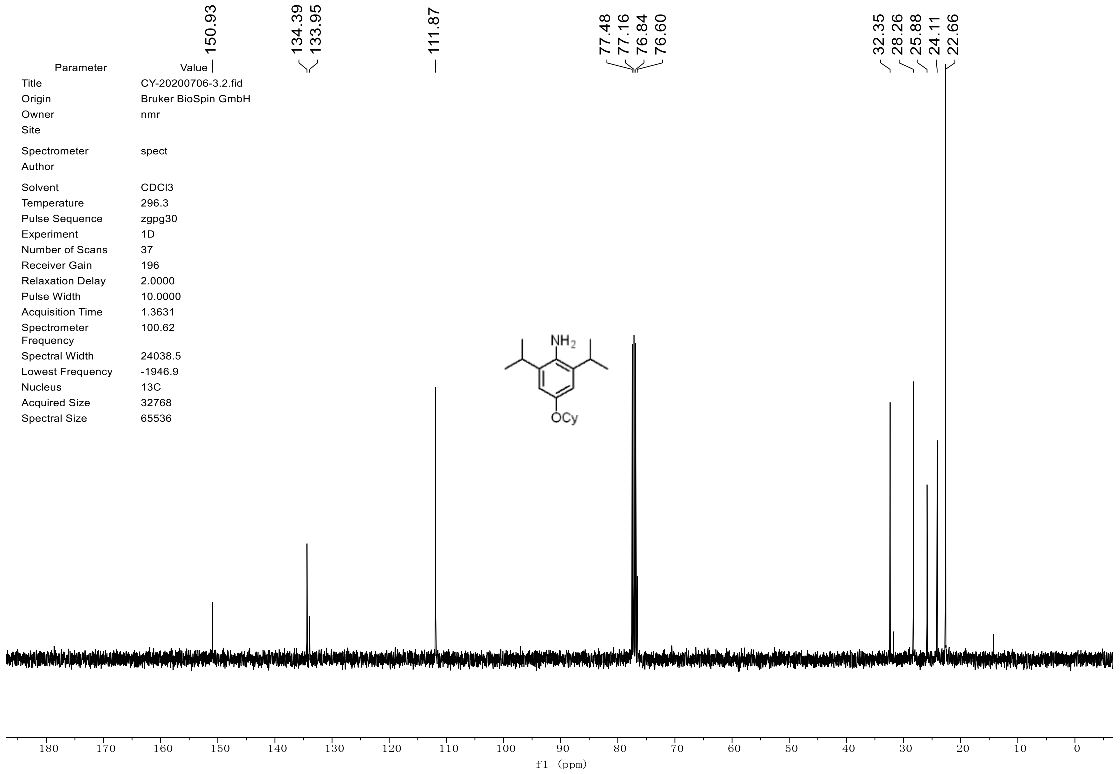

Supplementary Figure 74. ^{13}C NMR of amine for **L5***HBF_4

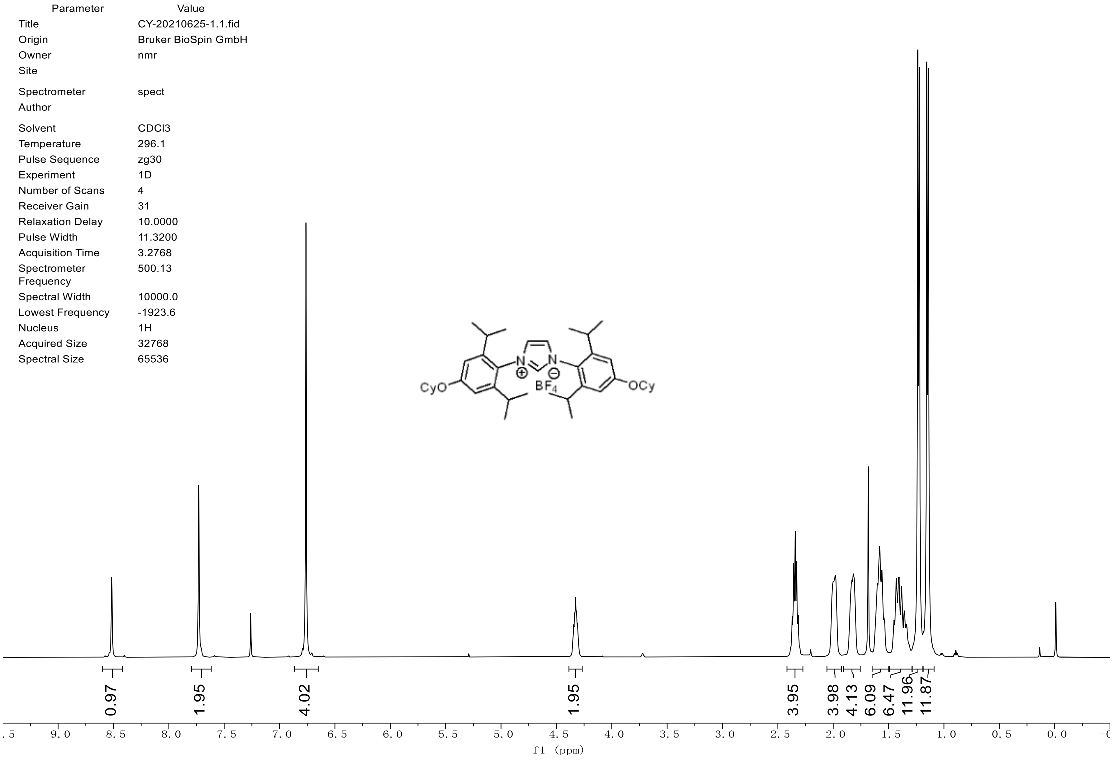

Supplementary Figure 75. ^{1}H NMR of **L5***HBF_4

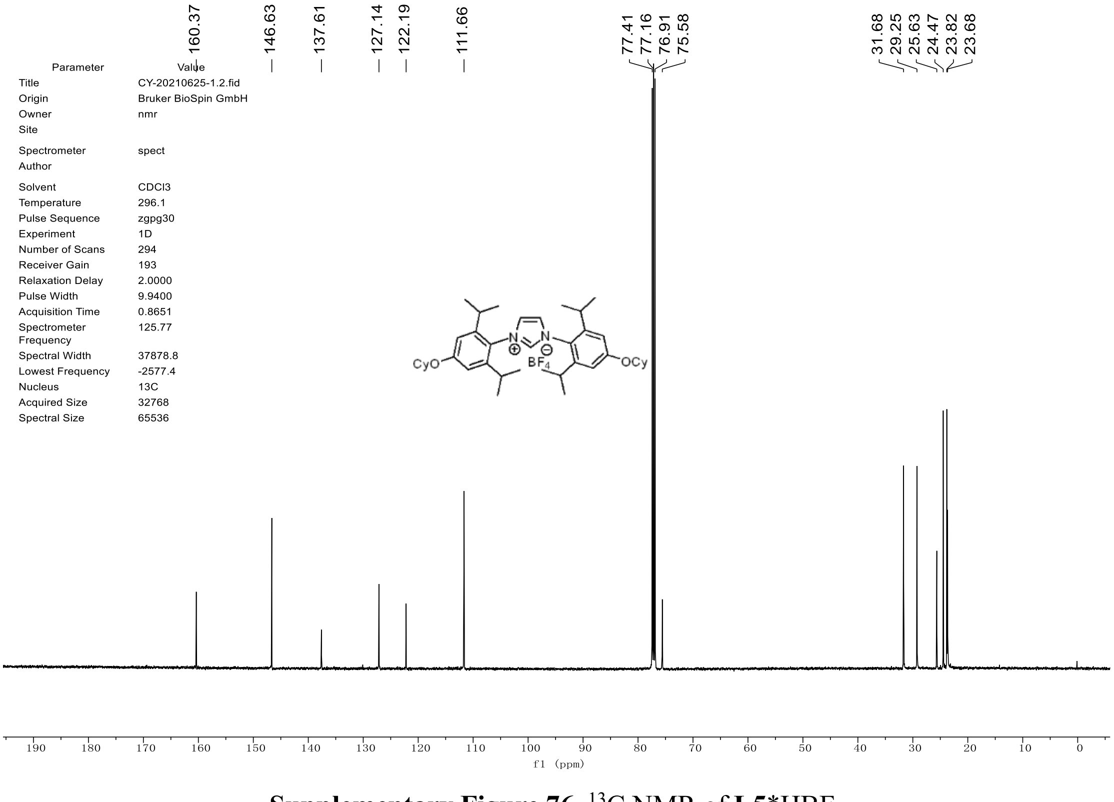

Supplementary Figure 76. ^{13}C NMR of **L5***HBF_4

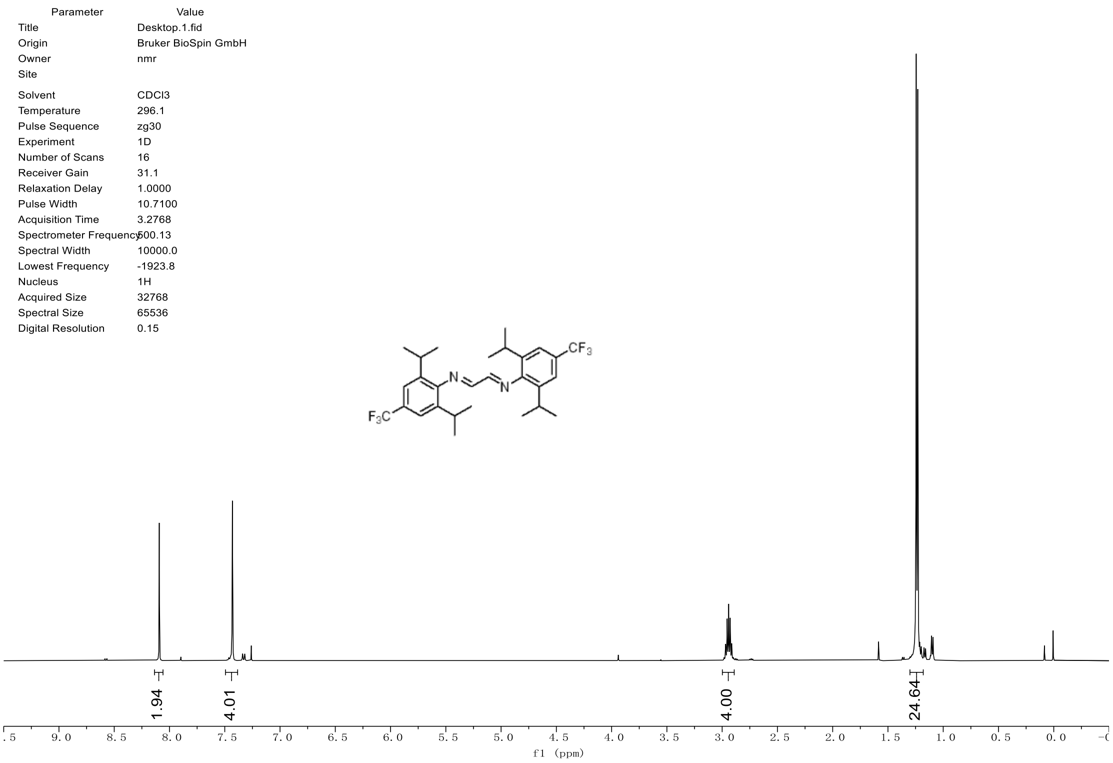

Supplementary Figure 77. ^{1}H NMR of amine for **L6***HCl

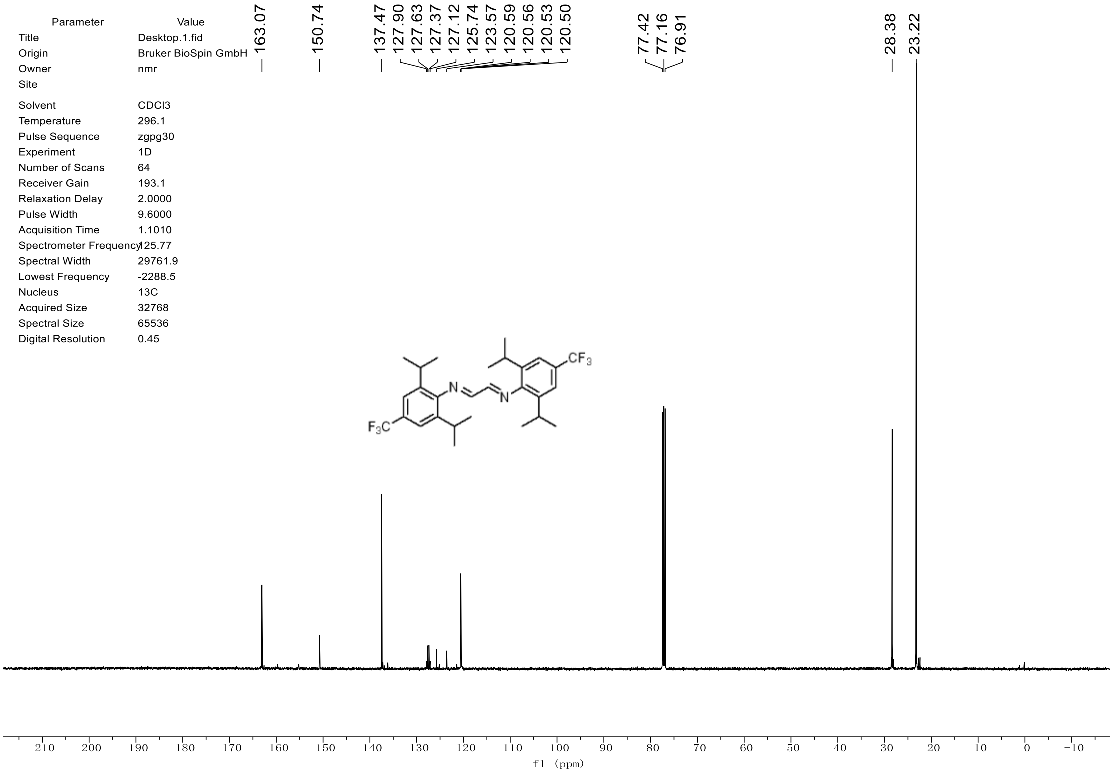

Supplementary Figure 78. ^{13}C NMR of amine for **L6***HCl

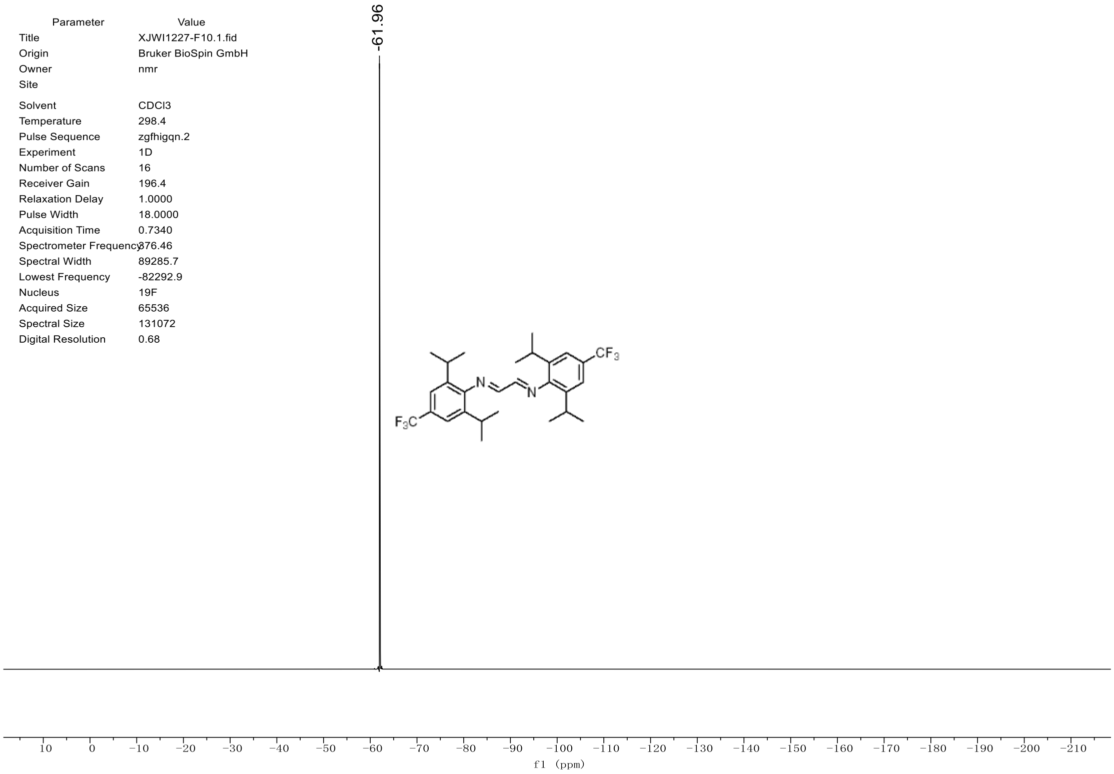

Supplementary Figure 79. ^{19}F NMR of amine for **L6***HCl

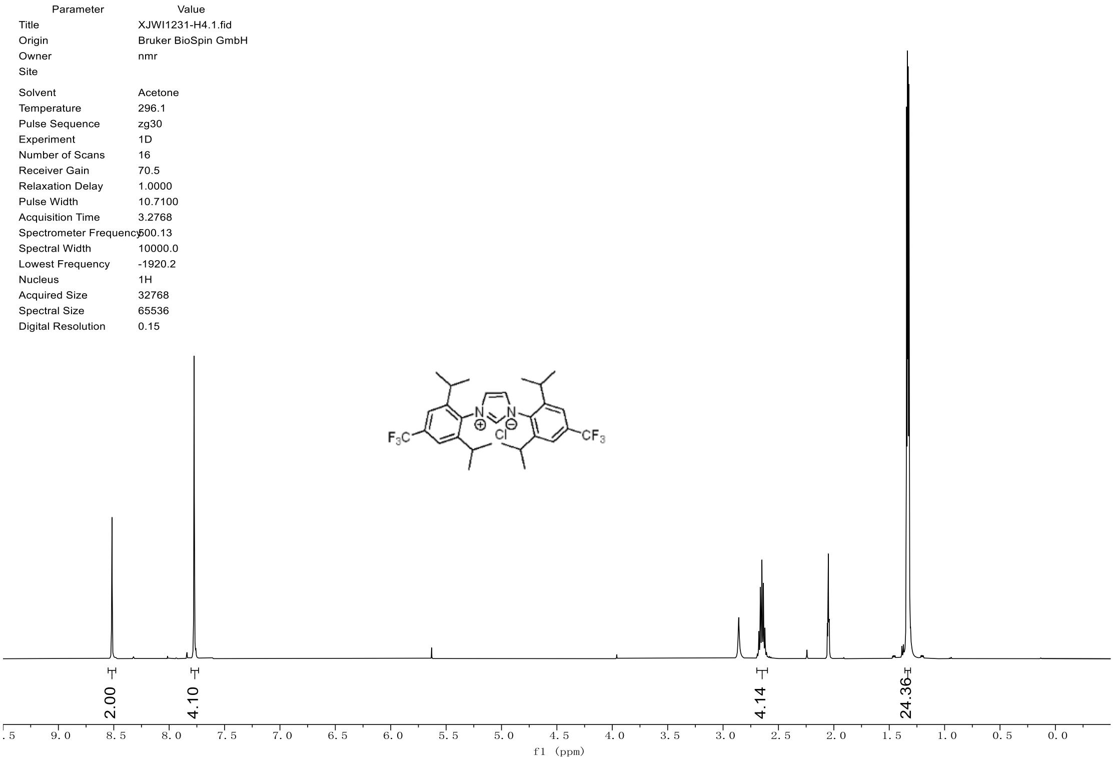

Supplementary Figure 80. ^{1}H NMR of amine for **L6***HCl

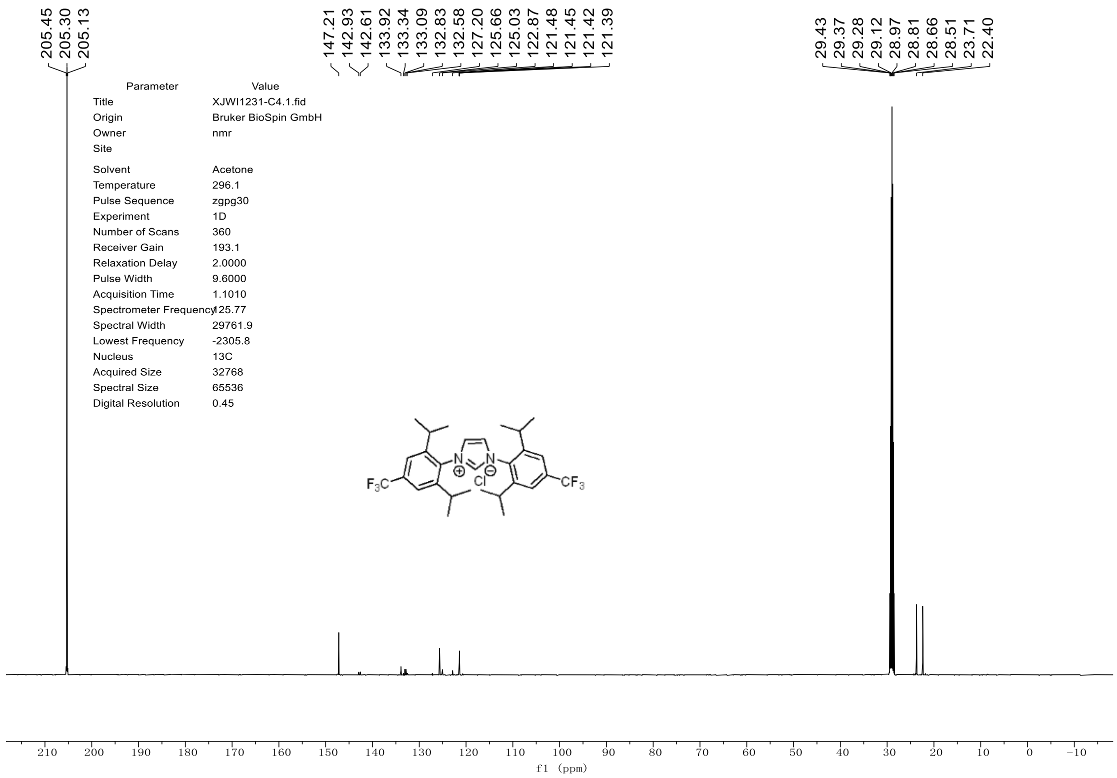

Supplementary Figure 81. ^{13}C NMR of amine for **L6***HCl

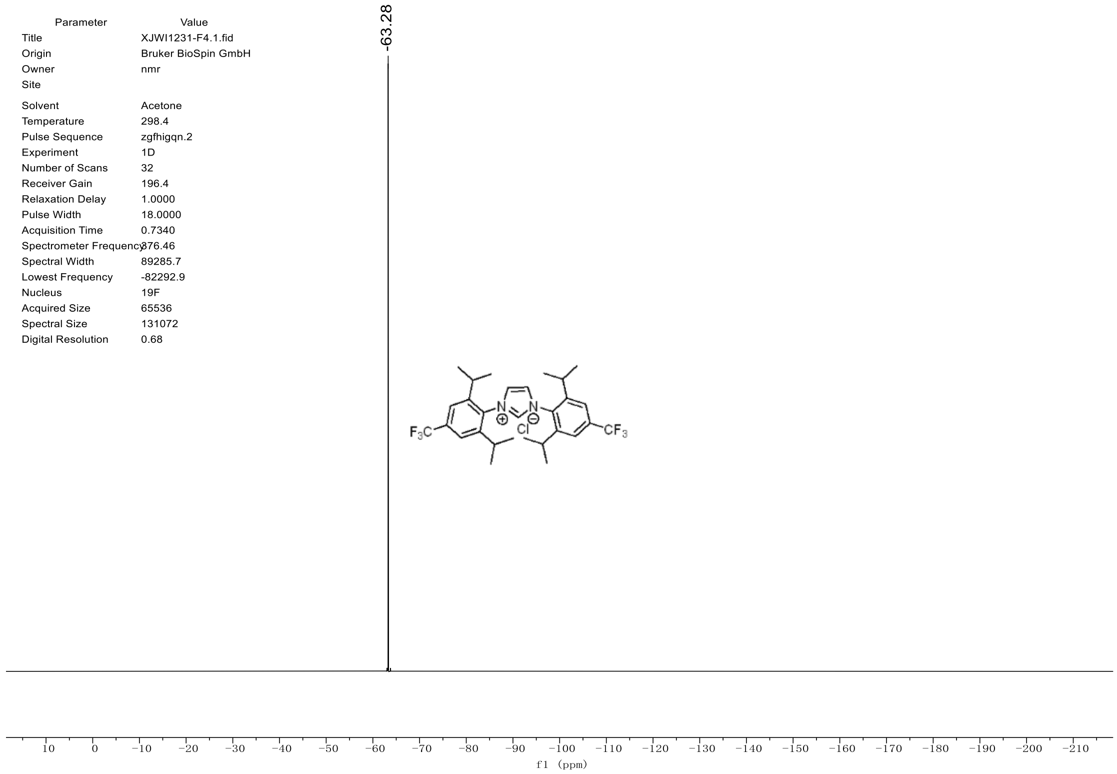

Supplementary Figure 82. ^{19}F NMR of amine for **L6***HCl

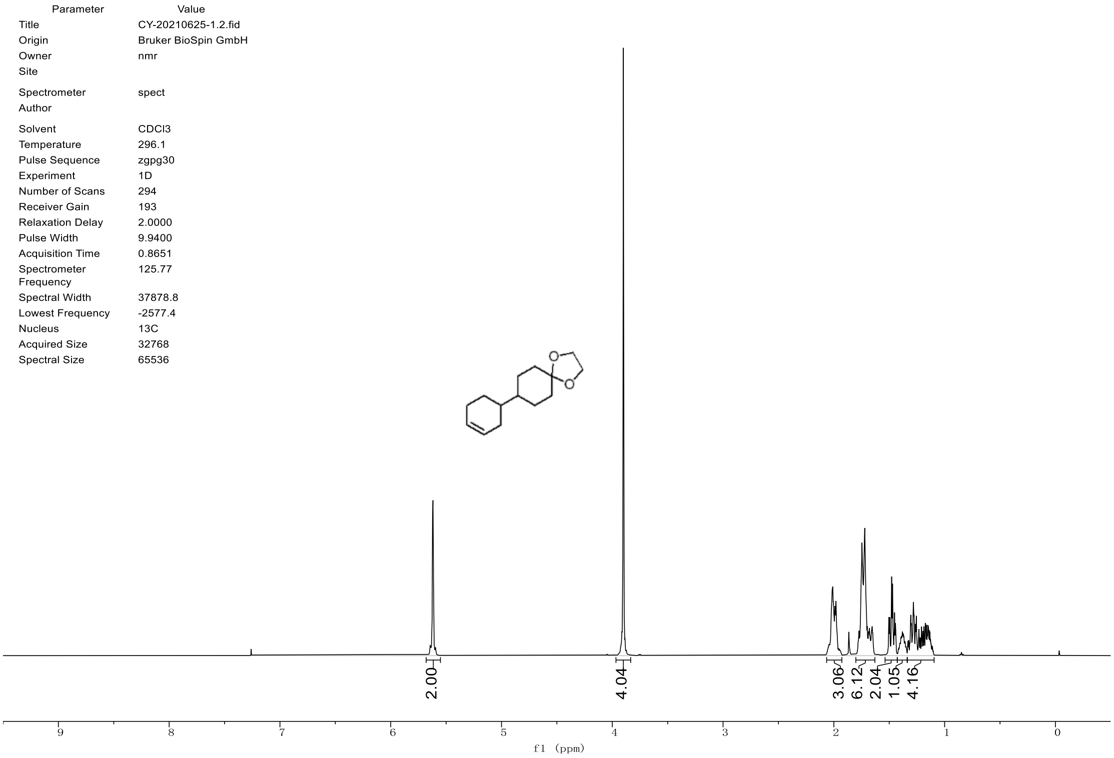

Supplementary Figure 83. ^{1}H NMR of acetal for **1r**

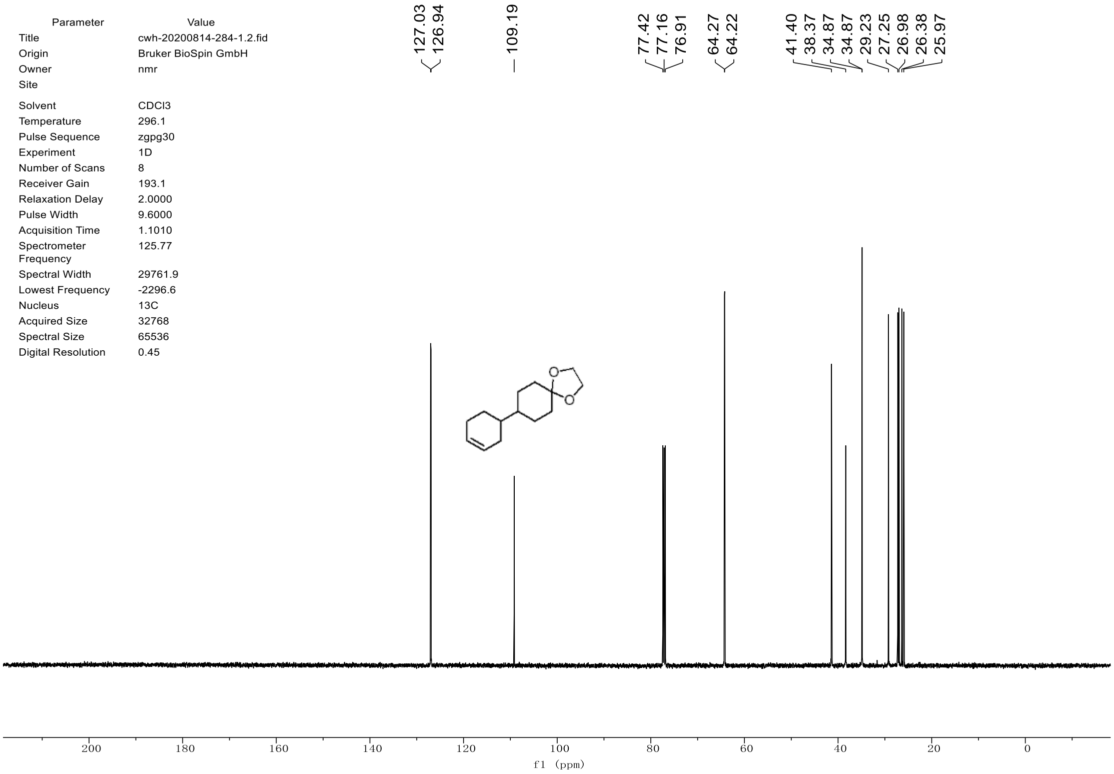

Supplementary Figure 84. ^{13}C NMR of acetal for **1r**

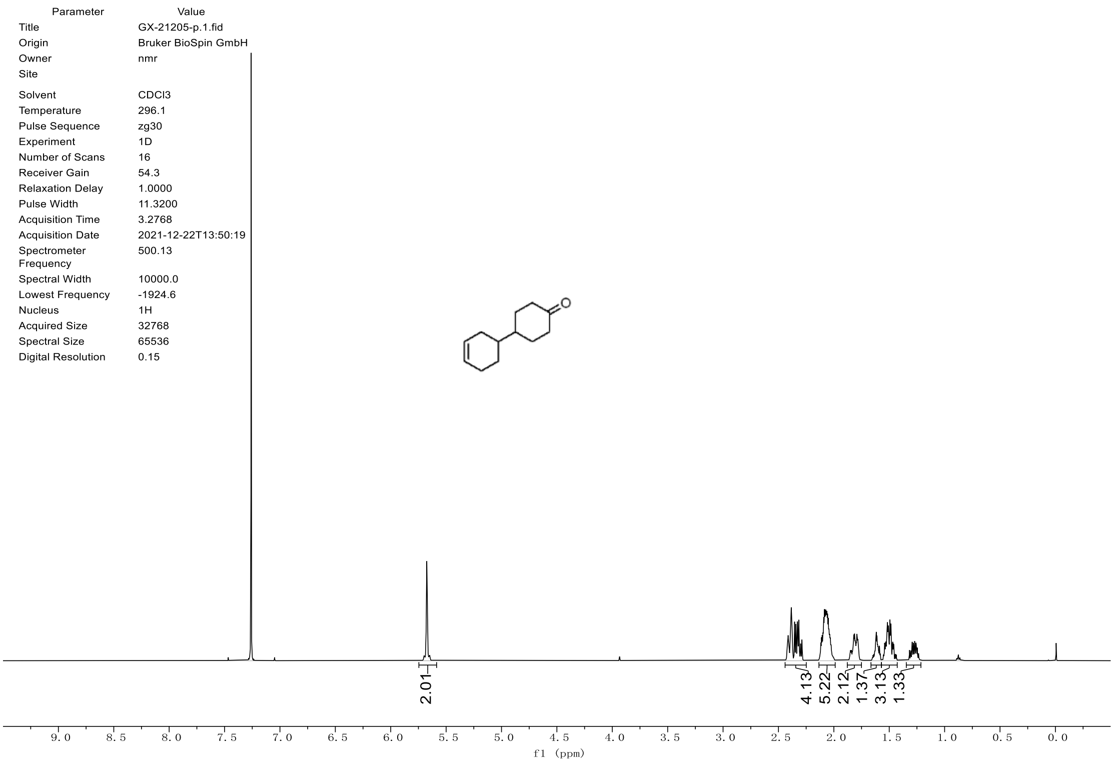

Supplementary Figure 85. ^{1}H NMR of **1r**

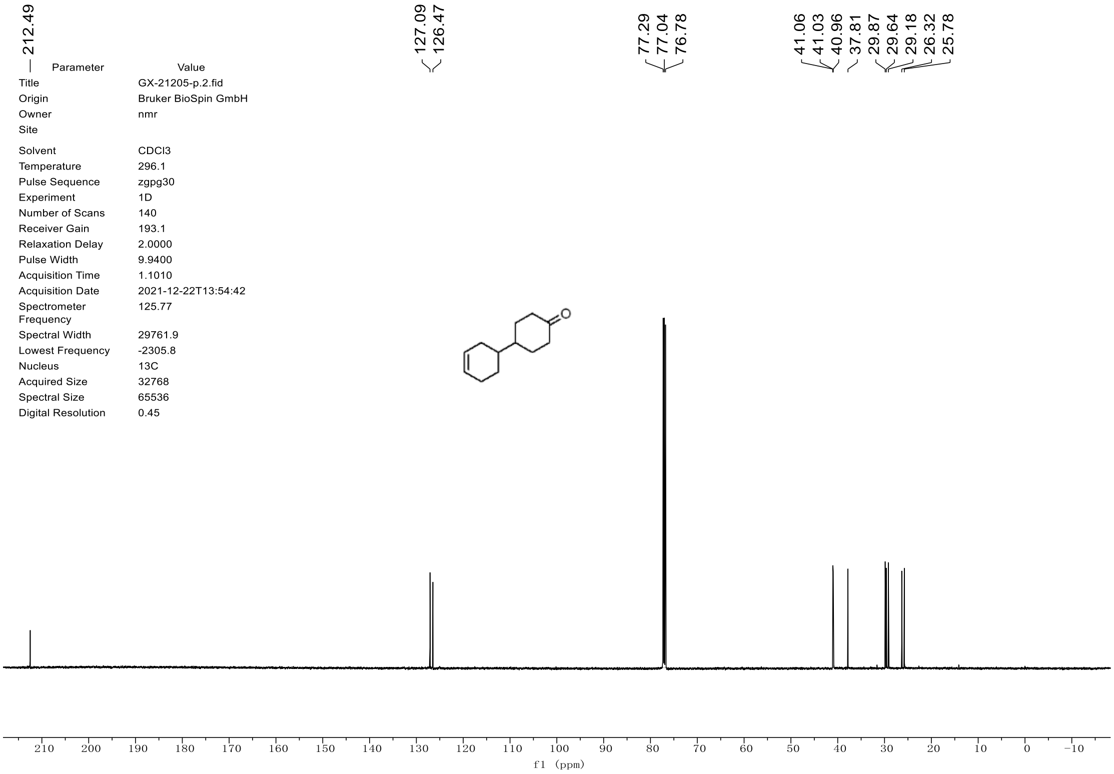

Supplementary Figure 86. ^{13}C NMR of **1r**

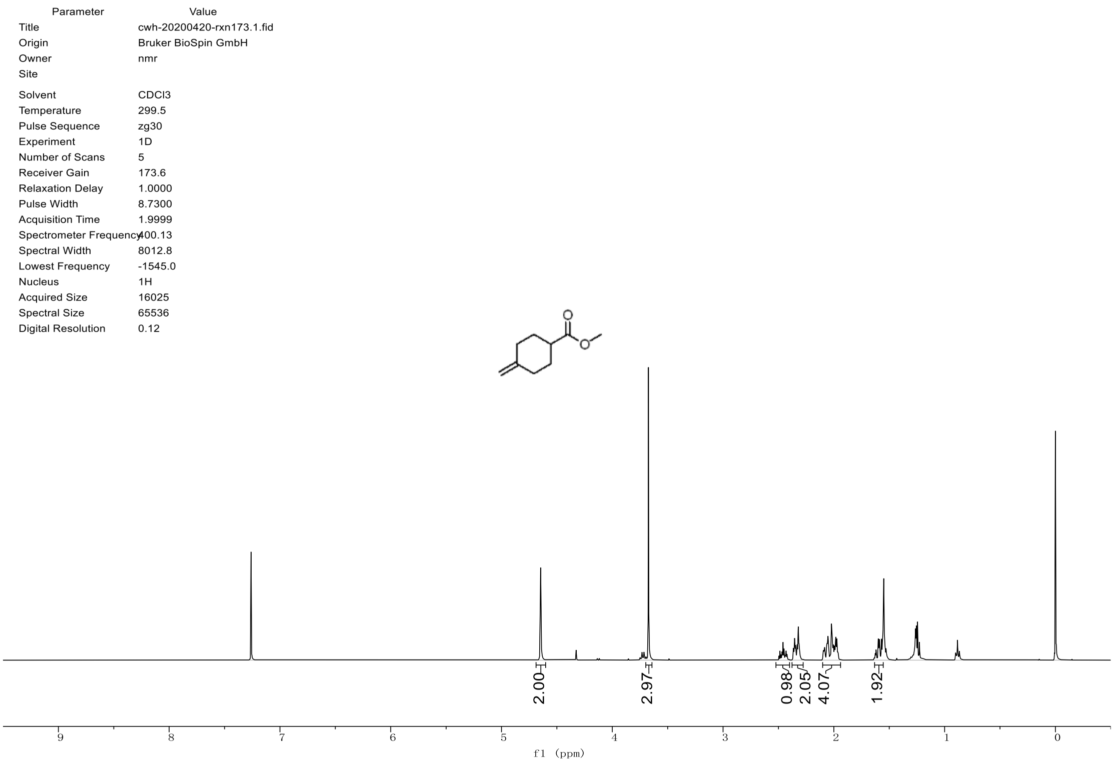

Supplementary Figure 87. ^{1}H NMR of **1′b**

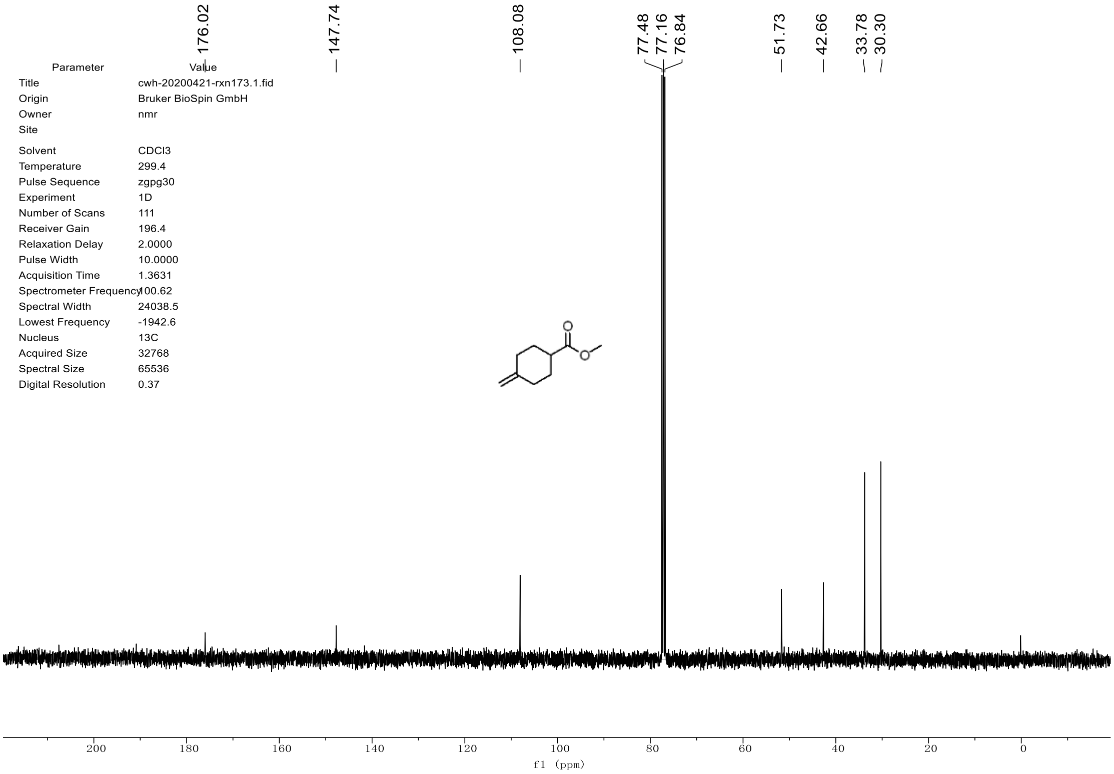

Supplementary Figure 88. ^{13}C NMR of **1′b**

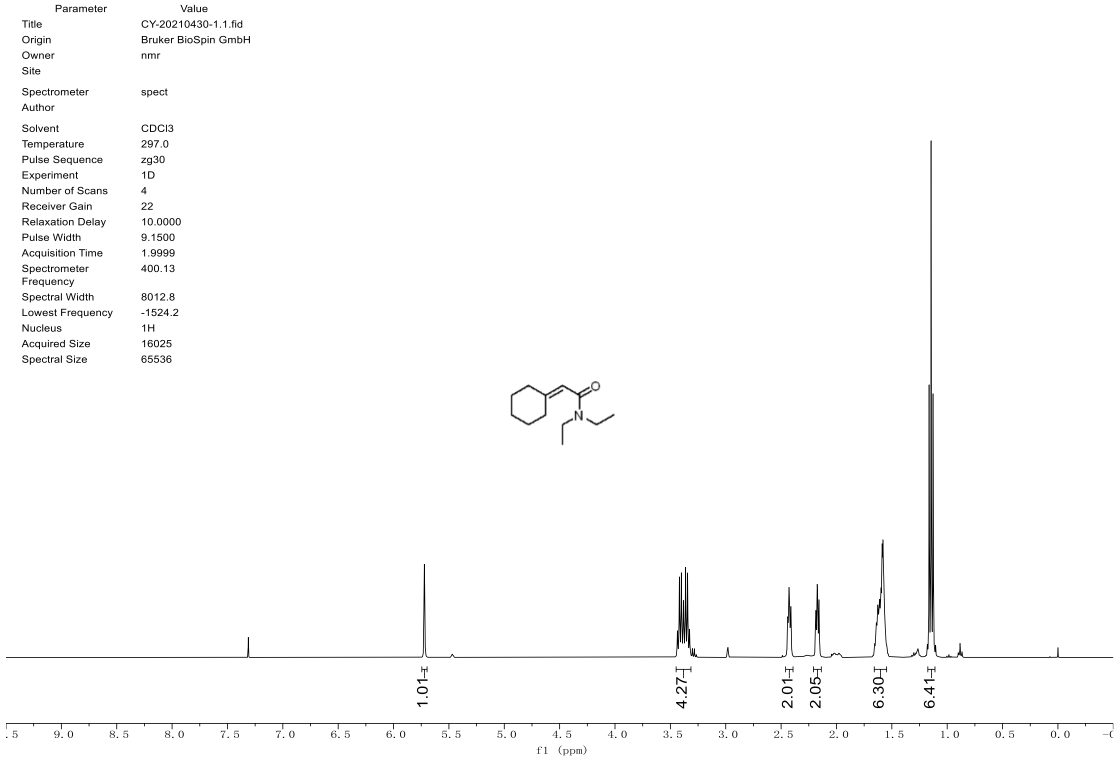

Supplementary Figure 89. ^{1}H NMR of **1′m**

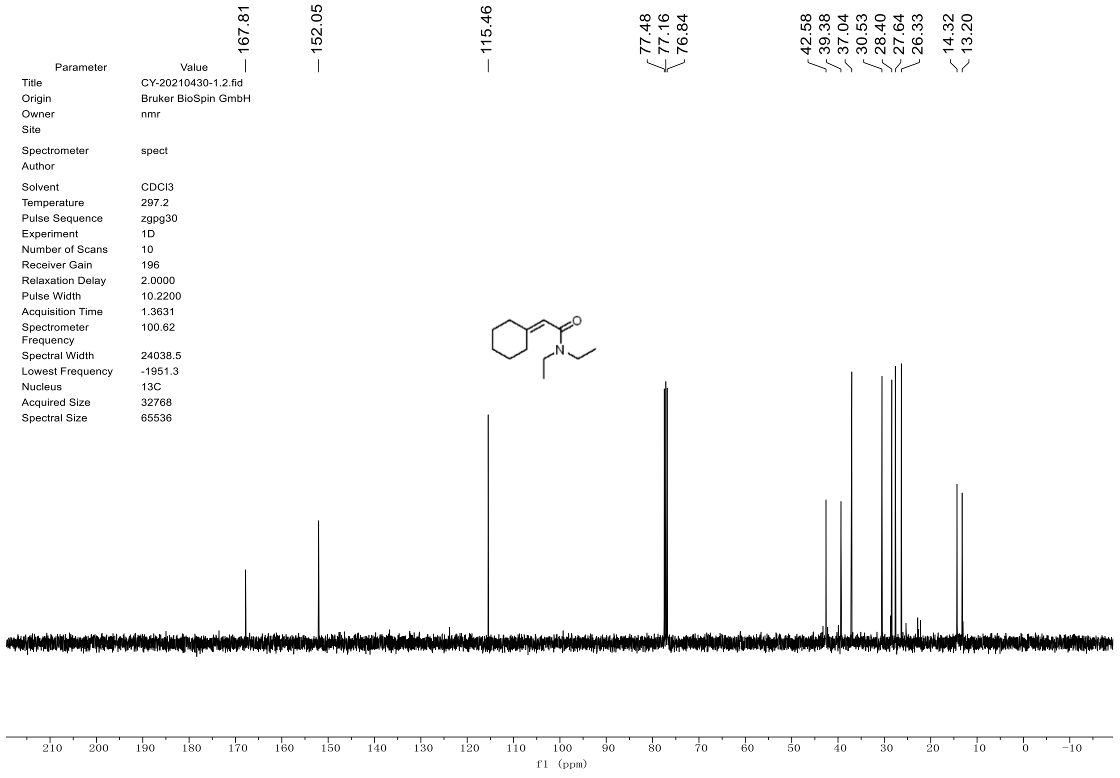

Supplementary Figure 90. ^{13}C NMR of **1′m**

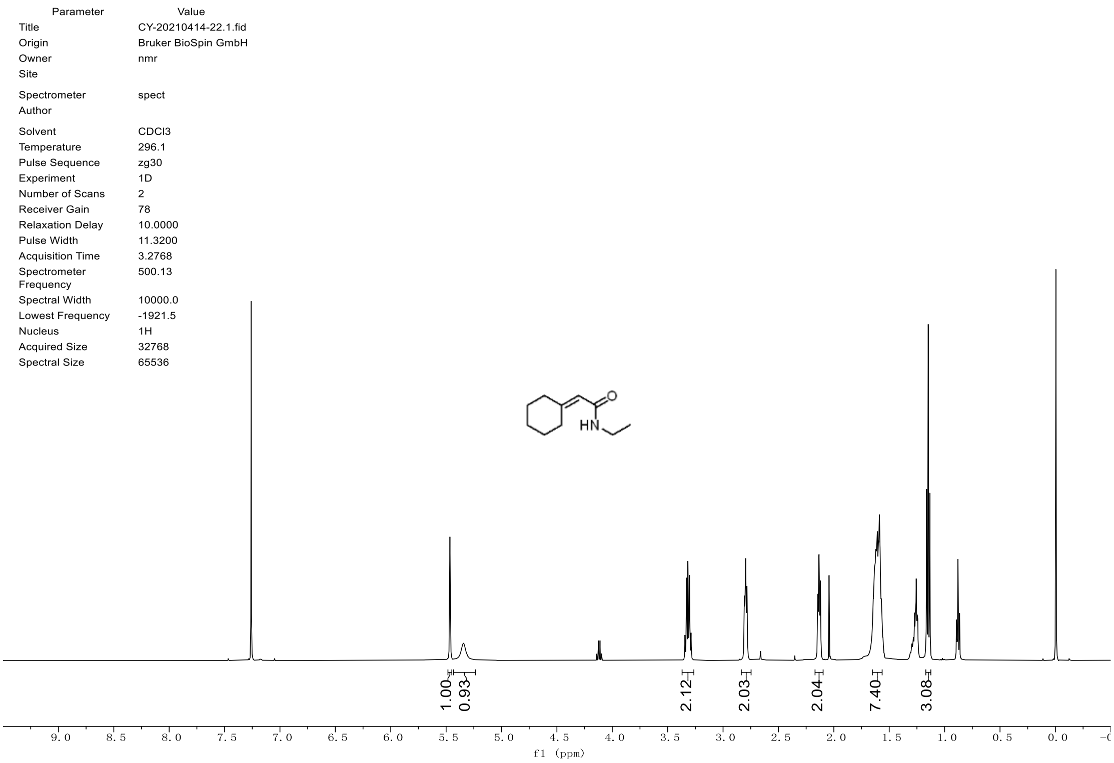

Supplementary Figure 91. ^{1}H NMR of **1′n**

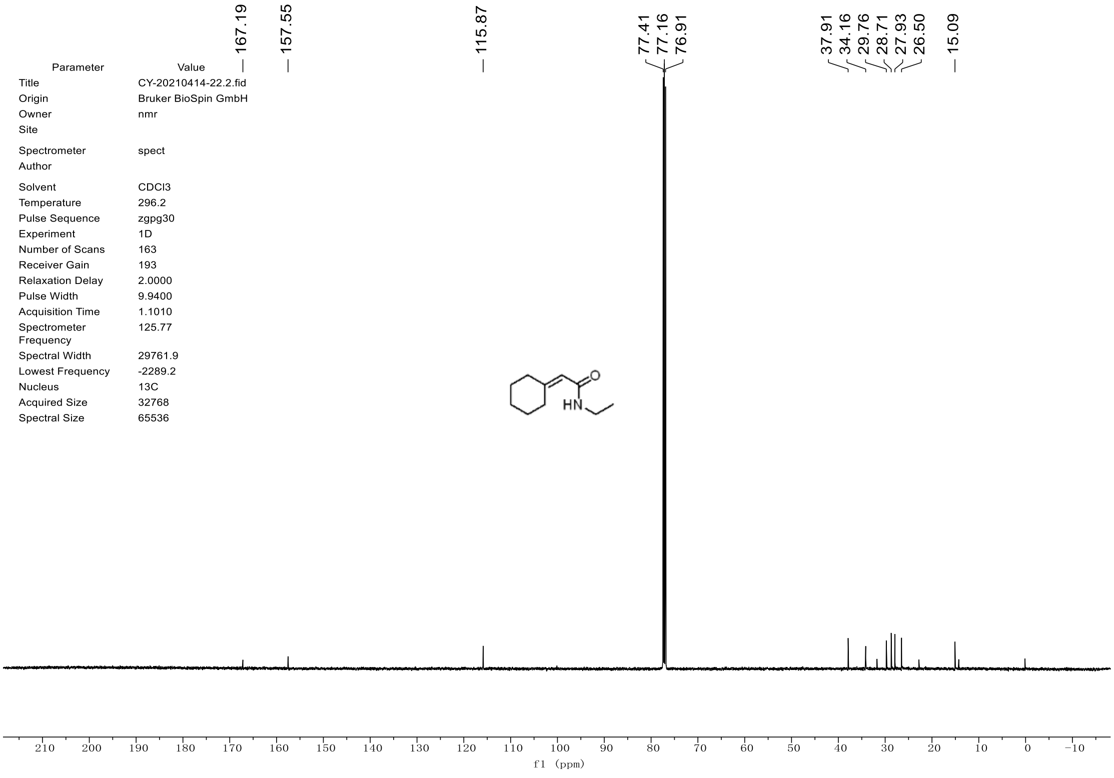

Supplementary Figure 92. ^{13}C NMR of **1′n**

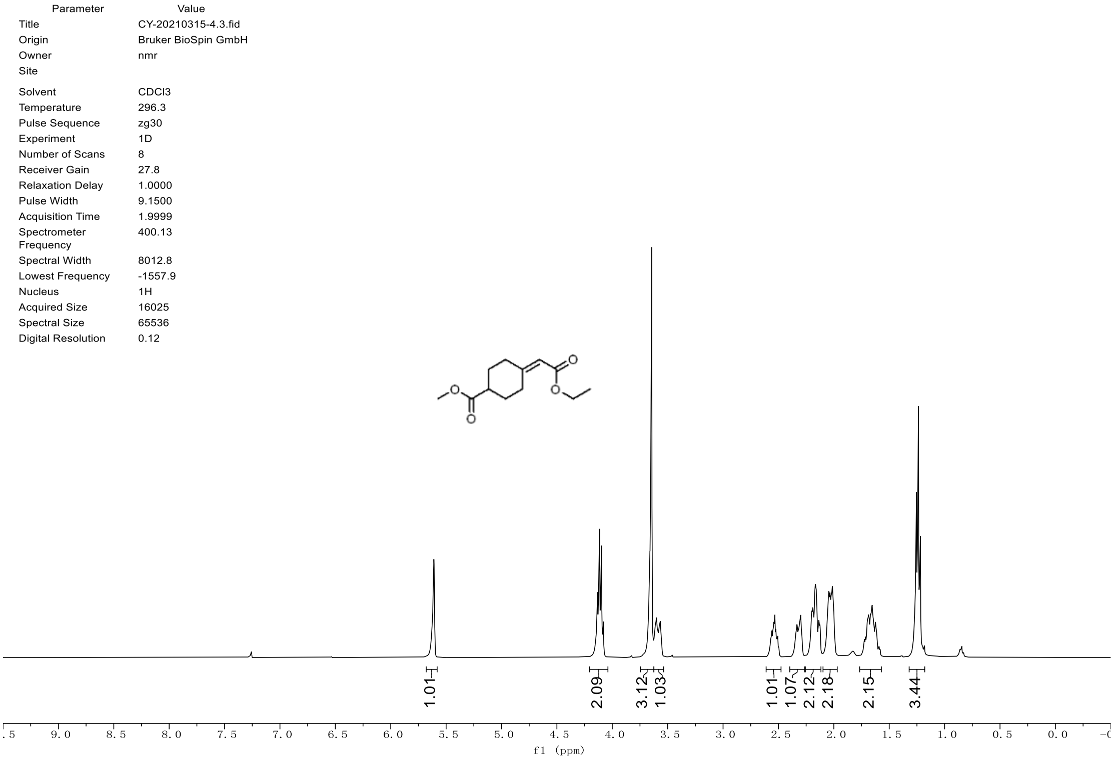

Supplementary Figure 93. ^{1}H NMR of **1′r**

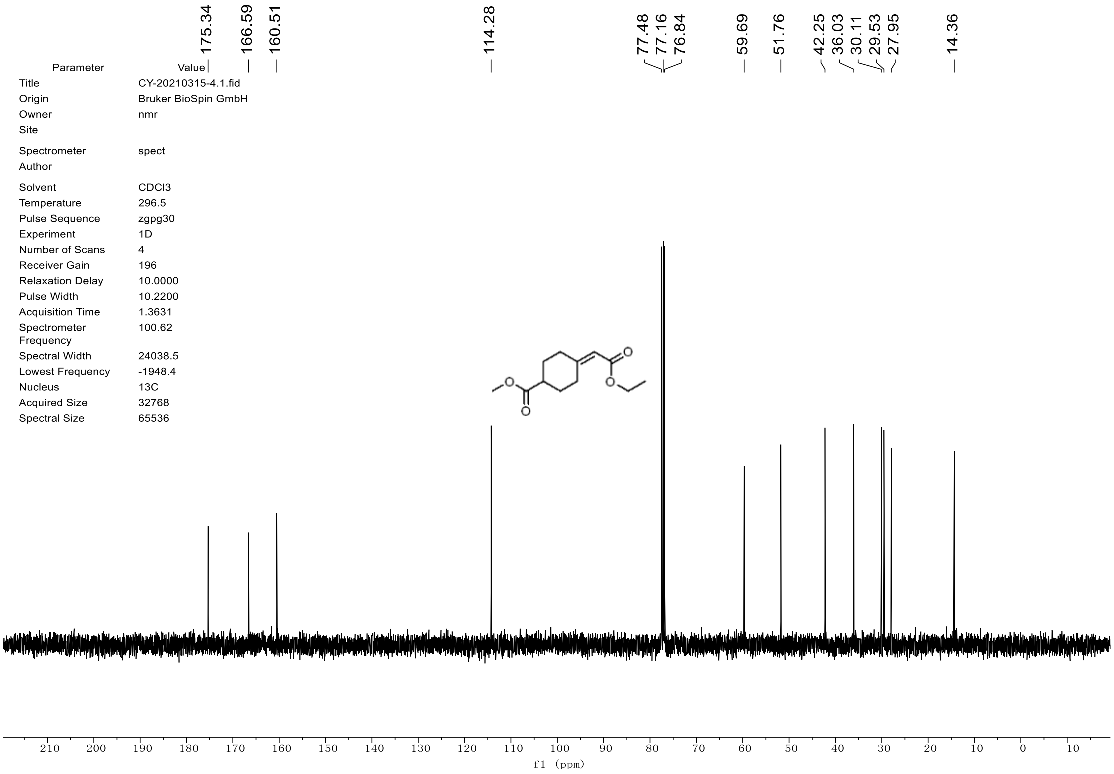

Supplementary Figure 94. ^{13}C NMR of **1′r**

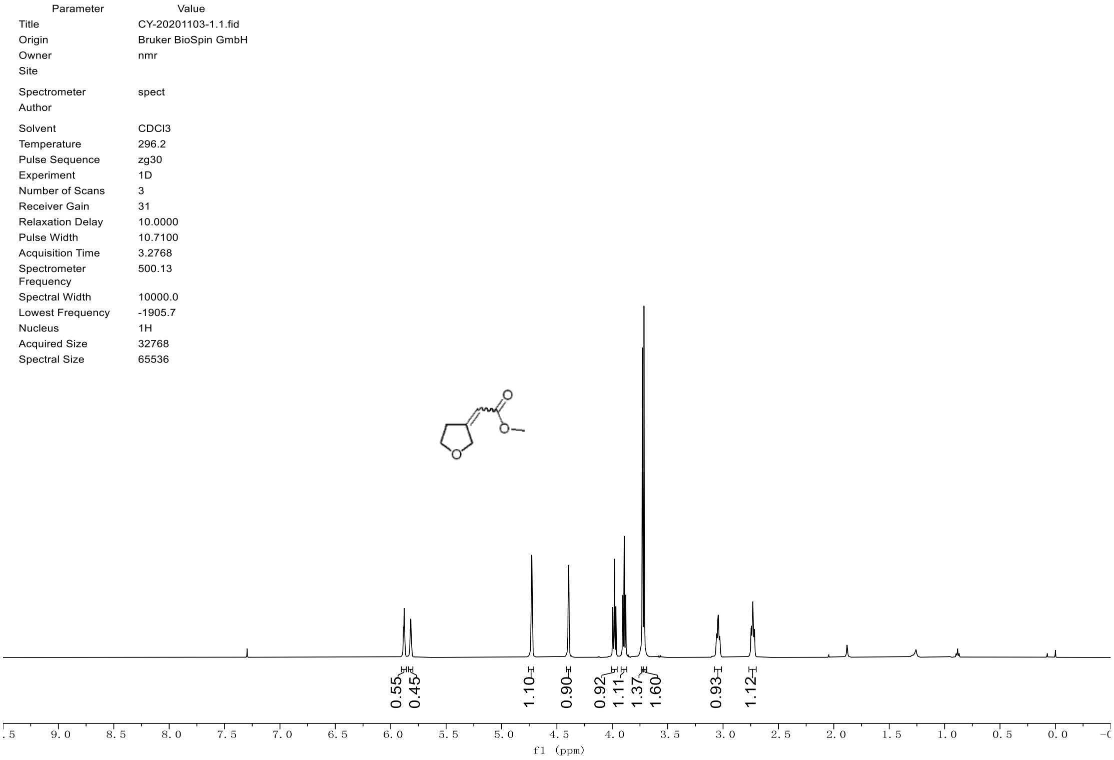

Supplementary Figure 95. ^{1}H NMR of **1′u**

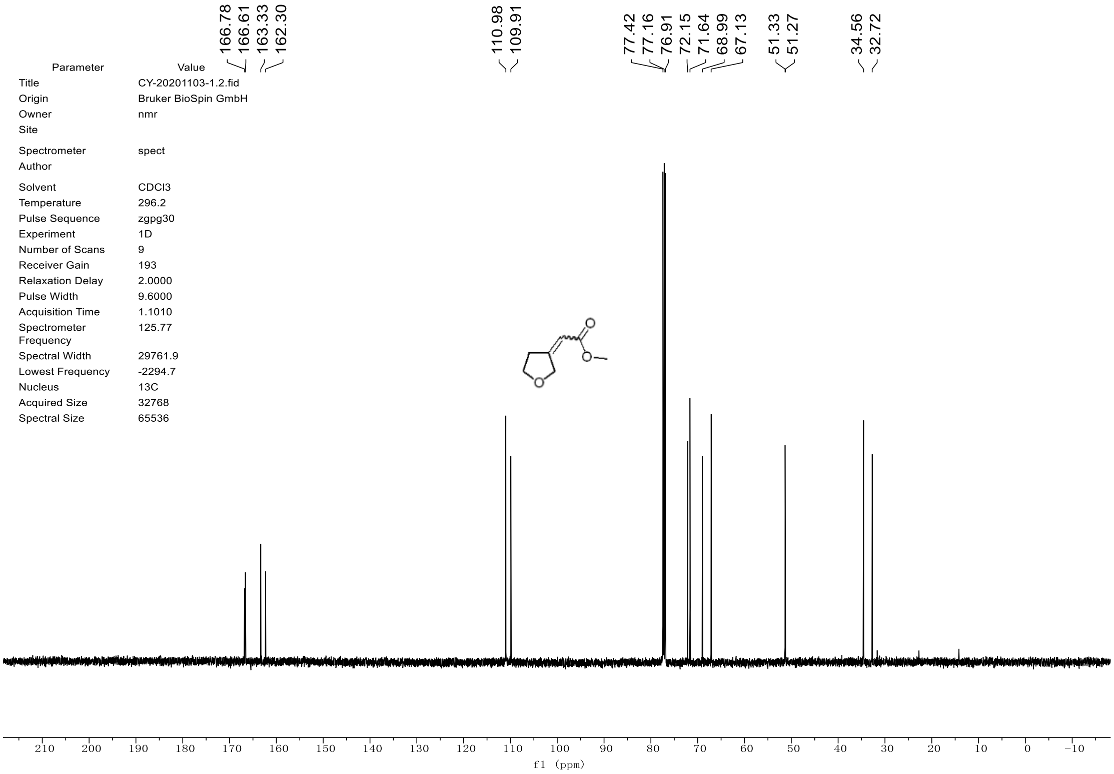

Supplementary Figure 96. ^{13}C NMR of **1′u**

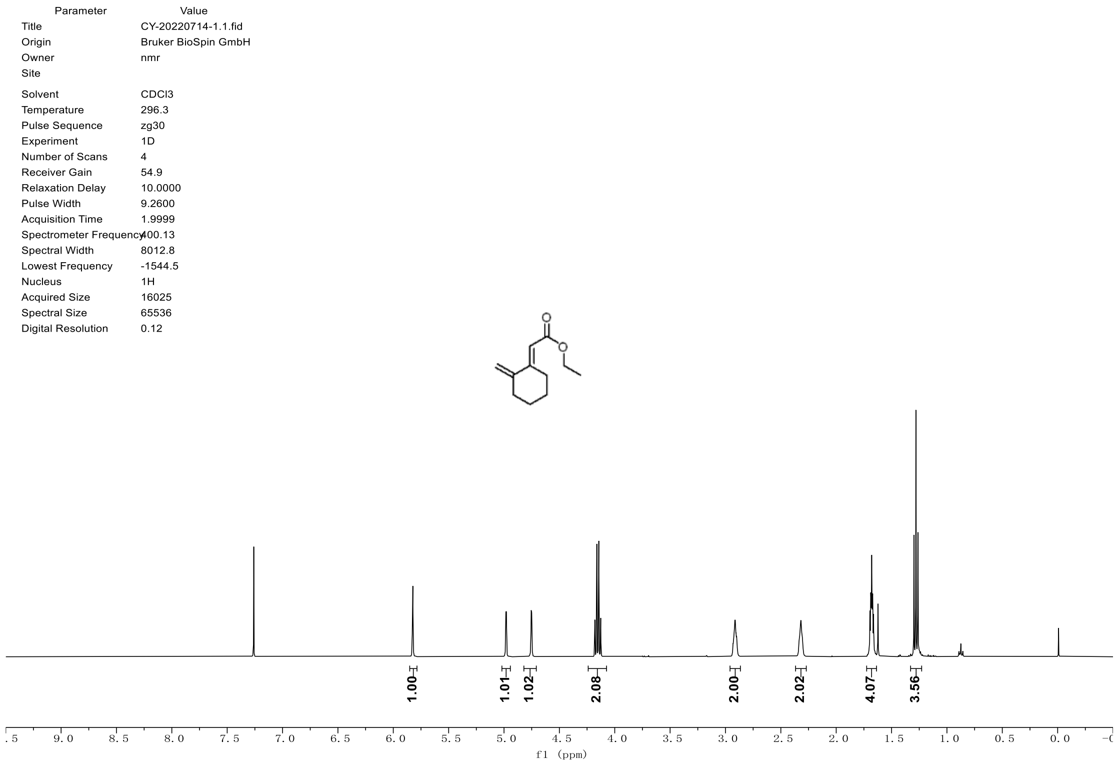

Supplementary Figure 97. ^{1}H NMR of **1′x**

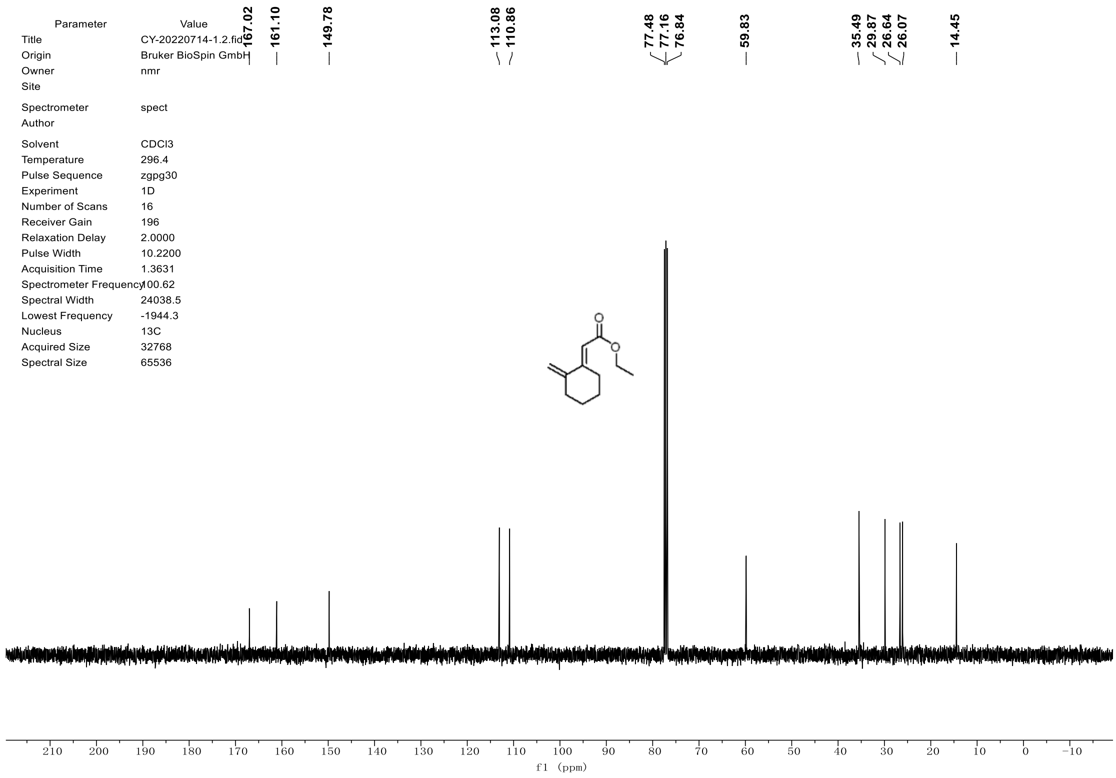

Supplementary Figure 98. ^{13}C NMR of **1′x**

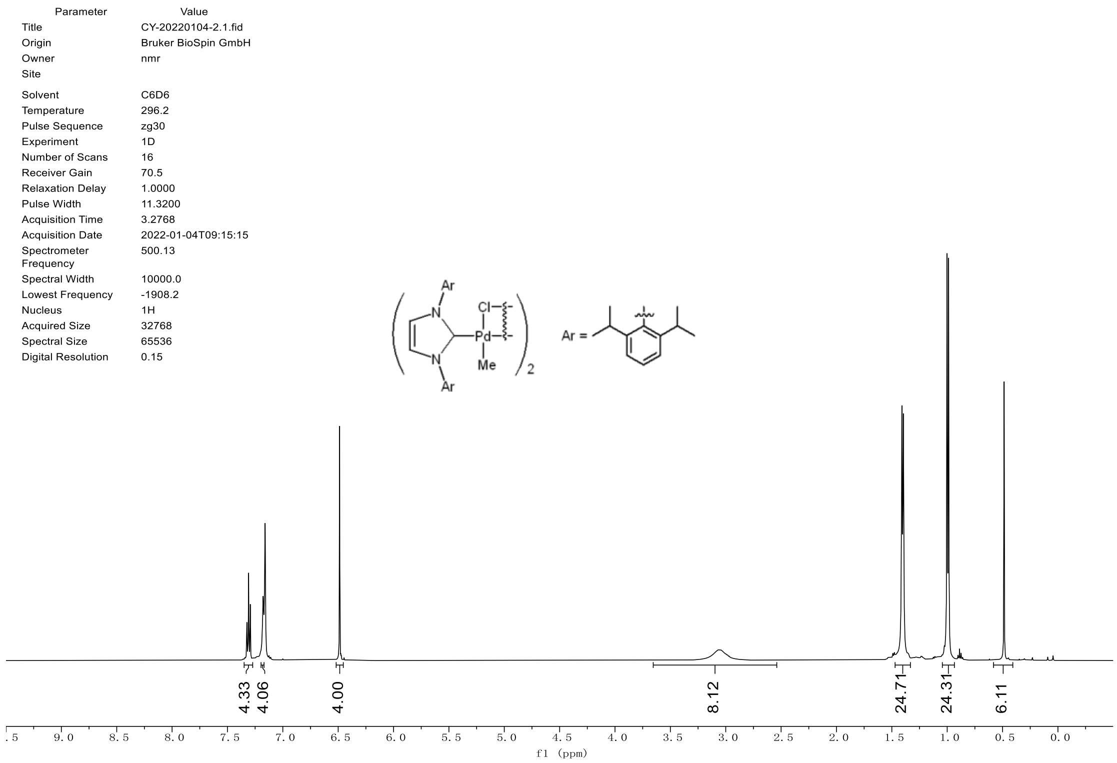

Supplementary Figure 99. ^{1}H NMR of [(**L1**)Pd(Me)Cl]$_2$

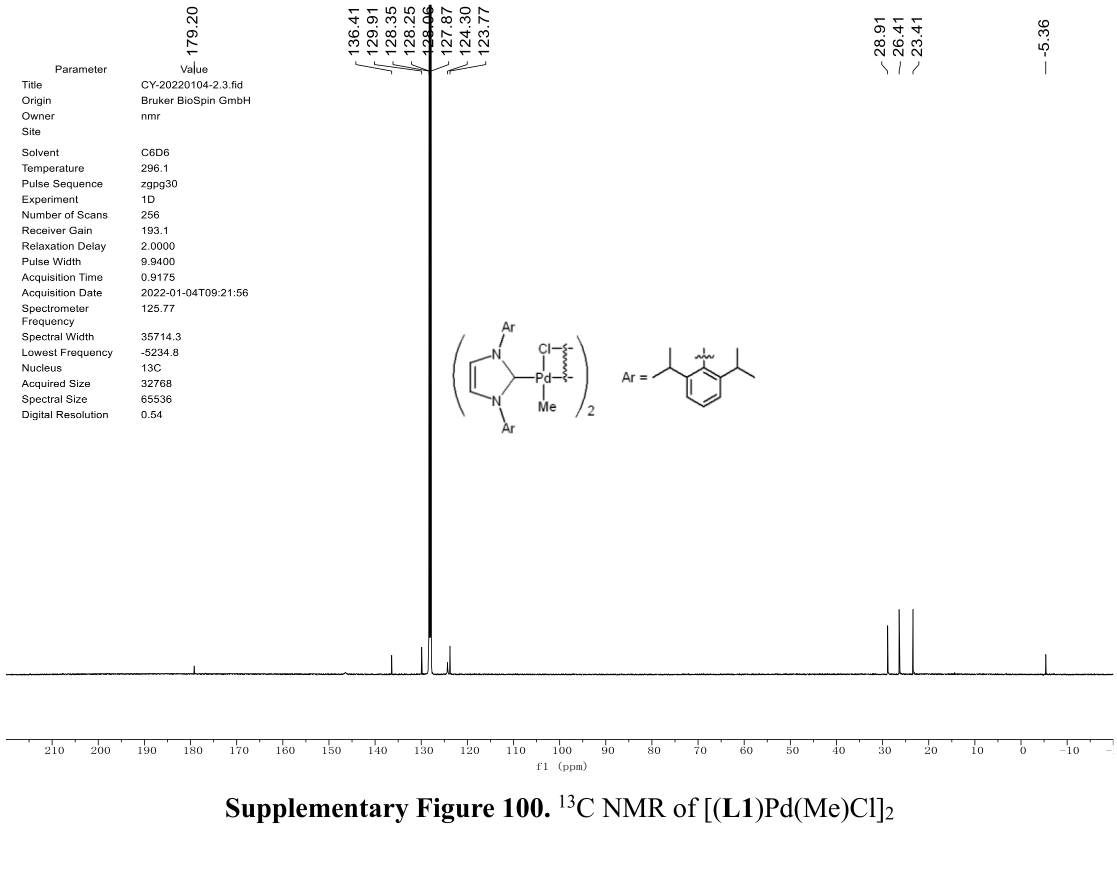

Supplementary Figure 100. ^{13}C NMR of [(**L1**)Pd(Me)Cl]$_2$

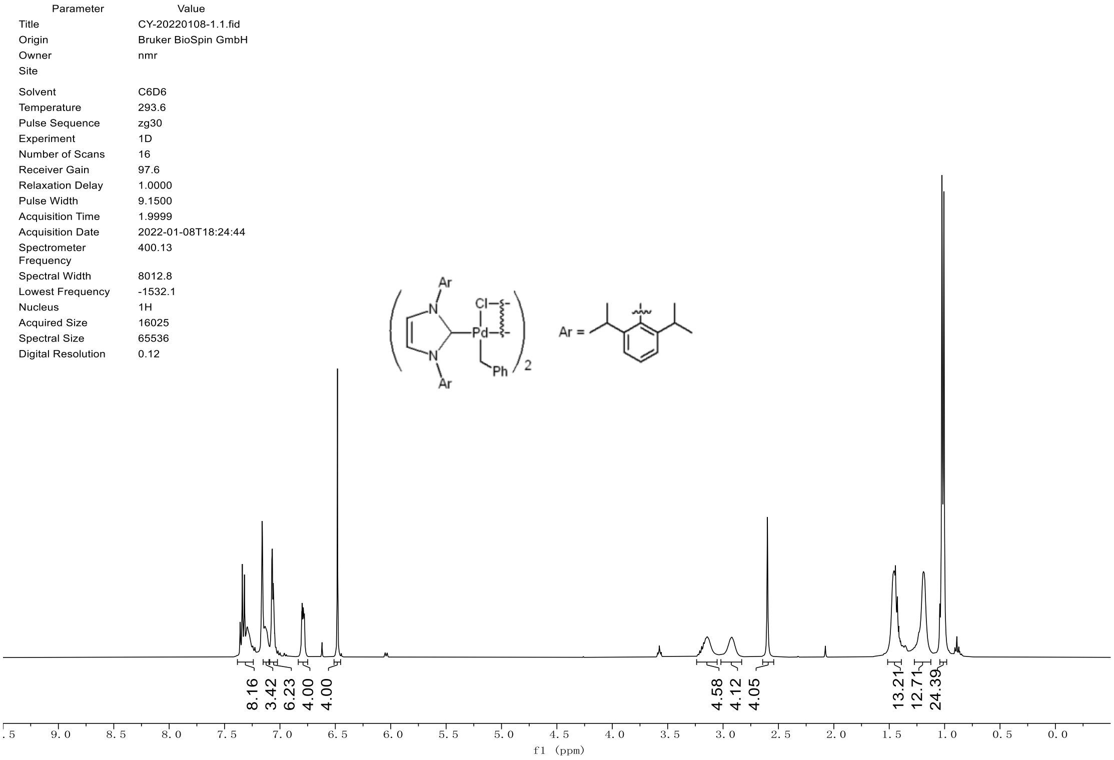

Supplementary Figure 101. ^{1}H NMR of [(**L1**)Pd(Bn)Cl]$_2$

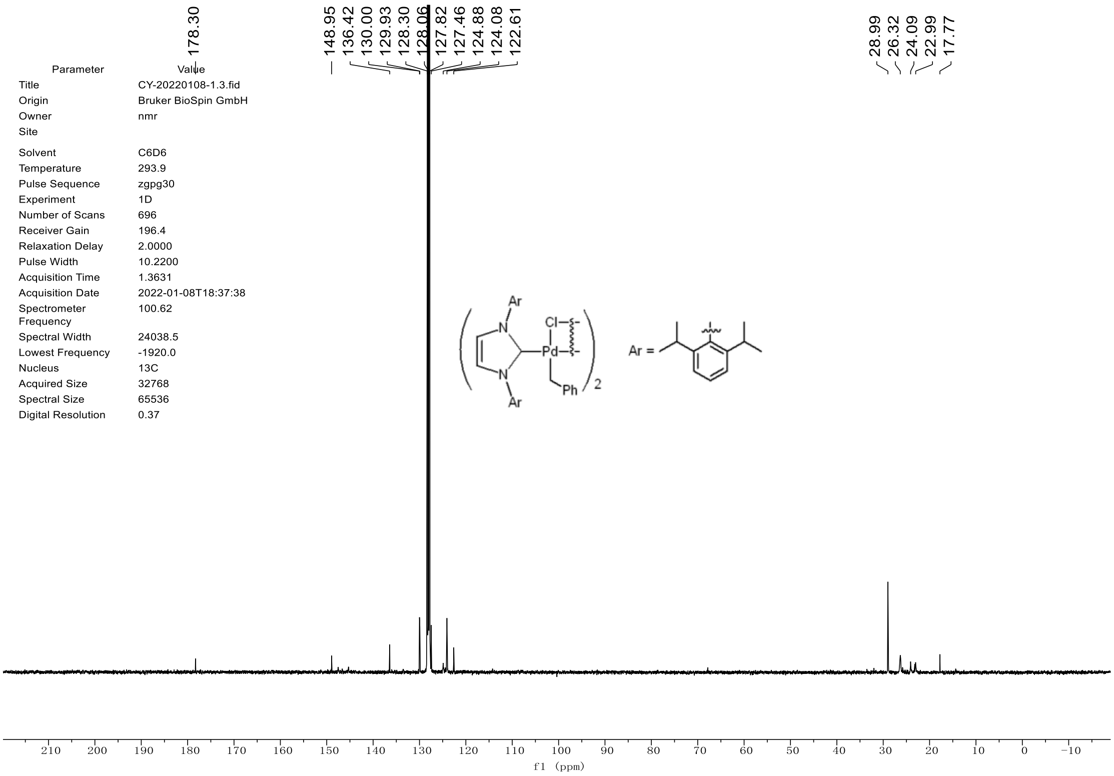

Supplementary Figure 102. ^{13}C NMR of [(**L1**)Pd(Bn)Cl]$_2$

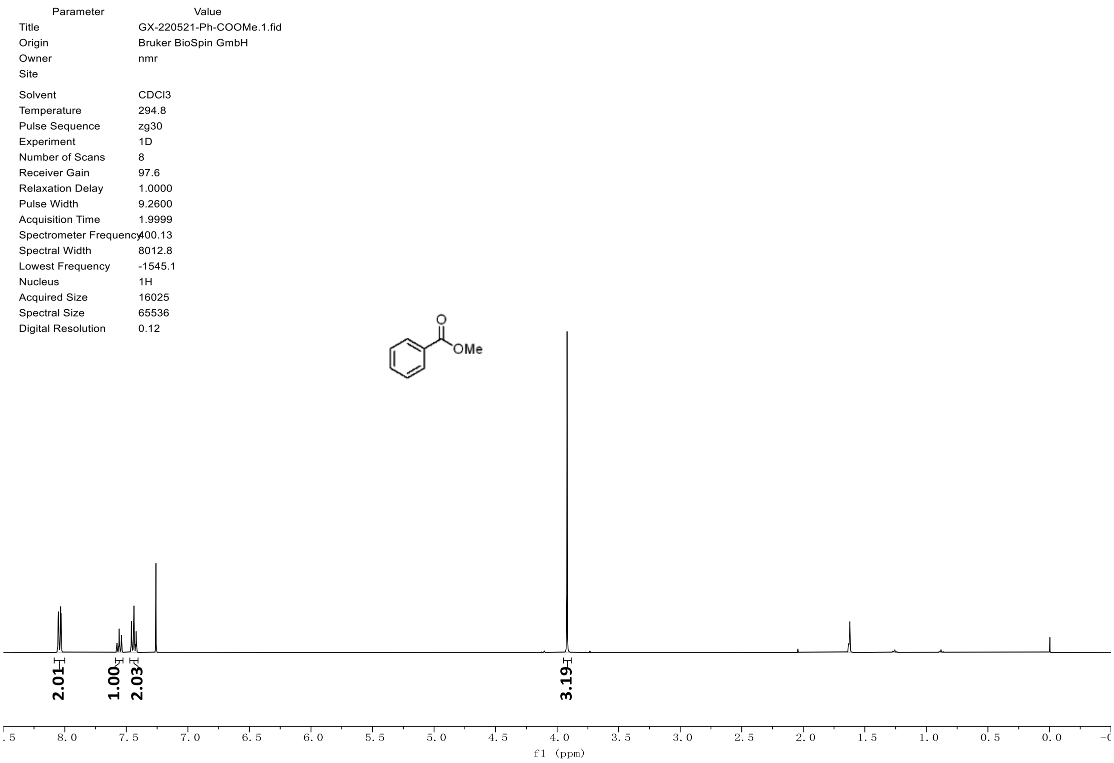

Supplementary Figure 103. Purified ^{1}H NMR of **2a**

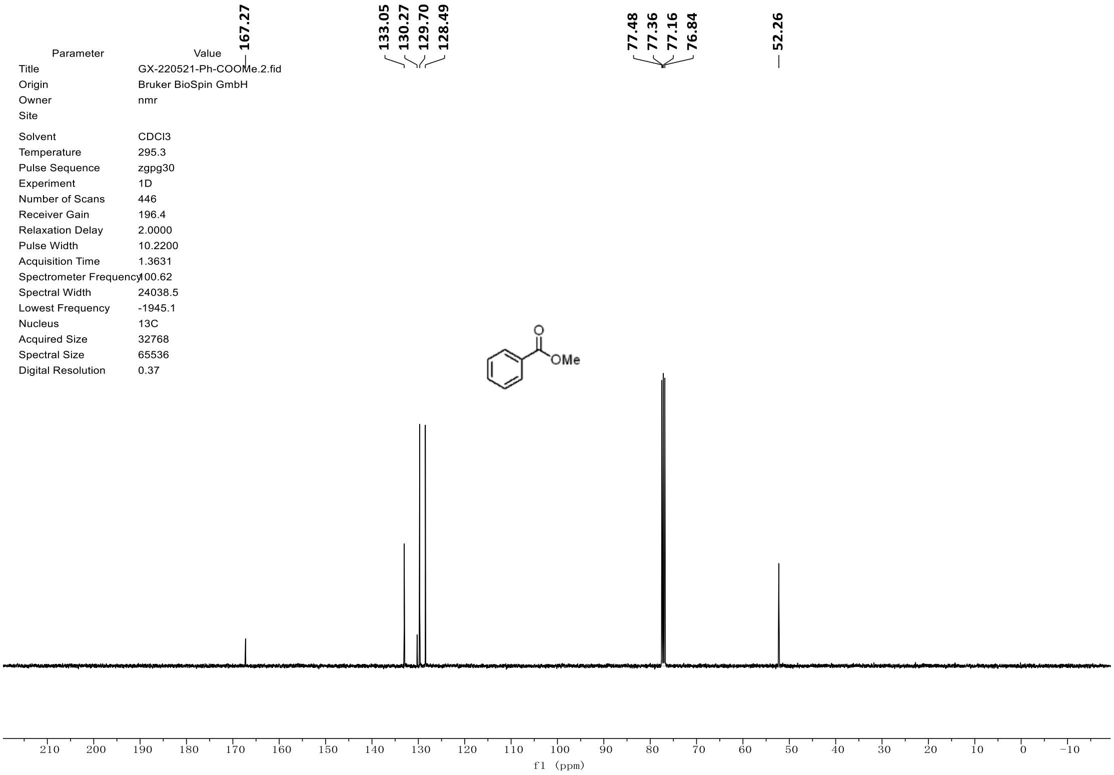

Supplementary Figure 104. Purified ^{13}C NMR of **2a**

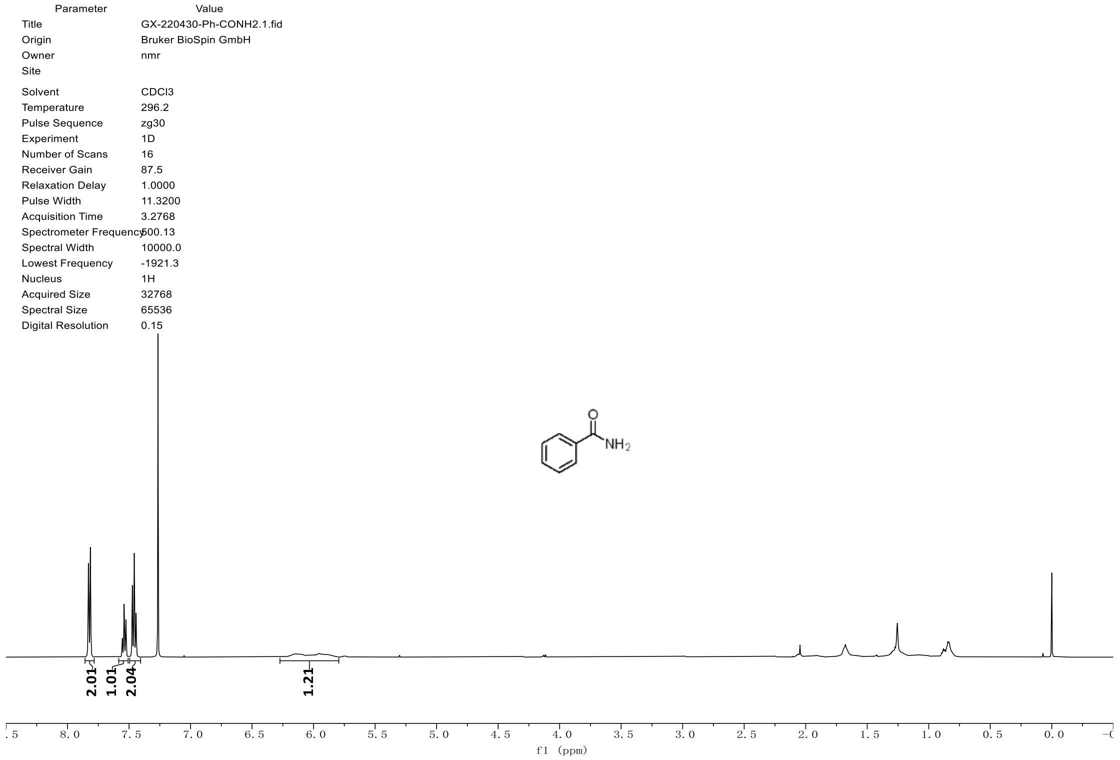

Supplementary Figure 105. Purified ^{1}H NMR of **2b**

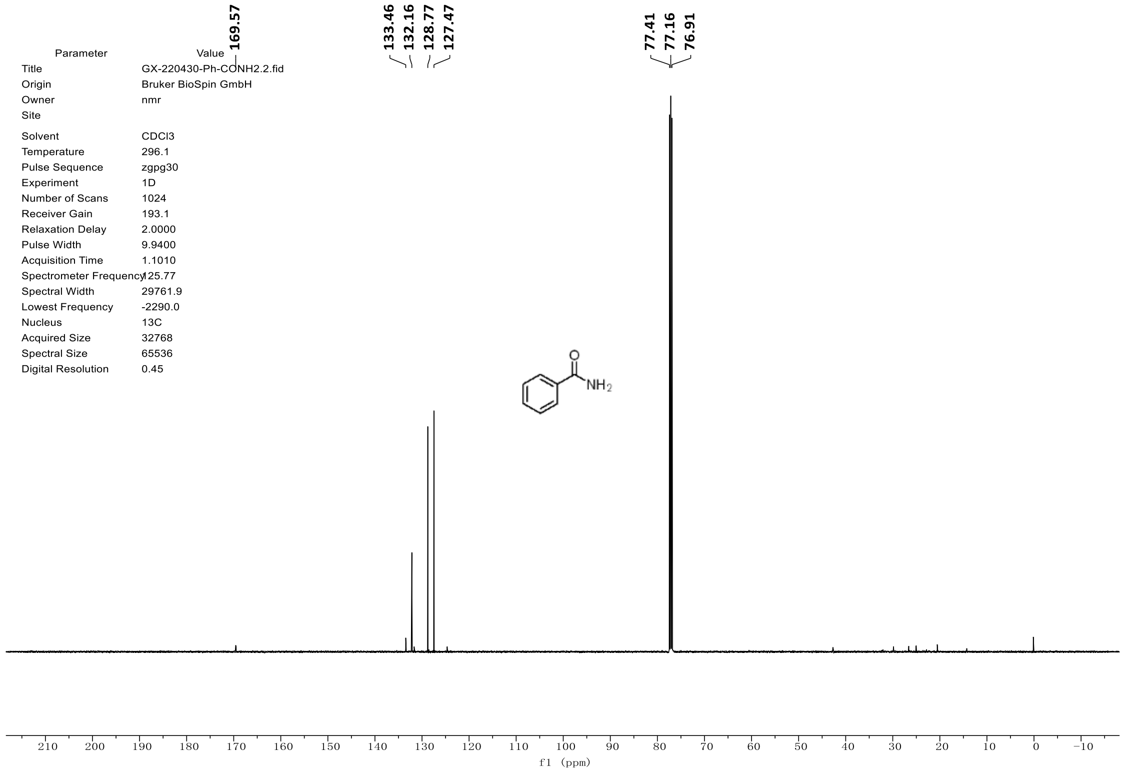

Supplementary Figure 106. Purified ^{13}C NMR of **2b**

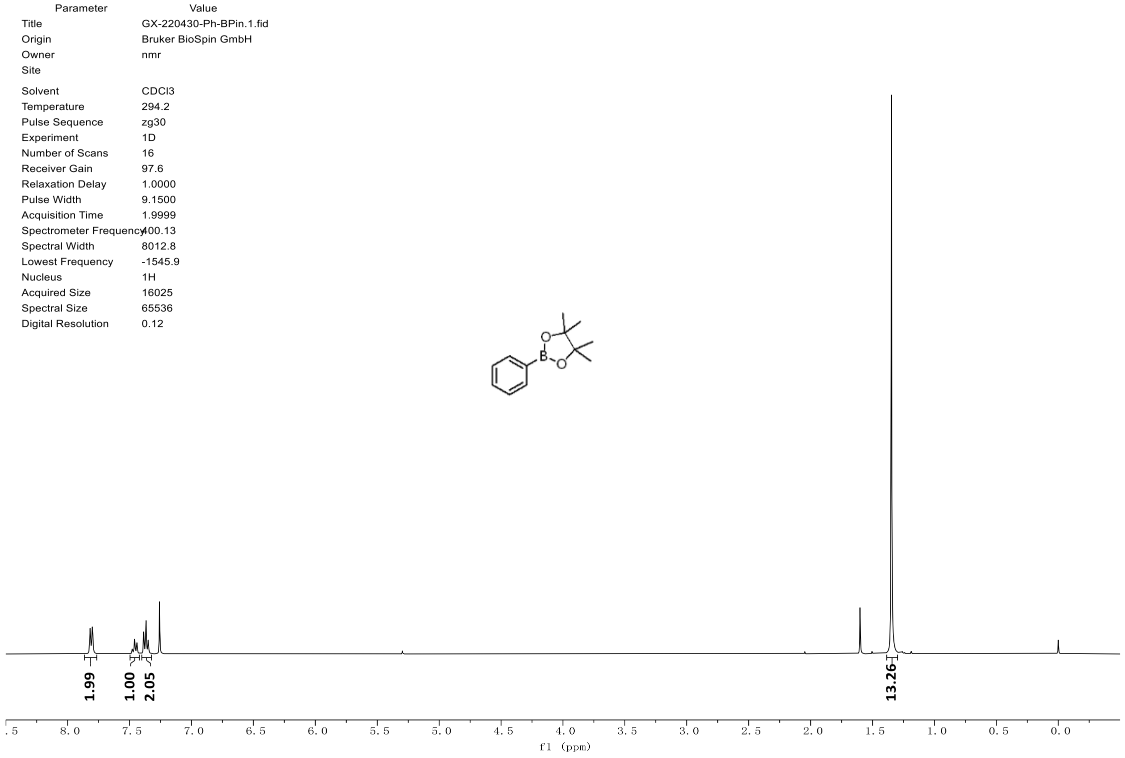

Supplementary Figure 107. Purified ^{1}H NMR of **2c**

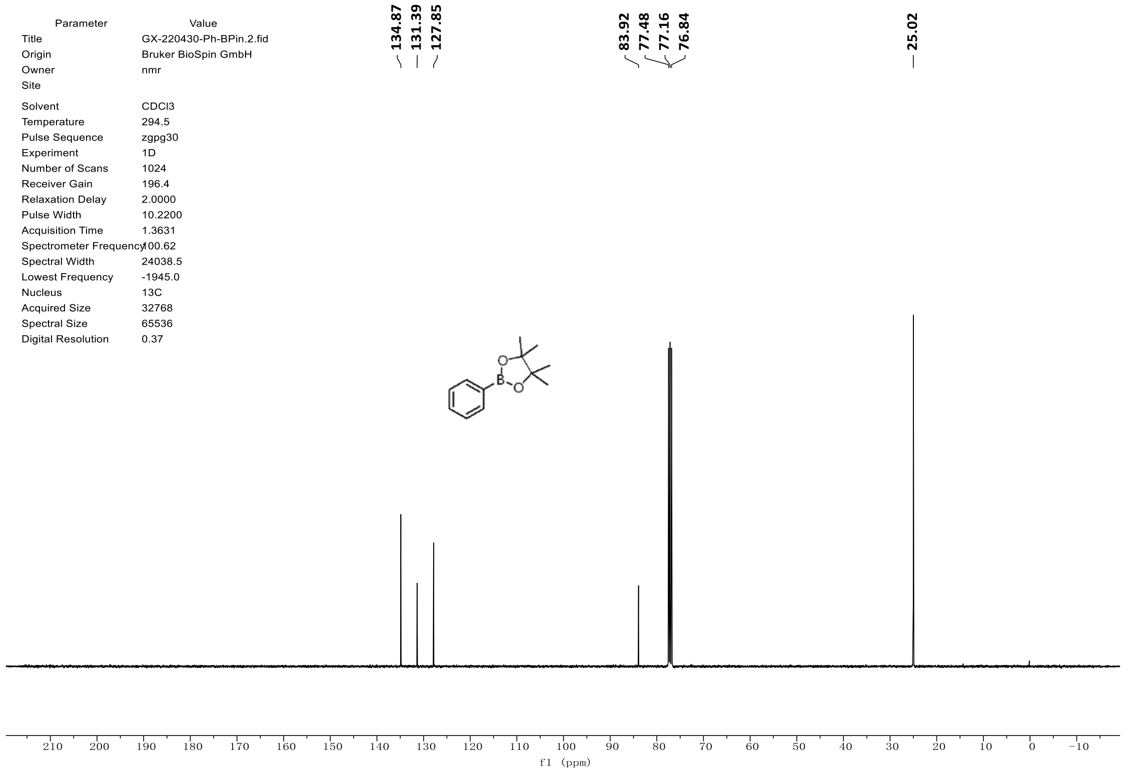

Supplementary Figure 108. Purified ^{13}C NMR of **2c**

Parameter	Value
Title	GX-220430-phenol.1.fid
Origin	Bruker BioSpin GmbH
Owner	nmr
Site	
Solvent	CDCl3
Temperature	294.3
Pulse Sequence	zg30
Experiment	1D
Number of Scans	16
Receiver Gain	97.6
Relaxation Delay	1.0000
Pulse Width	9.1500
Acquisition Time	1.9999
Spectrometer Frequency	400.13
Spectral Width	8012.8
Lowest Frequency	-1549.4
Nucleus	1H
Acquired Size	16025
Spectral Size	65536
Digital Resolution	0.12

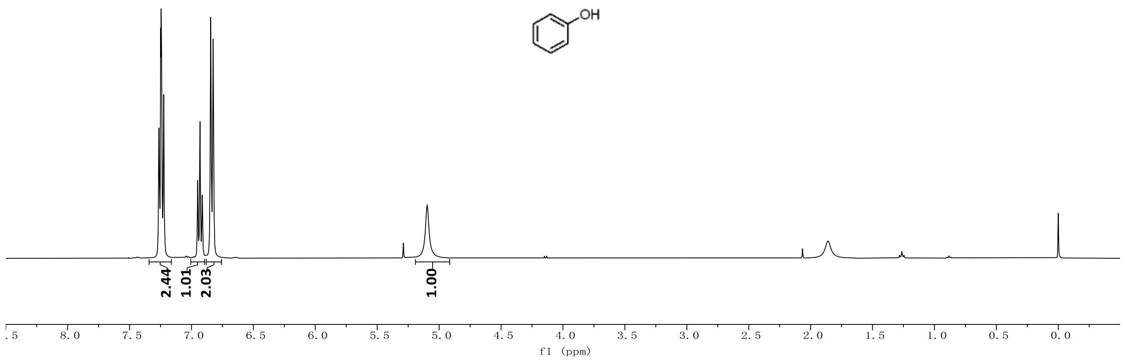

Supplementary Figure 109. Purified ^{1}H NMR of **2e**

Parameter	Value
Title	GX-220430-phenol.2.fid
Origin	Bruker BioSpin GmbH
Owner	nmr
Site	
Solvent	CDCl3
Temperature	294.6
Pulse Sequence	zgpg30
Experiment	1D
Number of Scans	1024
Receiver Gain	196.4
Relaxation Delay	2.0000
Pulse Width	10.2200
Acquisition Time	1.3631
Spectrometer Frequency	100.62
Spectral Width	24038.5
Lowest Frequency	-1946.9
Nucleus	13C
Acquired Size	32768
Spectral Size	65536
Digital Resolution	0.37

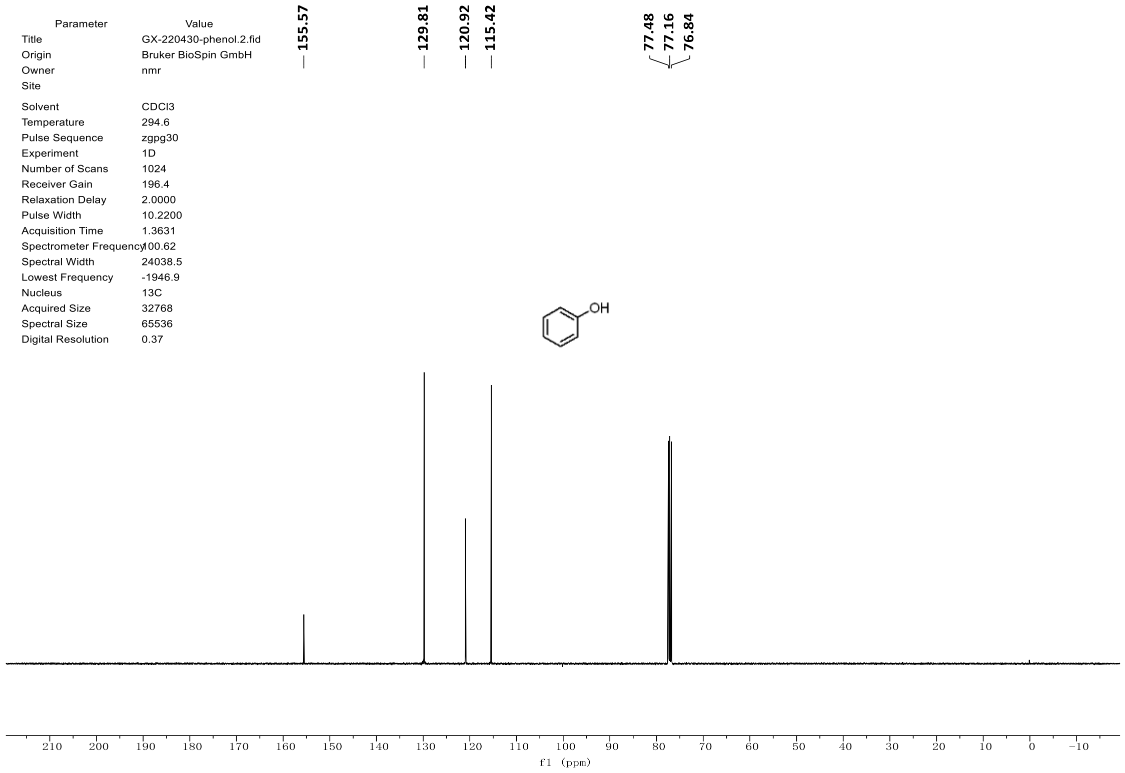

Supplementary Figure 110. Purified ^{13}C NMR of **2e**

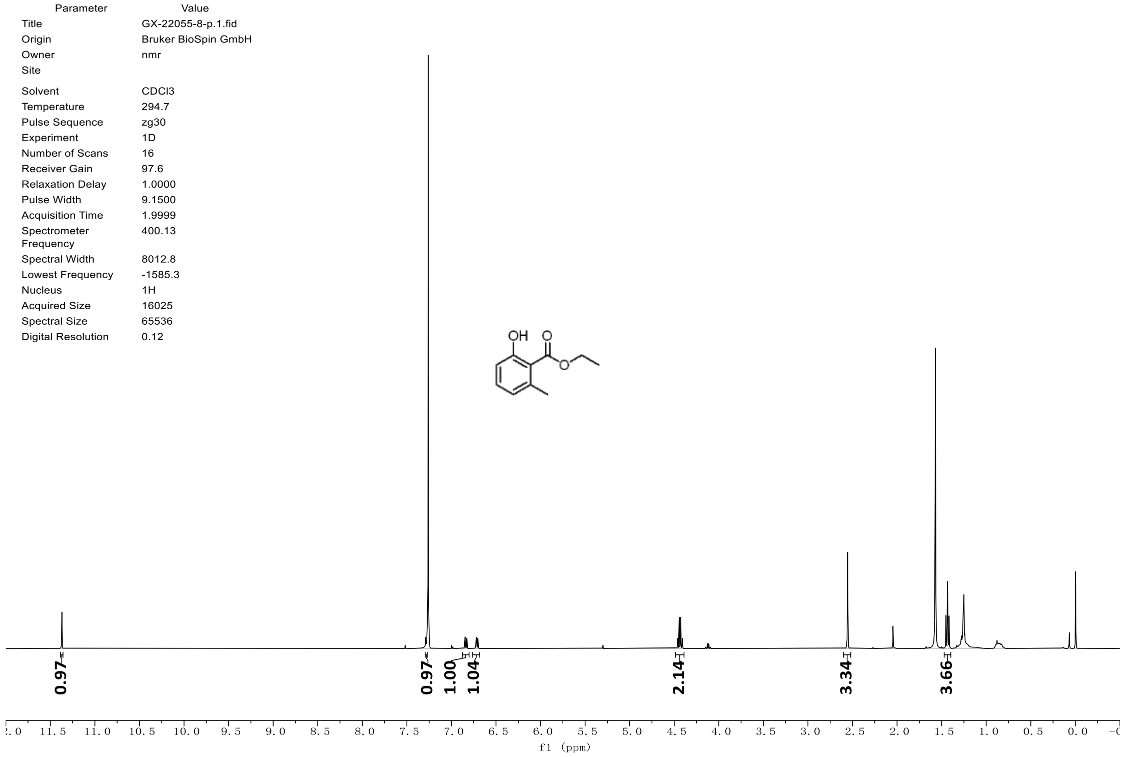

Supplementary Figure 111. Purified ^{1}H NMR of **2g**

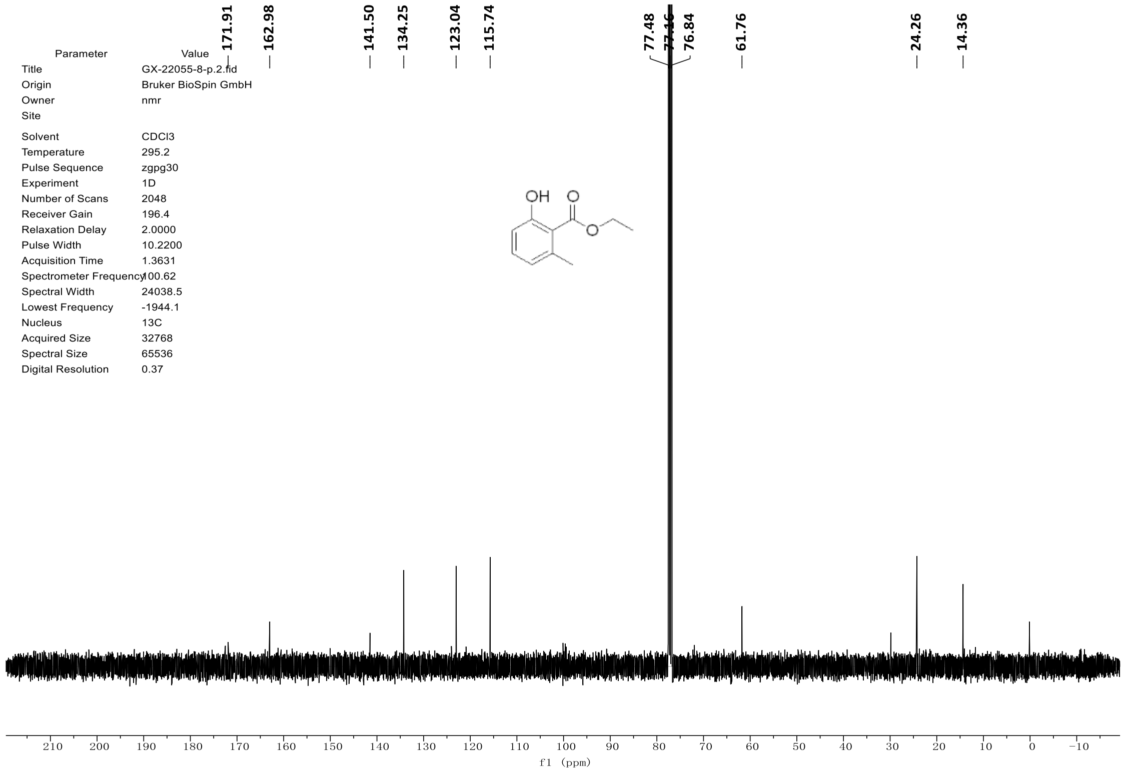

Supplementary Figure 112. Purified ^{13}C NMR of **2g**

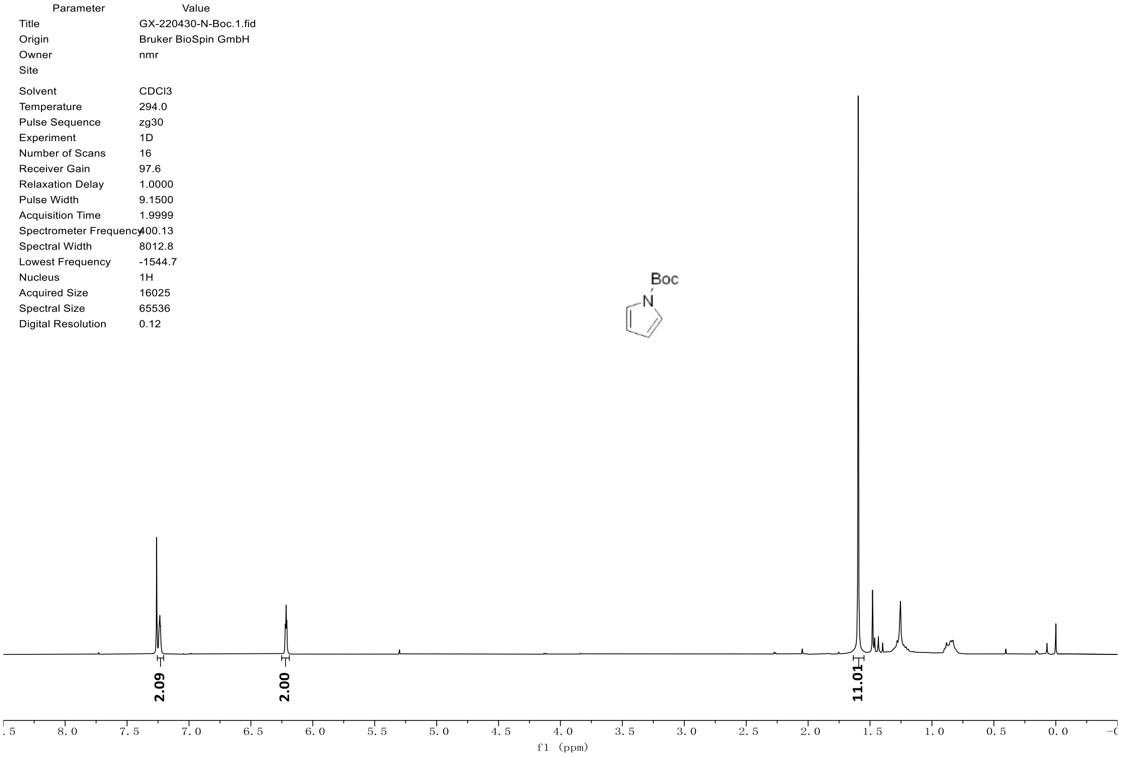

Supplementary Figure 113. Purified ^{1}H NMR of **2i**

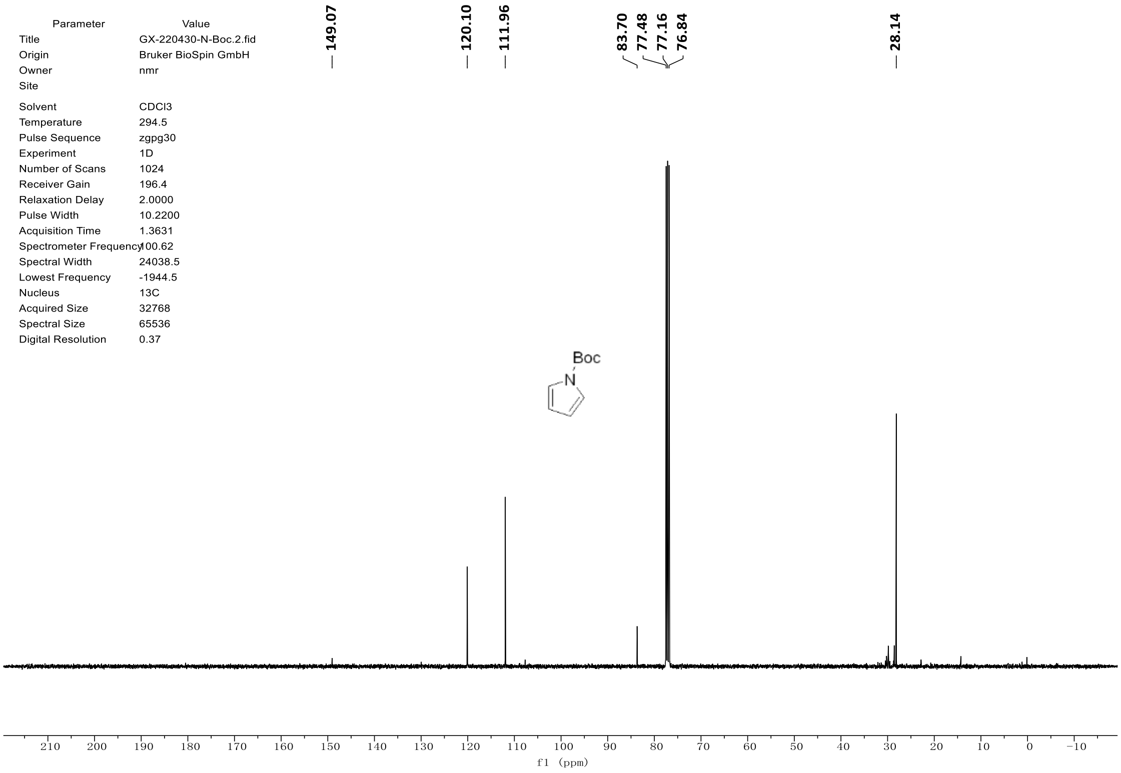

Supplementary Figure 114. Purified ^{13}C NMR of **2i**

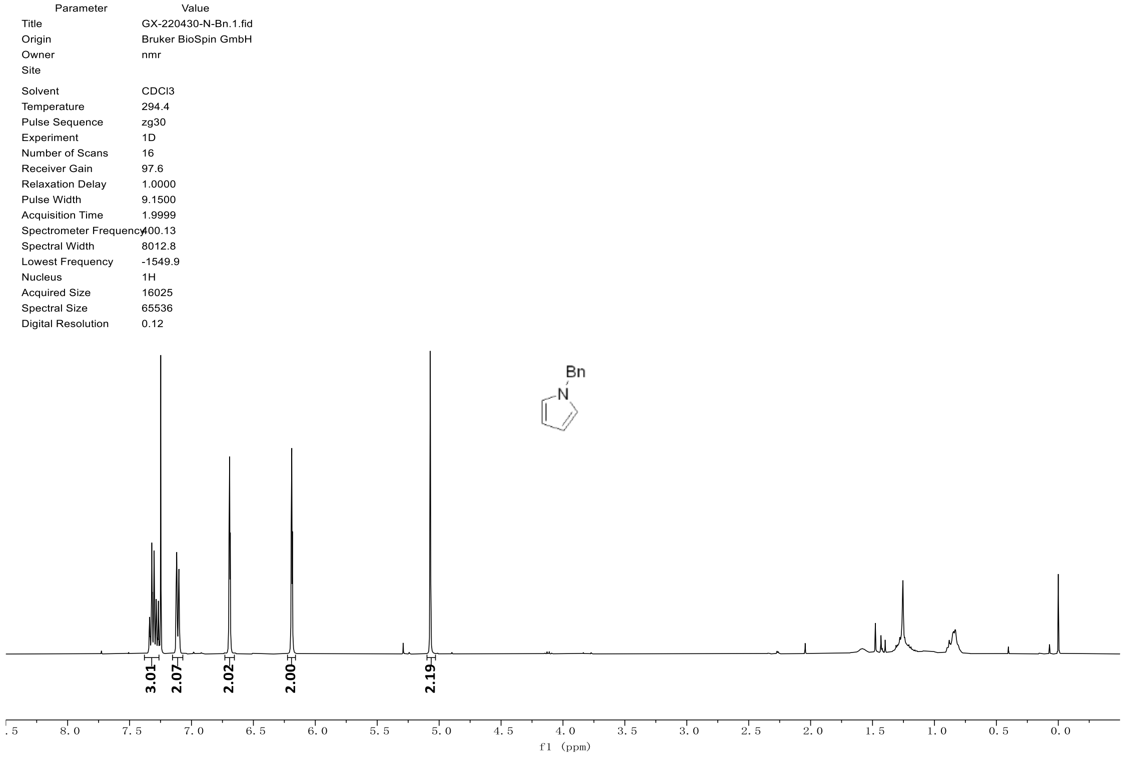

Supplementary Figure 115. Purified ^{1}H NMR of **2j**

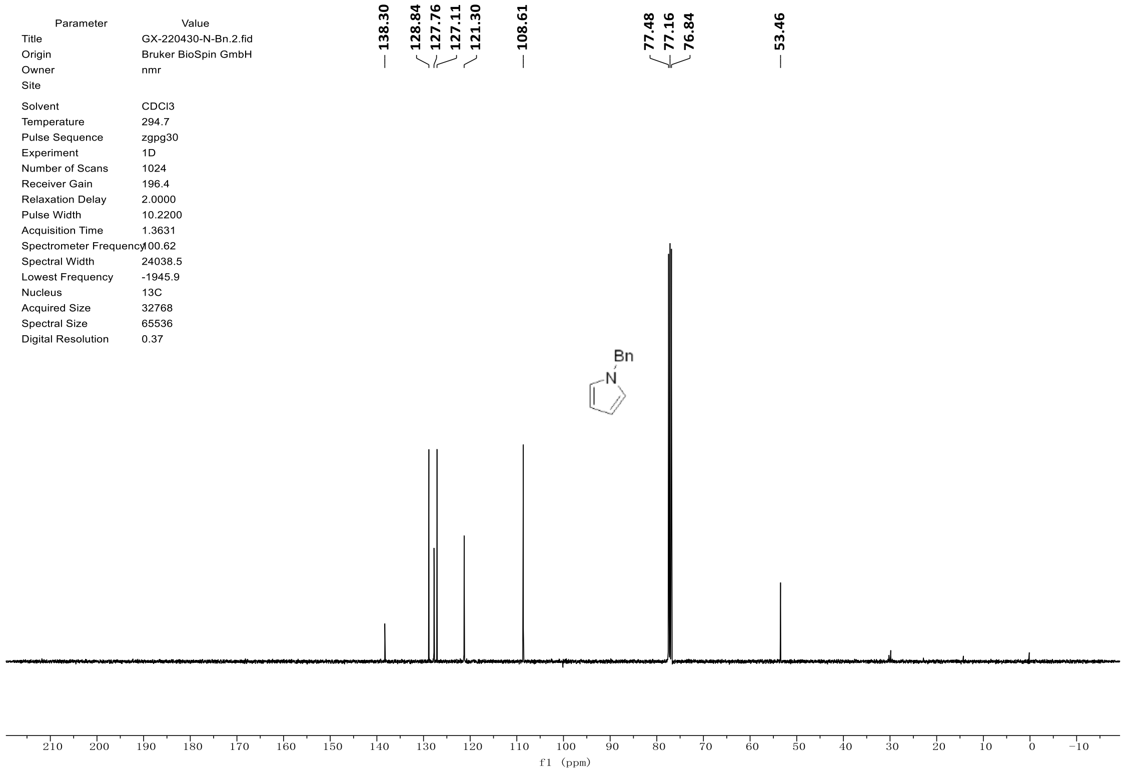

Supplementary Figure 116. Purified ^{13}C NMR of **2j**

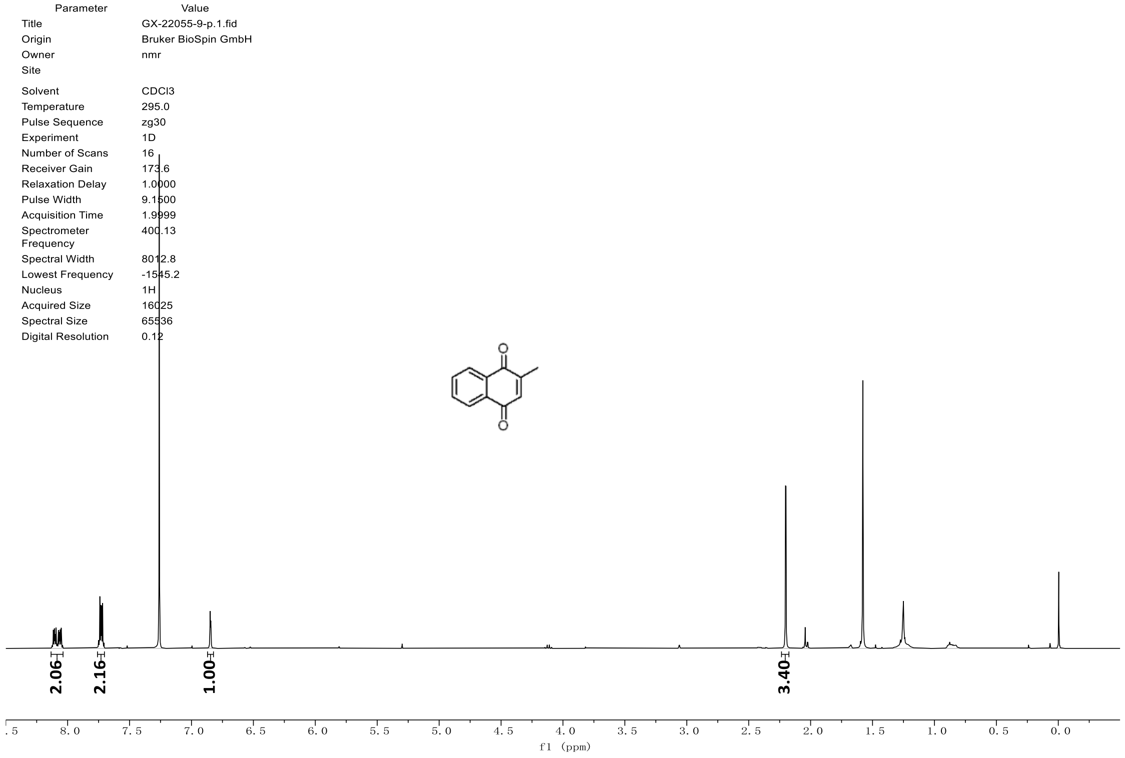

Supplementary Figure 117. Purified ^{1}H NMR of **2n**

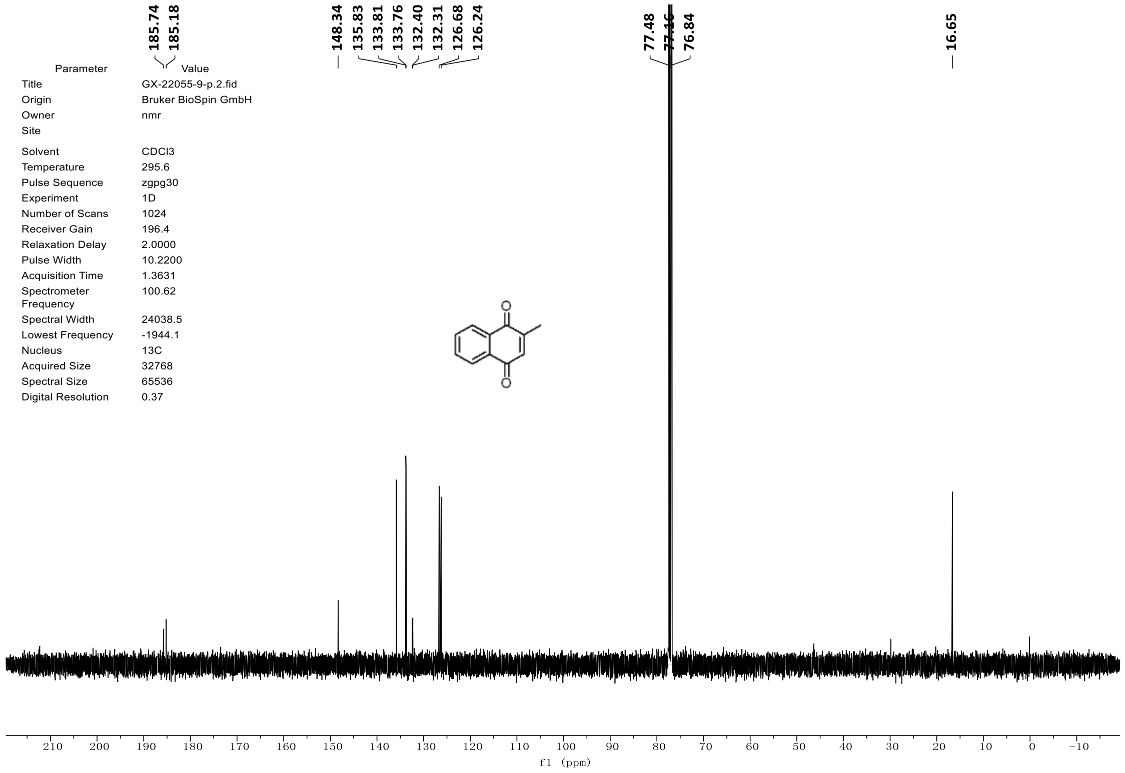

Supplementary Figure 118. Purified ^{13}C NMR of **2n**

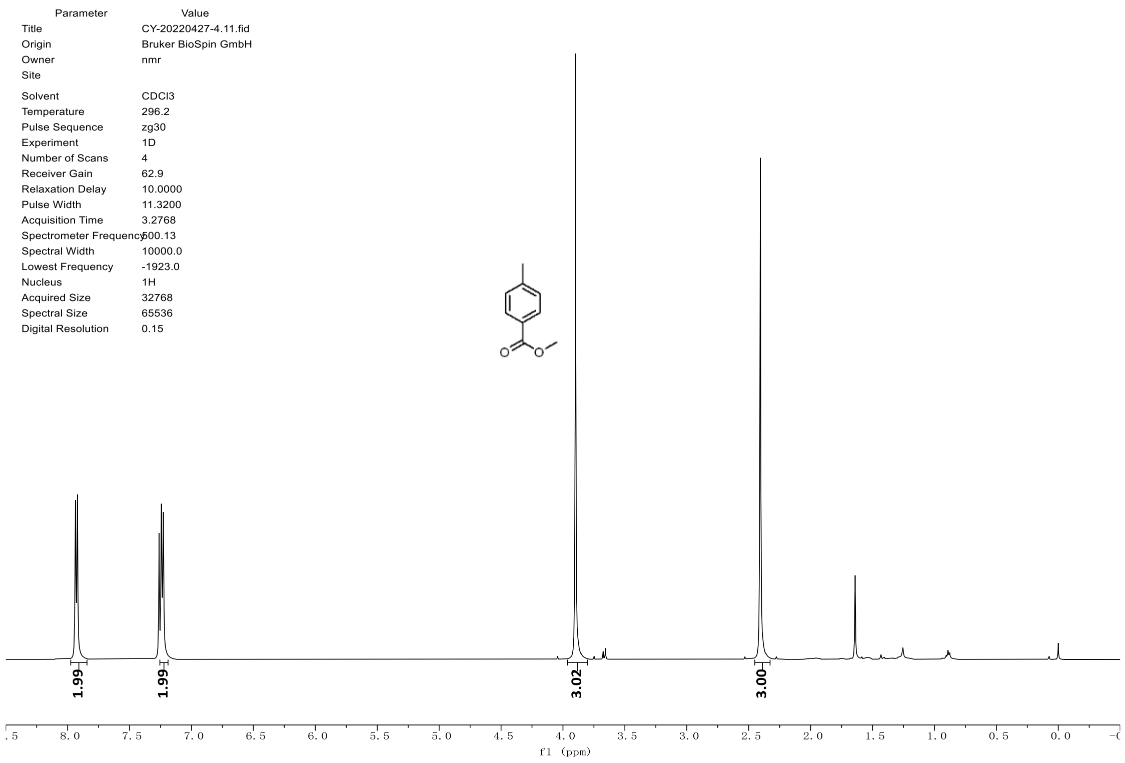

Supplementary Figure 119. Purified ^{1}H NMR of **2′b**

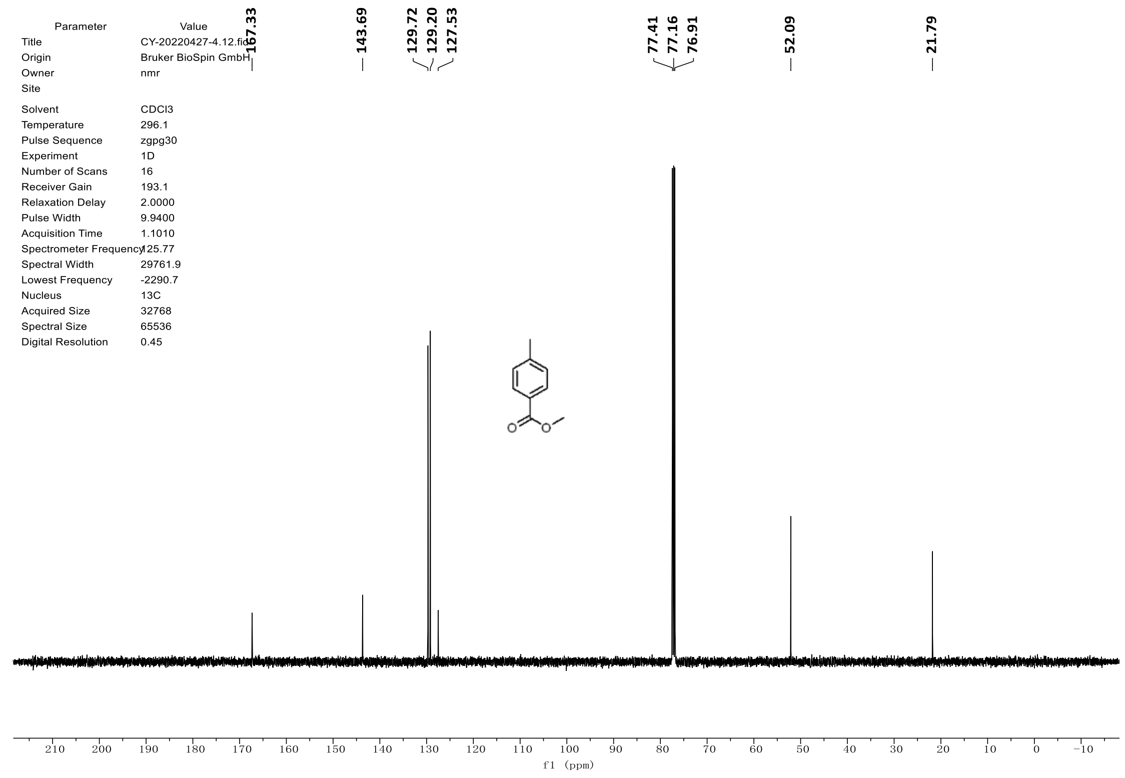

Supplementary Figure 120. Purified ^{13}C NMR of **2′b**

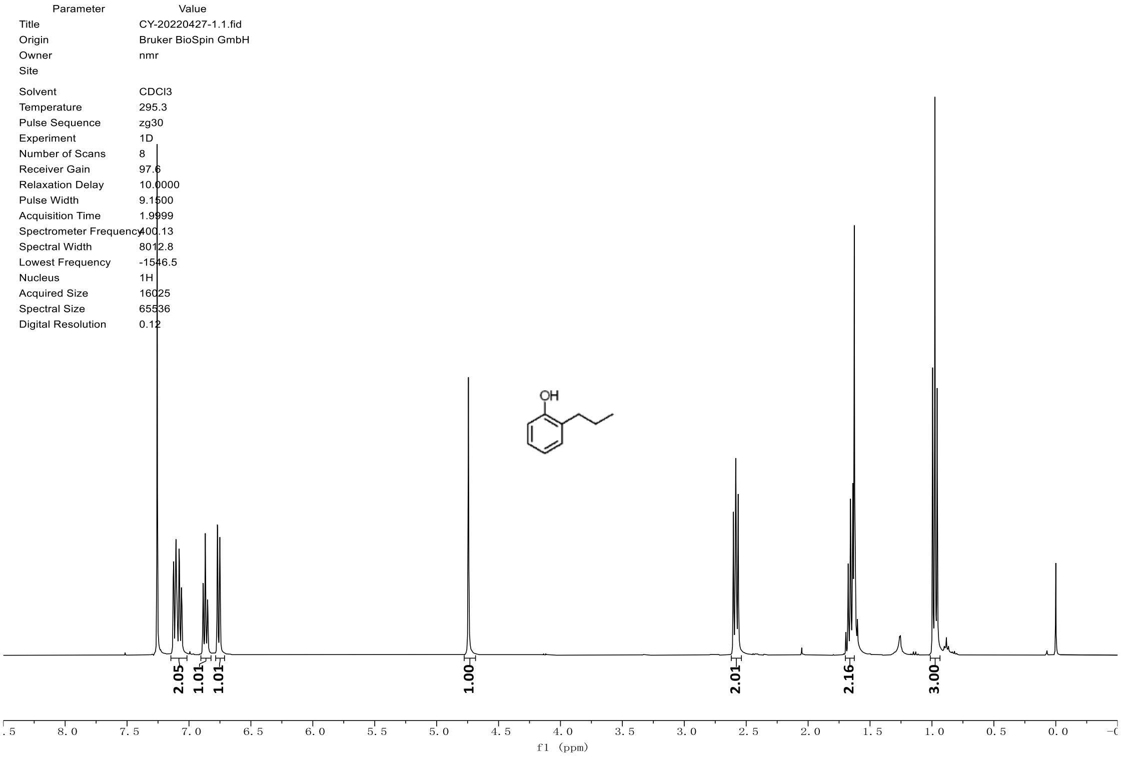

Supplementary Figure 121. Purified ^{1}H NMR of **2′i**

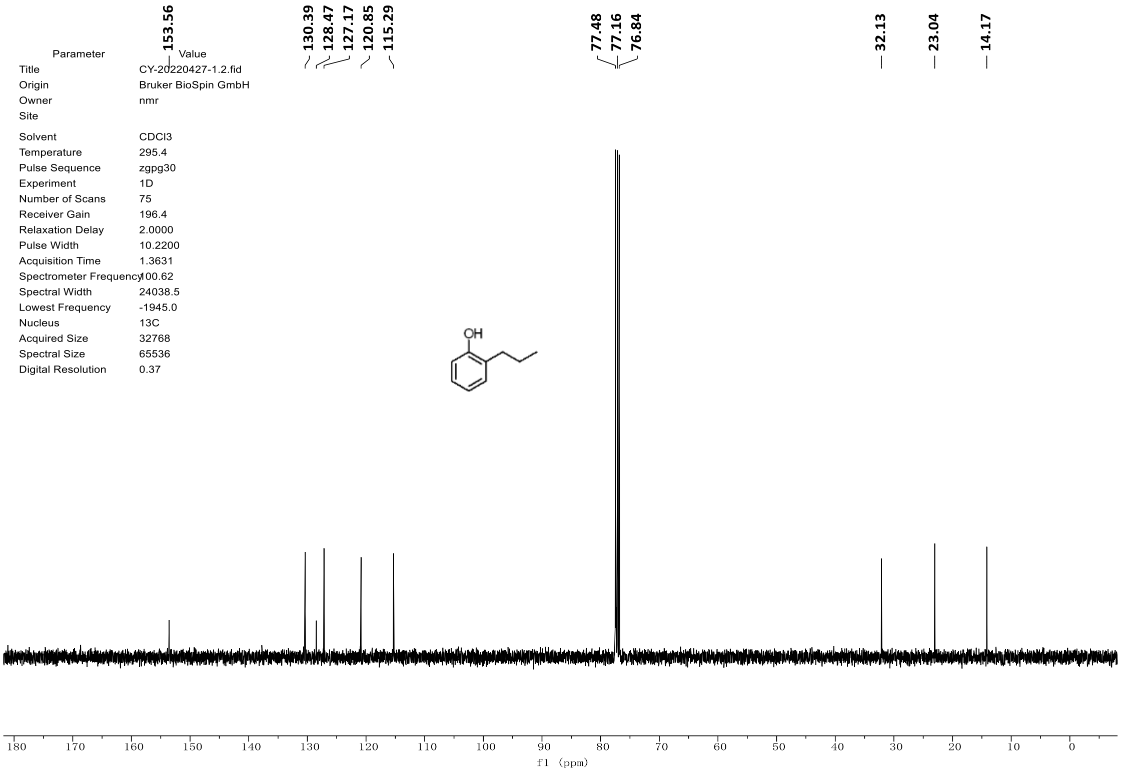

Supplementary Figure 122. Purified ^{13}C NMR of **2′i**

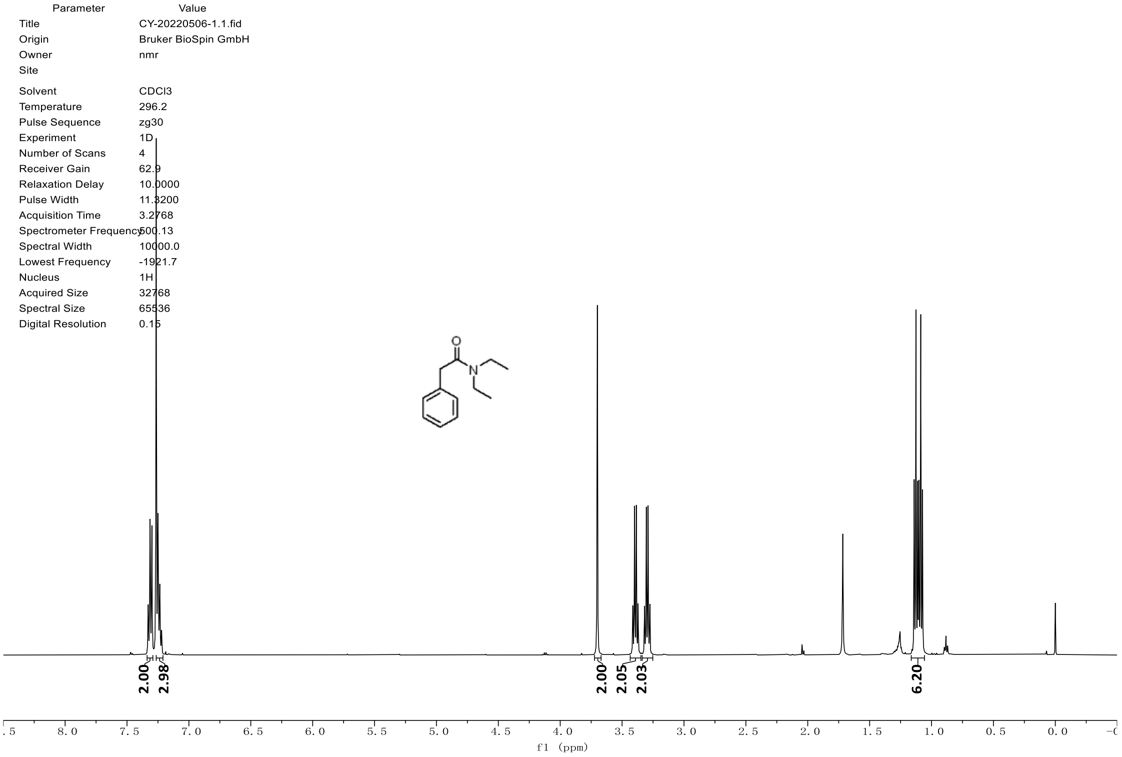

Supplementary Figure 123. Purified ^{1}H NMR of **2′m**

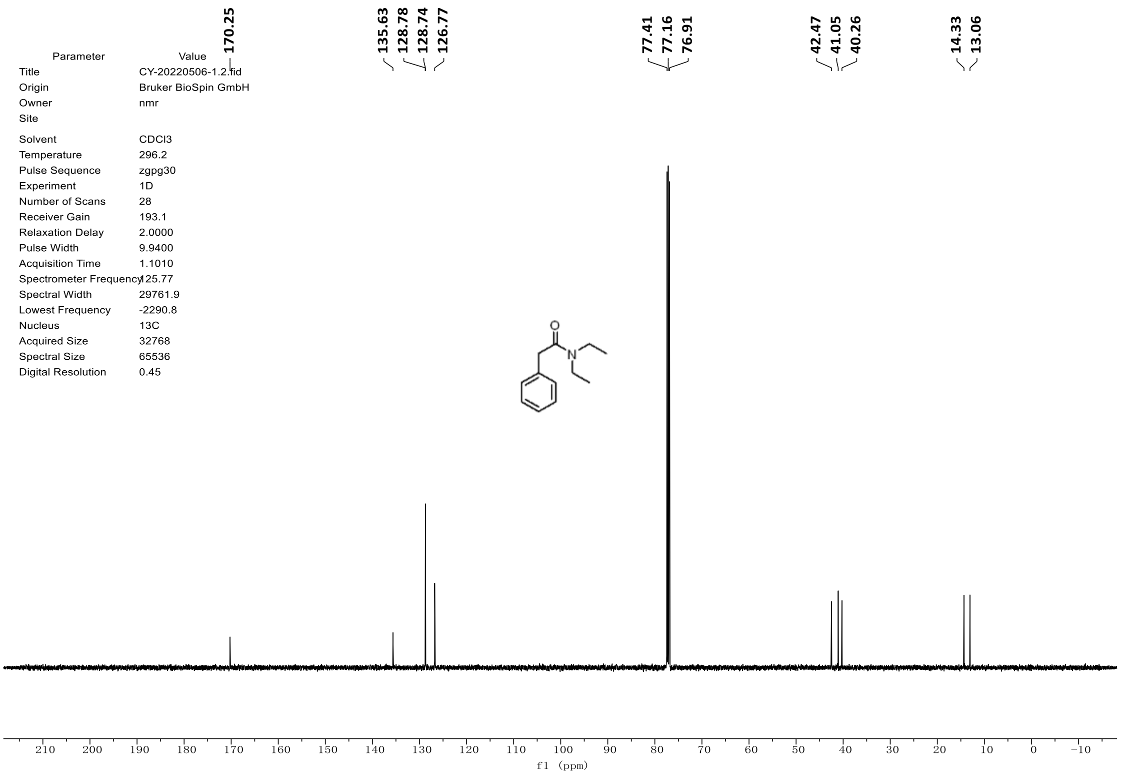

Supplementary Figure 124. Purified ^{13}C NMR of **2′m**

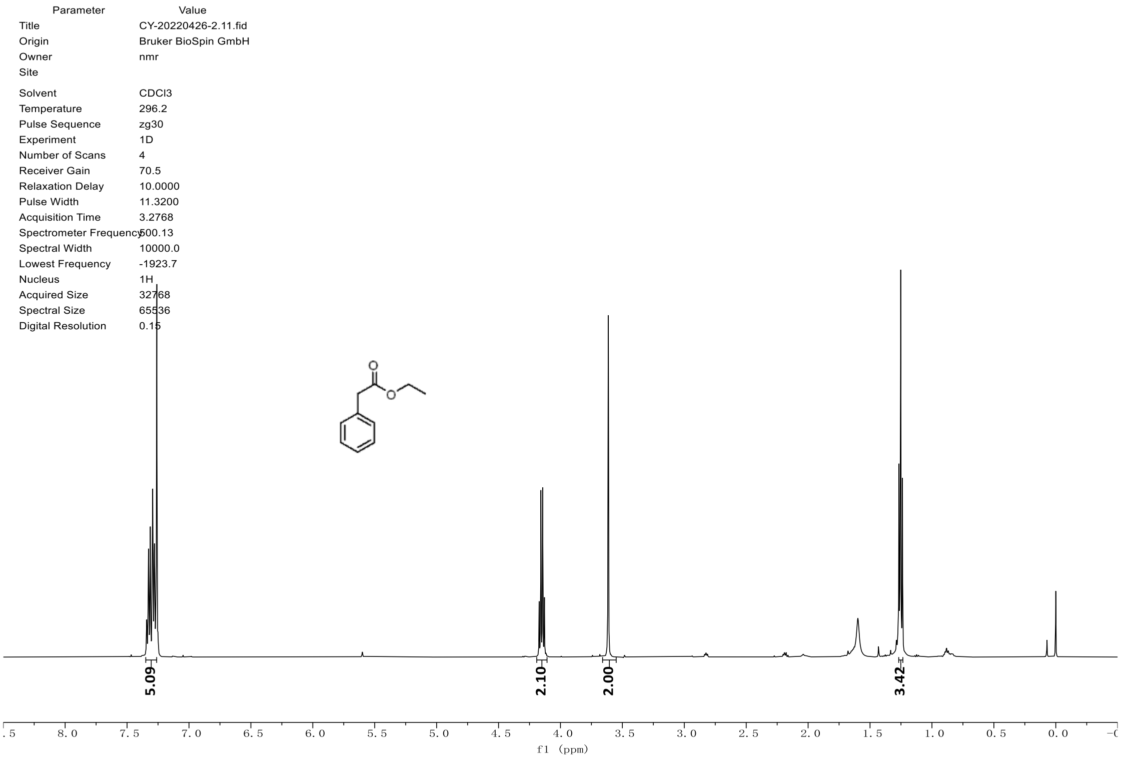

Supplementary Figure 125. Purified ^{1}H NMR of **2′o**

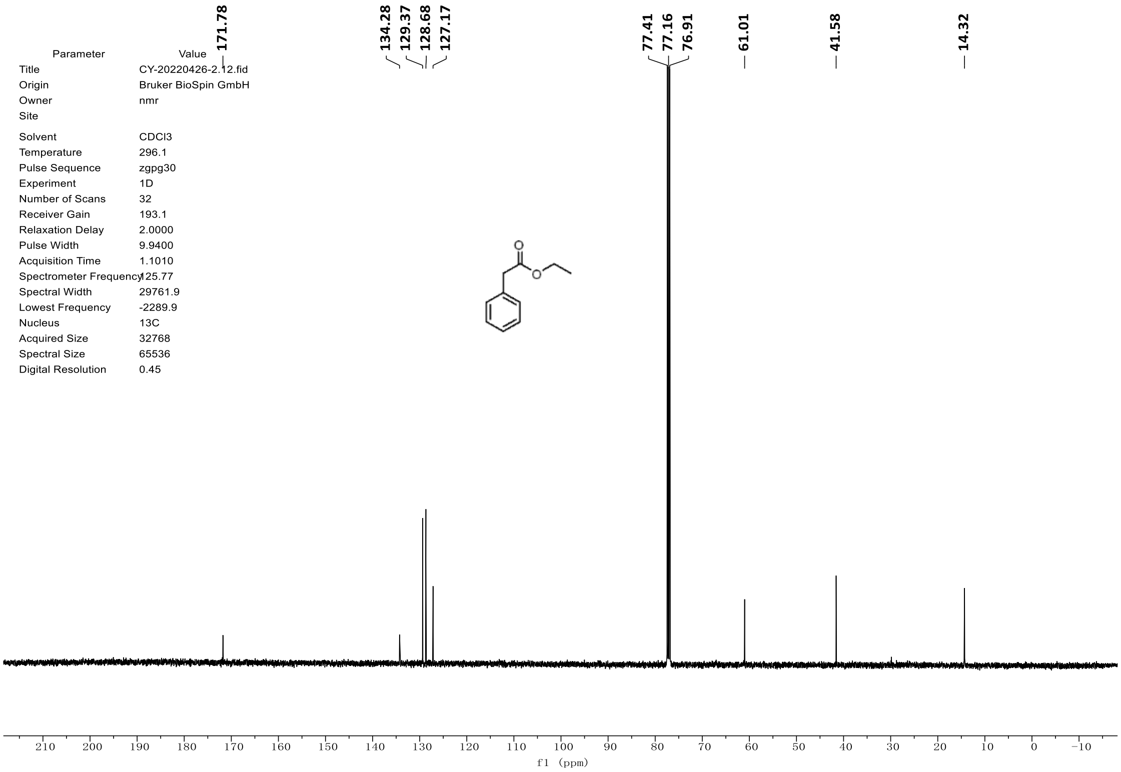

Supplementary Figure 126. Purified ^{13}C NMR of **2′o**

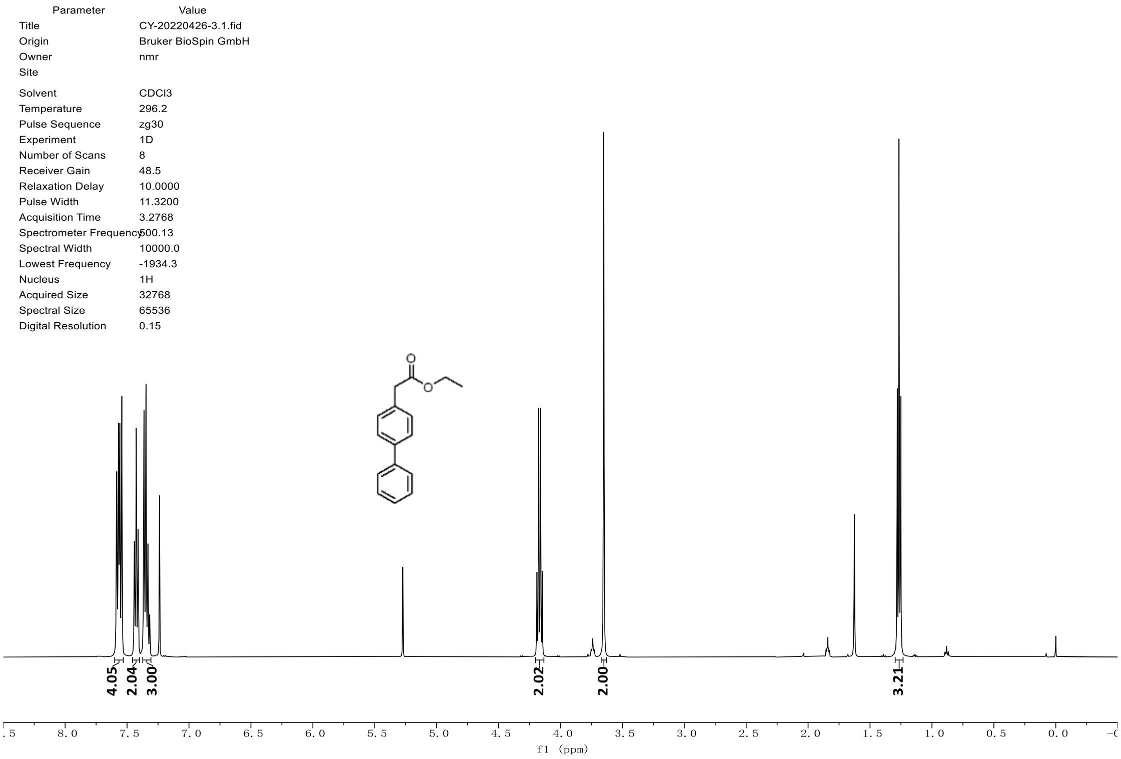

Supplementary Figure 127. Purified ^{1}H NMR of **2′p**

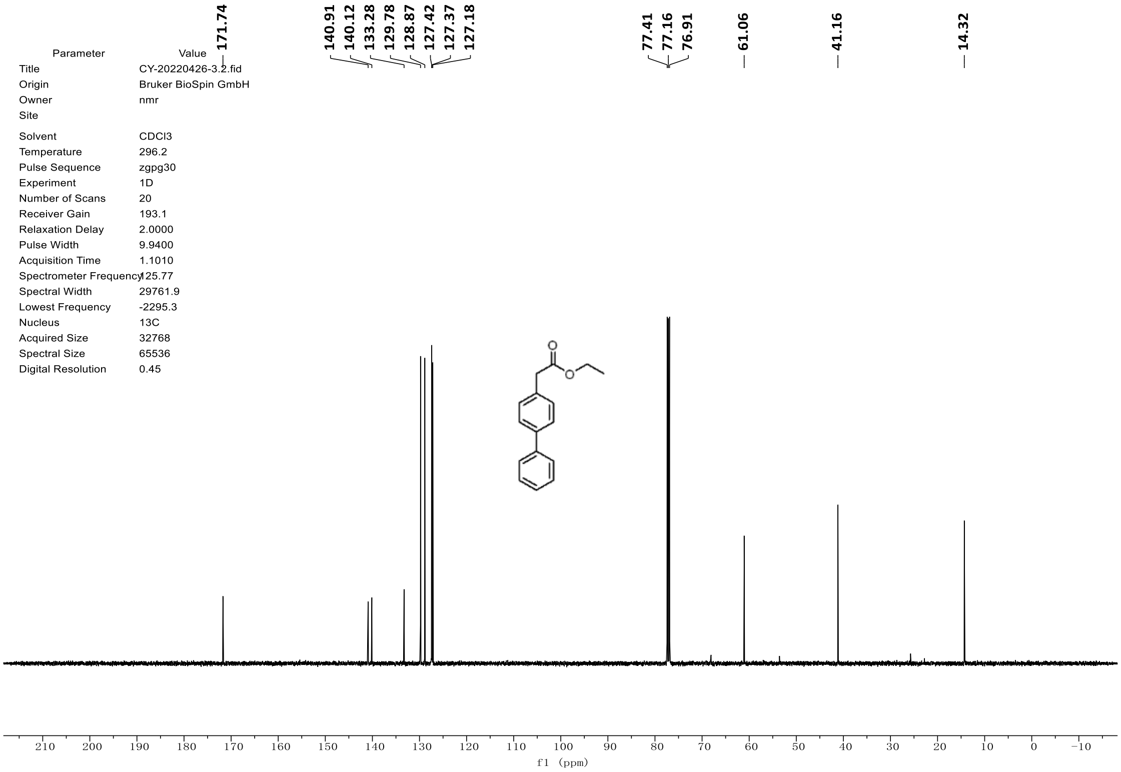

Supplementary Figure 128. Purified ^{13}C NMR of **2′p**

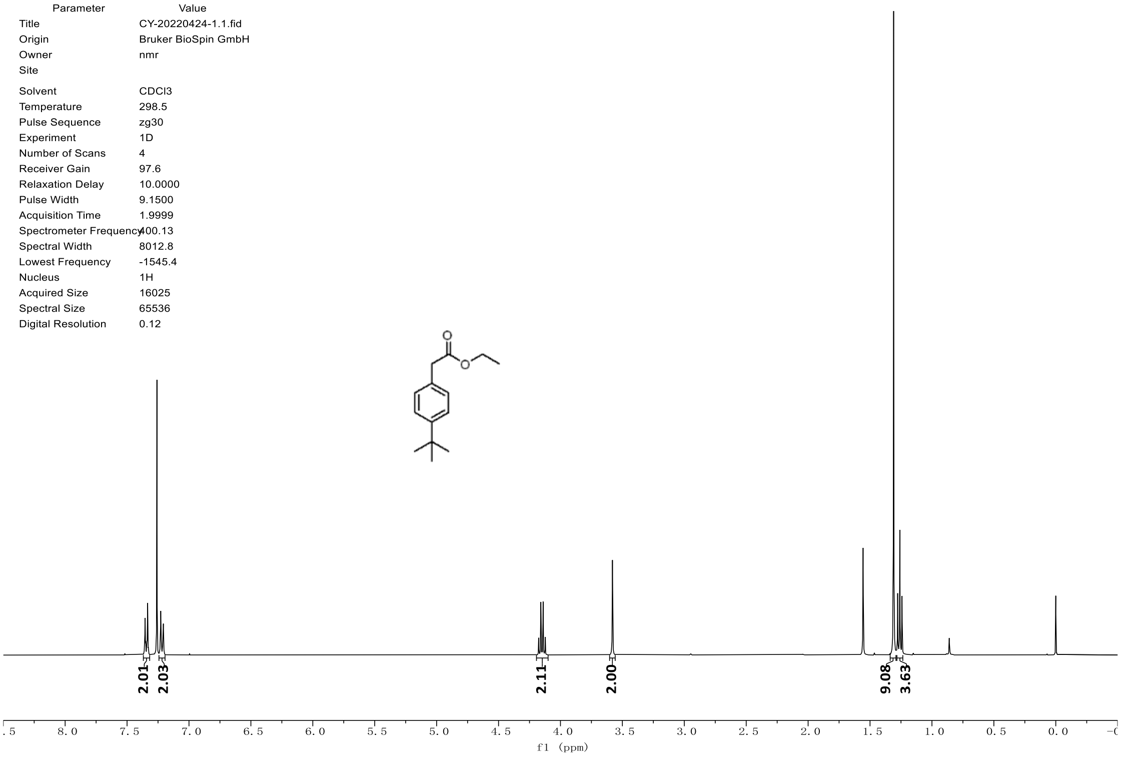

Supplementary Figure 129. Purified ^{1}H NMR of **2′q**

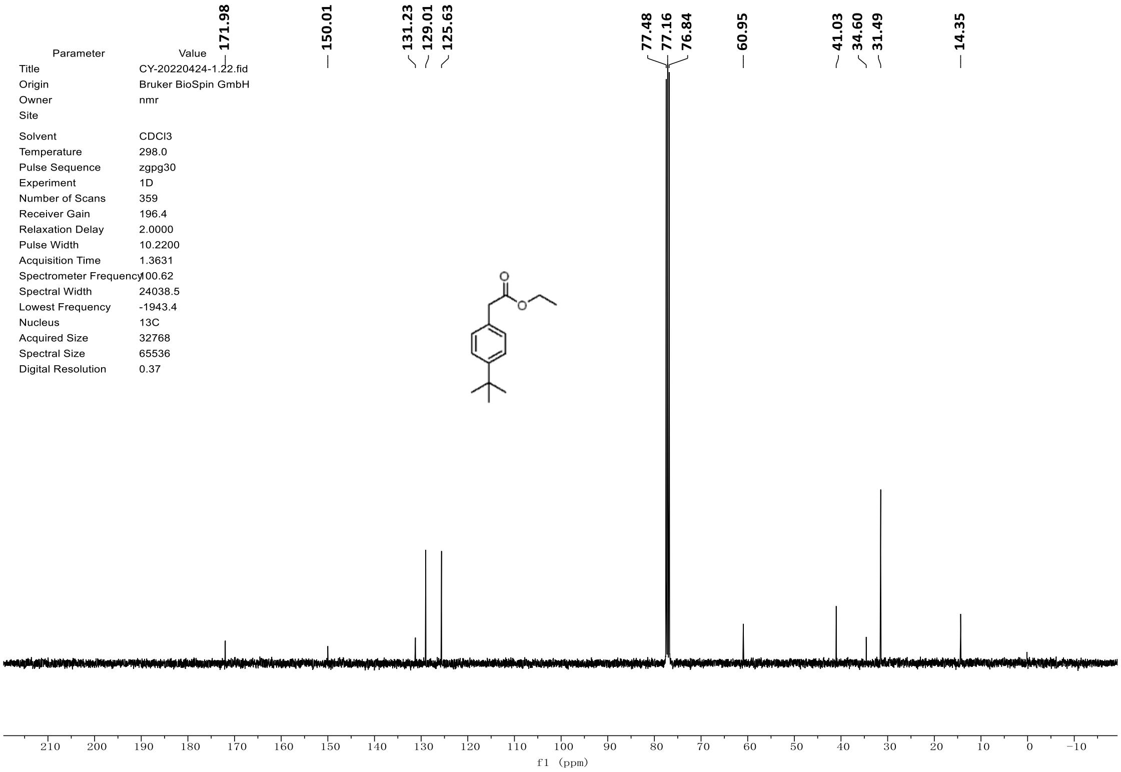

Supplementary Figure 130. Purified ^{13}C NMR of **2′q**

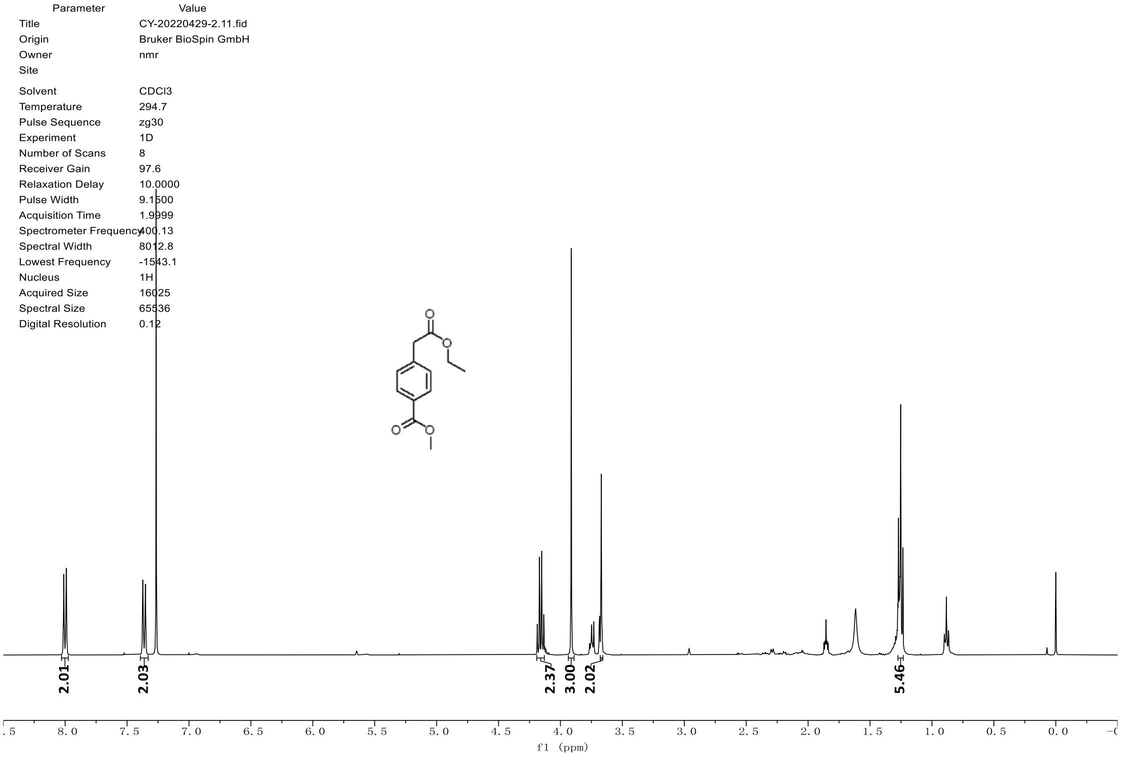

Supplementary Figure 131. Purified ^{1}H NMR of **2′r**

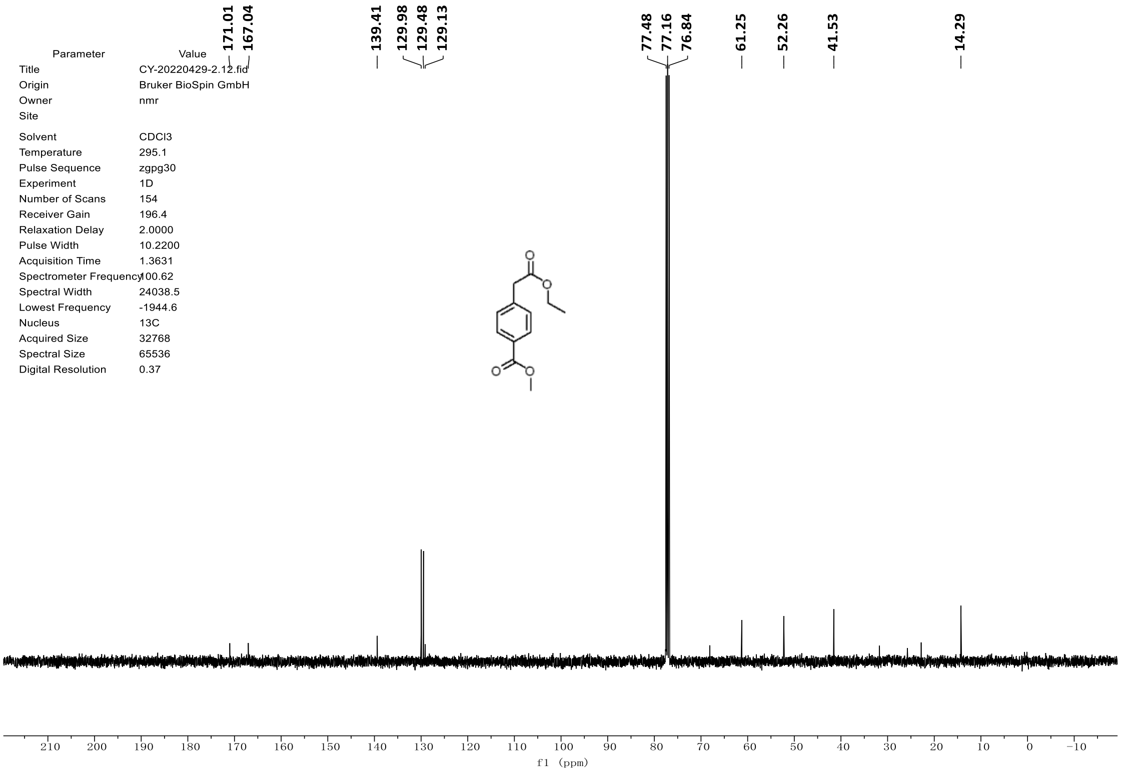

Supplementary Figure 132. Purified ^{13}C NMR of **2′r**

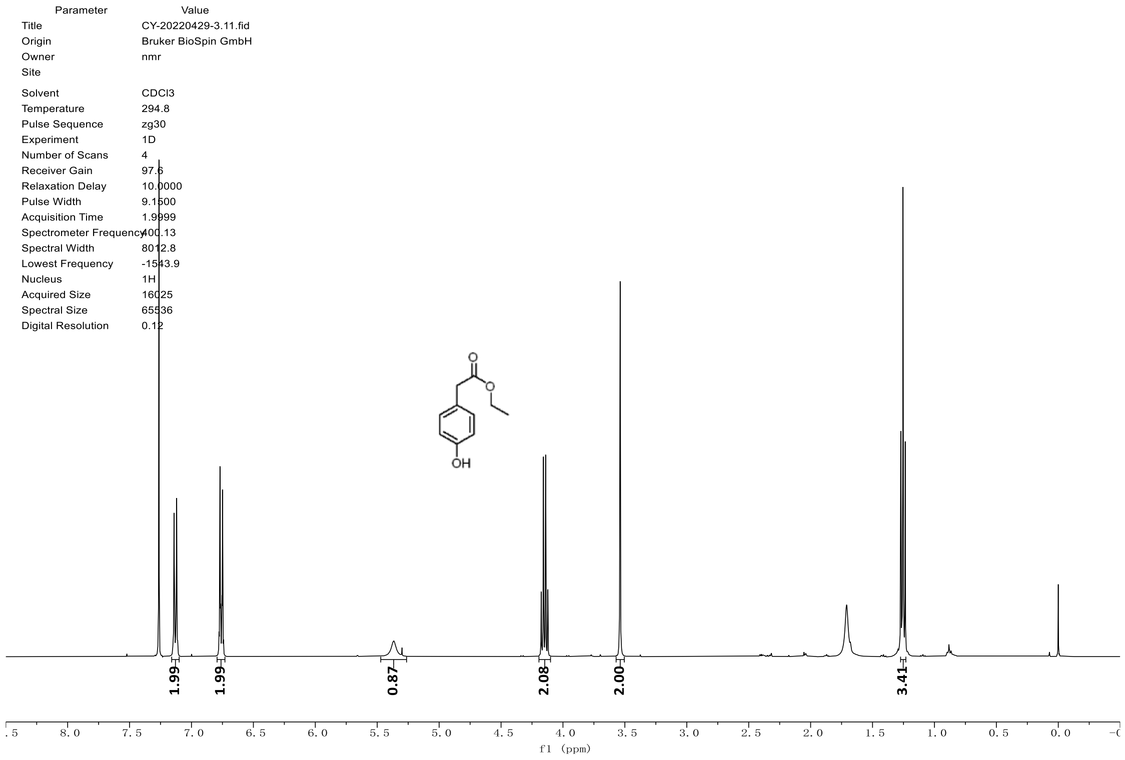

Supplementary Figure 133. Purified ^{1}H NMR of **2′s**

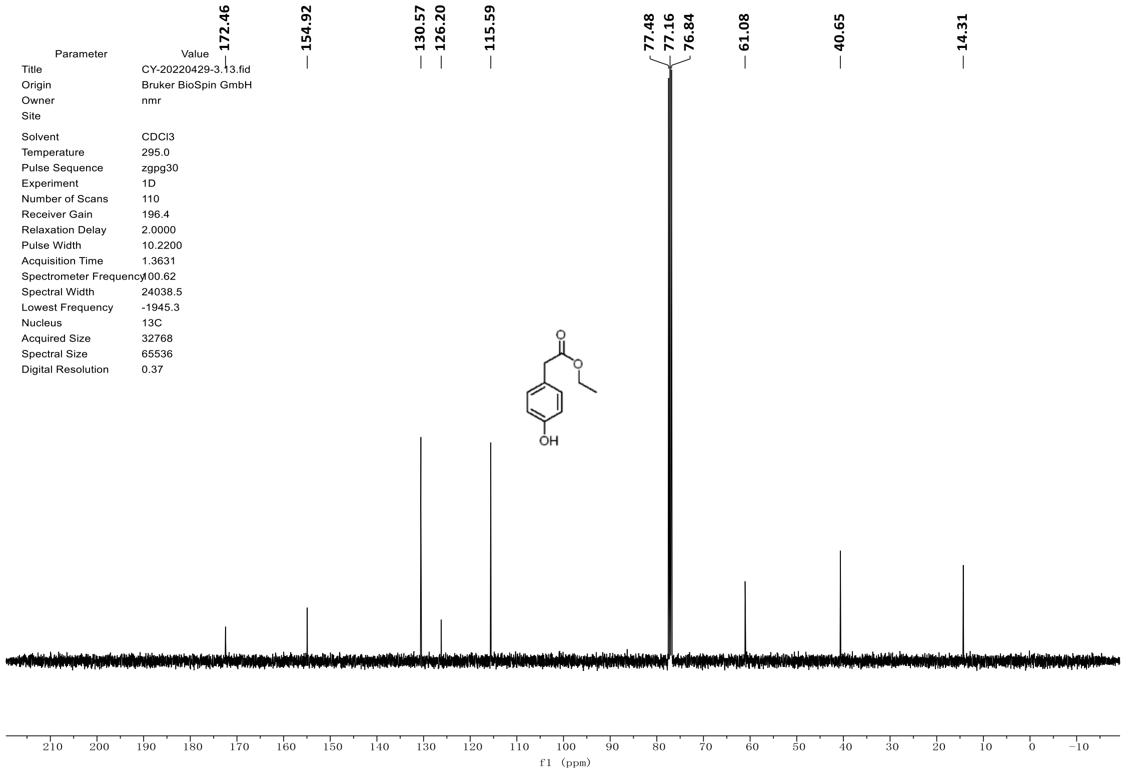

Supplementary Figure 134. Purified ^{13}C NMR of **2′s**

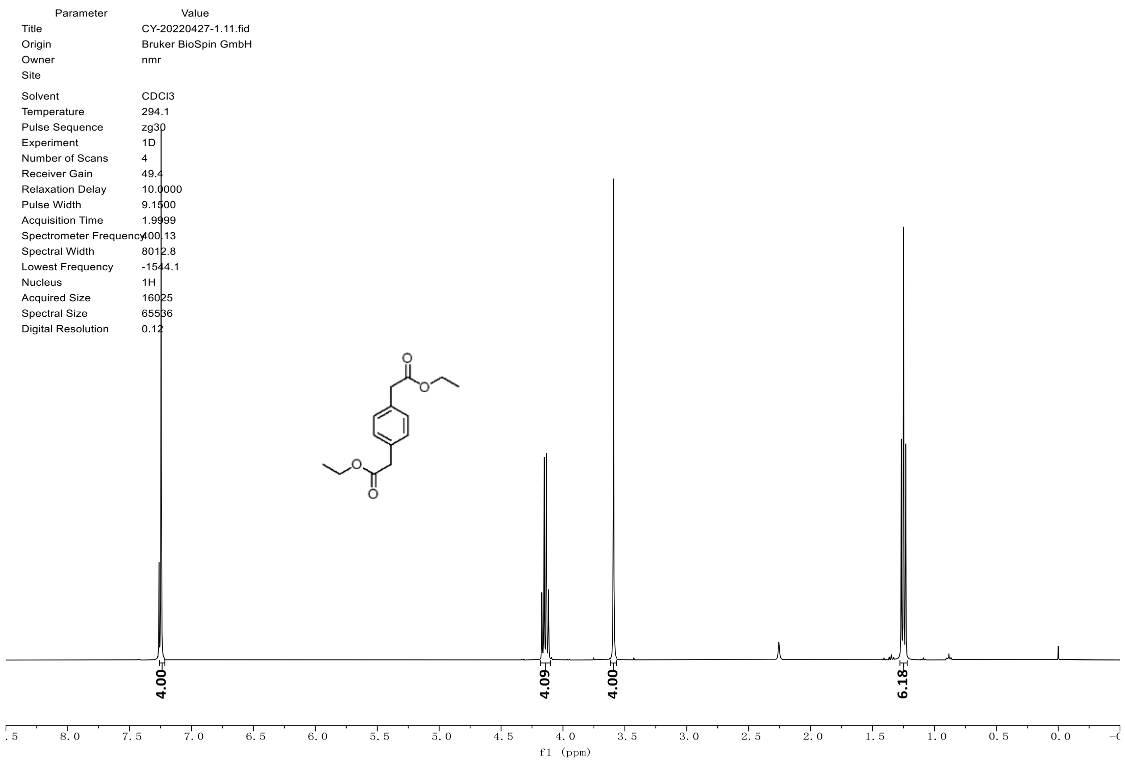

Supplementary Figure 135. Purified ^{1}H NMR of **2′t**

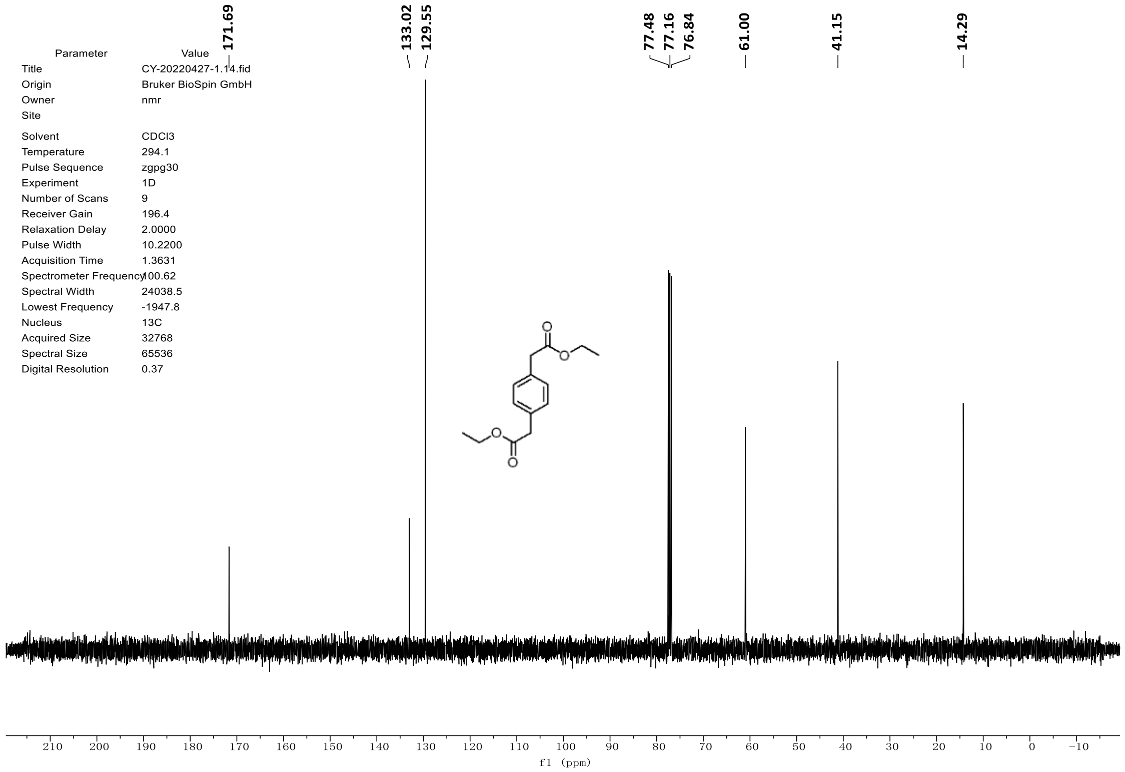

Supplementary Figure 136. Purified ^{13}C NMR of **2′t**

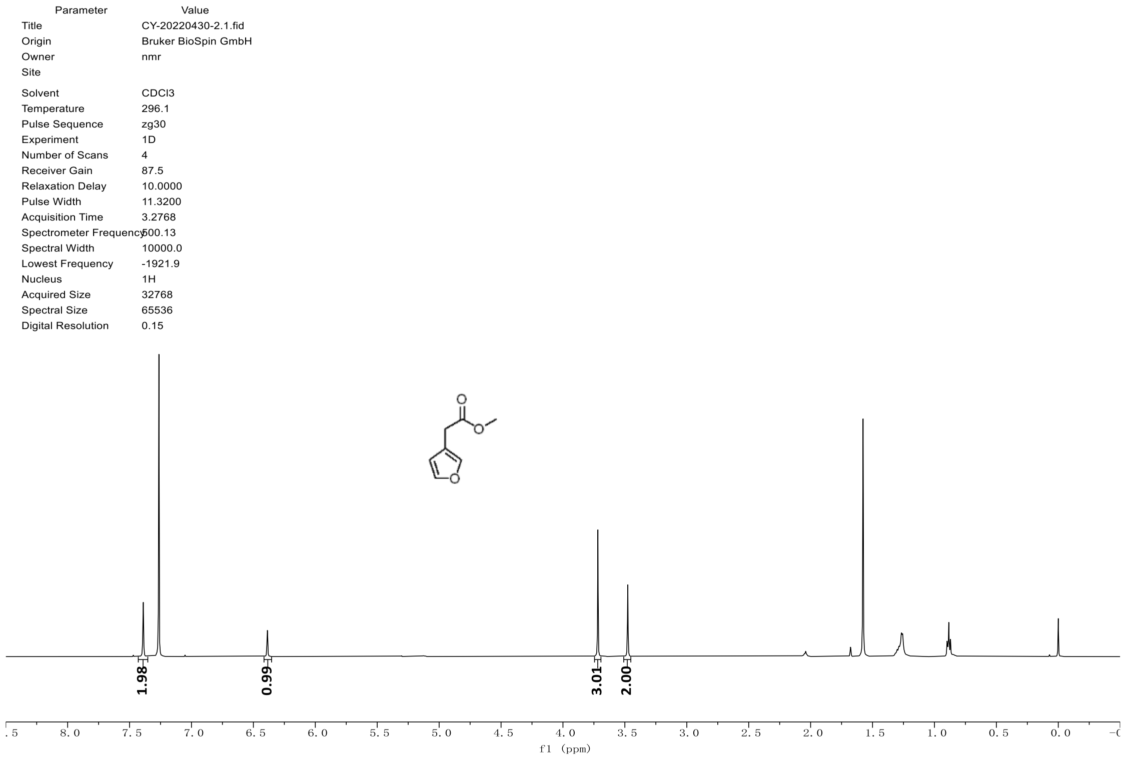

Supplementary Figure 137. Purified ^{1}H NMR of **2′u**

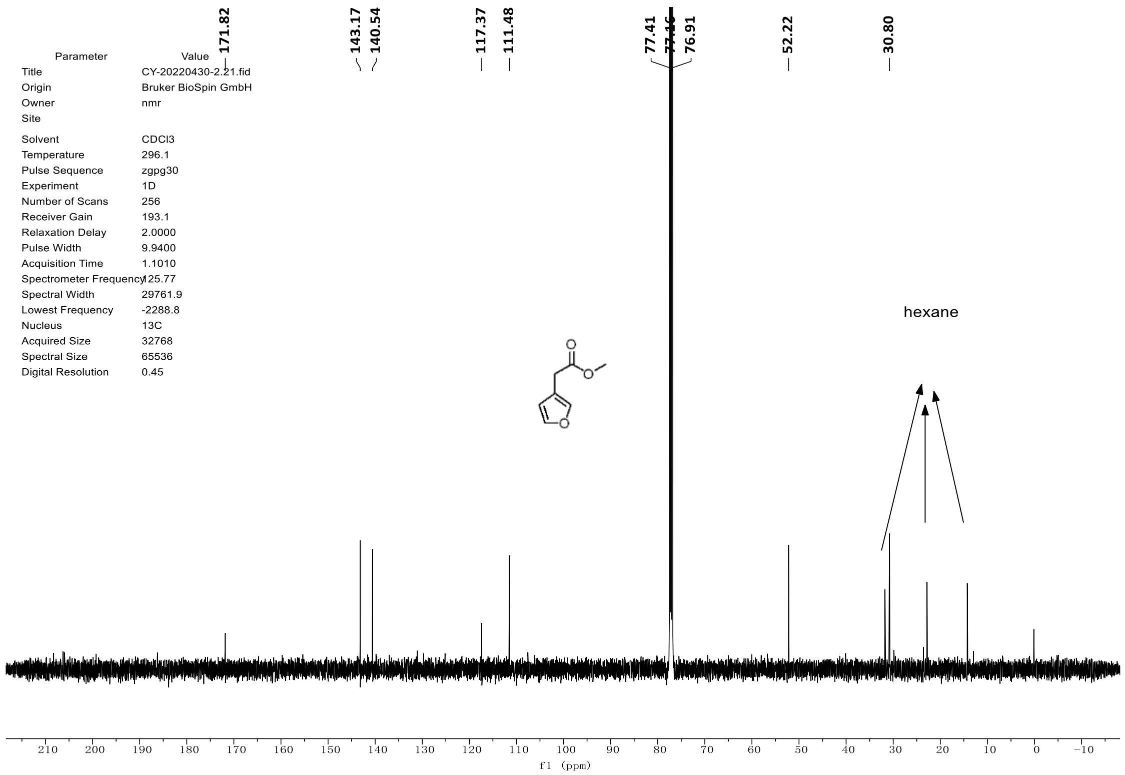

Supplementary Figure 138. Purified ^{13}C NMR of **2′u**

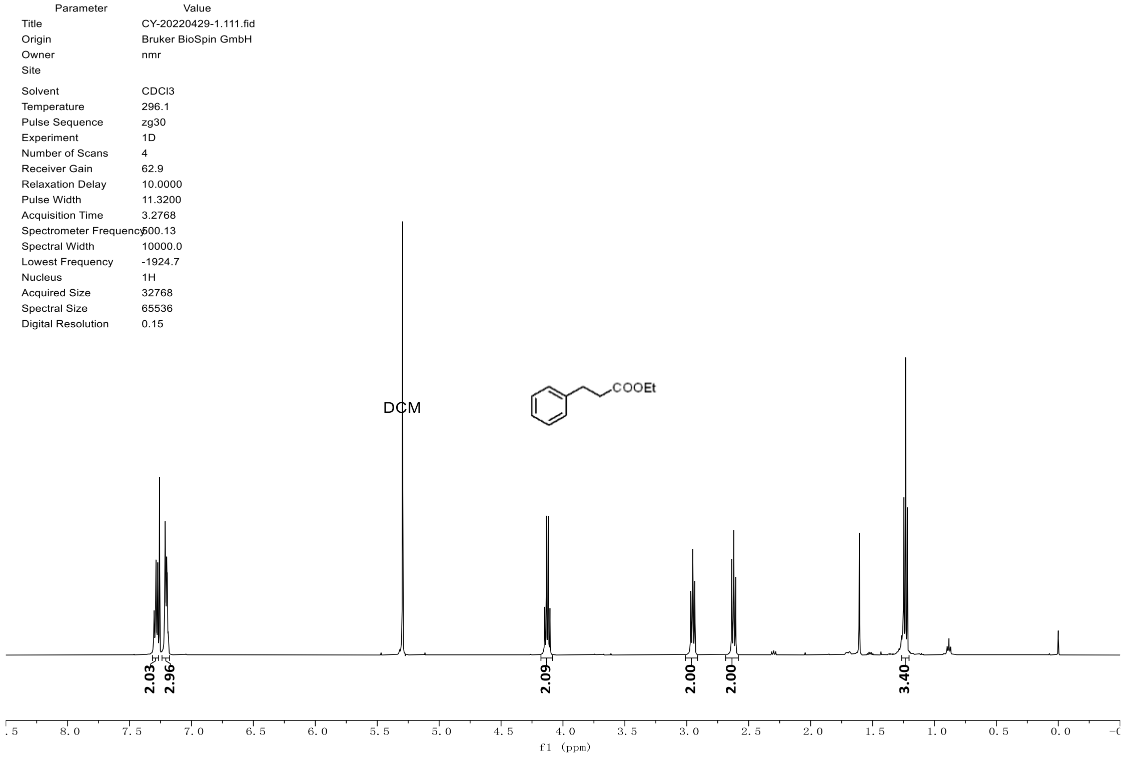

Supplementary Figure 139. Purified ^{1}H NMR of **2′v**

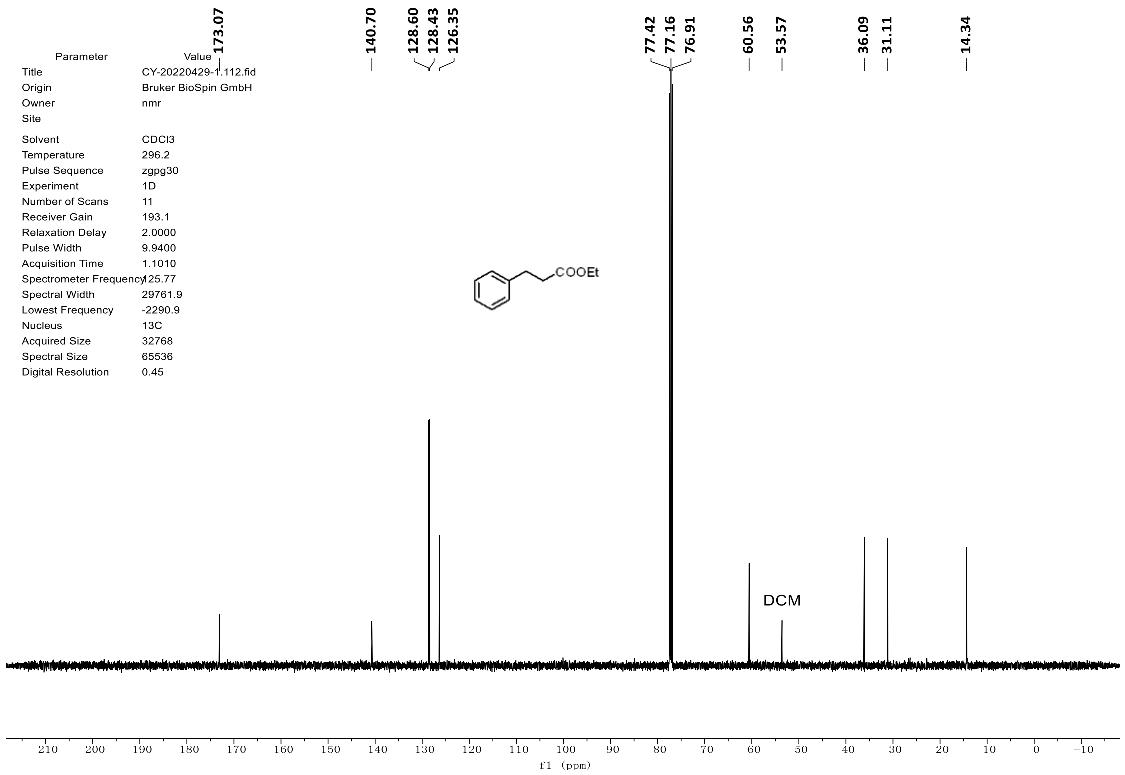

Supplementary Figure 140. Purified ^{13}C NMR of **2′v**

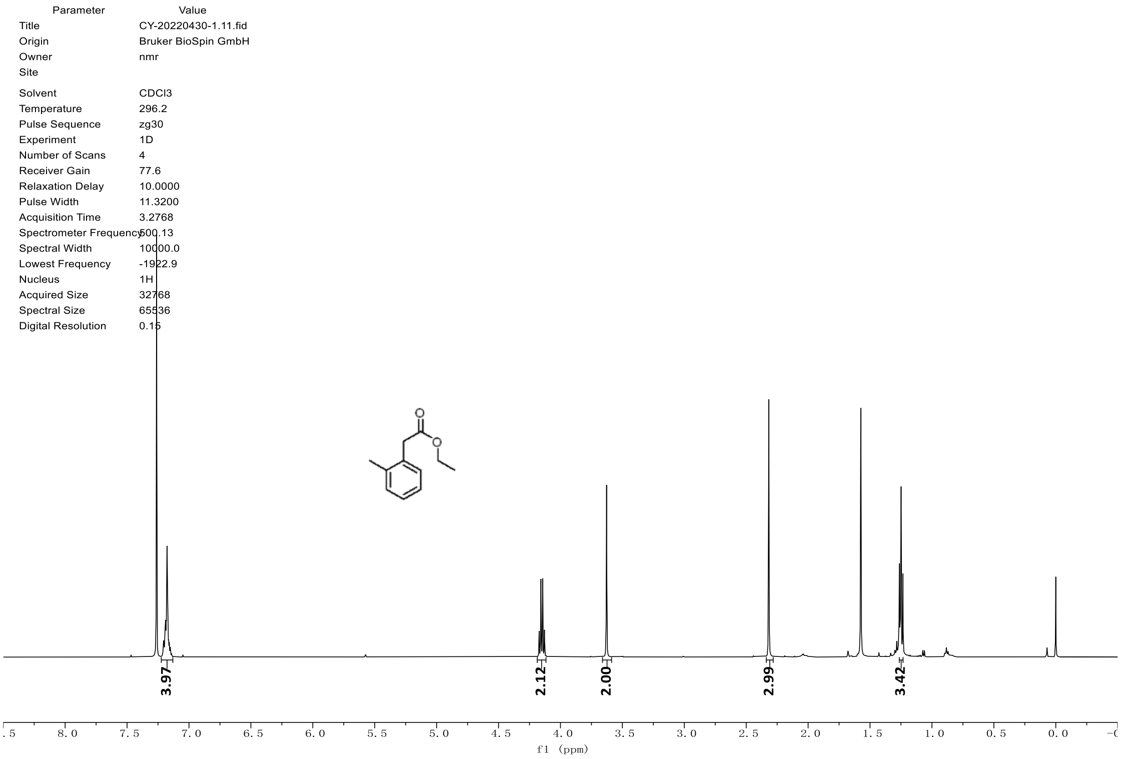

Supplementary Figure 141. Purified ^{1}H NMR of **2′x**

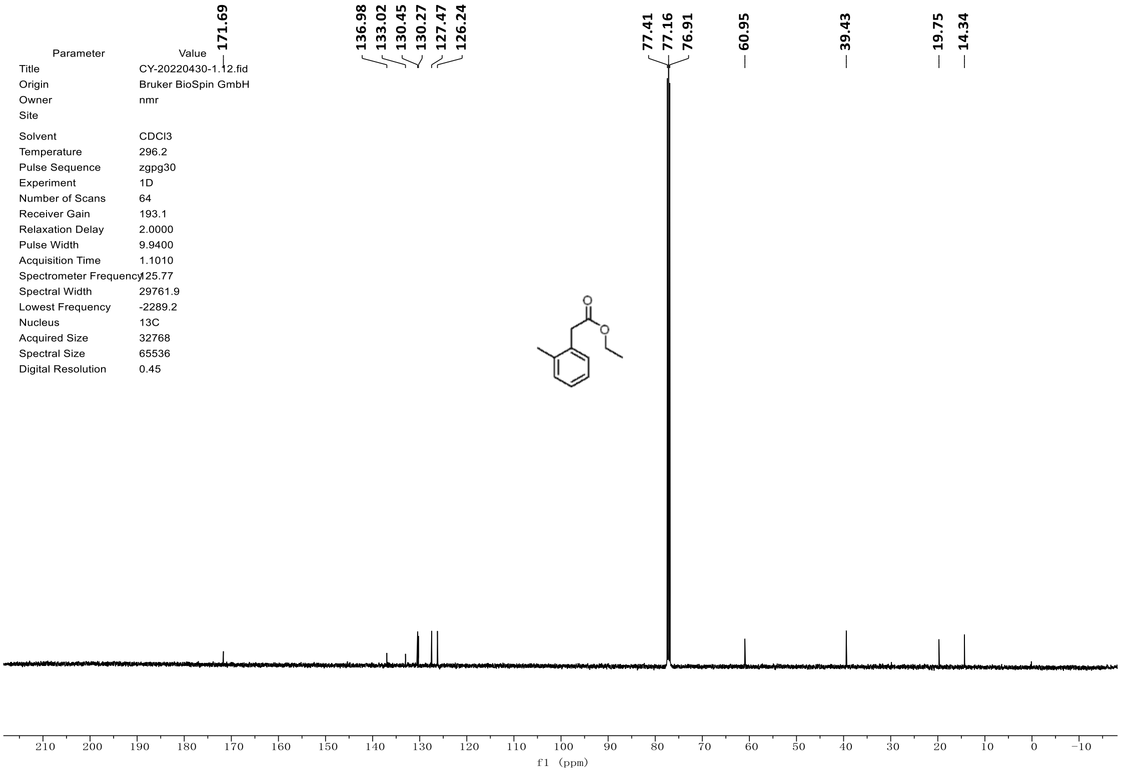

Supplementary Figure 142. Purified ^{13}C NMR of **2′x**

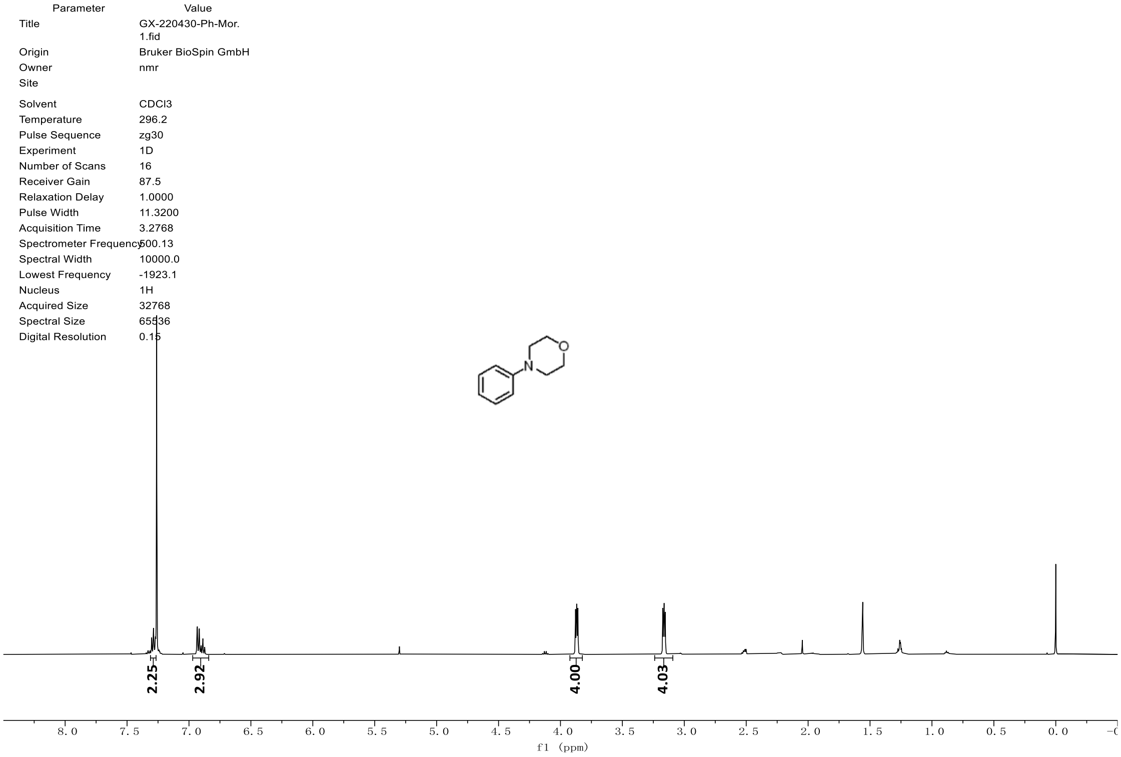

Supplementary Figure 143. Purified ^{1}H NMR of **2″a**

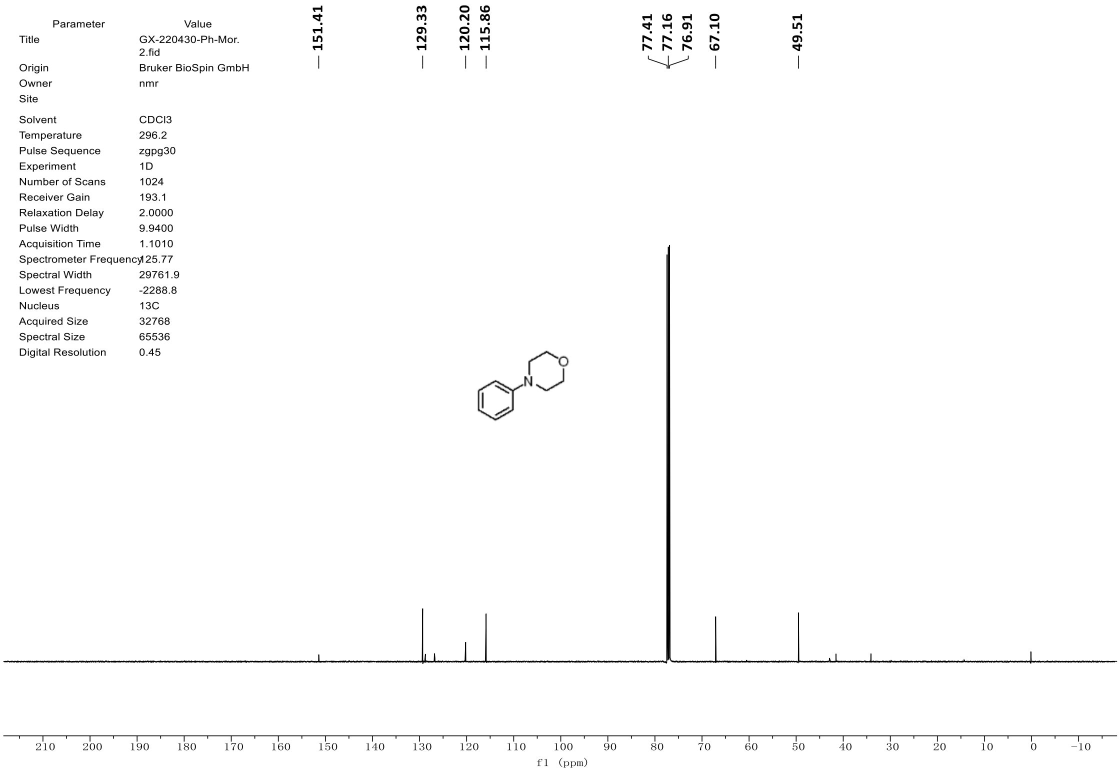

Supplementary Figure 144. Purified ^{13}C NMR of **2″a**

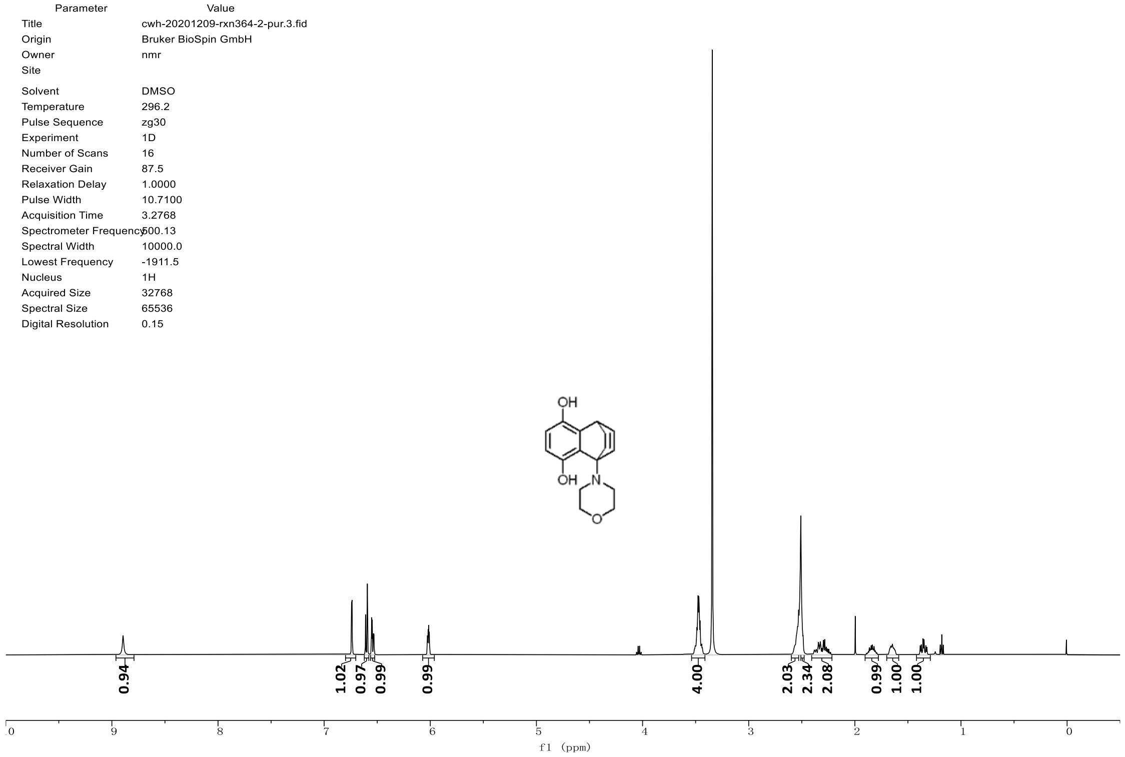

Supplementary Figure 145. Purified ^{1}H NMR of $\mathbf{2^{DA}a}$

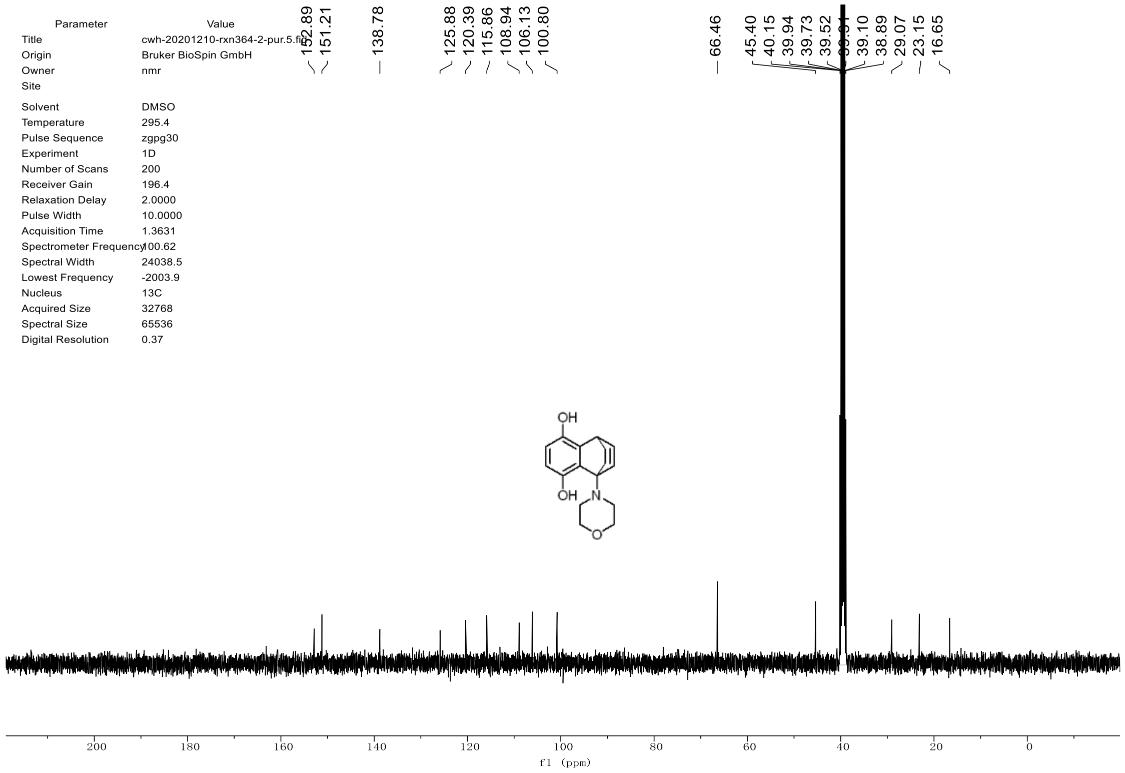

Supplementary Figure 146. Purified ^{13}C NMR of $\mathbf{2^{DA}a}$

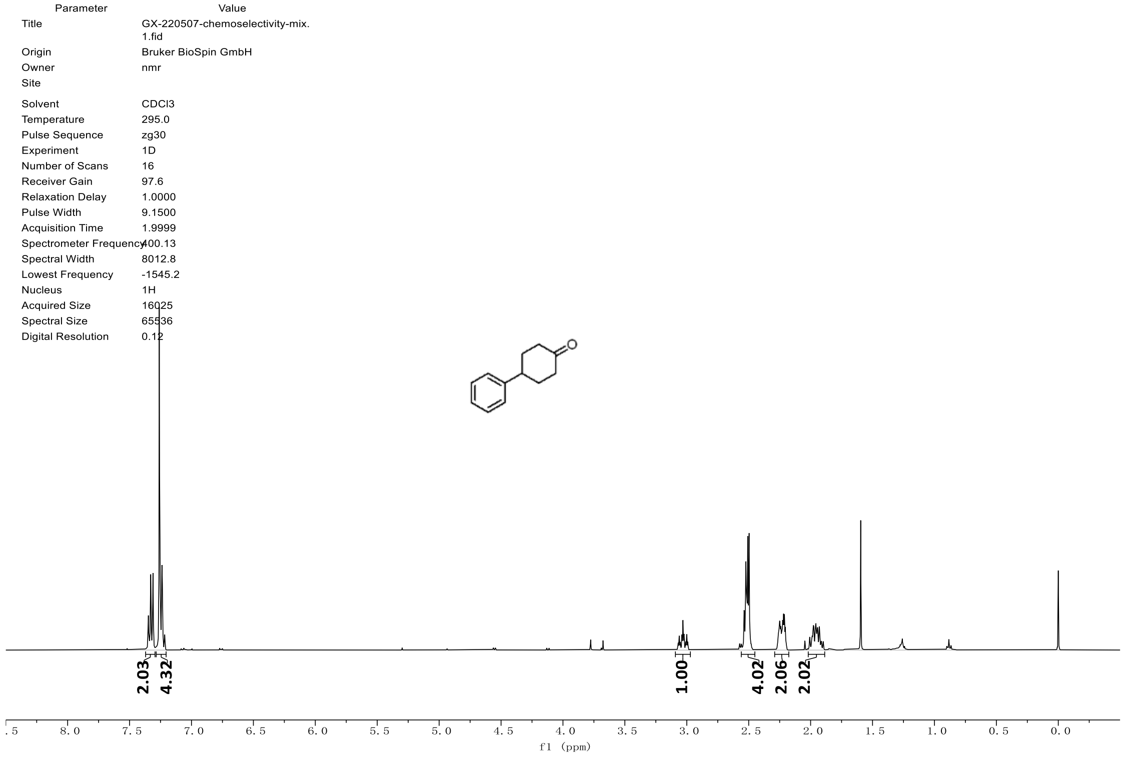

Supplementary Figure 147. Purified ^{1}H NMR of **2r**

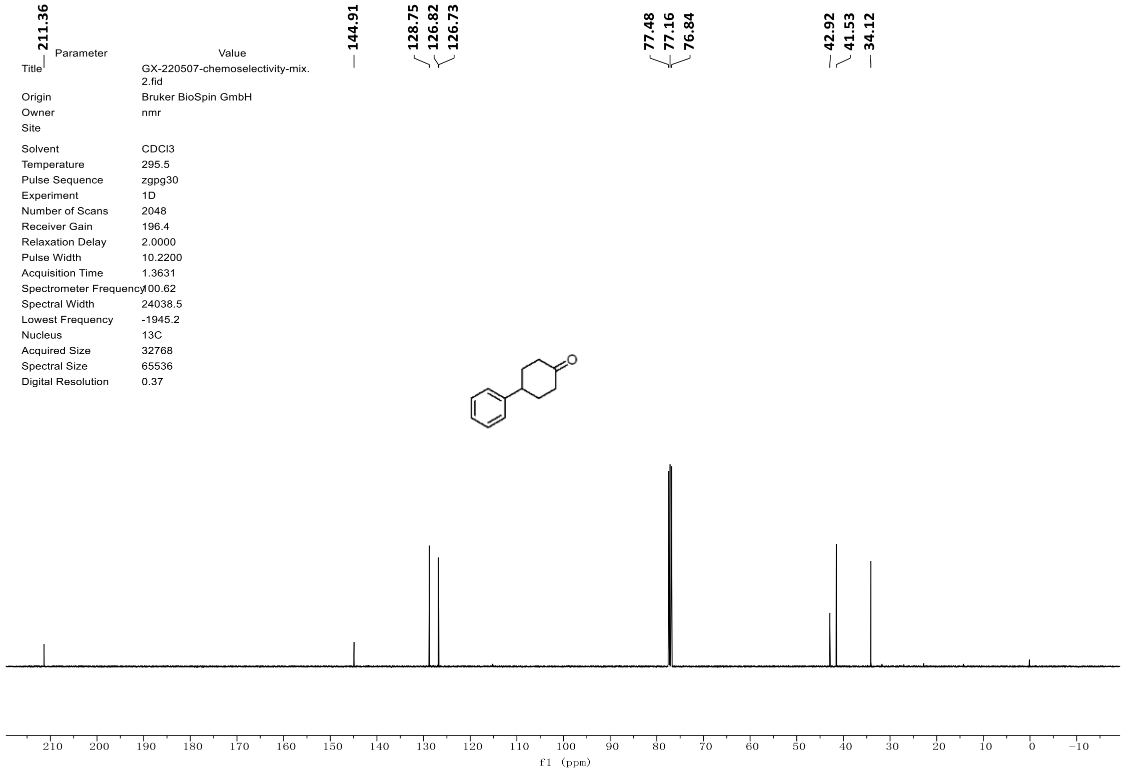

Supplementary Figure 148. Purified ^{13}C NMR of **2r**

Supplementary References

1. Elie M*, et al.* Designing NHC-Copper(I) Dipyridylamine Complexes for Blue Light-Emitting Electrochemical Cells. *ACS Appl Mater Interfaces* **8**, 14678-14691 (2016).

2. Altman RA, Shafir A, Choi A, Lichtor PA, Buchwald SL. An improved Cu-based catalyst system for the reactions of alcohols with aryl halides. *J Org Chem* **73**, 284-286 (2008).

3. Loudon GM, Radhakrishna AS, Almond MR, Blodgett JK, Boutin RH. Conversion of aliphatic amides into amines with [I,I-bis(trifluoroacetoxy)iodo]benzene. 1. Scope of the reaction. *The Journal of Organic Chemistry* **49**, 4272-4276 (1984).

4. Duvvuri K*, et al.* Cationic Co(I)-Intermediates for Hydrofunctionalization Reactions: Regio- and Enantioselective Cobalt-Catalyzed 1,2-Hydroboration of 1,3-Dienes. *J Am Chem Soc* **141**, 7365-7375 (2019).

5. Iwakura M, Tokura H, Tanino K. Construction of bicyclic systems containing an oxygen bridge by isomerization of cyclic epoxy alcohols. *Tetrahedron Lett* **58**, 1223-1226 (2017).

6. van Lint MJ, Hall M, Faber K, van Spanning RJM, Ruijter E, Orru RVA. Stereoselective Chemoenzymatic Cascade Synthesis of the bis-THF Core of Acetogenins. *Eur J Org Chem* **2019**, 1092-1101 (2019).

7. Tririya G, Zanger M. Synthesis of Anthracyclinone Precursor: 5,12 - Dihydroxy - 1,3,4 - trihydronaphthacene-2,6,11-quinone. *Synth Commun* **34**, 3047-3059 (2004).

8. Kapat A, Sperger T, Guven S, Schoenebeck F. E-Olefins through intramolecular radical relocation. *Science* **363**, 391-396 (2019).

9. Powers DC, Leber PA, Gallagher SS, Higgs AT, McCullough LA, Baldwin JE. Thermal chemistry of bicyclo[4.2.0]oct-2-enes. *J Org Chem* **72**, 187-194 (2007).

10. Takeuchi D, Watanabe K, Sogo K, Osakada K. Polymerization of Methylenecyclohexanes Catalyzed by Diimine–Pd Complex. Polymers Having trans- or cis-1,4- and trans-1,3-Cyclohexylene Groups. *Organometallics* **34**, 3007-3011 (2015).

11. Grygorenko OO, Gurbanov R, Sokolov A, Golovach S, Melnykov K, Dobrydnev AV. Synthesis of sp3-Enriched β-Fluoro Sulfonyl Chlorides. *Synthesis* **53**, 1771-1784 (2020).

12. Fuchs E, Keller M, Breit B. Phosphabarrelenes as Ligands in Rhodium - Catalyzed Hydroformylation of Internal Alkenes Essentially Free of Alkene Isomerization. *Chem Eur J* **12**, 6930-6939 (2006).

13. Reid WB, Watson DA. Synthesis of Trisubstituted Alkenyl Boronic Esters from Alkenes Using the Boryl-Heck Reaction. *Org Lett* **20**, 6832-6835 (2018).

14. Meng QY, Schirmer TE, Katou K, Konig B. Controllable Isomerization of Alkenes by Dual Visible-Light-Cobalt Catalysis. *Angew Chem Int Ed Engl* **58**, 5723-5728 (2019).

15. Zhang S, Bedi D, Cheng L, Unruh DK, Li G, Findlater M. Cobalt(II)-Catalyzed Stereoselective Olefin Isomerization: Facile Access to Acyclic Trisubstituted Alkenes. *J Am Chem Soc* **142**, 8910-8917 (2020).

16. Khan A, Silva LF, Rabnawaz M. Iodine(III)-Promoted Ring Expansion Reactions: A Metal-Free Approach toward Seven-Membered Heterocyclic Rings. *Asian Journal of Organic Chemistry* **10**, 2549-2552 (2021).

17. Vedejs E, Cabaj J, Peterson M. Wittig ethylidenation of ketones: reagent control of Z/E selectivity. *The Journal of Organic Chemistry* **58**, 6509-6512 (1993).

18. Sakagami K, Kumagai T, Taguchi T, Nakazato A. Scalable synthesis of (+)-2-amino-3-fluorobicyclo[3.1.0]hexane-2,6-dicarboxylic acid as a potent and selective group II metabotropic glutamate receptor agonist. *Chem Pharm Bull* **55**, 37-43 (2007).

19. Kwak HJ*, et al.* Synthesis and biological evaluation of aminobenzimidazole derivatives with a phenylcyclohexyl acetic acid group as anti-obesity and anti-diabetic agents. *Bioorg Med Chem Lett* **23**, 4713-4718 (2013).

20. Rodríguez M, Font G, Nadal‐Moradell J, Hernán‐Gómez A, Costas M. Iron‐Catalyzed Intermolecular Functionalization of Non‐Activated Aliphatic C−H Bonds via Carbene Transfer. *Advanced Synthesis & Catalysis* **362**, 5116-5123 (2020).

21. Agudo R, Roiban GD, Reetz MT. Induced axial chirality in biocatalytic asymmetric ketone reduction. *J Am Chem Soc* **135**, 1665-1668 (2013).

22. Itoh T*, et al.* Chemo-enzymatic synthesis of spiro type gem-difluorocyclopropane as core molecule candidate for liquid crystal compounds. *J Fluorine Chem* **130**, 1157-1163 (2009).

23. Ando K, Yamada K. Highly E-selective solvent-free Horner–Wadsworth–Emmons reaction catalyzed by DBU. *Green Chemistry* **13**, 1143-1146 (2011).

24. Gillard RM, Fernando JEM, Lupton DW. Enantioselective N-Heterocyclic Carbene Catalysis via the Dienyl Acyl Azolium. *Angew Chem Int Ed* **57**, 4712-4716 (2018).

25. Ahn H, Son I, Lee J, Lim HJ. Palladium−Hydride-Catalyzed Regiodivergent Isomerization of 1-Alkenes. *Asian Journal of Organic Chemistry* **6**, 335-341 (2017).

26. Magill AM, Yates BF, Cavell KJ, Skelton BW, White AH. Synthesis of N-heterocyclic carbene palladium(II) bis-phosphine complexes and their decomposition in the presence of aryl halides. *Dalton Trans* 3398-3406 (2007).

27. Esposito O, Lewis AK, Hitchcock PB, Caddick S, Cloke FG. Synthesis and reactivity of alkyl-palladium N-heterocyclic carbene complexes. *Chem Commun* 1157-1159 (2007).

28. Johns AM, Utsunomiya M, Incarvito CD, Hartwig JF. A highly active palladium catalyst for intermolecular hydroamination. Factors that control reactivity and additions of functionalized anilines to dienes and vinylarenes. *J Am Chem Soc* **128**, 1828-1839 (2006).

29. Chen YH, Grassl S, Knochel P. Cobalt-Catalyzed Electrophilic Amination of Aryl- and Heteroarylzinc Pivalates with N-Hydroxylamine Benzoates. *Angew Chem Int Ed* **57**, 1108-1111 (2018).

30. Laudadio G, *et al.* Selective C(sp(3))-H Aerobic Oxidation Enabled by Decatungstate Photocatalysis in Flow. *Angew Chem Int Ed* **57**, 4078-4082 (2018).

31. Lerebours R, Wolf C. Chemoselective nucleophilic arylation and single-step oxidative esterification of aldehydes using siloxanes and a palladium-phosphinous acid as a reaction switch. *J Am Chem Soc* **128**, 13052-13053 (2006).

32. Gowda RR, Chakraborty D. FeIII-Catalyzed Synthesis of Primary Amides from Aldehydes. *Eur J Org Chem* **2011**, 2226-2229 (2011).

33. Sergeev AG, Schulz T, Torborg C, Spannenberg A, Neumann H, Beller M. Palladium-catalyzed hydroxylation of aryl halides under ambient conditions. *Angew Chem Int Ed* **48**, 7595-7599 (2009).

34. Yang Y, Lin Y, Rao Y. Ruthenium(II)-catalyzed synthesis of hydroxylated arenes with ester as an effective directing group. *Org Lett* **14**, 2874-2877 (2012).

35. Taylor JE, Jones MD, Williams JM, Bull SD. Friedel-Crafts acylation of pyrroles and indoles using 1,5-diazabicyclo[4.3.0]non-5-ene (DBN) as a nucleophilic catalyst. *Org Lett* **12**, 5740-5743 (2010).

36. Murahashi S-I, *et al.* Ruthenium-Catalyzed Oxidative Dearomatization of Phenols to 4-(tert-Butylperoxy)cyclohexadienones: Synthesis of 2-Substituted Quinones from p-Substituted Phenols. *Eur J Org Chem* **2011**, 5355-5365 (2011).

37. Motoyama Y, Aoki M, Takaoka N, Aoto R, Nagashima H. Highly efficient synthesis of aldenamines from carboxamides by iridium-catalyzed silane-reduction/dehydration under mild conditions. *Chem Commun* 1574-1576 (2009).

38. Peng C, Zhang W, Yan G, Wang J. Arylation and vinylation of alpha-diazocarbonyl compounds with boroxines. *Org Lett* **11**, 1667-1670 (2009).

39. Rios-Lombardia N, Busto E, Garcia-Urdiales E, Gotor-Fernandez V, Gotor V. Enzymatic desymmetrization of prochiral 2-substituted-1,3-diamines: preparation of valuable nitrogenated compounds. *J Org Chem* **74**, 2571-2574 (2009).

40. Martínez R, Villarreal C. Synthesis of Novel Furo-, Thieno-, and Pyrroloazepines. *Synthesis* **2010**, 3346-3352 (2010).

41. Sohn SS, Bode JW. Catalytic generation of activated carboxylates from enals: a product-determining role for the base. *Org Lett* **7**, 3873-3876 (2005).

42. Tobisu M, Nakamura R, Kita Y, Chatani N. Rhodium-catalyzed reductive cleavage of carbon-cyano bonds with hydrosilane: a catalytic protocol for removal of cyano groups. *J Am Chem Soc* **131**, 3174-3175 (2009).

43. Brunel JM. Scope, limitations and mechanistic aspects in the selective homogeneous palladium-catalyzed reduction of alkenes under transfer hydrogen conditions. *Tetrahedron* **63**, 3899-3906 (2007).

44. Peng Z-Y, Wang J-P, Cheng J, Xie X-m, Zhang Z. Water works: an efficient palladium-catalyzed cross-coupling reaction between boronic acids and bromoacetate with aminophosphine ligand. *Tetrahedron* **66**, 8238-8241 (2010).

45. Zhang X, Yang C. Alkylations of Arylboronic Acids including Difluoroethylation/TrifluoroethylationviaNickel-Catalyzed Suzuki Cross-Coupling Reaction. *Advanced Synthesis & Catalysis* **357**, 2721-2727 (2015).

46. Fallon KJ*, et al.* Tyrian purple: an ancient natural dye for cross-conjugated n-type charge transport. *Journal of Materials Chemistry C* **9**, 4200-4205 (2021).

47. Bartoli S, De Nicola G, Roelens S. Binding of tetramethylammonium to polyether side-chained aromatic hosts. Evaluation of the binding contribution from ether oxygen donors. *J Org Chem* **68**, 8149-8156 (2003).

48. Arnold LA, Luo W, Guy RK. Synthesis of medium ring heterocycles using an intramolecular Heck reaction. *Org Lett* **6**, 3005-3007 (2004).

49. Ma W*, et al.* Direct construction of 2,3-unsubstituted benzofurans and benzothiophenes via a metal-free catalyzed intramolecular Friedel–Crafts reaction. *Organic Chemistry Frontiers* **6**, 493-497 (2019).

50. Pintori DG, Greaney MF. Oxidative C-H homodimerization of phenylacetamides. *Org Lett* **13**, 5713-5715 (2011).

51. Stalzer MM, Nicholas CP, Bhattacharyya A, Motta A, Delferro M, Marks TJ. Single-Face/All-cis Arene Hydrogenation by a Supported Single-Site d(0) Organozirconium Catalyst. *Angew Chem Int Ed Engl* **55**, 5263-5267 (2016).
